# Quantification of Difference in Nonselectivity Between In Vitro Diagnostic Medical Devices

**DOI:** 10.1002/bimj.70032

**Published:** 2025-01-02

**Authors:** Pernille Kjeilen Fauskanger, Sverre Sandberg, Jesper Johansen, Thomas Keller, Jeffrey Budd, W. Greg Miller, Anne Stavelin, Vincent Delatour, Mauro Panteghini, Bård Støve

**Affiliations:** ^1^ Norwegian Organization for Quality Improvement of Laboratory Examinations (Noklus) Haraldsplass Deaconess Hospital Bergen Norway; ^2^ Department of Mathematics University of Bergen Bergen Norway; ^3^ Department of Global Public Health and Primary Care University of Bergen Bergen Norway; ^4^ Department of Medical Biochemistry and Pharmacology Haukeland University Hospital Bergen Norway; ^5^ Radiometer Medical ApS Copenhagen Denmark; ^6^ ACOMED Statistic Leipzig Germany; ^7^ Jeff Budd Consulting St. Paul Minnesota USA; ^8^ Department of Pathology Virginia Commonwealth University Richmond Virginia USA; ^9^ Laboratoire national de métrologie et d'essais Paris France; ^10^ Department of Laboratory Medicine Ludwik Rydygier Collegium Medicum in Bydgoszcz Nicolaus Copernicus University in Torun Torun Poland

**Keywords:** differences in nonselectivity, method comparison, Monte Carlo simulations, plug‐in estimator, selectivity

## Abstract

Correct measurement results from in vitro diagnostic (IVD) medical devices (MD) are crucial for optimal patient care. The performance of IVD‐MDs is often assessed through method comparison studies. Such studies can be compromised by the influence of various factors. The effect of these factors must be examined in every method comparison study, for example, nonselectivity differences between compared IVD‐MDs are examined. Historically, selectivity or nonselectivity has been defined as a qualitative term. However, a quantification of nonselectivity differences between IVD‐MDs is needed. This paper fills this need by introducing a novel measure for quantifying differences in nonselectivity (DINS) between a pair of IVD‐MDs. Assuming one of the IVD‐MDs involved in the comparison exhibits high selectivity for the analyte, it becomes feasible to quantify nonselectivity in the other IVD‐MD by employing this DINS measure. Our approach leverages elements from univariate ordinary least squares regression and incorporates repeatability IVD‐MD variances, resulting in a normalized measure. We also introduce a plug‐in estimator for this measure, which is notably linked to the average relative increase in prediction interval widths attributable to DINS. This connection is exploited to establish a criterion for identifying excessive DINS utilizing a proof‐of‐hazard approach. Utilizing Monte Carlo simulations, we investigate how the estimator relates to population characteristics like DINS and heteroskedasticity. We find that DINS impacts the mean, variance, and 99th percentile of the estimator, while heteroskedasticity affects only the latter two, and to a considerably smaller extent compared to DINS. Importantly, the size of the study design modulates these effects. We also confirm, when using clinical data, that DINS between pairs of IVD‐MDs influence the estimator correspondingly to those of simulated data. Thus, the proposed estimator serves as an effective metric for quantifying DINS between IVD‐MDs and helping to determine the quality of a method comparison study.

## 1 Introduction

The evaluation of agreement limits between methods in comparison is a well‐established practice with extensive literature discussing the quantification of agreement between comparative methods. Various measures have been proposed to assess this agreement, including the mean square distance (MSD, Lin 2000) and the concordance correlation coefficient (CCC, Lin 1989). These measures aim to determine the extent to which measurements from one method align with those of another in a comparative analysis. Both parametric (Lin 1989, 2000) and nonparametric (Choudhary 2010) approaches have been developed for estimating these measures.

This paper, however, does not seek to directly quantify the closeness of agreement between compared methods. Instead, it aims to quantify the excess variability between measurements from the two methods in comparison, relative to the expected total variability attributed to the measurement error components (serving as a benchmark). Measures such as MSD and CCC are not suitable for this purpose, as they are sensitive to the magnitude of measurement error component variances and deviations from the diagonal regression model. In this context, the diagonal model is defined by a linear relationship with an intercept of 0 and a slope of 1.

In vitro diagnostic (IVD) medical devices (MD) used in medical laboratories produce a result for a measurand used to make a medical decision. Method comparisons are performed to evaluate if two IVD‐MDs produce equivalent results. When possible, results from an IVD‐MD in routine use are compared with results from a reference measurement procedure (CLSI EP09 2018). An important performance attribute is that the IVD‐MDs compared to measure the same measurand and that their results are exclusively dependent on the analyte, unaffected by other substances present in the sample matrix (e.g., whole blood, serum, urine, etc.). End‐user IVD‐MDs rarely include pretreatment to isolate the measurand from the matrix. Consequently, an important property for end‐user IVD‐MDs is selectivity for the measurand.

The definition of selectivity in VIM 2008 (VIM 2008) is “*Selectivity of a measurement procedure is a property whereby the measured value of a measurand is independent of other measurands or other quantities in the sample. Other measurands or quantities may be metabolites of the measurand or molecular forms of the measurand, other ions or molecules, or influences on the measurement from any source other than the measurand itself.”* The Eurochem Guide (Magnusson and Örnemark 2014) gives practical examples of how selectivity can be examined.

The selectivity of an IVD‐MD was formerly called analytical specificity. Medical laboratory end‐user IVD‐MDs should ideally have no or small nonselectivity for a measurand so that all IVD‐MDs measure the same quantity, and the results can be appropriately interpreted using decision values and reference intervals that reflect healthy or diseased conditions. An example of nonselectivity differences between IVD‐MDs can be seen in the varying measurement procedures for the liver enzyme alanine aminotransferase. Specifically, the omission of pyridoxal‐5‐phosphate (P‐5‐P) in the reagent by some IVD‐MDs leads to increased nonselectivity in comparison to those IVD‐MDs that include P‐5‐P. This discrepancy may result in a fluctuating underestimation of enzyme results, thereby obstructing the comparison and harmonization of results across different assays. The negative impact on assessing liver damage has been widely described (Panteghini et al. 2017; Panteghini 2024)

Evaluation of selectivity is an essential part of an IVD‐MD validation. Adequate selectivity is a requirement for using clinical outcome‐based decision values, for an IVD‐MD to be included in a harmonization protocol (ISO 21151 2020), for an IVD MD to be included in a commutability assessment (Miller et al. 2018; Sandberg et al. 2023), and to set a criterion for acceptable commutability of a reference material (Budd et al. 2018; Miller et al. 2023; Nilsson et al. 2018). Selectivity has been referred to as a qualitative entity (Theodorsson 2012). Historically, nonselectivity has been quantified through interference studies. However, such studies investigate potential interferents one at a time on a single IVD‐MD and frequently do not examine different molecular forms or metabolites of the measurand (CLSI EP07 2018). An approach is needed to quantify the combined effect of differences in nonselectivity (DINS) on the conclusion of method comparison studies for a pair of IVD‐MDs.

Assessment of DINS for the measurand should be an integral part in all studies where two IVD‐MDs are compared. This paper presents a model that quantifies DINS for a measurand measured using pairs of IVD‐MDs. Based on this measure, a plug‐in estimator is derived. The behavior of the plug‐in estimator is investigated using both simulated data and clinical data. This analysis aims to understand how the plug‐in estimator is influenced by DINS, as well as other factors such as heterogeneity in measurement variance. Furthermore, we examine how the size of the study design, specifically the number of clinical samples (CSs) and replicates, impacts the plug‐in estimator performance. Finally, the relationship between the plug‐in estimator and the average width of the pointwise ordinary least squares (OLS) prediction intervals are derived. This relationship is utilized to set an intuitive threshold value for what is deemed excessive DINS between compared IVD‐MDs, which ultimately depends on the scenario. This assessment includes when the evaluated pair of IVD‐MDs exhibits no DINS, demonstrates various forms and magnitudes of DINS, or exhibits heterogeneity in measurement variance.

## 2 Methods

### 2.1 Estimation of the DINS

We propose a criterion to detect excessive DINS within a comparison involving a pair of IVD‐MDs, denoted here as $\mathrm{IVD}-{\mathrm{MD}}_{X}$ and $\mathrm{IVD}-{\mathrm{MD}}_{Y}$. The DINS between the compared IVD‐MDs must first be quantified. Upon quantification of DINS, a criterion can be established to flag IVD‐MD comparisons exhibiting DINS exceeding tolerable limits. We define DINS as an attribute of two IVD‐MDs in a comparison, where the measurement results of CSs demonstrate deviations beyond those expected from random variation due to analytical uncertainty (e.g., variance) of the compared IVD‐MDs. Individual measurement results from $\mathrm{IVD}-{\mathrm{MD}}_{X}$ are represented as $\left\{x_{ir}:i\in \left\{1,\text{…},n\right\},r\in \left\{1,\text{…},R\right\}\right\}$. The corresponding measurement results from $\mathrm{IVD}-{\mathrm{MD}}_{Y}$ are denoted by $\left\{y_{ir}:i\in \left\{1,\text{…},n\right\},r\in \left\{1,\text{…},R\right\}\right\}$. The indices i and r indicate the CS identifier (ID) and replicate measurement ID, respectively. Furthermore, n is the total number of CSs and R is the total number of replicates performed on each individual CS. In addition, both $x_{ir}$ and $y_{ir}$ are assumed to be influenced by normally distributed measurement errors $h_{ir}$ and $v_{ir}$ for all (i,r)∈{1,…,n}×{1,…,R}, such that given the assumed latent values $\tau_{i}$ (for $x_{ir}$) and $\xi_{i}$ (for $y_{ir}$), we have that

$$\begin{array}{cc} x_{ir} & =\tau_{i}+h_{ir};\;h_{ir}∼N\left(0,\sigma_{\mathrm{IVD}-{\mathrm{MD}}_{X}}^{2}\right)\\ y_{ir} & =\xi_{i}+v_{ir};\;v_{ir}∼N\left(0,\sigma_{\mathrm{IVD}-{\mathrm{MD}}_{Y}}^{2}\right). \end{array}$$

Here, $\tau_{i}$ and $\xi_{i}$ are continuous random variables, signifying that both $x_{ir}$ and $y_{ir}$ are convolutions of the latent sample values and the measurement error components. The model assumes that $\tau_{i}∼\mathrm{unif}\left(\mathrm{cil},\mathrm{ciu}\right)$, where cil and ciu represent positive real numbers denoting to lower and upper concentration interval limits, respectively. The specific distributional assumption for $\tau_{i}$ is not critical, as demonstrated in the sensitivity analysis provided in the Supporting Information. The normality assumption for the measurement error components, $h_{ir}$ and $v_{ir}$, is based on the central limit theorem. This theorem applies because the measurement error effects in IVD‐MDs result from the sum of numerous independent random variables. The sensitivity analysis further reveals that the model used to quantify DINS is robust to this assumption. It is important to note that this model implicitly assumes the stability of both the n samples and the compared IVD‐MDs throughout the duration of the experiment.

The quantification of DINS between IVD‐MDs can be achieved by employing the variance of a regression model's error terms, utilizing the nR *pairs* ($x_{ir}$, $y_{ir}$) to fit the model. This variance captures the disparity in measurement results between the two IVD‐MDs. This is subsequently normalized by a suitable linear combination of $\sigma_{\mathrm{IVD}-{\mathrm{MD}}_{X}}^{2}$ and $\sigma_{\mathrm{IVD}-{\mathrm{MD}}_{Y}}^{2}$, which encapsulates the combined analytical uncertainty associated with both IVD‐MDs. Thus, consider the univariate OLS regression model derived by assuming the given model of $\left(x_{ir},y_{ir}\right)$ and $\xi_{i}=\beta_{0}+\beta_{1}\cdot \tau_{i}$,

(1) $$y_{ir}=\beta_{0}+\beta_{1}x_{ir}+e_{ir};e_{ir}∼N\left(0,\sigma^{2}\right).$$

In this context, $\beta_{0}$ and $\beta_{1}$ represent the OLS intercept and slope coefficients, respectively, while $e_{ir}$ denotes the model error terms. The choice to ground the quantification of DINS in the OLS regression model is driven by its ability to provide a comparatively straightforward formula. In contrast, alternative models such as Deming regression result in significantly more complex formulations. Notably, these intricate models do not necessarily offer proportionally improved statistical accuracy regarding quantification of DINS, making them less efficient options. The simplicity of OLS, therefore, offers a balance between formula complexity and accurate representation, thereby making it an adequate model for constructing the DINS measure. However, the OLS model might not fully account for measurement error variability inherent in IVD‐MD data, which can introduce bias and endogeneity issues if the measurement error variance in the predictor is large and exceeds the measurement error variance in the response variable. The bias magnitude is proportional to the ratio $\sigma_{\mathrm{IVD}-{\mathrm{MD}}_{X}}^{2}/\sigma_{\mathrm{IVD}-{\mathrm{MD}}_{Y}}^{2}$, with a greater ratio indicating a more biased OLS estimate. Therefore, to improve model accuracy, thereby optimizing the accuracy of the quantification of DINS, we recommend selecting the IVD‐MD with the smallest measurement error variance as the predictor. That is, if $\sigma_{\mathrm{IVD}-{\mathrm{MD}}_{X}}^{2}/\sigma_{\mathrm{IVD}-{\mathrm{MD}}_{Y}}^{2}>1$, it is be advisable to switch places of $y_{ir}$ and $x_{ir}$ in Equation (1), making $x_{ir}$ the response and $y_{ir}$ the predictor. By doing this, we manage to mitigate some of the OLS model bias in cases where $\sigma_{\mathrm{IVD}-{\mathrm{MD}}_{X}}^{2}/\sigma_{\mathrm{IVD}-{\mathrm{MD}}_{Y}}^{2}$ is large. This approach does not address inherent endogeneity‐attributed OLS model bias when both $\sigma_{\mathrm{IVD}-{\mathrm{MD}}_{X}}^{2}$ and $\sigma_{\mathrm{IVD}-{\mathrm{MD}}_{Y}}^{2}$ are large compared to $\mathrm{Var}[\tau_{i}]$ and $\sigma_{\mathrm{IVD}-{\mathrm{MD}}_{Y}}^{2}/\sigma_{\mathrm{IVD}-{\mathrm{MD}}_{X}}^{2}$ is close to 1. In practice, it is not uncommon for the ratio $\sigma_{\mathrm{IVD}-{\mathrm{MD}}_{Y}}^{2}/\sigma_{\mathrm{IVD}-{\mathrm{MD}}_{X}}^{2}$ to approach unity. However, the occurrence of $\sigma_{\mathrm{IVD}-{\mathrm{MD}}_{X}}^{2},\sigma_{\mathrm{IVD}-{\mathrm{MD}}_{Y}}^{2}>\mathrm{Var}\left[\tau_{i}\right]$ is exceedingly rare. Consequently, the strategy of interchanging the roles of $y_{ir}$ and $x_{ir}$ proves to be an adequate method for addressing endogeneity issues stemming from measurement error in the predictor variable. We assume that $\sigma_{\mathrm{IVD}-{\mathrm{MD}}_{Y}}^{2}/\sigma_{\mathrm{IVD}-{\mathrm{MD}}_{X}}^{2}\geq 1$ in the rest of this section, which entails that $x_{ir}$ is used as the predictor variable and $y_{ir}$ is used as the response variable, without loss of generality. Indeed, the derivations in the remaining part of this section will be methodically identical if $x_{ir}$ was the response variable and $y_{ir}$ was the predictor variable. The only difference is that $x_{ir}$ and $\sigma_{\mathrm{IVD}-{\mathrm{MD}}_{X}}^{2}$ change places with $y_{ir}$ and $\sigma_{\mathrm{IVD}-{\mathrm{MD}}_{Y}}^{2}$, respectively.

The methodology for quantifying the DINS between a pair of IVD‐MDs is grounded in the bias–variance trade‐off principle. This principle articulates that the mean squared prediction error (MSPE) of a new observation (in this instance, a new measurement result $y_{n+1,1}$) can be expressed as the sum of three statistical components: statistical squared model bias, model variance, and $\sigma^{2}$ (i.e., irreducible variance) (Hastie, Tibshirani, and Friedman 2009, 223). The first two terms, statistical squared model bias and model variance, serve to quantitatively determine the absolute magnitude of DINS between the IVD‐MDs being compared. Given the OLS prediction of $y_{n+1,1}$, denoted as ${\hat{y}}_{n+1,1}$, the MSPE can be expressed as follows:

$$\begin{array}{cc} \mathrm{MSPE}[y_{n+1,1}]=\; & E\left[{\left(y_{n+1,1}-{\hat{y}}_{n+1,1}\right)}^{2}\right]=E{\left[y_{n+1,1}-{\hat{y}}_{n+1,1}\right]}^{2}\\ & +\mathrm{Var}\left[{\hat{y}}_{n+1,1}\right]+\sigma^{2}. \end{array}$$

The OLS regression model is unbiased (Devore, Berk, and Carlton 2021, 738) provided that the model predictor is assumed measured without measurement error. In our case, both $x_{ir}$ and $y_{ir}$ are influenced by measurement error. However, if we ensure that the predictor is assigned to the measurements of the IVD‐MD in the IVD‐MD comparison with the smallest measurement error variance, we can state that the OLS regression model is approximately unbiased, implying that

$$\mathrm{MSPE}\left[y_{n+1,1}\right]\approx \mathrm{Var}\left[y_{n+1,1}-{\hat{y}}_{n+1,1}\right]=\mathrm{Var}\left[{\hat{y}}_{n+1,1}\right]+\sigma^{2}.$$

Consequently, a theoretical normalized quantification of DINS, represented as ζ, is defined in the following manner:

$$\zeta =\frac{\mathrm{Var}\left[y_{n+1,1}-{\hat{y}}_{n+1,1}\right]}{\sigma_{\mathrm{IVD}-{\mathrm{MD}}_{Y}}^{2}+\beta_{1}^{2}\sigma_{\mathrm{IVD}-{\mathrm{MD}}_{X}}^{2}}=\frac{\mathrm{Var}\left[{\hat{y}}_{n+1,1}\right]+\sigma^{2}}{\sigma_{\mathrm{IVD}-{\mathrm{MD}}_{Y}}^{2}+\beta_{1}^{2}\sigma_{\mathrm{IVD}-{\mathrm{MD}}_{X}}^{2}}.$$

Employing the OLS regression model, for i=1,…,n, the variance of the prediction error $y_{n+1,1}-{\hat{y}}_{n+1,1}$ can be estimated via the mean estimated prediction error variance across all observed measurement results $\left(x_{ir},y_{ir}\right)$

(2) $$\begin{array}{cc} \hat{\mathrm{Var}}\left[y_{n+1,1}-{\hat{y}}_{n+1,1}\right] & =\frac{1}{nR}\sum_{i=1}^{n}\sum_{r=1}^{R}\hat{\mathrm{Var}}\left[y_{ir}-{\hat{y}}_{ir}\right]\\ & =\frac{1}{nR}\sum_{i=1}^{n}\sum_{r=1}^{R}S^{2}\cdot \left(1+\frac{1}{nR}+\frac{{\left(x_{ir}-\bar{\bar{x}}\right)}^{2}}{\sum_{i=1}^{n}\sum_{r=1}^{R}{\left(x_{ir}-\bar{\bar{x}}\right)}^{2}}\right)\\ & =\frac{S^{2}}{nR}\left(nR+1+\frac{\sum_{i=1}^{n}\sum_{r=1}^{R}{\left(x_{ir}-\bar{\bar{x}}\right)}^{2}}{\sum_{i=1}^{n}\sum_{r=1}^{R}{\left(x_{ir}-\bar{\bar{x}}\right)}^{2}}\right)\\ & =S^{2}\cdot \frac{nR+2}{nR}. \end{array}$$

$S^{2}$ serves as an estimator of $\sigma^{2}$ (Devore, Berk, and Carlton 2021, 718), which accounts for $\sigma_{\mathrm{IVD}-{\mathrm{MD}}_{Y}}^{2}+\beta_{1}^{2}\sigma_{\mathrm{IVD}-{\mathrm{MD}}_{X}}^{2}$ exclusively in instances where a pair of IVD‐MDs exhibit identical nonselectivity profiles. In any other scenarios, $\sigma^{2}$ accounts for both $\sigma_{\mathrm{IVD}-{\mathrm{MD}}_{Y}}^{2}+\beta_{1}^{2}\sigma_{\mathrm{IVD}-{\mathrm{MD}}_{X}}^{2}$ and any additional variability arising from DINS. We thus present the closed‐form plug‐in estimator of ζ as follows:

(3) $$\hat{\zeta }=\frac{\hat{\mathrm{Var}}\left[y_{n+1,1}-{\hat{y}}_{n+1,1}\right]}{{\hat{\sigma }}_{\mathrm{IVD}-{\mathrm{MD}}_{Y}}^{2}+\beta_{1}^{2}{\hat{\sigma }}_{\mathrm{IVD}-{\mathrm{MD}}_{X}}^{2}}=\frac{S^{2}\cdot \frac{nR+2}{nR}}{{\hat{\sigma }}_{\mathrm{IVD}-{\mathrm{MD}}_{Y}}^{2}+{\hat{\beta }}_{1}^{2}{\hat{\sigma }}_{\mathrm{IVD}-{\mathrm{MD}}_{X}}^{2}},$$

where ${\hat{\sigma }}_{\mathrm{IVD}-{\mathrm{MD}}_{X}}^{2}$, ${\hat{\sigma }}_{\mathrm{IVD}-{\mathrm{MD}}_{Y}}^{2}$, and ${\hat{\beta }}_{1}$ are estimators for $\sigma_{\mathrm{IVD}-{\mathrm{MD}}_{X}}^{2}$, $\sigma_{\mathrm{IVD}-{\mathrm{MD}}_{Y}}^{2}$, and $\beta_{1}$, respectively. The two former estimators are statistically unbiased. The latter is approximately unbiased if IVD‐MD measurement results are assigned appropriately as predictor and response variables. Given the assumption that the variance of measurement errors is constant across the entire concentration interval, ${\hat{\sigma }}_{\mathrm{IVD}-{\mathrm{MD}}_{X}}^{2}$ and ${\hat{\sigma }}_{\mathrm{IVD}-{\mathrm{MD}}_{Y}}^{2}$ are computed utilizing the pooled within‐CS variances over all unique CSs:

$$\begin{array}{cc} {\hat{\sigma }}_{\mathrm{IVD}-{\mathrm{MD}}_{X}}^{2} & =\frac{1}{n}\sum_{i=1}^{n}\frac{1}{R-1}\sum_{r=1}^{R}{\left(x_{ir}-{\bar{x}}_{i}\right)}^{2}\\ {\hat{\sigma }}_{\mathrm{IVD}-{\mathrm{MD}}_{Y}}^{2} & =\frac{1}{n}\sum_{i=1}^{n}\frac{1}{R-1}\sum_{r=1}^{R}{\left(y_{ir}-{\bar{y}}_{i}\right)}^{2}. \end{array}$$

Here, ${\bar{x}}_{i}$ and ${\bar{y}}_{i}$ represent the replicate averages for CSs with an ID of i. ${\hat{\beta }}_{1}$ is simply the OLS estimator of the slope coefficient $\beta_{1}$ (Devore, Berk, and Carlton 2021, 715). Upon introducing the abbreviation for the estimated variance of the prediction error, denoted as $S_{P}^{2}$ (where “P” stands for prediction), given by $S^{2}\cdot \left(nR+2\right)/nR$, the equation for $\hat{\zeta }$ can be expressed as follows:

(4) $$\hat{\zeta }=\frac{S_{P}^{2}}{{\hat{\sigma }}_{\mathrm{IVD}-{\mathrm{MD}}_{Y}}^{2}+{\hat{\beta }}_{1}^{2}{\hat{\sigma }}_{\mathrm{IVD}-{\mathrm{MD}}_{X}}^{2}}.$$

In absence of DINS within a pair of IVD‐MDs, it is expected that the estimator $\hat{\zeta }$ will closely approximate a value of 1, because S estimates $\sigma_{\mathrm{IVD}-{\mathrm{MD}}_{Y}}^{2}+\beta_{1}^{2}\sigma_{\mathrm{IVD}-{\mathrm{MD}}_{X}}^{2}$ in this case. In the context of linear regression performed on IVD‐MD comparisons, it is generally anticipated that the value of ${\hat{\beta }}_{1}$ approximates 1. Based on this supposition, we infer that the formula for $\hat{\zeta }$—a relative measure of DINS—can be refined by assuming ${\hat{\beta }}_{1}=1$. While we must recognize the potential effect a ${\hat{\beta }}_{1}$ value deviating markedly from 1 might have on $\hat{\zeta }$, such substantial divergences are infrequently observed in practice. Consequently, if the assumption ${\hat{\beta }}_{1}=1$ holds reasonably well, we can streamline the $\hat{\zeta }$ equation without causing considerable compromise to its statistical accuracy

(5) $$\hat{\zeta }=\frac{S_{P}^{2}}{{\hat{\sigma }}_{\mathrm{IVD}-{\mathrm{MD}}_{X}}^{2}+{\hat{\sigma }}_{\mathrm{IVD}-{\mathrm{MD}}_{Y}}^{2}}.$$

While Equation (5) can be an appropriate alternative to Equation (4) used for estimating ζ, it is important to note that when the values of ${\hat{\beta }}_{1}$ deviate from 1 can lead to biased estimates of ζ. For ${\hat{\beta }}_{1}<1$, it tends to produce larger values of $\hat{\zeta }$ compared to Equation (4), and conversely, for ${\hat{\beta }}_{1}>1$, it tends to produce smaller $\hat{\zeta }$ values. To substantiate these claims, we simulated 100 simulated data sets with n=25, R=3, IVD‐MD measurement error coefficients of variation ${\mathrm{CV}}_{\mathrm{IVD}-{\mathrm{MD}}_{X}}=2\%$ and ${\mathrm{CV}}_{\mathrm{IVD}-{\mathrm{MD}}_{Y}}=1\%$, and $\beta_{1}=0.75$. For each simulated data set, we estimated ζ using both estimators. The most pronounced discrepancies in $\hat{\zeta }$ among these 100 data sets were when (4) and (5) produced $\hat{\zeta }$ values of 0.998 and 1.233, respectively. For $\beta_{1}=1.25$, the most pronounced discrepancies in $\hat{\zeta }$ among the 100 associated data sets were when (4) and (5) produced $\hat{\zeta }$ values of 1.037 and 0.892. While it may appear that $\beta_{1}<1$ yields larger discrepancies in $\hat{\zeta }$ than $\beta_{1}>1$, this relationship is multifaceted. It is predominately influenced by the ratio between ${\mathrm{CV}}_{\mathrm{IVD}-{\mathrm{MD}}_{Y}}$ and ${\mathrm{CV}}_{\mathrm{IVD}-{\mathrm{MD}}_{X}}$. Notably, the magnitudes of ${\mathrm{CV}}_{\mathrm{IVD}-{\mathrm{MD}}_{X}}$ and ${\mathrm{CV}}_{\mathrm{IVD}-{\mathrm{MD}}_{Y}}$, as well as the standard error of ${\hat{\beta }}_{1}$, also contribute to this relationship, albeit to a much lesser degree of importance. These complexities, however, are beyond the scope of this discussion. As a rule of thumb, Equation (5) is advisable if ${\hat{\beta }}_{1}\in \left[0.75,1.25\right]$. This criterion aims to limit the average percentage difference in $\hat{\zeta }$ to less than 20%. A wider range of ${\hat{\beta }}_{1}$ values is accepted, ${\hat{\beta }}_{1}\in \left[0.5,2\right]$, if the ratio between ${\mathrm{CV}}_{\mathrm{IVD}-{\mathrm{MD}}_{Y}}$ and ${\mathrm{CV}}_{\mathrm{IVD}-{\mathrm{MD}}_{X}}$ satisfies one of these two conditions: it is less than or equal to 1/4 or larger than or equal to 4. Upon reviewing the clinical data detailed in Section 3.2, we observe that the values of ${\hat{\beta }}_{1}$ range from 0.86 to 1.14. Consequently, Equation (5) is deemed appropriate for these data sets. Nonetheless, in this paper, the estimation of ζ will be solely based on Equation (4) since it is considered to be more robust than its simplified alternative.

### 2.2 Statistical Properties of the DINS Estimator

As established in Equation (4) or (5), the variable $\hat{\zeta }$ is a random variable, being defined by estimators, which are random by definition. In order to identify which values of $\hat{\zeta }$ correspond to excessive DINS, a thorough examination of the statistical distribution of $\hat{\zeta }$ must be conducted, contingent upon an assortment of statistical assumptions. It is important to note that the distribution of $\hat{\zeta }$ is anticipated to vary according to different study designs, specifically in terms of n and R.

By supposing a set of distributional assumptions regarding CS measurements, such as normality and homoskedasticity, the DINS detection principle can be formulated as a parametric hypothesis test. This is accomplished by deducing that $\hat{\zeta }$ follows a particular asymptotic distribution, subject to specific conditions. Under a certain collection of statistical assumptions, it can be inferred that $\hat{\zeta }$ asymptotically follows an F‐distribution. However, due to the practical limitations imposed on study designs, this approach may not be the most efficacious.

Provided that the CS measurements serve as an appropriate representation of the theoretical population of CS measurements, Monte Carlo simulation based on nonparametric resampling can be employed for inference on $\hat{\zeta }$. This method, commonly denoted as the Bootstrap (Efron and Tibshirani 1993), facilitates the bypassing of distributional suppositions, while concurrently permitting statistical inference on $\hat{\zeta }$, granted that the sample is a satisfactory representation of the theoretical population of interest. The bootstrap method permits the estimation of a multitude of summary statistic estimates for $\hat{\zeta }$, including bootstrap percentile confidence intervals (BPCI), standard deviation (SD), coefficient of variation (CV), and median absolute deviation (MAD). Considering that CS measurements are customarily conducted in replicates, leading to the subsequent configuration of these replicates into a cluster, our circumstances require the implementation of a cluster‐bootstrap algorithm. In this context, we employ a cluster‐bootstrap algorithm that resamples CSs with replacement, where each resampled CS retains its original measurements. More details about this algorithm and its associated bootstrap estimators are provided in Appendix A. Given the adaptability of the cluster‐bootstrap algorithm, it is used for statistical inference on $\hat{\zeta }$ within our clinical data (see Section 3.2).

### 2.3 Simulation Scenarios for the Investigation of $\hat{\zeta }$

In this section, we describe the setup for the simulation study aiming to analyze the distribution of $\hat{\zeta }$ in the presence of varying degrees of DINS between pairs of IVD‐MDs. The distribution of $\hat{\zeta }$ will be estimated using parametric Monte Carlo simulations based on different combinations of theoretical population parameters, with the aim of conducting a thorough exploration of $\hat{\zeta }$ and determining the values that signify excessive DINS. We refer to five main simulation scenarios, each generating $\hat{\zeta }$ values from assorted combinations of simulation parameters such as IVD‐MD CVs, number of CSs (n), number of replicates (R), and other parameters for the respective simulation scenario. The details of these five simulation scenarios are elaborated further in the *simulation settings* section of the Supporting Information.

In each simulation scenario, we generate 1,000,000 $\hat{\zeta }$ values for each combination of main simulation parameters, ensuring that the standard deviations of the approximated summary statistics of $\hat{\zeta }$ are smaller than 0.01. The main simulation parameters include n, R, the IVD‐MD CVs, and the concentration interval. The results of the first simulation setting, documented in the Supporting Information, demonstrate negligible influence of IVD‐MD CVs (between 0.1% and 10%) and concentration intervals ([5,10], [2,10], [500,750], [70,1600] units) on $\hat{\zeta }$. Consequently, our focus narrows to the 15 unique combinations of n=20,25,30,35,40 and R=2,3,4, allowing IVD‐MD CVs and concentration intervals to be generated from continuous random variables for each simulated $\hat{\zeta }$. Further details on these random variables are provided in the Supporting Information.

Alongside the main simulation parameters, we accommodate auxiliary parameters, including factors related to heteroskedasticity and parameters delineating the DINS structure. Prior to engaging with the five simulation scenarios, it is necessary to define the term *base standard deviation*. The interpretation of base standard deviation is dependent on the specific simulation scenario under consideration. In the second simulation scenario (refer to the list below), we simulate measurements of IVD‐MDs with heteroskedasticity. In this context, we consider two base standard deviations:

(6) $$\begin{array}{cc} \text{base standard}\;{\text{deviation}}_{X} & =\sigma_{\mathrm{IVD}-{\mathrm{MD}}_{X}} \end{array}$$

(7) $$\begin{array}{cc} \text{base standard}\;{\text{deviation}}_{Y} & =\sigma_{\mathrm{IVD}-{\mathrm{MD}}_{Y}}. \end{array}$$

For the third and fourth simulation scenarios listed below, we define the base standard deviation as the square root of the combined variance derived from the repeatability of each individual IVD‐MD:

(8) $$\text{base standard deviation}=\sqrt{\sigma_{\mathrm{IVD}-{\mathrm{MD}}_{Y}}^{2}+\sigma_{\mathrm{IVD}-{\mathrm{MD}}_{X}}^{2}}.$$

**FIGURE 1:**
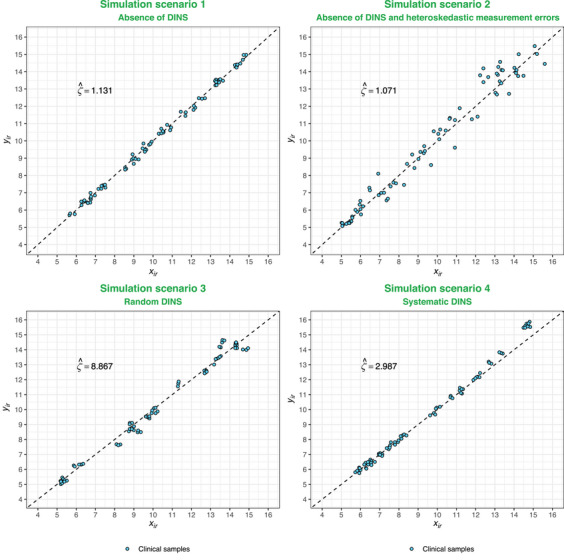
Visual representations of the simulation scenarios 1–4, along with their respective computed $\hat{\zeta }$ values for one realization. The blue‐filled circles signify individual pairs of IVD‐MD measurements $\left(x_{ir},y_{ir}\right)$. The black dashed lines in the background illustrate the lines of equivalence, defined by an intercept of zero and a slope of one.

The following list summarizes the five simulation scenarios:

1. **Simulation Scenario 1: No DINS, variance homogeneity:** Both IVD‐MDs have identical nonselectivity profiles.
2. **Simulation Scenario 2: No DINS, variance heterogeneity:** The two IVD‐MDs under consideration exhibit identical nonselectivity profiles. However, we introduce heteroskedasticity, a term synonymous with variance or standard deviation heterogeneity. In this context, heteroskedasticity implies that the measurement error standard deviations for these IVD‐MDs are functions of the concentration level. Consequently, within the concentration interval, the standard deviations at one point differ from those at another. This inherent standard deviation heterogeneity is accounted for via the auxiliary simulation parameter, η. This parameter, also denoted as the *heteroskedasticity factor*, is employed as a multiplier of the standard deviations at the lower limit of the concentration interval. The resultant products, η times the lower limit concentration interval standard deviations, serve as the upper limit concentration interval standard deviations. Thus, the lower limit concentration interval standard deviations are defined to be equal to the two base standard deviations. Indeed, the IVD‐MD standard deviations at the lower limit of the concentration interval are Equations (6) and (7). In contrast, the standard deviations at the upper limit of the concentration interval are given by $\eta \cdot [\text{base}\text{standard}\;{\text{deviation}}_{X}]$ and $\eta \cdot [\text{base standard}\;{\text{deviation}}_{Y}]$. The standard deviations gradually increase or decrease from the lower to the upper end of the concentration interval, with these two endpoints serving as the boundary. The degree of change in standard deviations is contingent on the number of CSs (n). The transition is linear between the two extremes, with n equally spaced steps calculated within the intervals $\left[\text{base standard}\;{\text{deviation}}_{X}\right]$ and $\left[\text{base standard}\;{\text{deviation}}_{Y}\right]$ to $\eta \cdot [\text{base standard}\;{\text{deviation}}_{X}]$ and $\eta \cdot [\text{base standard}\;{\text{deviation}}_{Y}]$, respectively. In particular, the jth standard deviations within the defined limits are $\left[\text{base standard}\;{\text{deviation}}_{X}\right]+\left(j\cdot \left[\text{base standard}\;{\text{deviation}}_{X}\right]\cdot \left(\eta -1\right)/n\right)$ and $\left[\text{base standard}\;{\text{deviation}}_{Y}\right]+\left(j\cdot \left[\text{base}\text{standard}\;{\text{deviation}}_{Y}\right]\cdot \left(\eta -1\right)/n\right)$, respectively. For example, if η=7.5, the standard deviations of measurement errors at the lower limit of the concentration interval are equal to the base standard deviations, and the standard deviations for measurement errors at the upper limit of the concentration interval are 7.5 times this. The standard deviations gradually increase from the base standard deviations to 7.5 times the base standard deviations from the lower to the upper concentration interval in accordance with the rule above. This specific example is visualized in the top‐right plot of Figure 1.
3. **Simulation Scenario 3: Random DINS, variance homogeneity:** The pair of IVD‐MDs is subject to the influence of *random DINS*. This phenomenon, termed as random DINS, arises when DINS impacts the measurement results of CSs randomly, adhering to specific probability distributions, such as the beta and binomial distributions. It is characterized by the simulation parameters p and $m_{\max}$. The parameter p represents the average proportion of the n CSs affected by random DINS, while $m_{\max}$ is the maximal relocation magnitude multiplier, referring to the maximum severity of random DINS. The product of $m_{\max}$ and the base standard deviation defines the upper bound of measurement results' relocation due to random DINS. Consequently, as $m_{\max}$ increases, CSs affected by random DINS are more likely to have their measurements deviate further from the equivalence line of the specific IVD‐MD comparison. $m_{\max}$ is called a multiplier because it is multiplied by the base standard deviation. Relocation of measurements due to random DINS occurs solely in the y‐direction to ensure that affected CSs do not move closer to the equivalence line. However, this relocation in the y‐direction can also be regarded as a relocation in the x‐direction or a combination of both directions, given that the corresponding unaffected measurement results are unknown.
4. **Simulation Scenario 4: Systematic DINS, variance homogeneity:** In this simulation, we will be comparing the results of two IVD‐MDs influenced by *systematic DINS*. Systematic DINS is a type of DINS where the results of the IVD‐MD comparison differ in a predictable and consistent manner. This can be observed by analyzing scatter plots of the comparison results, where it will be clear that the CSs results of the two IVD‐MDs form a distinct pattern. In this context, systematic DINS is defined by the simulation parameters q and $m_{\max}$. $m_{\max}$ is defined as in the previous simulation scenario, but q denotes the quantile interval, representing the subconcentration interval, where systematic DINS has its effect. q is an interval with lower limit l and upper limit u. Here, l,u∈[0,1] such that l≤u. Furthermore, q must satisfy that l=0oru=1. q must be a subset of [0,1] because of required domains of l and u. Those CSs having measurements coinciding with q will be affected but at different degrees. The effect of systematic DINS will be largest near the 0th or 100th percentile of the concentration interval and smallest where the interval of q begins or ends. For q with upper limit equal to u=1, the relocation multiplier will be smallest at l≤1, and for q with lower limit l=0, the relocation multiplier will be smallest at u≥l. For example, if q=[0.80,1] (l=0.80 and u=1) and a CS's measurements coincides with the 100th percentile of the concentration interval, we expect that the CS's measurements are relocated with the multiplier $m_{\max}$ times the base standard deviation. However, in the same example, if a CS's measurements coincides with the 90th percentile, we expect that the CS's measurements are relocated with the multiplier $0.5\cdot m_{\max}$ times the base standard deviation. Similarly, a CS's measurements coincides with the 85th percentile, results in its measurements being relocated with relocation multiplier $0.25\cdot m_{\max}$ times the base standard deviation. Or, if a CS's measurements coincide with the 95th percentile, the measurements are relocated with the relocation multiplier $0.75\cdot m_{\max}$ times the base standard deviation.
5. **Simulation Scenario 5: General DINS:** Pairs of IVD‐MDs exhibit DINS expressed with an average increase in the widths of the pointwise OLS prediction intervals (M). This specific manifestation of DINS is acknowledged as a more generalized setting wherein DINS may assume a broad range of forms. Our primary focus lies solely on the average increase in the widths of the pointwise OLS prediction intervals (M), without any regard to the specific type of DINS responsible for the expansion. M serves as the singular simulation parameter defining this DINS setting. The parameter M can be expressed in two forms—either as a decimal number or a percentage. To establish a clear distinction between the two, M(%) is employed to represent the percentage, while M refers to the decimal representation.

The full suite of simulation results can be found in the Supporting Information figures and tables. For those interested in replicating these results, an R seed has been provided to ensure reproducibility. The most relevant findings are presented in Section 3.1. To evaluate the robustness of the estimator $\hat{\zeta }$ under various conditions, including missing data and alternative distributional assumptions for $\tau_{i}$, $h_{ir}$, and $v_{ir}$, a sensitivity analysis was conducted. The detailed methodology and results of this analysis are documented in the Supporting Information, but only key findings and high‐level conclusions drawn from the sensitivity analysis are presented in here and are presented in Section 5.3.

Figure 1 portrays the first four simulation scenarios, each characterized by specific main and auxiliary parameters. The depictions of the first four simulation scenarios share the same main simulation parameters: n=25, R=3, IVD‐MD CVs equal to 1%, with the concentration interval defined between 5 and 15 units. The distinction arises with the auxiliary parameters: for the variance heterogeneity simulation scenario (scenario 2), η=7.5. Scenario 3, featuring random DINS, sets parameters p and $m_{\max}$ at 1/3 and 7.5, respectively. Simulation of systematic DINS (scenario 4) employ q=[2/3,1] and $m_{\max}=7.5$.

In illustrating standard deviation heterogeneity, the standard deviations of the IVD‐MDs at the maximum point of the concentration interval are 7.5 times greater than those at the minimum point. In the representation of random DINS, roughly one‐third of all CSs are affected, with measurements shifted by up to 7.5 times the base standard deviation. For systematic DINS, CSs with measurements equal to or exceeding the two‐thirds quantile of the concentration interval undergo a similar shift. In addition, each of these four scenarios includes calculated $\hat{\zeta }$ values derived from the corresponding simulated data sets in simulation scenarios 1–4.

The fifth simulation scenario, due to the limitless possible forms of DINS, is not depicted. However, one can imagine this scenario as an extension of the first or second scenario, where all pairs of IVD‐MD measurements are further displaced from the lines of equivalence.

### 2.4 Rejection Regions for Acceptable DINS

We propose to utilize the average relative increase of pointwise OLS regression prediction intervals, denoted as M, to establish a rule for identifying IVD‐MD comparisons with measurements influenced by excessive DINS. For a specific DINS study, we specify a suitable value for M. We define ${\hat{\zeta }}_{0}$ to be an estimate of ζ derived from a scenario with a pair of IVD‐MDs devoid of DINS, and $\hat{\zeta }$ to be the estimate of ζ when the same pair of IVD‐MDs features DINS. As derived in *Simulation setting 5: General differences in non‐selectivity* of the Supporting Information, the relationship between $\hat{\zeta }$, ${\hat{\zeta }}_{0}$, and M is outlined as follows:

(9) $$\hat{\zeta }={\left(1+M\right)}^{2}\cdot {\hat{\zeta }}_{0}.$$

Thus, M can be expressed as

$$M=\sqrt{\frac{\hat{\zeta }}{{\hat{\zeta }}_{0}}}-1.$$

The selection of M signifies the acceptable level of DINS. As such, we presume the existence of DINS, albeit its permissible magnitude is limited by the chosen M. Regardless of the M chosen, we examine the size of our study design. Larger study designs, that is, those with a high number of CSs and replicates, will yield broader rejection regions of $\hat{\zeta }$ in comparison to smaller study designs. The reason being, larger study designs result in lower variance of $\hat{\zeta }$, which in turn causes an expansion in the rejection regions of $\hat{\zeta }$. Therefore, it is crucial to consider the size of the study design when determining the range of $\hat{\zeta }$ values that would signify excessive DINS. Upon choosing an appropriate M and examining our study design's size, we conduct Monte Carlo simulations to approximate an upper limit of $\hat{\zeta }$—given the choice of M and the size of the study design, referred to as ${\hat{\zeta }}_{\mathrm{upper}}$. To ensure that merely 1% of all $\hat{\zeta }$ values exceed ${\hat{\zeta }}_{\mathrm{upper}}$ for the selected M, and given that ${\hat{\zeta }}_{0,0.99}$ is the 99th percentile of ${\hat{\zeta }}_{0}$, we proceed to define

$${\hat{\zeta }}_{\mathrm{upper}}={\left[\hat{\zeta }|M\right]}_{0.99}={\left(1+M\right)}^{2}\cdot {\hat{\zeta }}_{0,0.99},$$

which implies that the upper limit of $\hat{\zeta }$, corresponds to the 99th percentile of the conditional random variable $\hat{\zeta }$ given a specific choice of M. Theoretically, this signifies that fewer than 1% of all $\hat{\zeta }$ values will exceed ${\hat{\zeta }}_{\mathrm{upper}}$ for any average relative increase in pointwise prediction intervals less than M. Conversely, if the actual average relative increase in the pointwise prediction intervals surpasses M, more than 1% of $\hat{\zeta }$ values will be situated above ${\hat{\zeta }}_{\mathrm{upper}}$.

Upon simulating the value of ${\hat{\zeta }}_{\mathrm{upper}}$, we calculate $\hat{\zeta }$ using Equation (4) or (5), employing either raw or transformed CS data. The choice between raw or transformed data is dictated by the one producing a $\hat{\zeta }$ value most proximate to 1. Should the observed $\hat{\zeta }$ value for a pair of IVD‐MDs exceed the approximated ${\hat{\zeta }}_{\mathrm{upper}}$, we deduce that DINS is present with an excessive magnitude for that IVD‐MD pair.

**FIGURE 2:**
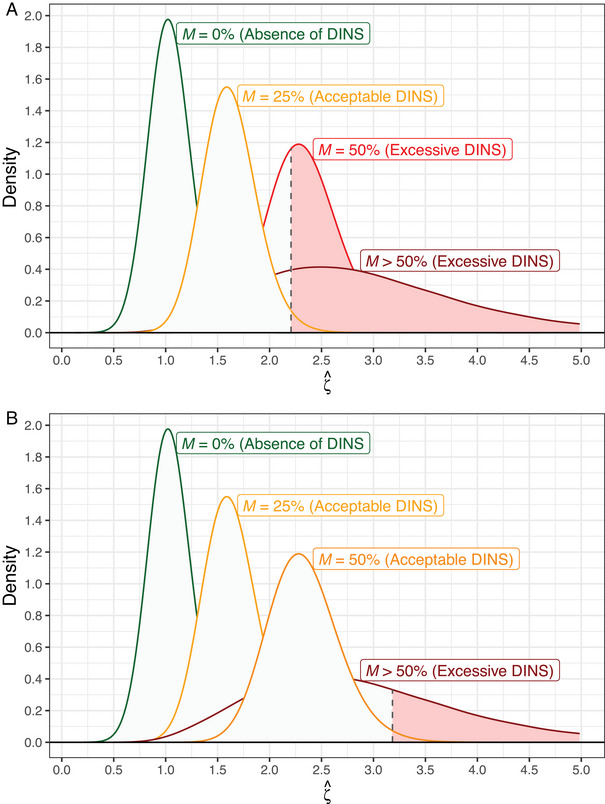
The statistical methodology for detecting excessive differences in nonselectivity (DINS) relies on the observed value of $\hat{\zeta }$ and a predetermined acceptable average percentage increase, M(%), in pointwise prediction interval widths attributable to DINS. Upon defining a suitable value for M(%), the subsequent step involves exploring the distribution of the conditional random variable $\hat{\zeta }$ given M(%), which inherently depends on the number of CSs and replicates. In these two examples, we have 25 clinical samples and three replicates. Excessive DINS is inferred if the observed $\hat{\zeta }$ surpasses the 99th percentile of this conditional random variable. Panel A illustrates a scenario with M(%) set at 25%, where the rejection region for $\hat{\zeta }$ (shown in red) extends from the 99th percentile of $\hat{\zeta }$ given M(%)=25%. Conversely, Panel B portrays an alternative scenario with M(%) chosen as 50%, and the corresponding rejection region for $\hat{\zeta }$ (also in red) starts from the 99th percentile of $\hat{\zeta }$ given M(%)=50%. The red shaded areas also illustrate the probability of falsely concluding with excessive DINS for the three other distributions.

The process for flagging IVD‐MD pairs having excessive DINS, employing M as a representation of the maximal tolerable magnitude of DINS, is graphically illustrated in Figure 2 for two examples of excessive DINS. This visual representation is predicated upon Monte Carlo simulations of $\left[\hat{\zeta }|M\left(\%\right)=0\%\right]$, $\left[\hat{\zeta }|M\left(\%\right)=25\%\right]$, $\left[\hat{\zeta }|M\left(\%\right)=50\%\right]$, and $\left[\hat{\zeta }|M\left(\%\right)>50\%\right]$. The simulations were executed with main simulation parameters consisting of n=25 CSs, R=3 replicates, IVD‐MD CVs of 2% and 1%, and a concentration interval defined between 5 and 90 units. Other parameter combinations of n, R, and M(%) are delineated in Tables 1 and B1.

**TABLE 1:** Monte Carlo simulations of the 99th percentile of $\hat{\zeta }$ across the five simulation scenarios and a pertinent subset of study designs. The parameter ${\hat{\zeta }}_{0.99}$ represents the 99th percentile of $\hat{\zeta }$. Other parameters in the table are defined as in Section 2.3.

n	R	Simulation scenario	η	$m_{\max}$	p	u−l	M(%)	${\hat{\zeta }}_{0.99}$
20	2	1	1.0	0	0.00	0.00	0%	1.908
20	2	2	0.1	0	0.00	0.00	0%	2.204
20	2	2	10.0	0	0.00	0.00	0%	2.336
20	2	3	1.0	5	0.05	0.00	>0%	3.818
20	2	3	1.0	10	0.05	0.00	>0%	10.312
20	2	3	1.0	5	0.30	0.00	>0%	8.496
20	2	3	1.0	10	0.30	0.00	>0%	27.990
20	2	4	1.0	5	0.00	0.05	>0%	3.542
20	2	4	1.0	10	0.00	0.05	>0%	9.361
20	2	4	1.0	5	0.00	0.30	>0%	4.796
20	2	4	1.0	10	0.00	0.30	>0%	15.406
20	2	5	1.0	0	0.00	0.00	25%	2.977
20	2	5	1.0	0	0.00	0.00	50%	4.282
20	2	5	1.0	0	0.00	0.00	75%	5.833
20	2	5	1.0	0	0.00	0.00	100%	7.602
20	3	1	1.0	0	0.00	0.00	0%	1.509
20	3	2	0.1	0	0.00	0.00	0%	1.645
20	3	2	10.0	0	0.00	0.00	0%	1.744
20	3	3	1.0	5	0.05	0.00	>0%	3.288
20	3	3	1.0	10	0.05	0.00	>0%	9.138
20	3	3	1.0	5	0.30	0.00	>0%	7.157
20	3	3	1.0	10	0.30	0.00	>0%	23.971
20	3	4	1.0	5	0.00	0.05	>0%	3.025
20	3	4	1.0	10	0.00	0.05	>0%	8.171
20	3	4	1.0	5	0.00	0.30	>0%	4.133
20	3	4	1.0	10	0.00	0.30	>0%	13.700
20	3	5	1.0	0	0.00	0.00	25%	2.354
20	3	5	1.0	0	0.00	0.00	50%	3.389
20	3	5	1.0	0	0.00	0.00	75%	4.612
20	3	5	1.0	0	0.00	0.00	100%	6.028
25	3	1	1.0	0	0.00	0.00	0%	1.442
25	3	2	0.1	0	0.00	0.00	0%	1.556
25	3	2	10.0	0	0.00	0.00	0%	1.650
25	3	3	1.0	5	0.05	0.00	>0%	3.035
25	3	3	1.0	10	0.05	0.00	>0%	8.224
25	3	3	1.0	5	0.30	0.00	>0%	6.650
25	3	3	1.0	10	0.30	0.00	>0%	22.193
25	3	4	1.0	5	0.00	0.05	>0%	2.797
25	3	4	1.0	10	0.00	0.05	>0%	7.451
25	3	4	1.0	5	0.00	0.30	>0%	3.976
25	3	4	1.0	10	0.00	0.30	>0%	13.108
25	3	5	1.0	0	0.00	0.00	25%	2.251
25	3	5	1.0	0	0.00	0.00	50%	3.240
25	3	5	1.0	0	0.00	0.00	75%	4.409
25	3	5	1.0	0	0.00	0.00	100%	5.762
30	3	1	1.0	0	0.00	0.00	0%	1.393
30	3	2	0.1	0	0.00	0.00	0%	1.496
30	3	2	10.0	0	0.00	0.00	0%	1.584
30	3	3	1.0	5	0.05	0.00	>0%	2.842
30	3	3	1.0	10	0.05	0.00	>0%	7.589
30	3	3	1.0	5	0.30	0.00	>0%	6.309
30	3	3	1.0	10	0.30	0.00	>0%	20.986
30	3	4	1.0	5	0.00	0.05	>0%	2.652
30	3	4	1.0	10	0.00	0.05	>0%	6.963
30	3	4	1.0	5	0.00	0.30	>0%	3.881
30	3	4	1.0	10	0.00	0.30	>0%	12.662
30	3	5	1.0	0	0.00	0.00	25%	2.180
30	3	5	1.0	0	0.00	0.00	50%	3.139
30	3	5	1.0	0	0.00	0.00	75%	4.269
30	3	5	1.0	0	0.00	0.00	100%	5.570
40	4	1	1.0	0	0.00	0.00	0%	1.260
40	4	2	0.1	0	0.00	0.00	0%	1.313
40	4	2	10.0	0	0.00	0.00	0%	1.398
40	4	3	1.0	5	0.05	0.00	>0%	2.512
40	4	3	1.0	10	0.05	0.00	>0%	6.563
40	4	3	1.0	5	0.30	0.00	>0%	5.626
40	4	3	1.0	10	0.30	0.00	>0%	18.722
40	4	4	1.0	5	0.00	0.05	>0%	2.362
40	4	4	1.0	10	0.00	0.05	>0%	6.101
40	4	4	1.0	5	0.00	0.30	>0%	3.675
40	4	4	1.0	10	0.00	0.30	>0%	11.856
40	4	5	1.0	0	0.00	0.00	25%	1.969
40	4	5	1.0	0	0.00	0.00	50%	2.836
40	4	5	1.0	0	0.00	0.00	75%	3.858
40	4	5	1.0	0	0.00	0.00	100%	5.039

The significance of the gray values in Table 1 is values for which there is no relevant effect from the simulation parameter.

Figure 2A represents a scenario where M(%)=25% is selected, suggesting that IVD‐MD comparisons yielding $\hat{\zeta }$ values exceeding ${\left[\hat{\zeta }|M\left(\%\right)=25\%\right]}_{0.99}$ signify excessive DINS. Conversely, those IVD‐MD comparisons generating $\hat{\zeta }$ values not surpassing this threshold are considered to possess acceptable DINS. Figure 2B demonstrates the same principle as Figure 2A. However, M(%)=50% is selected rather than M(%)=25%, which means that M(%)=50% now represent acceptable DINS levels, while it was considered to represent excessive DINS levels in 2A.

There exist two pragmatic approaches to derive the value of $\zeta_{\mathrm{upper}}$. The first method entails the use of a look‐up table, which is based on presimulated $\hat{\zeta }$ values for assorted combinations of n, R, and M(%). The alternative method involves conducting the simulation ourselves. While the latter may pose a challenge for individuals lacking statistical proficiency, it offers greater flexibility compared to a look‐up table. A practical look‐up table should not be too large, thereby implying that it cannot account for every possible combination of n, R, and M(%), but rather a pertinent subset. For a subset of {n:20≤n≤50}, and R=2,3,4,5, Table B1 may be employed to derive an appropriate upper value of $\hat{\zeta }$. For other values, one could resort to using the “simulate_zetas()” part of the “commutability” package, see Section 5, to simulate ${\left[\hat{\zeta }|M\left(\%\right)\right]}_{0.99}$ given n, R, and M(%).

## 3 Results

### 3.1 Simulation Results

In order to identify IVD‐MD pairs with excessive levels of DINS, we previously proposed the use of the 99% percentile of the conditional random variable $\hat{\zeta }$, contingent upon a tolerable average relative increase in pointwise prediction interval width due to DINS, M. This percentile serves as a threshold. Consequently, if an observed value of $\hat{\zeta }$ for a specific IVD‐MD pair surpasses this threshold, it signals excessive DINS within that pair for the specified value of M. This concept is further elucidated in Section 2.4. Henceforth, in our review of the simulation results, we will primarily focus on the outcomes pertaining to the 99% percentile of $\hat{\zeta }$, denoted ${\hat{\zeta }}_{0.99}$, associated with the simulation parameters related to each of the five simulation scenarios.

**FIGURE 3:**
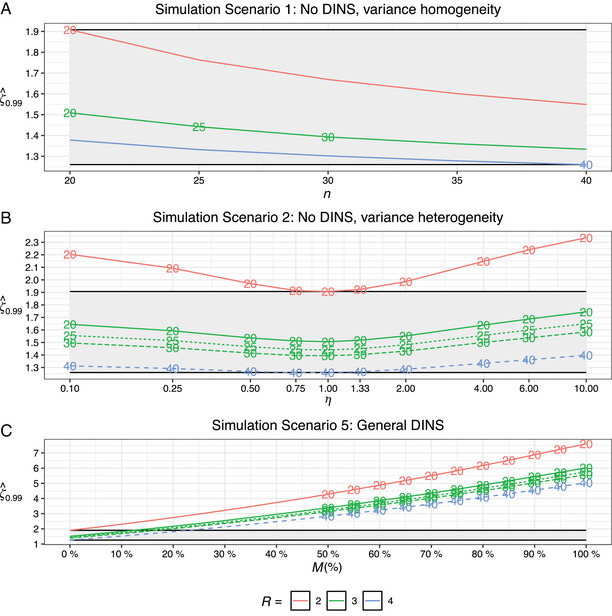
The 99% percentiles of $\hat{\zeta }$ (${\hat{\zeta }}_{0.99}$) against the simulation parameters for simulation scenarios 1, 2, and 5. Panel A illustrates ${\hat{\zeta }}_{0.99}$ across five study designs versus the number of clinical samples, n. Panel B shows that ${\hat{\zeta }}_{0.99}$ is depicted as a function of heteroskedasticity factors, η, ranging from 0.1 to 10, for the same quintet of study designs. Panel C shows ${\hat{\zeta }}_{0.99}$ in conjunction with the average percentage increase in pointwise prediction interval widths attributable to DINS, M(%), again for the identical five study designs. Gray ribbons in the plots visually represent the range between the smallest ${\hat{\zeta }}_{0.99}$, associated with the largest study design configured with 40 clinical samples and four replicates, and the largest ${\hat{\zeta }}_{0.99}$, associated with the smallest study design with 20 clinical samples and two replicates, under the conditions of zero DINS and homoskedastic measurement errors. The numbers within the plots indicate the number of clinical samples.

**FIGURE 4:**
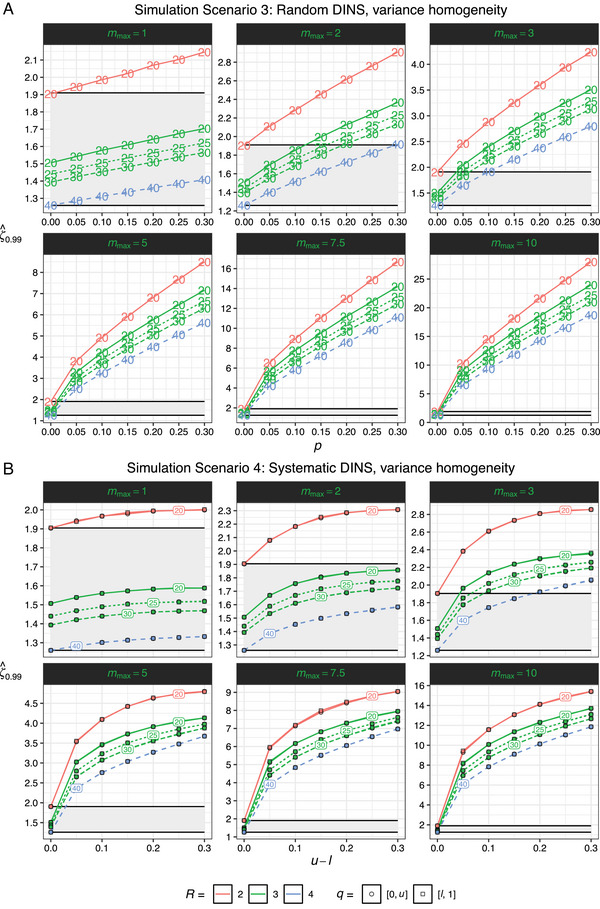
Demonstrations of the relationship between the 99% percentiles of $\hat{\zeta }$, ${\hat{\zeta }}_{0.99}$, and the simulation parameters of simulation scenarios 3 and 4 for a pertient subset of study designs. Panel A illustrates the relationship between ${\hat{\zeta }}_{0.99}$ and the average proportion of random DINS affected clinical samples, p, in conjunction with the maximum relocation multiplier, $m_{\max}$. Panel B portrays the relationship between the ${\hat{\zeta }}_{0.99}$ and the quantile interval length, u−l, where systematic DINS has its effect, in combination with $m_{\max}$. Gray ribbons in the plots visually represent the range between the smallest ${\hat{\zeta }}_{0.99}$, associated with the largest study design configured with 40 clinical samples and four replicates, and the largest ${\hat{\zeta }}_{0.99}$, associated with the smallest study design with 20 clinical samples and two replicates, under the conditions of zero DINS and homoskedastic measurement errors. The numbers within the plots indicate the number of clinical samples.

Figure 3 presents the ${\hat{\zeta }}_{0.99}$ simulation results for scenarios 1, 2, and 5, whereas Figure 4 displays those for scenarios 3 and 4. Table 1, while considering fewer simulation parameter combinations than the figures, offers exact numerical values of ${\hat{\zeta }}_{0.99}$ for a curated subset of simulation parameters across all five simulation scenarios.

It is important to note that while other metrics of $\hat{\zeta }$—such as mean, variance, skewness, kurtosis, and alternative percentiles—are documented in the Supporting Information, they are not discussed here in numerical detail due to their secondary relevance.

**Simulation Scenario 1: No DINS, variance homogeneity:** Comparing two IVD‐MDs with identical or near‐identical nonselectivity profiles, ${\hat{\zeta }}_{0.99}$ for the largest considered study design (n=40,R=4) is 1.260. For the smallest study design (n=20,R=2), it stands at 1.908. Under typical study designs—(n=20,R=3); (n=25,R=3); and (n=30,R=3), the ${\hat{\zeta }}_{0.99}$ values are 1.509, 1.442, and 1.393, respectively. Figure 3A illustrates that increasing R by one results in a more substantial reduction in ${\hat{\zeta }}_{0.99}$ than a five‐point increase in n. This argument can be made for the remaining four simulation scenarios as well.

**Simulation Scenario 2: No DINS, variance heterogeneity:** When comparing two IVD‐MDs with identical nonselectivity profiles given heteroskedastic measurement errors, $\hat{\zeta }$ is altered in terms of variance, skewness, and kurtosis, with the influence on the mean being typically minimal. Based on the U‐patterns observed in Figure 3B, we deduce that ${\hat{\zeta }}_{0.99}$ is smallest when η is close to 1 and increases with the magnitude of |η−1|.

In Table 1, a 10‐fold increase in measurement error standard deviations throughout the concentration interval (i.e., η=10), corresponds to ${\hat{\zeta }}_{0.99}$ values of 2.336, 1.744, 1.650, 1.584, and 1.398 for the respective study designs: (n=20,R=2); (n=20,R=3); (n=25,R=3); (n=30,R=3); and (n=40,R=4). Conversely, a 10‐fold decrease in standard deviations (i.e., η=1/10) corresponds to ${\hat{\zeta }}_{0.99}$ values of 2.204, 1.645, 1.556, 1.496, and 1.313. In summary, when comparing two IVD‐MDs with identical nonselectivity profiles, heteroskedasticity is associated with slightly elevated ${\hat{\zeta }}_{0.99}$ values, with a more pronounced increase under increasing heteroskedasticity than decreasing. However, the relative difference in the ${\hat{\zeta }}_{0.99}$ values between these two types of heteroskedasticity is marginal, approximately 6%.

Addressing heteroskedasticity to potentially reduce the variance of $\hat{\zeta }$ and thereby the ${\hat{\zeta }}_{0.99}$ value commonly involves a univariate transformation like log‐transformation, suitable for the types of heteroskedasticity examined here. Alternative transformations or weighting techniques could also be employed, given thorough inspection of IVD‐MD variances.

**Simulation Scenario 3: Random DINS, variance homogeneity:** As illustrated in Figure 4A, within the context of CSs subject to random DINS, the maximum relocation magnitude multiplier ($m_{\max}$) and the proportion of DINS‐impacted CSs (p) both positively correlate with ${\hat{\zeta }}_{0.99}$. With a constant $m_{\max}$ greater than 0, an evident leap of ${\hat{\zeta }}_{0.99}$ is observed as p increase from 0 to 0.05. For p≥0.05, the trajectory of ${\hat{\zeta }}_{0.99}$ is linear. In contrast, with p being constant and greater than 0, the interrelation between ${\hat{\zeta }}_{0.99}$ and $m_{\max}$ is distinctly parabolic. Notably, $m_{\max}$ exerts a more potent impact on the increase rate of ${\hat{\zeta }}_{0.99}$ juxtaposed to equivalent relative increments in p. Due to the parabolic nature of this correlation, the rate at which ${\hat{\zeta }}_{0.99}$ increase also elevates as $m_{\max}$ increases.

Table 1 affirms these observations with specific examples. For example, when p equals 1/20 and $m_{\max}$ is 5, the ${\hat{\zeta }}_{0.99}$ values for the study designs with (n=20,R=2); (n=20,R=3); (n=25,R=3); (n=30,R=3); and (n=40,R=4) are 3.818, 3.288, 3.035, 2.842, and 2.512, respectively. However, altering $m_{\max}$ to 10, while keeping the other parameters constant, results in ${\hat{\zeta }}_{0.99}$ values for these study designs at 10.312, 9.138, 8.224, 7.589, and 6.563, respectively. Conversely, keeping $m_{\max}$ unchanged, but setting p to 0.30, the ${\hat{\zeta }}_{0.99}$ values for the aforementioned study designs become 8.496, 7.157, 6.650, 6.309, and 5.626.

In the simulation study of CSs impacted by random DINS, it is evident that both the maximum relocation magnitude multiplier, which signifies the degree to which CS measurements affected by DINS deviate from the equivalence, and the average proportion of CSs affected by DINS play pivotal roles in influencing ${\hat{\zeta }}_{0.99}$. Specifically, an increase in either the proportion of CSs affected or the extent to which their measurement results are relocated leads to a corresponding rise in ${\hat{\zeta }}_{0.99}$. However, the maximum relocation magnitude multiplier has a more pronounced effect on the rate at which ${\hat{\zeta }}_{0.99}$ increases compared to equivalent relative changes in the proportion of DINS‐affected CSs.

**Simulation Scenario 4: Systematic DINS, variance homogeneity:** In the context of systematic DINS, Figure 4B illustrates a correlation of ${\hat{\zeta }}_{0.99}$ with both $m_{\max}$ and the range of the quantile interval, denoted u−l. Particularly, when $m_{\max}$ is held constant and is larger than 0, ${\hat{\zeta }}_{0.99}$ increases with u−l at first, then reaches a particular maximum value specific to the study design and the value of $m_{\max}$, and then decreases again as u−l approaches 1. When u−l is held constant and is larger than 0, ${\hat{\zeta }}_{0.99}$ increases with $m_{\max}$ in a parabolic manner.

Furthermore, a relative increase in $m_{\max}$ leads to a more pronounced increase in ${\hat{\zeta }}_{0.99}$ compared to an equivalent relative increase in u−l. As observed for random DINS with p, the impact of increasing u−l on ${\hat{\zeta }}_{0.99}$ becomes more pronounced as $m_{\max}$ increases.

Through Figure 4B, we observe that the ${\hat{\zeta }}_{0.99}$ values for systematic DINS are remarkably consistent across both lower and upper concentration ranges in every considered study design. The almost congruent positioning of circle and squares, which represent these two systematic DINS types, affirm this finding.

Specific examples of these observed trends can be drawn from Table 1. Notably, when u−l equals 1/20 and $m_{\max}$ is set at 5, the corresponding ${\hat{\zeta }}_{0.99}$ values for the study designs (n=20,R=2); (n=20,R=3); (n=25,R=3); (n=30,R=3); and (n=40,R=4) were calculated as 3.542, 3.025, 2.797, 2.652, and 2.362, respectively. For other combinations of u−l and $m_{\max}$, respective ${\hat{\zeta }}_{0.99}$ values are documented in Table 1 across these five study designs.

In conclusion, ${\hat{\zeta }}_{0.99}$ manifests a distinct relationship with both the severity of systematic relocation applied to DINS‐affected CSs and the relative range of the concentration interval where systematic DINS has its effect. Particularly, the former correlation is always positive, but this is not necessarily the case for the latter. In addition, the quantile interval range within which systematic DINS occurs is more consequential than whether DINS affect CS results in the lower or upper extremes of the concentration interval. We also find that random DINS influence ${\hat{\zeta }}_{0.99}$ on a larger scale compared to systematic DINS for the comparable simulation parameters p and u−l and for every value of $m_{\max}$.

**Simulation Scenario 5: General DINS:** In the setting defined by the average percentage increase in pointwise OLS prediction interval widths, denoted by M(%) and attributed to an unspecified form of DINS, Figure 3C elucidates a positive, parabolic correlation between M(%) and ${\hat{\zeta }}_{0.99}$. Thus, an increase in M(%) not only augments the value of ${\hat{\zeta }}_{0.99}$ but also intensifies its rate of increase.

Exemplifying this, data from Table 1 illustrate that with M(%) set at 50% and 75%, the corresponding ${\hat{\zeta }}_{0.99}$ values are 4.282 and 5.833; 3.389 and 4.612; 3.240 and 4.409; 3.139 and 4.269; and 2.836 and 3.858 for the associated study designs (n=20,R=2); (n=20,R=3); (n=25,R=3); (n=30,R=3); and (n=40,R=4). For M(%) values of 25% and 100%, the corresponding ${\hat{\zeta }}_{0.99}$ values can be found in the same table.

In summary, our findings from simulation scenario 5 endorse a positive parabolic relationship between the average percentage increase in pointwise OLS prediction interval widths—attributable to DINS and ${\hat{\zeta }}_{0.99}$. This correlation is congruent with expectations, considering the theoretical relationship among $\hat{\zeta }$, ${\hat{\zeta }}_{0}$, and M displayed in Equation (9).

To summarize, we found that the extent and severity of DINS, whether random or systematic, have a pronounced effect on ${\hat{\zeta }}_{0.99}$. Specifically p, u−l, $m_{\max}$, and M—exert a considerable impact on ${\hat{\zeta }}_{0.99}$. Particularly, ${\hat{\zeta }}_{0.99}$ generally correlated positively with p, $m_{\max}$, and M. However, ${\hat{\zeta }}_{0.99}$ relates to u−l according to an inverted U‐pattern relationship. Thus, ${\hat{\zeta }}_{0.99}$ correlates positively with u−l up till some point, and then correlates negatively after that point. The heteroskedasticity factor, η only exerts a slight influence on ${\hat{\zeta }}_{0.99}$, which in most cases is negligible.

In addition, with p (pertaining to random DINS) and u−l (pertaining to systematic DINS) fixed above zero, the relationship between ${\hat{\zeta }}_{0.99}$ and $m_{\max}$ manifests as a positive parabola. A parabolic relationship was also observed between ${\hat{\zeta }}_{0.99}$ and M(%). Thus, for each value of M(%) and a particular study design, we can roughly associate it with a pair of parameters: $m_{\max}$ and p for random DINS, or $m_{\max}$ and u−l for systematic DINS. As a result, the relationship between M(%) and ${\hat{\zeta }}_{0.99}$ serves as an approximate representation of the relationships involving ${\hat{\zeta }}_{0.99}$ with both p and $m_{\max}$, as well as u−l and $m_{\max}$.

Furthermore, we found that study designs affect the relationships between ${\hat{\zeta }}_{0.99}$ and DINS‐specific parameters, involving p, u−l, $m_{\max}$, and M(%), as well as the heteroskedasticity factor η. Particularly, larger study designs, such as (n=40,R=4), are associated with lower elevations of ${\hat{\zeta }}_{0.99}$ compared to smaller designs, such as (n=25,R=3) or (n=20,R=2), regardless of whether the elevated $\hat{\zeta }$ values are due to random DINS, systematic DINS, or heteroskedasticity. Including a greater number of CSs or, more efficacious, replicates in the study design not only lowers ${\hat{\zeta }}_{0.99}$, but also increases the robustness of the methodology for detecting excessive levels of DINS due to decreased variance of $\hat{\zeta }$.

### 3.2 Clinical Data Analysis

Our analysis of clinical data focuses on the measurements of three specific analytes: glucose, hemoglobin (HB), and C‐reactive protein (CRP). Detailed information about these analytes, including variable names, variable types, IVD‐MD imprecision estimates, and descriptive statistics of the IVD‐MD measurements, are presented in Table 2. This table also provides a comprehensive overview of the CS and replicate IDs for each respective analyte.

**TABLE 2:** Information on the clinical data sets to be evaluated regarding differences in nonselectivity. “BPCI” is short for bootstrap percentile confidence interval. Note that “lower” refers to the lower part of the 95% BPCI confidence intervals, whereas “upper” refers to the upper part.

Variable information			Summary statistics for measurements						IVD‐MD imprecision estimates with 95% BPCI					
Variable	Type	Max. decimal places	Mean	Q1	Median	Q3	Min.	Max.	SD	${\mathrm{SD}}_{\mathrm{lower}}$	${\mathrm{SD}}_{\mathrm{upper}}$	CV (%)	${\mathrm{CV}}_{\mathrm{lower}}\;\left(\%\right)$	${\mathrm{CV}}_{\mathrm{upper}}\;\left(\%\right)$
**Glucose, Serum (unit: mmol/L)**														
SampleID	identifier						1.00	25.00						
ReplicateID	identifier						1.00	3.00						
Advia	numeric	2	7.875	5.520	7.55	9.850	4.22	12.86	0.048	0.036	0.062	0.615	0.453	0.805
Alinity	numeric	2	7.940	5.550	7.57	9.940	4.19	13.28	0.040	0.032	0.048	0.507	0.435	0.572
Cholestech	numeric	2	7.647	5.360	7.52	9.390	4.04	12.60	0.184	0.138	0.228	2.401	1.902	2.814
Cobas	numeric	2	7.955	5.550	7.63	9.980	4.22	13.14	0.072	0.050	0.092	0.905	0.687	1.086
Vitros	numeric	2	8.024	5.660	7.68	9.990	4.28	13.13	0.035	0.027	0.042	0.430	0.340	0.521
**Hemoglobin (unit: g/dL)**														
SampleID	identifier						2.00	25.00						
ReplicateID	identifier						1.00	3.00						
Dia Spect Tm	numeric	1	12.035	10.175	13.00	13.325	8.80	15.80	0.059	0.047	0.070	0.490	0.401	0.569
HemoCue Hb201	numeric	1	12.149	10.225	12.90	13.650	8.60	16.00	0.062	0.051	0.073	0.513	0.417	0.607
HemoCue Hb801	numeric	1	12.335	10.650	13.15	13.700	8.90	16.40	0.070	0.051	0.087	0.565	0.411	0.717
QR go	numeric	1	12.418	10.275	13.35	14.075	8.40	16.70	0.242	0.199	0.281	1.945	1.573	2.314
**C‐reactive protein (unit: mg/L)**														
SampleID	identifier						1.00	25.00						
ReplicateID	identifier						1.00	3.00						
Advia	numeric	1	38.081	14.900	38.60	53.600	4.50	84.40	0.326	0.217	0.428	0.856	0.647	1.047
Architect	numeric	1	36.468	12.800	36.50	52.500	4.60	82.40	0.380	0.297	0.456	1.041	0.779	1.379
Cobas	numeric	1	32.777	11.100	32.40	48.100	4.40	77.10	0.669	0.490	0.824	2.042	1.685	2.409
Dimension	numeric	2	37.096	12.500	35.10	53.400	5.20	86.40	0.698	0.550	0.859	1.880	1.364	2.602
Vitros	numeric	1	34.655	13.600	33.90	47.500	4.20	79.60	0.658	0.495	0.809	1.899	1.547	2.263

Utilizing the clinical data sets, we estimate the descriptive statistics for $\hat{\zeta }$. The descriptive statistics of $\hat{\zeta }$ are estimated by employing the cluster‐bootstrap algorithm, documented in Appendix A. We use 5000 bootstrap replicates for each IVD‐MD comparison to compute BPCIs and estimate summary statistics for $\hat{\zeta }$. The estimated descriptive statistics for $\hat{\zeta }$ are provided in Table 3. Glucose exhibit the smallest $\hat{\zeta }$ values on average, followed by HB and CRP, respectively.

**TABLE 3:** Descriptive statistics and bootstrap confidence intervals of $\hat{\zeta }$ for glucose, hemoglobin (HB), and C‐reactive protein (CRP) analytes. The descriptive statistics and confidence intervals of $\hat{\zeta }$ are estimated using the cluster‐bootstrap resampling algorithm for each comparison involving the analytes glucose, HB, and CRP. The abbreviation BPCI stands for bootstrap percentile confidence interval.

IVD‐MD comparison	Point estimate	95% BPCI		Bootstrap summary statistics of $\hat{\zeta }$									
Comparison	$\hat{\zeta }$	${\hat{\zeta }}_{\mathrm{lower}}$	${\hat{\zeta }}_{\mathrm{upper}}$	Mean	SD	Skewness	Kurtosis	MAD	Q1	Median	Q3	Min.	Max.
**Glucose, Serum (unit: mmol/L)**													
Advia ‐ Alinity	1.205	0.761	1.511	1.124	0.193	0.138	2.939	0.195	0.990	1.122	1.253	0.572	1.888
Advia ‐ Cholestech	1.133	0.827	1.648	1.136	0.216	1.235	5.741	0.186	0.987	1.099	1.244	0.691	2.572
Advia ‐ Cobas	0.770	0.564	1.045	0.770	0.120	0.656	3.999	0.111	0.688	0.757	0.838	0.425	1.399
Advia ‐ Vitros	1.902	1.159	2.739	1.836	0.404	0.489	3.139	0.407	1.537	1.804	2.088	0.823	3.640
Alinity ‐ Cholestech	1.148	0.854	1.578	1.139	0.184	0.955	4.615	0.166	1.010	1.113	1.239	0.711	2.134
Alinity ‐ Cobas	0.872	0.454	1.486	0.854	0.266	1.220	5.919	0.229	0.673	0.807	0.990	0.264	2.580
Alinity ‐ Vitros	2.836	1.798	3.597	2.653	0.460	0.168	3.046	0.456	2.339	2.633	2.951	1.095	4.263
Cholestech ‐ Cobas	1.193	0.871	1.665	1.193	0.203	0.788	4.131	0.189	1.050	1.168	1.310	0.690	2.554
Cholestech ‐ Vitros	1.388	0.991	1.945	1.364	0.245	0.951	4.507	0.225	1.191	1.329	1.497	0.807	2.880
Cobas ‐ Vitros	1.239	0.965	1.498	1.208	0.129	0.379	4.432	0.112	1.127	1.200	1.280	0.667	1.883
**Hemoglobin (unit: g/dL)**													
Dia Spect Tm ‐ HemoCue Hb201	5.687	2.692	8.661	5.327	1.528	0.459	3.286	1.527	4.227	5.207	6.289	1.448	11.930
Dia Spect Tm ‐ HemoCue Hb801	3.655	2.107	5.429	3.483	0.844	0.649	3.583	0.819	2.875	3.394	3.986	1.483	8.173
Dia Spect Tm ‐ QR go	1.113	0.848	1.426	1.091	0.146	0.645	3.675	0.139	0.988	1.076	1.177	0.719	1.770
HemoCue Hb201 ‐ HemoCue Hb801	6.110	2.775	9.061	5.630	1.573	0.468	3.722	1.493	4.563	5.526	6.584	1.172	14.647
HemoCue Hb201 ‐ QR go	1.553	1.160	1.906	1.499	0.190	0.324	3.177	0.187	1.366	1.489	1.620	0.895	2.298
HemoCue Hb801 ‐ QR go	1.833	1.213	2.588	1.777	0.358	0.818	4.404	0.343	1.522	1.733	1.990	0.937	4.077
**C‐reactive protein (unit: mg/L)**													
Advia ‐ Architect	3.609	1.693	6.110	3.476	1.125	0.829	4.116	1.034	2.686	3.334	4.117	0.723	8.776
Advia ‐ Cobas	4.044	2.174	6.103	3.819	1.010	1.099	6.693	0.901	3.138	3.707	4.364	1.172	11.472
Advia ‐ Dimension	8.141	4.095	13.649	7.926	2.437	0.815	4.007	2.304	6.170	7.600	9.324	2.004	21.587
Advia ‐ Vitros	2.547	1.758	3.167	2.420	0.351	0.277	3.910	0.322	2.191	2.403	2.627	0.985	3.998
Architect ‐ Cobas	2.500	1.027	3.246	2.273	0.512	−0.222	3.567	0.480	1.968	2.281	2.609	0.631	4.490
Architect ‐ Dimension	3.935	2.262	5.793	3.761	0.916	0.627	3.604	0.890	3.099	3.663	4.307	1.210	8.279
Architect ‐ Vitros	2.459	1.684	3.139	2.348	0.370	0.365	3.226	0.365	2.087	2.332	2.579	1.200	4.013
Cobas ‐ Dimension	3.742	1.815	5.052	3.485	0.809	−0.081	2.825	0.790	2.954	3.515	4.022	1.192	6.241
Cobas ‐ Vitros	3.374	1.838	5.086	3.194	0.863	0.646	3.421	0.889	2.529	3.104	3.738	1.222	7.227
Dimension ‐ Vitros	3.225	1.695	5.368	3.104	0.956	0.906	4.138	0.908	2.392	2.966	3.635	1.064	8.328

The estimated moments for $\hat{\zeta }$ across glucose, HB, and CRP data sets exhibit the following ranges. In the glucose data set, the means range from 0.77 to 2.653, variances range from 0.014 to 0.212, skewness values fall between 0.138 and 1.235, and kurtosis values fall between 2.939 and 5.919. For the HB data set, the means span from 1.091 to 5.63, variances span from 0.021 to 2.476, skewness values fall between 0.324 and 0.818, and kurtosis values fall between 3.177 and 4.404. Lastly, in the CRP data set, the means lie between 2.273 and 7.926, variances lie between 0.123 and 5.937, skewness values fall between −0.222 and 1.099, and kurtosis values fall between 2.825 and 6.693.

The relationship between high $\hat{\zeta }$ values and DINS may not be readily discernible when examining the IVD‐MD comparisons’ scatter plots. As an illustration, regard the comparison involving Abbott Alinity and Ortho Vitros within the glucose data set. Despite observing $\hat{\zeta }=2.836$, the scatter plot corresponding to this specific IVD‐MD comparison fails to exhibit a clear manifestation of DINS.

However, the presence of DINS becomes more discernible when we simulate a data set based on the estimated statistical characteristics of the compared IVD‐MDs without DINS and compare it with the original data set. Using the results in Table 2, we simulate CS glucose concentrations from a uniform probability distribution defined from 4.19 to 13.28 mmol/L. Subsequently, we add normally distributed measurement errors to the CS glucose concentrations, with zero mean values and standard deviations of 0.040 and 0.035 mmol/L for Abbott Alinity and Ortho Vitros, respectively.

**FIGURE 5:**
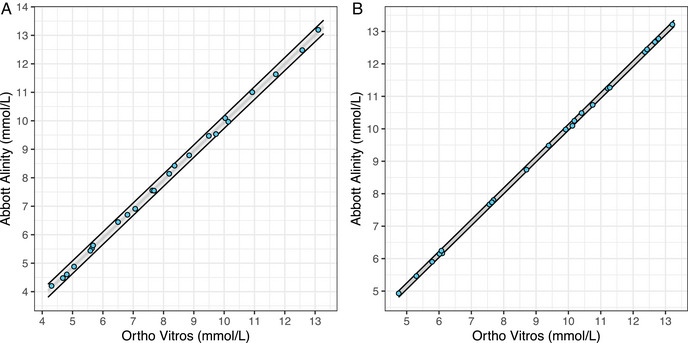
Scatter plots of glucose measurements results of the Abbott Alinity versus Ortho Vitros comparison. (A) Scatter plot illustrating the original pairs of glucose measurements of Abbott Alinity and Ortho Vitros, featuring the 99% pointwise prediction intervals (shaded gray area) derived from Fuller and Gillards’ (F–G) Deming regression model. For the original data, we have that $\hat{\zeta }=2.836$. (B) Scatter plot showcasing simulated pairs of glucose measurement results for the Abbott Alinity and Ortho Vitros comparison (with DINS removed), accompanied by the 99% pointwise prediction intervals (shaded gray area) based on the F–G Deming regression model. For the simulated data, we have that $\hat{\zeta }=1.013$.

The scatter plots with corresponding prediction intervals for both the original and simulated measurements of the Abbott Alinity versus Ortho Vitros IVD‐MD comparison are illustrated in Figure 5. A side‐by‐side comparison of these scatter plots disclose why $\hat{\zeta }$ is relatively large for this specific IVD‐MD comparison, demonstrated by the fact that the pointwise prediction intervals for the original measurements are, on average, wider than those for the simulated data.

**FIGURE B1:**
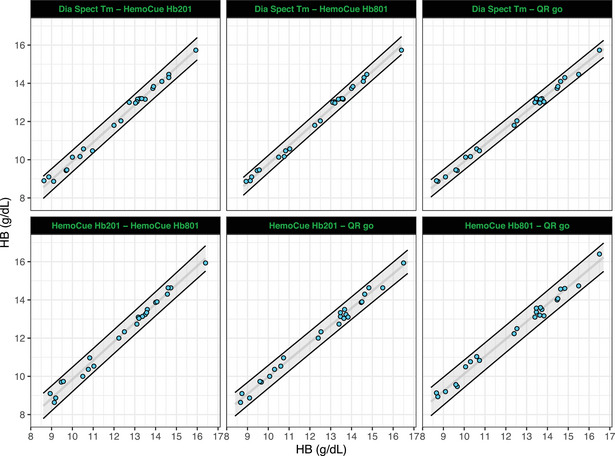
Scatter plots of the six IVD‐MD comparisons for the HB data set. The pointwise prediction intervals, depicted as gray regions, are estimated using the F–G approach.

The data from the HB data set raises an intriguing question: why do three out of six IVD‐MD comparisons (those excluding QR go) yield high $\hat{\zeta }$ values of 3.665, 5.687, and 6.110, while the remaining three result in $\hat{\zeta }$ values near 1? A simple examination of the IVD‐MD scatter plots (see Figure B1) does not provide a clear answer, as the dispersion of CS measurement results around the estimated Deming linear regression lines appears similar across both high and low $\hat{\zeta }$ value comparisons.

However, a more comprehensive interpretation becomes feasible when we combine the scatter plot analysis with the IVD‐MD repeatability CV estimates from Table 2. The table shows that the estimated CVs for the IVD‐MDs DiaSpect Tm, HemoCue Hb 201, and HemoCue Hb 801 are 0.490%, 0.513%, and 0.565%, respectively, while the estimated CV for QR go is significantly higher at 1.945%.

Under the assumption that DINS is absent, it would be reasonable to anticipate narrower pointwise prediction intervals for the IVD‐MD comparisons that do not involve QR go, particularly when contrasted with the remaining three IVD‐MD comparisons. However, upon inspection of Figure B1, the pointwise prediction intervals across all six comparisons reveals a remarkable degree of uniformity. This striking similarity implies a plausible presence of DINS within the IVD‐MD comparisons that do not include QR go. This inference is congruent with the observed elevated $\hat{\zeta }$ values, thereby supporting the stated hypothesis of DINS of those IVD‐MD comparisons not including QR go.

Within the CRP data set, $\hat{\zeta }$ values are generally high, IVD‐MD comparisons producing $\hat{\zeta }$ values ranging from 2.459 to 8.141. To investigate these considerable $\hat{\zeta }$ values, one might consider simulating data sets that emulate the IVD‐MD comparisons from the CRP data set, based on its statistical estimates documented in Table 2—an approach previously utilized in the Abbott Alinity versus Ortho Vitros comparison from the glucose data set. Nevertheless, a different perspective for elucidating these elevated $\hat{\zeta }$ values involves contrasting the widths of the pointwise Fuller–Gillard (F‐G) Deming regression prediction intervals (Sandberg et al. 2023) with the pointwise CLSI Deming regression prediction intervals (CLSI EP14 2022), as illustrated in Figures B2A and B2B. The Supporting Information of Sandberg et al. (2023) illustrates that, unlike the pointwise F–G prediction intervals, the pointwise CLSI prediction intervals do not account for DINS. As a result, narrower pointwise CLSI prediction intervals compared to pointwise F–G prediction intervals suggest presence of DINS. This phenomenon is demonstrated by the side‐by‐side comparison of Figure B2A,B, where the pointwise CLSI prediction intervals are generally narrower than the corresponding pointwise F–G prediction intervals across all 10 IVD‐MD comparisons. The differences in pointwise prediction interval widths are largely conspicuous, and especially for those comparisons associated with the largest $\hat{\zeta }$ values.

Systematic DINS is identified in a subset of the IVD‐MD comparisons within the CRP data set, as clearly delineated by the scatter plots featured in Figure B2A,B. In particular, the lower concentration interval reveals notable systematic DINS in the following IVD‐MD comparisons: Siemens Advia versus Abbott Architect, Siemens Advia versus Roche Cobas, Siemens Advia versus Siemens Dimension, Roche Cobas versus Ortho Vitros, and Siemens Dimension versus Ortho Vitros. In addition to the presence of systematic DINS in particular IVD‐MD comparisons, we also observe indications of random DINS across the majority of the IVD‐MD comparisons within the CRP data set. This conclusion is drawn from the observation that several CS measurements exceed the pointwise CLSI prediction interval limits showcased in Figure B2B.

## 4 Discussion

This paper sought to quantify DINS using a plug‐in estimator based on the theoretical measure ζ, incorporating components from the univariate OLS regression model. In addition, we introduced a criterion to detect excessive relative DINS by employing the average increase in pointwise prediction interval widths attributable to nonselectivity differences. Our primary goal is to demonstrate that the estimator for DINS correlates with DINS parameters, confirming its effectiveness for its intended use. Based on both simulated data and clinical data, we find that the estimator generally produces values exceeding 1 when IVD‐MD pairs exhibit DINS and proximates 1 when they are devoid of DINS, regardless of heteroskedasticity influences. This underscores the estimator's capability to detect, isolate, and quantify DINS.

Utilizing our proposed estimator for DINS, denoted $\hat{\zeta }$, which quantifies additional variability beyond the linear combination of repeatability IVD‐MD variances, we discovered its significant correlation with various DINS parameters in our simulation study—an expected finding. Specifically, ${\hat{\zeta }}_{0.99}$ demonstrated a positive correlation with both random DINS parameters, p and $m_{\max}$. Regarding systematic DINS, ${\hat{\zeta }}_{0.99}$ correlated positively with $m_{\max}$. ${\hat{\zeta }}_{0.99}$ also correlated with u−l, albeit not necessarily in a positive manner. Our observations led to the understanding that the ${\hat{\zeta }}_{0.99}$ and M relationship could represent those between ${\hat{\zeta }}_{0.99}$ and individual parameters of both random and systematic DINS.

Given the straightforward nature of the relationship between ${\hat{\zeta }}_{0.99}$ and M, we proposed employing M as an intuitive criterion for assessing whether the DINS for a IVD‐MD pair exceeded an acceptable magnitude, thereby simplifying the evaluation process. This approach enables us to define acceptable DINS levels using a single parameter, M, rather than a complex combination of parameters (p, $m_{\max}$ or u−l, $m_{\max}$). Equation (9) precisely illustrates the relationship between $\hat{\zeta }$ and M, which in turn allows us to derive a suitable rejection region for DINS using only M and the study design.

We further discovered the significant correlation between ${\hat{\zeta }}_{0.99}$ and the heteroskedasticity factor η, albeit not uniformly positive. The relationship is negative for η<1 and positive for η>1, implying a positive correlation between ${\hat{\zeta }}_{0.99}$ and |η−1|. However, it should be noted that this correlation is modest, and compared to those correlations observed for DINS, it is inconsiderable. Indeed, an increase in |η−1| primarily elevates the variance of $\hat{\zeta }$, thus introducing additional instability to the estimator, but does not necessarily produce larger values of $\hat{\zeta }$ on a general basis.

Our results highlight the influence of study design size, represented by n and R, on the effects of both DINS and heteroskedasticity on the variance of $\hat{\zeta }$. Importantly, while both DINS and heteroskedasticity influence the variance of $\hat{\zeta }$, only the presence of DINS affects its mean noticeably. Furthermore, larger study designs not only reduce the influence of both factors on the variance of $\hat{\zeta }$, but also its mean. However, the reduction of influence on the mean of $\hat{\zeta }$ is marginal compared to the influence on its variance. In addition, increasing the number of replicates is more effective in mitigating the effects of heteroskedasticity and DINS on the variance and mean of $\hat{\zeta }$, compared to increasing the number of CSs.

Upon scrutinizing the CS data, we observed high $\hat{\zeta }$ values in all 10 CRP IVD‐MD comparisons, three out of the six HB IVD‐MD comparisons, and one of the 10 glucose dataset comparisons. These elevated $\hat{\zeta }$ values are highly likely a product of DINS. However, an alternative explanation for these large $\hat{\zeta }$ values may be due to a potential limitation. This limitation involves the numerators of Equations (4) and (5). They may encompass the linear combination of reproducibility IVD‐MD variances, rather than the linear combination of repeatability IVD‐MD variances, or something in between. This situation could prove challenging as we generally anticipate reproducibility IVD‐MD variances to exceed repeatability IVD‐MD variances. Consequently, even if the IVD‐MDs do not exhibit DINS, the observed $\hat{\zeta }$ values might still be high. This situation could lead to our methodology erroneously flagging excessive DINS contrary to the actual circumstance. This potential bias may be a limitation in our approach for quantifying and detecting DINS. This is an additional reason to set a larger M value that encompasses both acceptable DINS levels and the additional variability resulting from reproducibility IVD‐MD variances exceeding repeatability IVD‐MD variances. However, determining the extent to which we should augment the M value beyond that representing acceptable DINS is not straightforward and necessitates further investigation.

To summarize, our proposed procedure to detect excessive DINS within a data set involving multiple IVD‐MD comparisons incorporates the following steps for each pair of IVD‐MDs:

**Procedure to detect excessive DINS**

1. Omit CSs that present outliers in the replicated measurements from the CS data. A standard method for this would be utilizing the studentized range test as demonstrated in (CLSI EP14 2022).
2. Select an M(%) value such that M(%)≥0%.
3. Review the study design and acquire the number of CSs (n) and the number of replicates (R) within the CS data.
4. Utilize n and R as derived from step 3, alongside the chosen M(%) from step 2, to determine ${\hat{\zeta }}_{\mathrm{upper}}$ either via a look‐up table or through Monte Carlo simulations.
5. Compute $\hat{\zeta }$ based on either raw or transformed CS data, choosing the transformation that results in a $\hat{\zeta }$ value most proximate to 1.
6. If $\hat{\zeta }>{\hat{\zeta }}_{\mathrm{upper}}$, the associated IVD‐MD pair is deemed to exhibit excessive DINS, while if $\hat{\zeta }\leq {\hat{\zeta }}_{\mathrm{upper}}$ the IVD‐MD pair is concluded to possess acceptable levels of DINS.

It is imperative to note that the methodology proposed herein for the identification of IVD‐MD pairs exhibiting excessive DINS is optimally effective when employed in analytical scenarios that utilize prediction intervals. Nevertheless, the applicability of this methodology is not restricted and may be extended to any context where empirical data pertaining to an IVD‐MD pair is available. For example, in instances where difference plots are employed for specific analytical reasons, the proposed methodology remains viable for flagging IVD‐MD pairs with excessive DINS. It is important to clarify that the criteria for determining the magnitude of DINS that is deemed acceptable or excessive, as operationalized through the variable M(%) in this paper, may vary when difference plots are the analytical tool of choice. Should a direct link be established between M(%) and another variable—designated as X(%)—that serves a similar function but offers more contextually appropriate interpretation and implications, the methodology can be adapted accordingly. In such cases, X(%) can be set and subsequently converted to its corresponding M(%), and the procedural steps numbered 2 through 6, as previously delineated, can be executed. However, the absence of a direct relationship between M(%) and X(%) could introduce complexities that must be judiciously addressed.

If such complexities cannot be bypassed, one could apply other methods to flag IVD‐MD pairs with excessive DINS. For example, Nilsson et al. (2018) utilizes an F‐test to detect IVD‐MD pairs with excessive random DINS based on the CS‐wise differences ${\bar{y}}_{i}-{\bar{x}}_{i}$. The null hypothesis of the test is however complete absence of random DINS within the regarded IVD‐MD pair, meaning that even a slight random DINS magnitude is considered inappropriate.

## 5 Detailed Methods and Extended Results

### 5.1 Software

The analyses, simulations, and results presentation throughout this paper were executed using the R software. Specifically, we utilized R version 4.3.1, codenamed “Beagle Scouts.” The software was operated on a Windows 11 x64 platform.

To streamline our project's simulations and calculations, we developed two custom R packages, commutability and fasteqa. These packages were designed explicitly with this research in mind and can be accessed for further inspection at https://github.com/pernille267/commutability and https://github.com/pernille267/fasteqa , respectively.

### 5.2 Simulation Results

Accompanying this paper is a supplementary material meticulously detailing each of the five simulation scenarios. This supplement elucidates the specific probability distributions employed and the simulation parameters for each scenario. Moreover, it lists the combinations of simulation parameter values harnessed in each distinct scenario. With a methodical approach, the simulation results for each scenario are sequentially documented. Through a complementary use of both plots and tables, readers are granted a comprehensive understanding of the results, paving the way for informed conclusions.

Here, in the main paper, only the 99% percentile result of $\hat{\zeta }$ is presented in numerical detail. However, the Supporting Information [Link], [Link] includes numerical details on many other statistics. These statistics pertain to the moments of $\hat{\zeta }$. such as the mean, variance, skewness, and kurtosis. They also include percentiles of $\hat{\zeta }$, specifically the 1%, 2.5%, 5%, 10%, 25%, 50%, 75%, 90%, 95%, 97.5%, and 99% percentiles. Each statistic is meticulously juxtaposed against the simulation parameters pertinent to each scenario, complemented by the sizes of study designs.

### 5.3 Sensitivity Analysis

The behavior of the estimator, $\hat{\zeta }$, in the absence of DINS between a pair of IVD‐MDs, is predicated on several assumptions. These include complete balanced data, with $\tau_{i}$ following a uniform distribution, $\tau_{i}∼\mathrm{Unif}\left(\mathrm{cil},\mathrm{ciu}\right)$; a linear relationship between $\xi_{i}$ and $\tau_{i}$, expressed as $\xi_{i}=\beta_{0}+\beta_{1}\cdot \tau_{i}$; and normally distributed error terms, $h_{ir}∼N\left(0,\sigma_{\mathrm{IVD}-{\mathrm{MD}}_{X}}^{2}\right)$ and $v_{ir}∼N\left(0,\sigma_{\mathrm{IVD}-{\mathrm{MD}}_{Y}}^{2}\right)$. To assess the robustness of $\hat{\zeta }$, we conducted a sensitivity analysis through simulations, detailed in the Supporting Information. The rationale for this analysis stems from the expectation that, in the absence of DINS, $\hat{\zeta }$ should demonstrate minimal dependence on the distributional assumptions of $\tau_{i}$, $h_{ir}$, and $v_{ir}$.

We examined the impact on $\hat{\zeta }$ under various conditions:

1. Missing data structures such as missing at random (MAR) and missing not at random (MNAR).
2. Alternative distributions for $\tau_{i}$, $h_{ir}$, and $v_{ir}$.

The primary aim of this sensitivity analysis is to ensure that elevated $\hat{\zeta }$ values observed in empirical data are attributable to DINS rather than artifacts of data structure or distributional characteristics. This approach enhances the reliability and interpretability of the $\hat{\zeta }$ estimator in practical applications involving IVD‐MD comparisons.

By systematically evaluating these factors, we can better understand the limitations and strengths of $\hat{\zeta }$ as an estimator, thereby improving its utility in clinical and research settings.

#### 5.3.1 Missing Data

The estimator $\hat{\zeta }$ is affected by missing data, primarily due to the resulting smaller effective study design. However, the impact is negligible when less than 5% of data is missing. In relevant applications of $\hat{\zeta }$, the occurrence of missing data is infrequent. In instances where missing data does occur, it is improbable that the proportion exceeds 5%.

#### 5.3.2 Distribution of $\tau_{i}$

The plug‐in estimator, $\hat{\zeta }$, demonstrates robustness to alternative distributions of $\tau_{i}$. The following distributions were tested, with parameters adjusted to match the 1% and 99% percentiles of unif(cil,ciu):

- Local‐scale *t*‐distribution (degrees of freedom: 5 and 15)
- Normal distribution
- Log‐normal distribution

Notably, the log‐normal distribution, which generates some extreme observations, did not significantly affect $\hat{\zeta }$, indicating its robustness to more extreme observations in $\tau_{i}$.

#### 5.3.3 Distribution of $h_{ir}$ and $v_{ir}$

Replacing the normal distribution of $h_{ir}$ and $v_{ir}$ with a location‐scale *t*‐distribution had minimal impact on $\hat{\zeta }$. Here, we used 5 and 15 degrees of freedom, but the location parameter of the location‐scale *t*‐distribution was set to 0.

#### 5.3.4 Conclusion

The plug‐in estimator $\hat{\zeta }$ demonstrates robustness to various assumption violations. While missing data can affect $\hat{\zeta }$ due to reduced effective study design, alternative distributions for $\tau_{i}$, $h_{ir}$, and $v_{ir}$ do not significantly alter its behavior. These findings suggest that the estimator's validity extends beyond the normal distribution assumption, enhancing its applicability in diverse statistical scenarios.

## Conflicts of Interest

The authors declare no conflicts of interest.

### Open Research Badges

This article has earned an Open Data badge for making publicly available the digitally‐shareable data necessary to reproduce the reported results. The data is available in the Supporting Information section.

This article has earned an open data badge “**Reproducible Research**” for making publicly available the code necessary to reproduce the reported results. The results reported in this article could fully be reproduced.

## Supporting information

Supporting Information

Supporting Information

## Data Availability

Clinical sample data sets are not shared due to confidentiality. The data that support the findings of this study are available in the Supporting Information of this article.

## Appendix A

### A.1 The Cluster‐Bootstrap Algorithm

The cluster‐bootstrap algorithm is defined by an algorithm that resamples clusters of observations alone rather than hierarchical resampling where both clusters and individual observations within each cluster are resampled. As deduced by Davison and Hinkley (1997), within the context of a balanced one‐way nested design, the exclusive resampling of clusters (clincial samples [CSs]) is optimal, averting the requirement for hierarchical resampling. This conclusion was reached through a comparison indicating that the expected variance and covariance of the cluster‐bootstrap samples are closer to those of the original data set, compared to hierarchical resampling. The applicability of this finding extends to unbalanced one‐way nested designs as well (Ren et al. 2010). Consequently, despite variations in the number of replicates among the CSs, cluster resampling remains preferential over hierarchical resampling.

By denoting B as the number of bootstrap replications, the cluster‐bootstrap algorithm can be stated as follows:

For b=1,…,B:

1. Extract n CSs with replacement from the original data set to generate the bth resampled CS data set.
2. Compute ${\hat{\zeta }}_{b}$ utilizing the bth resampled CS data set.

We can calculate the mean, variance, coefficient of variation (CV), and median absolute deviation (MAD) of $\hat{\zeta }$ based on the set of B cluster‐bootstrap replicates of $\hat{\zeta }$, denoted as ${\hat{\zeta }}_{1},{\hat{\zeta }}_{2},\text{…},{\hat{\zeta }}_{B}$. These calculations use the B bootstrap replicates and their arithmetic average, represented as ${\bar{\hat{\zeta }}}_{b}$:

$$\begin{array}{cc} \hat{E}\left[\hat{\zeta }\right] & ={\bar{\hat{\zeta }}}_{b}\\ \hat{\mathrm{Var}}\left[\hat{\zeta }\right] & =\frac{1}{B-1}\sum_{b=1}^{B}{\left({\hat{\zeta }}_{b}-{\bar{\hat{\zeta }}}_{b}\right)}^{2}\\ \hat{\mathrm{CV}}\left[\hat{\zeta }\right] & =\frac{\hat{\mathrm{SD}}\left[\hat{\zeta }\right]}{\hat{E}\left[\hat{\zeta }\right]}\\ \hat{\mathrm{MAD}}\left[\hat{\zeta }\right] & =\mathrm{median}|{\hat{\zeta }}_{b}-\hat{\mathrm{median}}\left[\hat{\zeta }\right]|. \end{array}$$

We can also use the cluster‐bootstrap method to estimate the median of $\hat{\zeta }$, denoted as $\hat{\mathrm{median}}\left[\hat{\zeta }\right]$, as well as other quantiles and confidence intervals for $\hat{\zeta }$. To do this, we rank the bootstrap replicates ${\hat{\zeta }}_{1},{\hat{\zeta }}_{2},\text{…},{\hat{\zeta }}_{B}$ in order, resulting in ${\hat{\zeta }}_{\left(1\right)},{\hat{\zeta }}_{\left(2\right)},\text{…},{\hat{\zeta }}_{\left(B\right)}$. These ordered values can then be used to compute percentile estimates for $\hat{\zeta }$. For instance, if we have B=1000 bootstrap replicates, we can estimate the median, lower quartile, and upper quartile of $\hat{\zeta }$ as follows:

$$\begin{array}{cc} \hat{\mathrm{median}}\left[\hat{\zeta }\right] & ={\hat{\zeta }}_{\left(500\right)}\\ \hat{{\hat{\zeta }}_{0.25}} & ={\hat{\zeta }}_{\left(250\right)}\\ \hat{{\hat{\zeta }}_{0.75}} & ={\hat{\zeta }}_{\left(750\right)}. \end{array}$$

Within the same set of resampled $\hat{\zeta }$ values, we can also estimate the 95% bootstrap percentile confidence intervals (BPCI) for $\hat{\zeta }$. To do this, we use the 25th smallest observation as the lower limit and the 25th largest observation as the upper limit, following the method proposed by Rizzo (2007):

$${\hat{\mathrm{CI}}}_{\hat{\zeta }}=\left[{\hat{\zeta }}_{\left(25\right)},{\hat{\zeta }}_{\left(975\right)}\right].$$

## Appendix B

### B.2 Look‐up Table for Rejection Region of Acceptable Differences in Nonselectivity

**TABLE B1:** Look‐up table showcasing presimulated values of ${\left[\hat{\zeta }|M\left(\%\right)\right]}_{0.99}$ based on the most typical study designs and upper bounds of acceptable differences in nonselectivity, M(%).

CSs	*M*(%)																				
*n*	0%	5%	10%	15%	20%	25%	30%	35%	40%	45%	50%	55%	60%	65%	70%	75%	80%	85%	90%	95%	100%
*R* **= Two replicates**																					
20	1.901	2.101	2.307	2.518	2.748	2.975	3.224	3.472	3.735	4.004	4.286	4.578	4.871	5.187	5.506	5.847	6.172	6.517	6.878	7.241	7.619
21	1.872	2.066	2.268	2.479	2.693	2.923	3.166	3.408	3.664	3.928	4.209	4.496	4.797	5.087	5.412	5.732	6.070	6.407	6.750	7.117	7.465
22	1.840	2.029	2.230	2.434	2.647	2.877	3.110	3.358	3.615	3.863	4.141	4.422	4.718	5.012	5.316	5.645	5.969	6.304	6.638	7.004	7.351
23	1.816	1.998	2.196	2.402	2.615	2.834	3.064	3.301	3.561	3.817	4.083	4.355	4.641	4.928	5.229	5.555	5.880	6.195	6.544	6.893	7.246
24	1.787	1.969	2.162	2.363	2.576	2.796	3.013	3.259	3.498	3.753	4.022	4.293	4.576	4.852	5.168	5.468	5.793	6.115	6.458	6.796	7.145
25	1.765	1.947	2.137	2.331	2.535	2.759	2.978	3.211	3.460	3.701	3.971	4.238	4.511	4.799	5.092	5.393	5.719	6.043	6.365	6.698	7.053
26	1.743	1.921	2.111	2.304	2.507	2.725	2.944	3.179	3.424	3.658	3.921	4.181	4.459	4.737	5.028	5.326	5.642	5.964	6.287	6.619	6.972
27	1.724	1.898	2.082	2.280	2.481	2.691	2.912	3.137	3.380	3.611	3.869	4.139	4.408	4.695	4.986	5.275	5.578	5.886	6.223	6.547	6.886
28	1.703	1.878	2.063	2.250	2.454	2.661	2.878	3.100	3.339	3.580	3.829	4.098	4.356	4.634	4.924	5.210	5.519	5.828	6.152	6.480	6.814
29	1.688	1.858	2.038	2.231	2.428	2.634	2.845	3.072	3.300	3.543	3.790	4.049	4.311	4.579	4.871	5.166	5.458	5.767	6.080	6.414	6.747
30	1.670	1.839	2.021	2.207	2.403	2.606	2.825	3.040	3.274	3.510	3.753	4.008	4.271	4.552	4.825	5.108	5.399	5.713	6.020	6.344	6.663
32	1.639	1.807	1.986	2.169	2.364	2.561	2.772	2.989	3.212	3.445	3.689	3.943	4.199	4.462	4.745	5.018	5.305	5.611	5.929	6.233	6.564
34	1.612	1.778	1.952	2.134	2.321	2.522	2.725	2.941	3.163	3.387	3.633	3.875	4.133	4.391	4.662	4.944	5.220	5.522	5.817	6.131	6.459
35	1.599	1.766	1.940	2.117	2.303	2.501	2.707	2.915	3.140	3.369	3.602	3.846	4.100	4.361	4.628	4.900	5.187	5.481	5.772	6.091	6.398
36	1.589	1.752	1.922	2.103	2.290	2.480	2.686	2.899	3.113	3.338	3.581	3.821	4.067	4.326	4.590	4.869	5.149	5.439	5.739	6.048	6.360
38	1.569	1.731	1.896	2.075	2.255	2.447	2.651	2.860	3.071	3.300	3.525	3.766	4.013	4.267	4.533	4.806	5.092	5.369	5.654	5.963	6.273
40	1.549	1.709	1.874	2.048	2.233	2.422	2.615	2.820	3.033	3.257	3.488	3.717	3.966	4.210	4.481	4.742	5.023	5.300	5.591	5.887	6.194
45	1.507	1.662	1.824	1.995	2.172	2.357	2.546	2.745	2.954	3.172	3.392	3.623	3.856	4.106	4.363	4.615	4.885	5.157	5.442	5.730	6.039
50	1.475	1.625	1.785	1.950	2.123	2.305	2.492	2.689	2.888	3.097	3.317	3.543	3.775	4.015	4.261	4.515	4.779	5.046	5.325	5.609	5.898
*R* **= Three replicates**																					
20	1.507	1.660	1.822	1.992	2.171	2.356	2.547	2.749	2.953	3.170	3.387	3.620	3.857	4.098	4.353	4.614	4.880	5.151	5.435	5.723	6.027
21	1.491	1.643	1.804	1.970	2.145	2.333	2.522	2.717	2.922	3.137	3.359	3.581	3.820	4.060	4.312	4.573	4.833	5.102	5.384	5.671	5.963
22	1.477	1.628	1.786	1.957	2.128	2.308	2.496	2.692	2.892	3.108	3.322	3.548	3.783	4.022	4.263	4.521	4.786	5.055	5.323	5.617	5.911
23	1.463	1.613	1.772	1.935	2.108	2.286	2.473	2.668	2.867	3.074	3.295	3.514	3.747	3.982	4.231	4.480	4.741	5.012	5.287	5.570	5.854
24	1.452	1.601	1.755	1.918	2.090	2.268	2.452	2.644	2.842	3.055	3.262	3.490	3.715	3.949	4.193	4.445	4.701	4.970	5.240	5.518	5.810
25	1.440	1.588	1.742	1.906	2.073	2.251	2.434	2.624	2.820	3.028	3.240	3.459	3.685	3.921	4.164	4.410	4.664	4.929	5.196	5.476	5.760
26	1.429	1.576	1.728	1.891	2.059	2.235	2.416	2.605	2.803	3.007	3.217	3.437	3.657	3.896	4.132	4.378	4.631	4.896	5.163	5.435	5.716
27	1.421	1.566	1.717	1.877	2.044	2.219	2.397	2.588	2.782	2.985	3.196	3.412	3.635	3.871	4.105	4.348	4.602	4.859	5.123	5.402	5.679
28	1.411	1.555	1.708	1.865	2.032	2.203	2.385	2.571	2.763	2.964	3.175	3.391	3.607	3.845	4.074	4.324	4.572	4.830	5.097	5.360	5.643
29	1.402	1.545	1.696	1.855	2.018	2.190	2.370	2.555	2.750	2.948	3.156	3.368	3.592	3.818	4.054	4.294	4.544	4.798	5.061	5.327	5.609
30	1.394	1.538	1.686	1.842	2.008	2.177	2.357	2.543	2.733	2.932	3.136	3.351	3.571	3.795	4.030	4.269	4.518	4.773	5.030	5.302	5.577
32	1.380	1.521	1.671	1.825	1.986	2.156	2.331	2.513	2.704	2.900	3.104	3.316	3.530	3.754	3.987	4.224	4.470	4.722	4.977	5.247	5.516
34	1.366	1.506	1.653	1.804	1.967	2.135	2.307	2.490	2.680	2.872	3.074	3.282	3.499	3.719	3.949	4.182	4.425	4.675	4.930	5.191	5.463
35	1.359	1.499	1.646	1.796	1.957	2.126	2.299	2.479	2.665	2.858	3.060	3.270	3.481	3.704	3.928	4.163	4.404	4.654	4.905	5.178	5.440
36	1.354	1.494	1.641	1.790	1.949	2.118	2.288	2.468	2.654	2.847	3.047	3.252	3.468	3.686	3.911	4.149	4.387	4.632	4.887	5.151	5.417
38	1.343	1.481	1.626	1.776	1.934	2.099	2.271	2.448	2.633	2.827	3.024	3.230	3.440	3.655	3.881	4.114	4.355	4.597	4.848	5.113	5.367
40	1.333	1.470	1.614	1.765	1.921	2.083	2.253	2.429	2.616	2.804	3.002	3.202	3.413	3.629	3.855	4.086	4.323	4.564	4.815	5.074	5.336
45	1.313	1.447	1.589	1.735	1.890	2.049	2.219	2.391	2.571	2.758	2.954	3.154	3.361	3.571	3.792	4.020	4.251	4.494	4.740	4.986	5.251
50	1.295	1.428	1.568	1.713	1.866	2.025	2.190	2.361	2.539	2.723	2.915	3.112	3.316	3.529	3.742	3.965	4.196	4.435	4.677	4.926	5.176
*R* **= Four replicates**																					
20	1.378	1.519	1.668	1.822	1.983	2.152	2.329	2.508	2.700	2.896	3.097	3.311	3.530	3.753	3.979	4.222	4.466	4.715	4.973	5.243	5.509
21	1.367	1.508	1.655	1.807	1.969	2.136	2.311	2.492	2.679	2.873	3.076	3.282	3.501	3.723	3.952	4.187	4.433	4.677	4.938	5.196	5.471
22	1.358	1.497	1.643	1.795	1.955	2.120	2.295	2.474	2.661	2.855	3.053	3.261	3.476	3.697	3.921	4.158	4.403	4.646	4.901	5.162	5.427
23	1.348	1.487	1.632	1.784	1.942	2.107	2.279	2.459	2.646	2.837	3.035	3.240	3.456	3.672	3.899	4.131	4.372	4.617	4.865	5.130	5.395
24	1.340	1.478	1.621	1.774	1.931	2.094	2.265	2.442	2.627	2.821	3.018	3.222	3.437	3.650	3.877	4.107	4.344	4.588	4.836	5.100	5.362
25	1.333	1.470	1.613	1.764	1.920	2.082	2.252	2.431	2.612	2.802	2.999	3.200	3.413	3.629	3.855	4.078	4.314	4.561	4.810	5.071	5.329
26	1.326	1.461	1.604	1.753	1.908	2.072	2.239	2.417	2.598	2.784	2.984	3.187	3.393	3.609	3.832	4.062	4.298	4.537	4.784	5.040	5.304
27	1.320	1.455	1.596	1.745	1.900	2.062	2.229	2.402	2.587	2.773	2.970	3.170	3.378	3.591	3.816	4.039	4.272	4.512	4.761	5.019	5.276
28	1.313	1.447	1.588	1.737	1.890	2.052	2.218	2.394	2.572	2.758	2.956	3.156	3.362	3.574	3.795	4.021	4.250	4.493	4.744	4.993	5.252
29	1.307	1.440	1.581	1.729	1.882	2.043	2.211	2.381	2.562	2.750	2.941	3.140	3.347	3.561	3.778	4.003	4.238	4.476	4.716	4.970	5.232
30	1.302	1.434	1.575	1.721	1.874	2.033	2.200	2.373	2.553	2.736	2.928	3.127	3.330	3.543	3.762	3.987	4.216	4.454	4.696	4.948	5.205
32	1.291	1.423	1.563	1.709	1.860	2.017	2.182	2.354	2.532	2.719	2.906	3.103	3.306	3.516	3.731	3.958	4.179	4.420	4.663	4.912	5.167
34	1.282	1.414	1.553	1.696	1.848	2.004	2.168	2.338	2.513	2.696	2.885	3.082	3.284	3.490	3.706	3.928	4.155	4.390	4.631	4.880	5.129
35	1.278	1.411	1.547	1.690	1.841	1.998	2.160	2.329	2.505	2.688	2.875	3.071	3.272	3.479	3.696	3.911	4.139	4.374	4.613	4.860	5.112
36	1.275	1.405	1.542	1.686	1.835	1.991	2.154	2.323	2.497	2.679	2.866	3.062	3.263	3.469	3.684	3.901	4.128	4.361	4.601	4.844	5.096
38	1.266	1.396	1.532	1.676	1.825	1.978	2.142	2.309	2.483	2.664	2.851	3.044	3.242	3.451	3.657	3.878	4.103	4.338	4.572	4.817	5.063
40	1.260	1.389	1.524	1.667	1.814	1.971	2.129	2.296	2.471	2.649	2.836	3.027	3.227	3.432	3.640	3.862	4.083	4.314	4.548	4.790	5.042
45	1.246	1.373	1.507	1.649	1.793	1.946	2.105	2.270	2.441	2.619	2.803	2.992	3.189	3.391	3.603	3.813	4.037	4.259	4.494	4.738	4.979
50	1.234	1.360	1.492	1.633	1.777	1.927	2.085	2.249	2.419	2.595	2.777	2.963	3.158	3.358	3.564	3.778	3.996	4.222	4.458	4.693	4.936
*R* **= Five replicates**																					
20	1.314	1.448	1.590	1.738	1.893	2.053	2.222	2.393	2.574	2.763	2.957	3.155	3.363	3.576	3.798	4.023	4.255	4.497	4.744	4.994	5.253
21	1.306	1.440	1.582	1.727	1.880	2.040	2.208	2.382	2.560	2.745	2.939	3.136	3.342	3.554	3.774	3.999	4.228	4.469	4.716	4.966	5.220
22	1.299	1.431	1.571	1.718	1.870	2.029	2.195	2.365	2.544	2.729	2.923	3.119	3.322	3.534	3.752	3.979	4.206	4.446	4.692	4.940	5.194
23	1.291	1.424	1.564	1.708	1.859	2.019	2.182	2.353	2.531	2.715	2.908	3.102	3.308	3.515	3.735	3.953	4.184	4.422	4.663	4.909	5.166
24	1.286	1.417	1.555	1.698	1.851	2.008	2.174	2.342	2.518	2.704	2.891	3.086	3.291	3.499	3.715	3.936	4.163	4.398	4.644	4.887	5.140
25	1.279	1.411	1.548	1.691	1.842	1.998	2.162	2.332	2.506	2.690	2.879	3.073	3.277	3.480	3.697	3.919	4.147	4.379	4.619	4.866	5.119
26	1.274	1.404	1.541	1.684	1.835	1.992	2.154	2.321	2.496	2.679	2.864	3.060	3.260	3.466	3.680	3.900	4.130	4.361	4.597	4.847	5.094
27	1.268	1.400	1.535	1.677	1.827	1.983	2.143	2.312	2.486	2.666	2.855	3.047	3.246	3.453	3.668	3.886	4.111	4.342	4.581	4.824	5.073
28	1.264	1.394	1.528	1.671	1.820	1.975	2.135	2.304	2.477	2.659	2.844	3.036	3.237	3.441	3.652	3.870	4.097	4.325	4.565	4.807	5.054
29	1.259	1.388	1.523	1.665	1.814	1.968	2.129	2.296	2.470	2.649	2.834	3.027	3.225	3.428	3.640	3.857	4.081	4.308	4.547	4.786	5.038
30	1.254	1.384	1.519	1.660	1.807	1.961	2.120	2.286	2.461	2.640	2.822	3.015	3.213	3.416	3.626	3.845	4.067	4.298	4.531	4.771	5.020
32	1.247	1.375	1.510	1.648	1.795	1.949	2.108	2.274	2.445	2.623	2.806	2.998	3.192	3.398	3.608	3.819	4.041	4.267	4.503	4.743	4.989
34	1.241	1.368	1.501	1.641	1.786	1.940	2.096	2.261	2.430	2.607	2.791	2.982	3.175	3.376	3.584	3.802	4.017	4.246	4.478	4.717	4.963
35	1.237	1.365	1.497	1.636	1.781	1.934	2.092	2.255	2.426	2.601	2.782	2.972	3.166	3.368	3.576	3.790	4.009	4.233	4.466	4.704	4.948
36	1.234	1.361	1.494	1.632	1.777	1.929	2.088	2.250	2.418	2.594	2.779	2.966	3.161	3.361	3.566	3.780	4.001	4.226	4.453	4.696	4.935
38	1.228	1.354	1.486	1.624	1.770	1.918	2.077	2.239	2.406	2.583	2.766	2.953	3.143	3.346	3.550	3.763	3.981	4.204	4.437	4.671	4.916
40	1.224	1.348	1.480	1.618	1.762	1.910	2.067	2.229	2.398	2.571	2.753	2.939	3.134	3.331	3.537	3.748	3.965	4.187	4.419	4.653	4.894
45	1.212	1.336	1.467	1.603	1.745	1.895	2.049	2.209	2.377	2.548	2.727	2.911	3.101	3.300	3.502	3.710	3.929	4.148	4.374	4.608	4.851
50	1.203	1.327	1.455	1.592	1.733	1.881	2.034	2.192	2.357	2.530	2.706	2.891	3.081	3.274	3.477	3.684	3.900	4.117	4.343	4.577	4.812
