# Supplementary material for: Quantification of Difference in Nonselectivity Between In Vitro Diagnostic Medical Devices
Source: Biom J. 2025 Jan 2;67(1):e70032. doi: 10.1002/bimj.70032 (PMC11695778; doi:10.1002/bimj.70032)
Supplement: Supplementary file 1 — Supporting Information [file BIMJ-67-e70032-s001.zip › Reproducibility resubmission v2/README.pdf]

README

Pernille Kjeilen Fauskanger[a,b*] Sverre Sandberg[a,c,d] Jesper Johansen[e]
Thomas Keller[f] Jeffrey Budd[g] W. Greg Miller[h] Anne Stavelin[a]
Vincent Delatour[i] Mauro Panteghini[j] Bård Støve[b]

1 Reproducing results in the supplemental file and manuscript

1. Introduction
   - This research aims to quantify differences in non-selectivity between in vitro diagnostic medical devices, and setting a threshold for unacceptable differences in non-selectivity using the average relative increase in prediction interval widths as a measure of what is considered acceptable to the end-user.
   - In this folder, you will find two `R` markdown documents, one `LaTeX` file, one folder containing real data and one folder containing seven empty folders. You will also find two folders containing simulation results that were generated by the main author Pernille Fauskanger 22. October 2024.
2. Software Needed
   - `R` version 4.3.1, with the following packages installed: `base`, `stats`, `data.table`, `stringi`, `ggplot2`, `readxl`, `utils`, `moments`, `devtools`, `pbapply`, `parallel`, `kableExtra`, `fasteqa` and `commutability`. You do not need to install these manually, as they will be automatically installed when running the **`setup`** chunks in `Supplemental file repr.Rmd` and `Reproducing manuscript results.Rmd`
   - The latest version of `Rstudio`, `Rtools`, `tinytex`, `rmarkdown` and `bookdown`
   - `MiKTeX` with installed packages `bbm`, `nicefrac`, `xcolor`, `mathtools`, `float`, `tcolorbox`, `soul` and `setspace`
3. Files and Folders
   - `Supplemental file repr.Rmd`: Contains scripts for reproducing the supplemental file tables and plots
   - `Reproducing manuscript results.Rmd`: Contains scripts for reproducing the manuscript tables and plots
   - `header.tex`: Latex syntax for changing labeling of tables and plots. Can be deleted if this file creates problems. However, if it is deleted, ensure that you delete lines 17-18 of `Supplemental file repr.Rmd` and `Reproducing manuscript results.Rmd` too.
   - `real data/`: Contains clinical sample data for glucose, hemoglobin and C-reactive protein.
   - `simulation results relevant/`: Contains seven empty folders, and each will be filled with simulation results after successfully knitting `Supplemental file repr.Rmd`
   - `results pkf 22 10 2024 7 cores/`: This folder contains all results generated by the main author, Pernille Fauskanger, on October 22, 2024. If you are using 7 cores for parallelization, compare your outputs with the contents of this folder.
   - `results pkf 22 10 2024 15 cores/`: This folder contains all results generated by the main author, Pernille Fauskanger, on October 22, 2024. If you are using 15 cores for parallelization, compare your outputs with the contents of this folder.
4. How to Run the Code
   - Step 1: Open `Supplemental file repr.Rmd`
   - Step 2: Go to **`setup`** chunk.
   - Step 3: Assign either **`ncores`** $\leftarrow$ **`15`** or **`ncores`** $\leftarrow$ **`7`** (line 146), depending on the computer's

specifications. If the computer has fewer than 16 cores, set `ncores` to 15. If it has between 8 and 14 cores (inclusive), set `ncores` to 7.

- Step 4: Assign `base_path` to the path of the `simulation results relevant` folder ending with a back slash (line 159). E.g., `base_path <- ".../ simulation results relevant/"`
- Step 5: Run the `setup` chunk to ensure that all packages are correctly installed and loaded. An error is thrown if at least one required package is not correctly installed. If problems occurs trying to install the development packages `fasteqa` and `commutability`, ensure you have the latest version of `Rtools` installed. If problems still persists, restart `R` and try again.
- Step 6: Knit the document. The knitting may take some minutes due to the size of the simulation study. The simulation results of the supplemental file are found in this pdf.
- Step 7: Open `Reproducing manuscript results.Rmd`
- Step 8: Go to the `setup` chunk
- Step 9: Run the `setup` chunk to ensure that all packages are correctly installed and loaded. You will get an error if this is not the case.
- Step 10: Go to the `percentiles-of-zeta-1-2-5` chunk
- Step 11: Assign `base_path` to the path of the `simulation results relevant` folder ending with a back slash (line 436). E.g., `base_path <- ".../ simulation results relevant/"`
- Step 12: Go to the `clinical-data-analysis` chunk
- Step 13: Assign `base_path` to the path of the `real data/` folder ending with a back slash (line 878). E.g., `base_path <- ".../ real data/"`
- Step 14: Knit the document. The knitting may take some minutes due to the number of required calculations and simulations. The plots and tables found in the original manuscript are found in the pdf document generated from knitting this file.

5. Expected Results
   - The figures and tables of the supplemental file will be knitted to a pdf document named `Supplemental-file-repr.pdf`.
   - The figures and tables of the manuscript will be knitted to a pdf document named `Reproducing-manuscript-results.pdf`.
   - The figures of the supplemental file can be found separately in the `Supplemental-files-repr_files` folder.
   - The figures of the manuscript can be found separately in the `Reproducing-manuscript-results_files` folder.
   - The raw simulation results from the supplemental file for each simulation setting is to be found in the `simulation results relevant` folder.
6. Notes
   - To ensure reproducibility, use the same number of cores for parallelization as was used to generate the results in either `results pkf 22 10 2024 7 cores/` or `results pkf 22 10 2024 15 cores/`. If the computer has fewer than 8 available cores, please contact us using the information provided below. In such cases, the main author will need to create new results based on the chosen core configuration.
   - The simulation results presented in the supplemental file are originally based on $N = 10^6$ replicates for each simulation parameter combination. Consequently, even with parallel computation using 27 cores, the run time of the simulations are around 8 hours. Therefore, we should instead focus on $N = 10^4$, which is likely to complete within 30 to 60 minutes using a standard computer.
   - For some reason, Table 2 and Table 3 of the manuscript are not reproducible when knitting `Reproducing manuscript results.Rmd`, but the results are reproducible in the console when running the `dataset-descriptions` and `descriptive-statistics-zeta` chunks. Thus, there are probably some reproducibility issues uniquely to Rmarkdown handling the `resample_samples()` function of the `fasteqa` package. However, the general conclusions are still the same, even if the numbers are slightly different.
   - The original manuscript results were generated using 27 cores, while the reproducibility study utilized fewer cores (7 or 15). This difference in computational resources may lead to slight variations in the results.
   - The original manuscript includes results from $N = 10^6$ replicated simulations, whereas the

reproducibility study was conducted with $N = 10^4$ replications. This difference in the number of iterations can also contribute to discrepancies between the figures and tables in the original manuscript and those obtained in the reproducibility study.

7. Contact Information
    - Pernille Kjeilen Fauskanger (pernille.fauskanger@noklus.no)

2 Other information

2.1 Session information

After loading the necessary packages, we end up the following output of `sessionInfo()` in R:

```r
sessionInfo()
```

```
## R version 4.4.2 (2024-10-31 ucrt)
## Platform: x86_64-w64-mingw32/x64
## Running under: Windows 11 x64 (build 22631)
##
## Matrix products: default
##
##
## locale:
## [1] LC_COLLATE=Norwegian Bokmål_Norway.utf8
## [2] LC_CTYPE=Norwegian Bokmål_Norway.utf8
## [3] LC_MONETARY=Norwegian Bokmål_Norway.utf8
## [4] LC_NUMERIC=C
## [5] LC_TIME=Norwegian Bokmål_Norway.utf8
##
## time zone: Europe/Oslo
## tzcode source: internal
##
## attached base packages:
## [1] parallel  stats     graphics  grDevices utils     datasets  methods
## [8] base
##
## other attached packages:
##  [1] bookdown_0.41        rmarkdown_2.29       tinytex_0.54
##  [4] boot_1.3-31          kableExtra_1.4.0     pbapply_1.7-2
##  [7] commutability_0.1.0  fasteqa_1.0          devtools_2.4.5
## [10] usethis_3.0.0        moments_0.14.1       readxl_1.4.3
## [13] ggplot2_3.5.1        microbenchmark_1.5.0 stringi_1.8.4
## [16] data.table_1.16.2
##
## loaded via a namespace (and not attached):
##  [1] tidyselect_1.2.1         viridisLite_0.4.2        dplyr_1.1.4
##  [4] fastmap_1.2.0            promises_1.3.0           digest_0.6.37
##  [7] mime_0.12                lifecycle_1.0.4          ellipsis_0.3.2
## [10] ROI.plugin.qpoases_1.0-3 magrittr_2.0.3           compiler_4.4.2
## [13] skedastic_2.0.2          rlang_1.1.4              tools_4.4.2
## [16] utf8_1.2.4               yaml_2.3.10              knitr_1.49
## [19] htmlwidgets_1.6.4        pkgbuild_1.4.5           xml2_1.3.6
## [22] pkgload_1.4.0            miniUI_0.1.1.1           registry_0.5-1
## [25] withr_3.0.2              purrr_1.0.2              numDeriv_2016.8-1.1
## [28] grid_4.4.2               fansi_1.0.6              urlchecker_1.0.1
## [31] profvis_0.4.0            xtable_1.8-4             colorspace_2.1-1
```

```
## [34] scales_1.3.0           cli_3.6.3                 generics_0.1.3
## [37] remotes_2.5.0          rstudioapi_0.17.1         sessioninfo_1.2.2
## [40] cachem_1.1.0           stringr_1.5.1             splines_4.4.2
## [43] cellranger_1.1.0       vctrs_0.6.5               Matrix_1.7-1
## [46] slam_0.1-55            systemfonts_1.1.0         glue_1.8.0
## [49] ROI_1.0-1              smooth.commutability_1.0 gtable_0.3.6
## [52] later_1.3.2            munsell_0.5.1             tibble_3.2.1
## [55] pillar_1.9.0           htmltools_0.5.8.1         R6_2.5.1
## [58] Rdpack_2.6.2           evaluate_1.0.1            shiny_1.9.1
## [61] lattice_0.22-6         rbibutils_2.3             backports_1.5.0
## [64] memoise_2.0.1          httpuv_1.6.15             Rcpp_1.0.13-1
## [67] svglite_2.1.3          nlme_3.1-166              checkmate_2.3.2
## [70] mgcv_1.9-1             xfun_0.49                 fs_1.6.5
## [73] pkgconfig_2.0.3
```

2.2 Commit hashes

The packages `fasteqa` and `commutability` is only available on Github. Their relevant commit hashes are `adea38b` and `c47a2d1`.
