# Supplementary material for: Quantification of Difference in Nonselectivity Between In Vitro Diagnostic Medical Devices
Source: Biom J. 2025 Jan 2;67(1):e70032. doi: 10.1002/bimj.70032 (PMC11695778; doi:10.1002/bimj.70032)
Supplement: Supplementary file 1 — Supporting Information [file BIMJ-67-e70032-s001.zip › Reproducibility resubmission v2/results pkf 22 10 2024 15 cores/Reproducing-manuscript-results.pdf]

Reproducing Figures and Tables of Manuscript

Supplemental files

Pernille Kjeilen Fauskanger[a,b*] Sverre Sandberg[a,c,d] Jesper Johansen[e]
Thomas Keller[f] Jeffrey Budd[g] W. Greg Miller[h] Anne Stavelin[a]
Vincent Delatour[i] Mauro Panteghini[j] Bård Støve[b]

[*] Corresponding author; E-mail: pernille.fauskanger@noklus.no

[a] Norwegian Organization for Quality Improvement of Laboratory Examinations (Noklus), Haraldsplass Deaconess Hospital, Bergen, Norway.

[b] Department of Mathematics, University of Bergen, Bergen, Norway.

[c] Department of Global Public Health and Primary Care, University of Bergen, Bergen, Norway.

[d] Department of Medical Biochemistry and Pharmacology, Haukeland University Hospital, Bergen, Norway.

[e] Radiometer Medical ApS, Copenhagen, Denmark.

[f] ACOMED Statistic, Leipzig, Germany.

[g] Jeff Budd Consulting, St. Paul, MN, United States.

[h] Department of Pathology, Virginia Commonwealth University, Richmond, VA, United States.

[i] Laboratoire national de métrologie et d'essais, Paris, France.

[j] Department of Laboratory Medicine, Ludwik Rydygier Collegium Medicum in Bydgoszcz, Nicolaus Copernicus University in Torun, Torun, Poland.

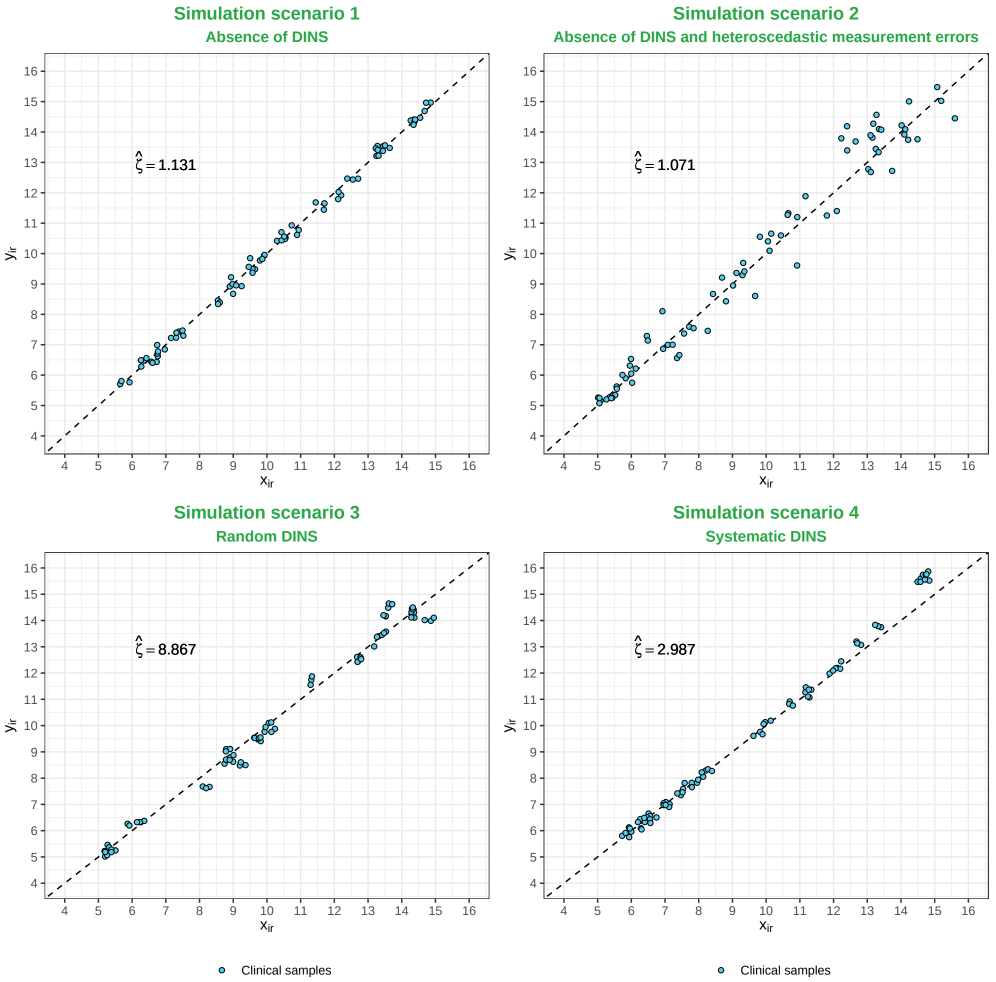

Figure 1. Visual representations of the simulation scenarios 1-4, along with their respective computed $\hat{\zeta}$ values for one realization. The blue-filled circles signify individual pairs of IVD-MD measurements (x_{ir}, y_{ir}). The black dashed lines in the background illustrate the lines of equivalence, defined by an intercept of zero and a slope of one.

0.1 Figure 1

Reproducing Figure 1 of the manuscript.

0.2 Figure 2

Reproducing Figure 2 in manuscript

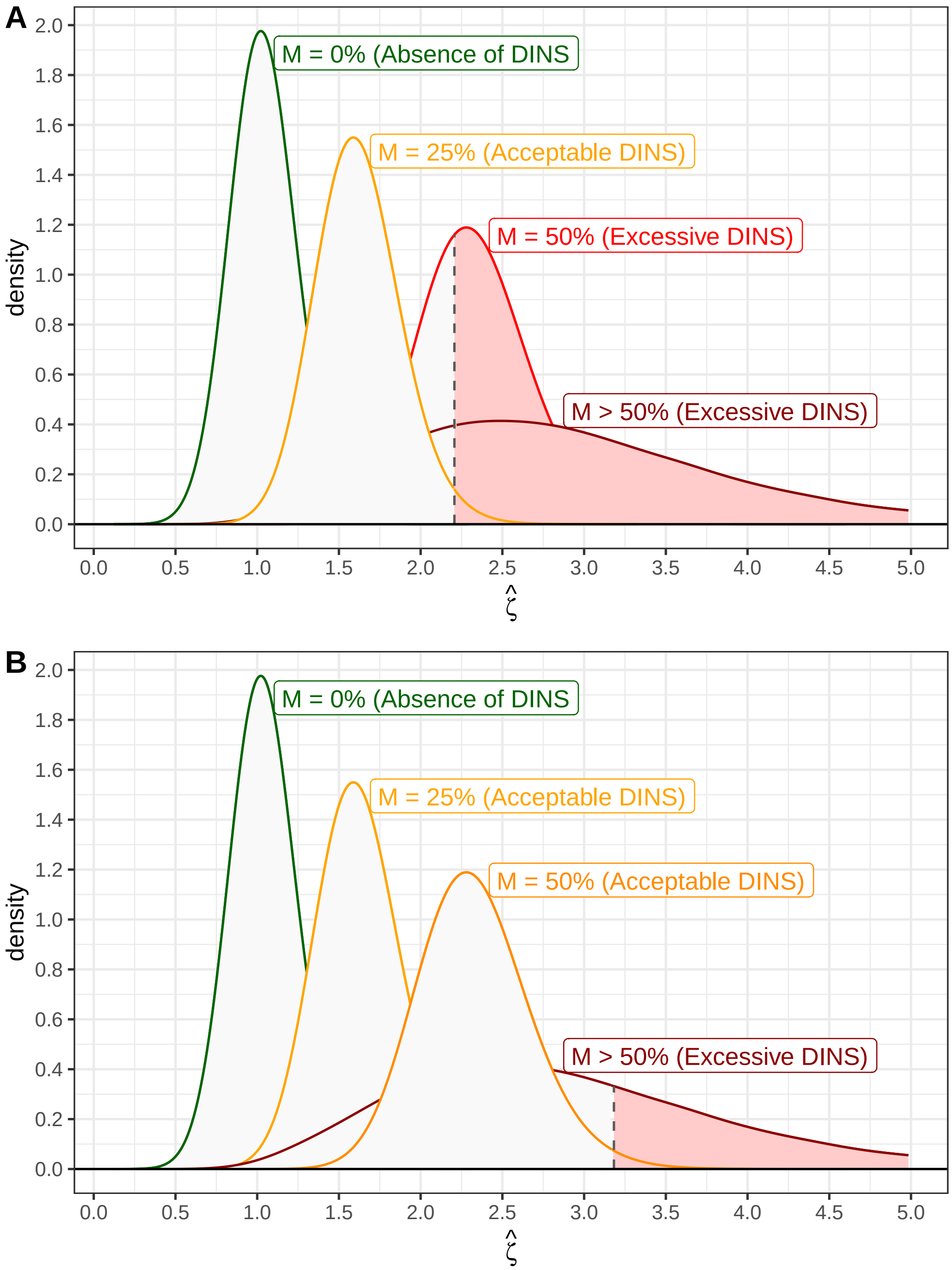

Figure 2. The statistical methodology for detecting excessive differences in non-selectivity (DINS) relies on the observed value of $\hat{\zeta}$ and a predetermined acceptable average percentage increase, $M(\%)$, in pointwise prediction interval widths attributable to DINS. Upon defining a suitable value for $M(\%)$, the subsequent step involves exploring the distribution of the conditional random variable $\hat{\zeta}$ given $M(\%)$, which inherently depends on the number of CSs and replicates. In these two examples, we have 25 clinical samples and three replicates. Excessive DINS is inferred if the observed $\hat{\zeta}$ surpasses the 99th percentile of this conditional random variable. Panel **A** illustrates a scenario with $M(\%)$ set at 25%, where the rejection region for $\hat{\zeta}$ (shown in red) extends from the 99th percentile of $\hat{\zeta}$ given $M(\%) = 25\%$. Conversely, Panel **B** portrays an alternative scenario with $M(\%)$ chosen as 50%, and the corresponding rejection region for $\hat{\zeta}$ (also in red) starts from the 99th percentile of $\hat{\zeta}$ given $M(\%) = 50\%$. The red shaded areas also illustrate the probability of falsely concluding with excessive DINS for the three other distributions.

0.3 Figure 3

Reproducing Figure 3 in the manuscript.

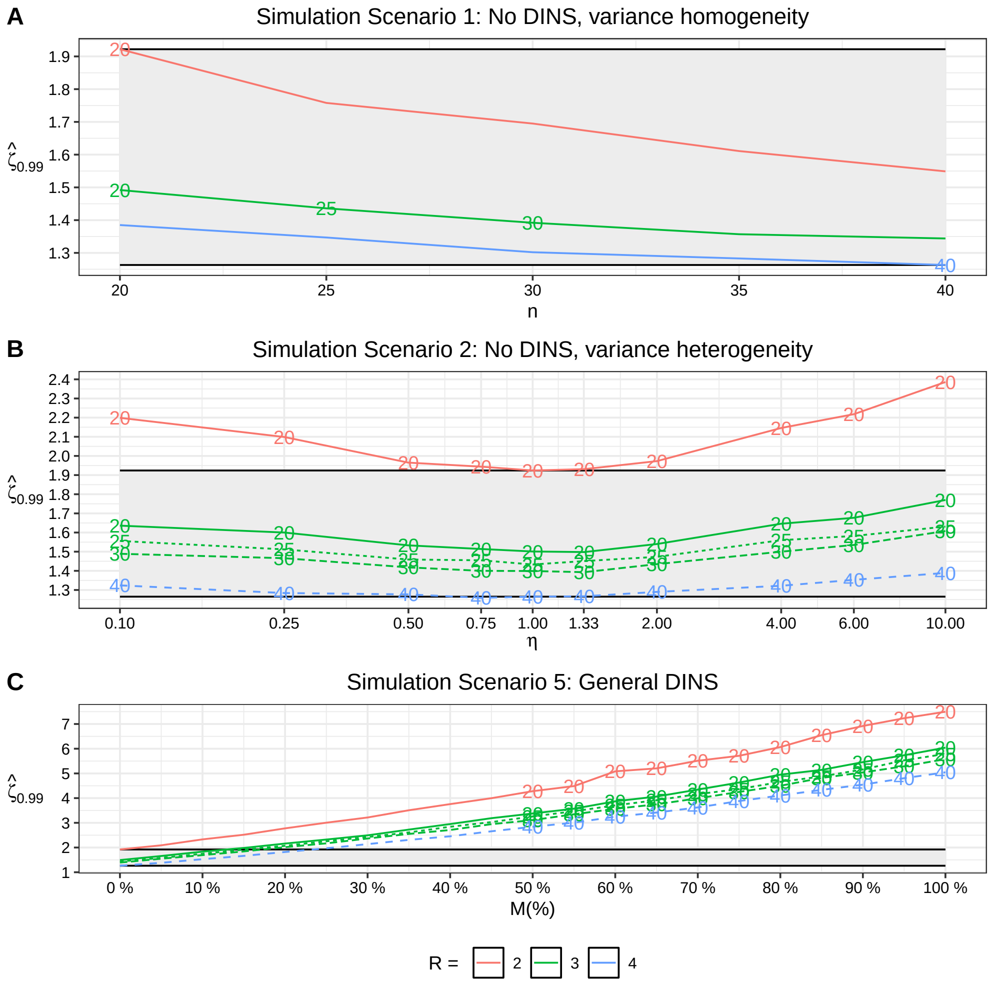

Figure 3. The 99% percentiles of $\hat{\zeta}$ ($\hat{\zeta}_{0.99}$) against the simulation parameters for simulation scenarios 1, 2, and 5. Panel **A** illustrates $\hat{\zeta}_{0.99}$ across five study designs vs. the number of clinical samples, n. Panel **B** $\hat{\zeta}_{0.99}$ is depicted as a function of heteroscedasticity factors, η, ranging from 0.1 to 10, for the same quintet of study designs. Panel **C** shows $\hat{\zeta}_{0.99}$ in conjuction with the average percentage increase in pointwise prediction interval widths attributable to DINS, $M(\%)$, again for the identical five study designs. Gray ribbons in the plots visually represent the range between the smallest $\hat{\zeta}_{0.99}$, associated with the largest study design configured with 40 clinical samples and four replicates, and the largest $\hat{\zeta}_{0.99}$, associated with the smallest study design with 20 clinical samples and two replicates, under the conditions of zero DINS and homoscedastic measurement errors. The numbers within the plots indicate the number of clinical samples.

1 Figure 4

Reproducing Figure 4 in the manuscript.

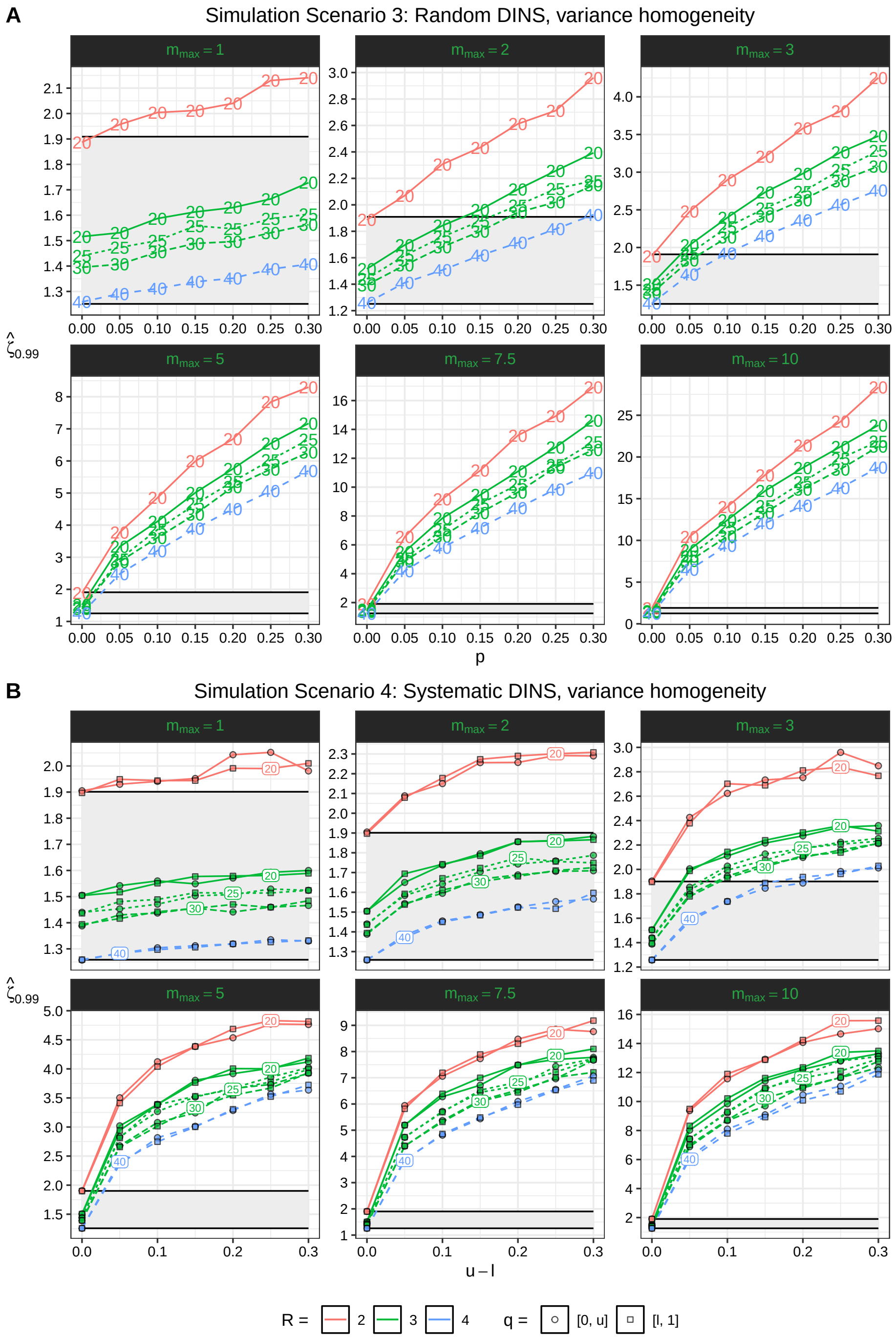

Figure 4. Demonstrations of the relationship between the 99% percentiles of $\hat{\zeta}$, $\hat{\zeta}_{0.99}$, and the simulation parameters of simulation scenarios 3 and 4 for a pertient subset of study designs. **A** illustrates the relationship between $\hat{\zeta}_{0.99}$ and the average proporton of random DINS affected clinical samples, p, in conjuction with the maximum relocation multiplier, $m_{\max}$. **B** portrays the relationship between the $\hat{\zeta}_{0.99}$ and the quantile interval length, $u - l$, where systematic DINS has its effect, in combination with $m_{\max}$. Gray ribbons in the plots visually represent the range between the smallest $\hat{\zeta}_{0.99}$, associated with the largest study design configured with 40 clinical samples and four replicates, and the largest $\hat{\zeta}_{0.99}$, associated with the smallest study design with 20 clinical samples and two replicates, under the conditions of zero DINS and homoscedastic measurement errors. The numbers within the plots indicate the number of clinical samples.

1.1 Table 1

Reproducing Table 1 in the manuscript

Table 1. Monte Carlo simulations of the 99th percentile of $\hat{\zeta}$ across the five simulation scenarios and a pertinent subset of study designs. The parameter $\hat{\zeta}_{0.99}$ represents the 99th percentile of $\hat{\zeta}$. Other parameters in the table are defined as in the **Simulation scenarios for the investigation of** $\hat{\zeta}$ section.

n	R	Simulation scenario	η	$m_{\max}$	p	$u-l$	$M(\%)$	$\hat{\zeta}_{0.99}$
20	2	1	1.0	0	0.00	0.00	0 %	1.922
20	2	2	0.1	0	0.00	0.00	0 %	2.199
20	2	2	10.0	0	0.00	0.00	0 %	2.386
20	2	3	1.0	5	0.05	0.00	> 0 %	3.774
20	2	3	1.0	10	0.05	0.00	> 0 %	10.461
20	2	3	1.0	5	0.30	0.00	> 0 %	8.298
20	2	3	1.0	10	0.30	0.00	> 0 %	28.365
20	2	4	1.0	5	0.00	0.05	> 0 %	3.458
20	2	4	1.0	10	0.00	0.05	> 0 %	9.429
20	2	4	1.0	5	0.00	0.30	> 0 %	4.789
20	2	4	1.0	10	0.00	0.30	> 0 %	15.298
20	2	5	1.0	0	0.00	0.00	25 %	3.005
20	2	5	1.0	0	0.00	0.00	50 %	4.281
20	2	5	1.0	0	0.00	0.00	75 %	5.713
20	2	5	1.0	0	0.00	0.00	100 %	7.497
20	3	1	1.0	0	0.00	0.00	0 %	1.492
20	3	2	0.1	0	0.00	0.00	0 %	1.636
20	3	2	10.0	0	0.00	0.00	0 %	1.770
20	3	3	1.0	5	0.05	0.00	> 0 %	3.329
20	3	3	1.0	10	0.05	0.00	> 0 %	8.877
20	3	3	1.0	5	0.30	0.00	> 0 %	7.169
20	3	3	1.0	10	0.30	0.00	> 0 %	23.809
20	3	4	1.0	5	0.00	0.05	> 0 %	2.981
20	3	4	1.0	10	0.00	0.05	> 0 %	8.174
20	3	4	1.0	5	0.00	0.30	> 0 %	4.153
20	3	4	1.0	10	0.00	0.30	> 0 %	13.391
20	3	5	1.0	0	0.00	0.00	25 %	2.324
20	3	5	1.0	0	0.00	0.00	50 %	3.378
20	3	5	1.0	0	0.00	0.00	75 %	4.631
20	3	5	1.0	0	0.00	0.00	100 %	6.043
25	3	1	1.0	0	0.00	0.00	0 %	1.436
25	3	2	0.1	0	0.00	0.00	0 %	1.556
25	3	2	10.0	0	0.00	0.00	0 %	1.631
25	3	3	1.0	5	0.05	0.00	> 0 %	2.946
25	3	3	1.0	10	0.05	0.00	> 0 %	8.085
25	3	3	1.0	5	0.30	0.00	> 0 %	6.668
25	3	3	1.0	10	0.30	0.00	> 0 %	21.886
25	3	4	1.0	5	0.00	0.05	> 0 %	2.821
25	3	4	1.0	10	0.00	0.05	> 0 %	7.423
25	3	4	1.0	5	0.00	0.30	> 0 %	4.007
25	3	4	1.0	10	0.00	0.30	> 0 %	13.025
25	3	5	1.0	0	0.00	0.00	25 %	2.253
25	3	5	1.0	0	0.00	0.00	50 %	3.258
25	3	5	1.0	0	0.00	0.00	75 %	4.363
25	3	5	1.0	0	0.00	0.00	100 %	5.789

30	3	1	1.0	0	0.00	0.00	0 %	1.392
30	3	2	0.1	0	0.00	0.00	0 %	1.489
30	3	2	10.0	0	0.00	0.00	0 %	1.608
30	3	3	1.0	5	0.05	0.00	>0 %	2.863
30	3	3	1.0	10	0.05	0.00	>0 %	7.539
30	3	3	1.0	5	0.30	0.00	>0 %	6.268
30	3	3	1.0	10	0.30	0.00	>0 %	21.250
30	3	4	1.0	5	0.00	0.05	>0 %	2.667
30	3	4	1.0	10	0.00	0.05	>0 %	6.950
30	3	4	1.0	5	0.00	0.30	>0 %	3.928
30	3	4	1.0	10	0.00	0.30	>0 %	12.553
30	3	5	1.0	0	0.00	0.00	25 %	2.169
30	3	5	1.0	0	0.00	0.00	50 %	3.111
30	3	5	1.0	0	0.00	0.00	75 %	4.251
30	3	5	1.0	0	0.00	0.00	100 %	5.577
40	4	1	1.0	0	0.00	0.00	0 %	1.263
40	4	2	0.1	0	0.00	0.00	0 %	1.324
40	4	2	10.0	0	0.00	0.00	0 %	1.389
40	4	3	1.0	5	0.05	0.00	>0 %	2.482
40	4	3	1.0	10	0.05	0.00	>0 %	6.493
40	4	3	1.0	5	0.30	0.00	>0 %	5.692
40	4	3	1.0	10	0.30	0.00	>0 %	18.744
40	4	4	1.0	5	0.00	0.05	>0 %	2.380
40	4	4	1.0	10	0.00	0.05	>0 %	6.098
40	4	4	1.0	5	0.00	0.30	>0 %	3.680
40	4	4	1.0	10	0.00	0.30	>0 %	12.020
40	4	5	1.0	0	0.00	0.00	25 %	1.969
40	4	5	1.0	0	0.00	0.00	50 %	2.840
40	4	5	1.0	0	0.00	0.00	75 %	3.881
40	4	5	1.0	0	0.00	0.00	100 %	5.045

1.2 Table 2

Reproducing Table 2 in the manuscript

Table 2. Information on the clinical datasets to be evaluated regarding differences in non-selectivity. **BPCI** is short for bootstrap percentile confidence interval. Note that **lower** refers to the lower part of the 95% BPCI confidence intervals, whereas **upper** refers to the upper part.

Variable information			Summary statistics for measurements						IVD-MD imprecision estimates with 95% BPCI					
**Variable**	**Type**	**Max. decimal places**	**Mean**	**Q1**	**Median**	**Q3**	**Min.**	**Max.**	**SD**	$\mathbf{SD_{lower}}$	$\mathbf{SD_{upper}}$	**CV(%)**	$\mathbf{CV_{lower}}$(%)	$\mathbf{CV_{upper}}$(%)
**Glucose, Serum (unit: mmol/L)**														
SampleID	identifier						1.00	25.00						
ReplicateID	identifier						1.00	3.00						
Advia	numeric	2	7.875	5.520	7.55	9.850	4.22	12.86	0.048	0.036	0.062	0.615	0.454	0.807
Alinity	numeric	2	7.940	5.550	7.57	9.940	4.19	13.28	0.040	0.032	0.048	0.507	0.436	0.572
Cholestech	numeric	2	7.647	5.360	7.52	9.390	4.04	12.60	0.184	0.137	0.228	2.401	1.903	2.815
Cobas	numeric	2	7.955	5.550	7.63	9.980	4.22	13.14	0.072	0.050	0.092	0.905	0.684	1.086
Vitros	numeric	2	8.024	5.660	7.68	9.990	4.28	13.13	0.035	0.027	0.042	0.430	0.340	0.520
**Hemoglobin (unit: g/dL)**														
SampleID	identifier						2.00	25.00						
ReplicateID	identifier						1.00	3.00						
Dia Spect Tm	numeric	1	12.035	10.175	13.00	13.325	8.80	15.80	0.059	0.047	0.070	0.490	0.401	0.569
HemoCue Hb201	numeric	1	12.149	10.225	12.90	13.650	8.60	16.00	0.062	0.051	0.073	0.513	0.419	0.607
HemoCue Hb801	numeric	1	12.335	10.650	13.15	13.700	8.90	16.40	0.070	0.051	0.087	0.565	0.411	0.717
QR go	numeric	1	12.418	10.275	13.35	14.075	8.40	16.70	0.242	0.199	0.281	1.945	1.573	2.314
**C-reactive protein (unit: mg/L)**														
SampleID	identifier						1.00	25.00						
ReplicateID	identifier						1.00	3.00						
Advia	numeric	1	38.081	14.900	38.60	53.600	4.50	84.40	0.326	0.216	0.429	0.856	0.647	1.045
Architect	numeric	1	36.468	12.800	36.50	52.500	4.60	82.40	0.380	0.296	0.457	1.041	0.776	1.381
Cobas	numeric	1	32.777	11.100	32.40	48.100	4.40	77.10	0.669	0.489	0.823	2.042	1.689	2.413
Dimension	numeric	2	37.096	12.500	35.10	53.400	5.20	86.40	0.698	0.551	0.859	1.880	1.367	2.607
Vitros	numeric	1	34.655	13.600	33.90	47.500	4.20	79.60	0.658	0.494	0.809	1.899	1.546	2.262

1.3 Table 3

Reproducing Table 3 in the manuscript:

Table 3. Descriptive statistics and bootstrap confidence intervals of $\hat{\zeta}$ for glucose, hemoglobin (HB), and C-reactive protein (CRP) analytes. The descriptive statistics and confidence intervals of $\hat{\zeta}$ are estimated using the cluster-bootstrap resampling algorithm for each comparison involving the analytes glucose, HB, and CRP. The abbreviation **BPCI** stands for bootstrap percentile confidence interval.

IVD-MD comparison	Point estimate	95% BPCI		Bootstrap summary statistics of $\hat{\zeta}$									
**Comparison**	$\hat{\zeta}$	$\hat{\zeta}_{\text{lower}}$	$\hat{\zeta}_{\text{upper}}$	**Mean**	**SD**	**Skewness**	**Kurtosis**	**MAD**	**Q1**	**Median**	**Q3**	**Min.**	**Max.**
**Glucose, Serum (unit: mg/L)**													
Advia - Alinity	1.205	0.770	1.503	1.124	0.190	0.117	2.894	0.196	0.989	1.127	1.253	0.573	1.898
Advia - Cholestech	1.133	0.827	1.661	1.135	0.218	1.225	5.586	0.191	0.982	1.096	1.247	0.683	2.469
Advia - Cobas	0.770	0.575	1.035	0.774	0.118	0.559	3.692	0.113	0.691	0.763	0.845	0.415	1.400
Advia - Vitros	1.902	1.165	2.728	1.828	0.400	0.562	3.413	0.395	1.539	1.786	2.079	0.627	3.658
Alinity - Cholestech	1.148	0.858	1.538	1.134	0.174	0.779	4.006	0.163	1.011	1.110	1.234	0.703	2.040
Alinity - Cobas	0.872	0.461	1.462	0.846	0.256	1.112	5.599	0.229	0.665	0.807	0.980	0.304	2.982
Alinity - Vitros	2.836	1.775	3.616	2.670	0.467	0.085	3.161	0.467	2.350	2.659	2.980	1.017	4.306
Cholestech - Cobas	1.193	0.867	1.659	1.195	0.201	0.747	4.029	0.187	1.054	1.170	1.313	0.705	2.261
Cholestech - Vitros	1.388	0.991	1.930	1.357	0.242	1.019	4.920	0.223	1.186	1.319	1.494	0.785	2.835
Cobas - Vitros	1.239	0.974	1.482	1.206	0.126	0.322	4.488	0.112	1.124	1.196	1.276	0.634	1.862
**Hemoglobine (unit: g/dl)**													
Dia Spect Tm - HemoCue Hb201	5.687	2.748	8.590	5.323	1.504	0.493	3.363	1.491	4.240	5.209	6.278	1.406	11.993
Dia Spect Tm - HemoCue Hb801	3.655	2.071	5.371	3.464	0.851	0.640	3.640	0.832	2.851	3.372	3.978	1.218	7.729
Dia Spect Tm - QR go	1.113	0.853	1.424	1.094	0.146	0.640	3.743	0.136	0.993	1.078	1.177	0.677	1.804
HemoCue Hb201 - HemoCue Hb801	6.110	2.868	9.095	5.660	1.594	0.498	3.617	1.526	4.546	5.542	6.613	1.209	14.520
HemoCue Hb201 - QR go	1.553	1.154	1.893	1.497	0.186	0.334	3.242	0.185	1.368	1.487	1.616	0.964	2.256
HemoCue Hb801 - QR go	1.833	1.194	2.607	1.775	0.367	0.861	4.282	0.342	1.510	1.725	1.981	0.933	3.836
**C-reactive protein (unit: mg/L)**													
Advia - Architect	3.609	1.680	6.228	3.489	1.152	0.923	4.629	1.061	2.672	3.319	4.133	1.000	11.512
Advia - Cobas	4.044	2.131	6.015	3.788	0.999	0.944	5.702	0.881	3.130	3.689	4.322	1.158	10.540
Advia - Dimension	8.141	4.029	13.837	7.935	2.512	0.859	4.163	2.346	6.137	7.605	9.360	1.790	21.177
Advia - Vitros	2.547	1.757	3.179	2.420	0.355	0.290	4.016	0.326	2.190	2.404	2.631	0.773	4.251
Architect - Cobas	2.500	1.064	3.258	2.293	0.516	-0.259	3.479	0.483	1.981	2.308	2.632	0.552	4.124
Architect - Dimension	3.935	2.164	5.883	3.772	0.940	0.569	3.605	0.909	3.113	3.691	4.343	1.245	8.147
Architect - Vitros	2.459	1.677	3.156	2.346	0.370	0.440	3.558	0.356	2.093	2.321	2.576	1.133	4.579
Cobas - Dimension	3.742	1.836	4.984	3.456	0.799	-0.094	2.869	0.785	2.932	3.488	3.997	1.073	6.580
Cobas - Vitros	3.374	1.796	5.151	3.173	0.877	0.684	3.530	0.893	2.513	3.068	3.721	1.221	7.675
Dimension - Vitros	3.225	1.687	5.329	3.084	0.940	0.940	4.391	0.878	2.394	2.951	3.620	1.252	9.056

Reproducing the text that interprets Table 3, utilizing variables in the script chunk.

The estimated moments for $\hat{\zeta}$ across glucose, HB, and CRP datasets exhibit the following ranges. In the glucose dataset, the means range from 0.774 to 2.67, variances range from 0.014 to 0.218, skewness values fall between 0.085 and 1.225, and kurtosis values fall between 2.894 and 5.599. For the HB dataset, the means span from 1.094 to 5.66, variances span from 0.021 to 2.541, skewness values fall between 0.334 and 0.861, and kurtosis values fall between 3.242 and 4.282. Lastly, in the CRP dataset, the means lie between 2.293 and 7.935, variances lie between 0.126 and 6.311, skewness values fall between -0.259 and 0.944, and kurtosis values fall between 2.869 and 5.702.

1.4 Figure 5

Reproducing Figure 5 in the manuscript.

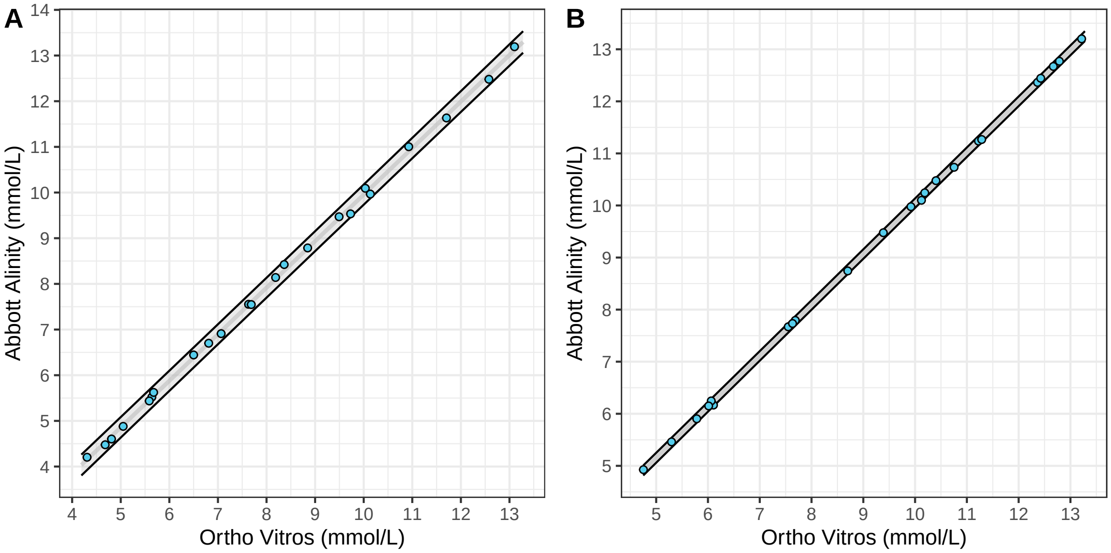

Figure 5. Scatter plots of glucose measurements results of the Abbott Alinity versus Ortho Vitros comparison. **A**: Scatter plot illustrating the original pairs of glucose measurements of Abbott Alinity and Ortho Vitros, featuring the 99% pointwise prediction intervals (shaded gray area) derived from Fuller and Gillards' (F-G) Deming regression model. For the original data, we have that $\hat{\zeta} = 2.836$. **B**: Scatter plot showcasing simulated pairs of glucose measurement results for the Abbott Alinity and Ortho Vitros comparison (with DINS removed), accompanied by the 99% pointwise prediction intervals (shaded gray area) based on the F-G Deming regression model. For the simulated data, we have that $\hat{\zeta} = 1.013$.

1.5 Figure 6

Reproducing Figure 6 in the manuscript (Appendix B).

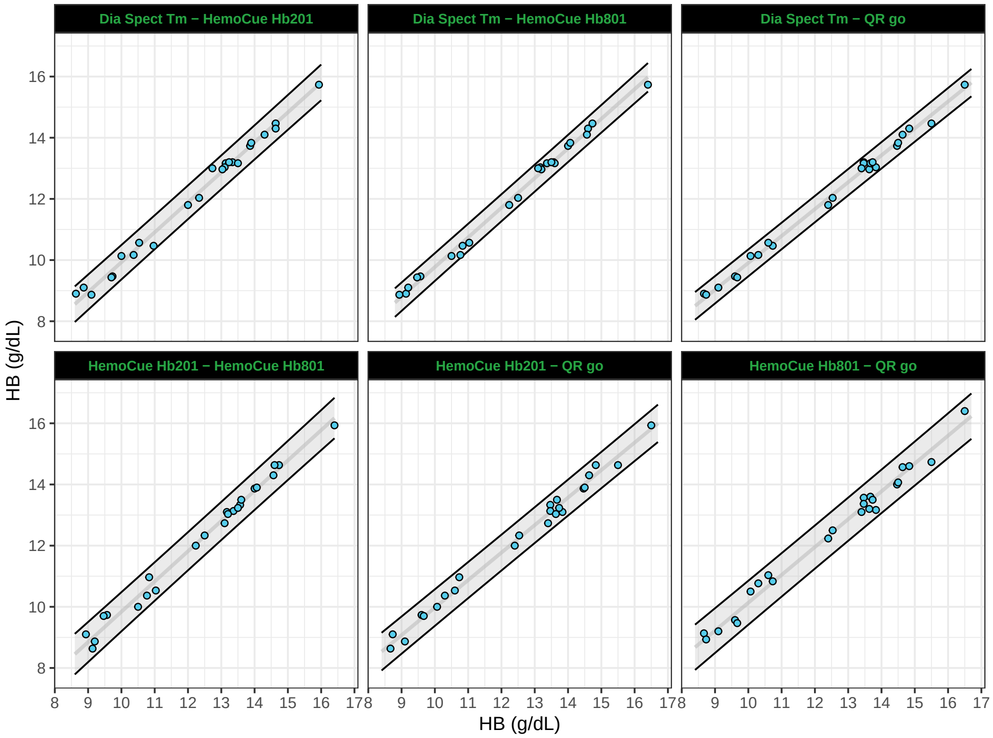

Figure 6. Scatter plots of the six IVD-MD comparisons for the HB dataset. The pointwise prediction intervals, depicted as gray regions, are estimated using the F-G approach.

1.6 Figure 7

Reproducing Figure 7 in the manuscript (Appendix B).

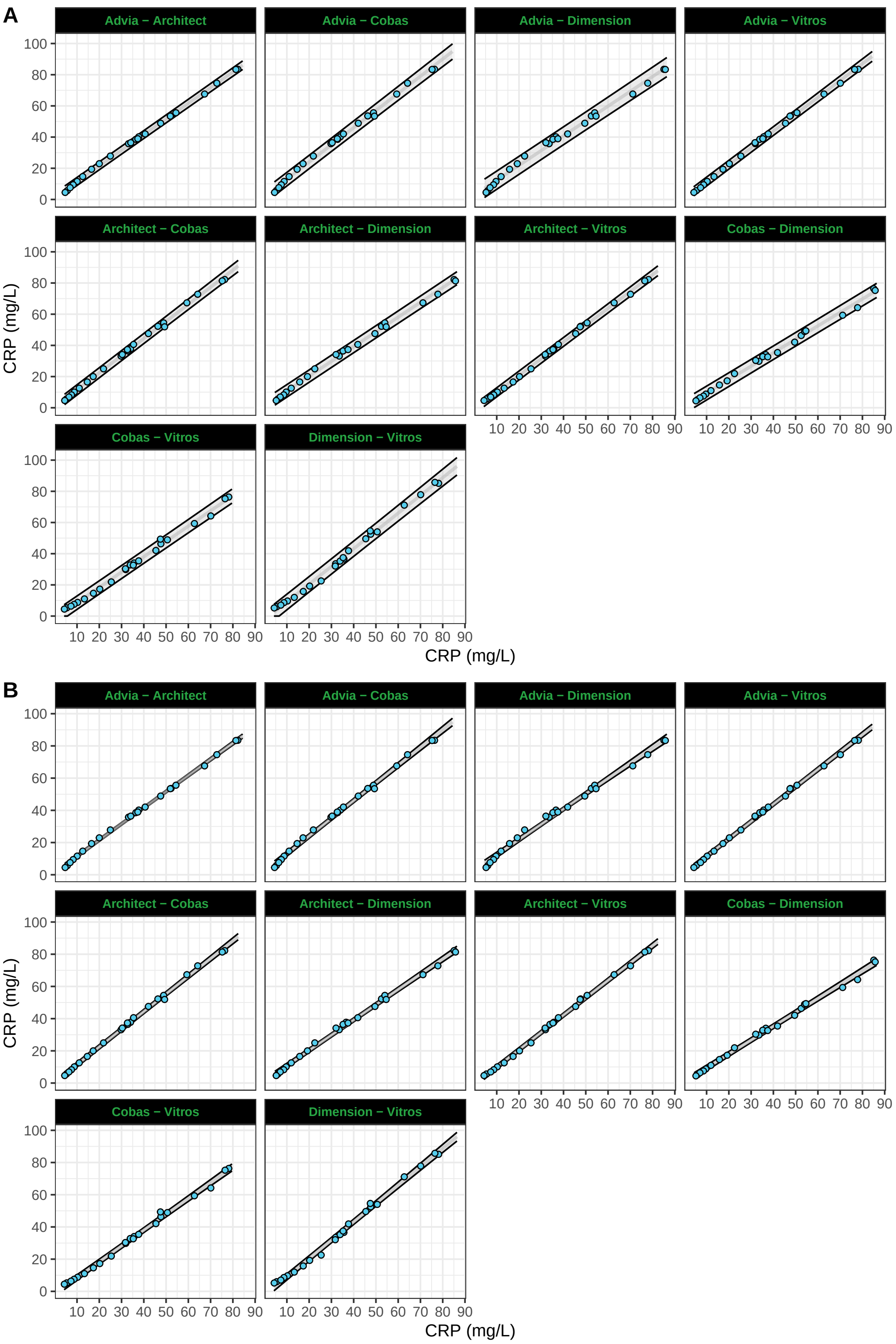

Figure 7. **A**: Scatter plots of the ten IVD-MD comparisons for the CRP dataset. The pointwise prediction intervals, indicated by the gray areas, are estimated via the F-G approach. **B**: Scatter plots of the ten IVD-MD comparisons for the CRP dataset, similar to **A**, but with pointwise prediction intervals estimated through the CLSI approach.

1.7 Table 4

Reproducing Table 4 in the manuscript (Appendix B).

Table 4. Look-up table showcasing pre-simulated values of $[\hat{\zeta}|M(\%)]_{0.99}$ based on the most typical study designs and upper bounds of acceptable differences in non-selectivity, $M(\%)$.

CSs	M(%)																				
**n**	**0%**	**5%**	**10%**	**15%**	**20%**	**25%**	**30%**	**35%**	**40%**	**45%**	**50%**	**55%**	**60%**	**65%**	**70%**	**75%**	**80%**	**85%**	**90%**	**95%**	**100%**
**R = 2 replicates**																					
20	1.901	2.101	2.307	2.518	2.748	2.975	3.224	3.472	3.735	4.004	4.286	4.578	4.871	5.187	5.506	5.847	6.172	6.517	6.878	7.241	7.619
21	1.872	2.066	2.268	2.479	2.693	2.923	3.166	3.408	3.664	3.928	4.209	4.496	4.797	5.087	5.412	5.732	6.070	6.407	6.750	7.117	7.465
22	1.840	2.029	2.230	2.434	2.647	2.877	3.110	3.358	3.615	3.863	4.141	4.422	4.718	5.012	5.316	5.645	5.969	6.304	6.638	7.004	7.351
23	1.816	1.998	2.196	2.402	2.615	2.834	3.064	3.301	3.561	3.817	4.083	4.355	4.641	4.928	5.229	5.555	5.880	6.195	6.544	6.893	7.246
24	1.787	1.969	2.162	2.363	2.576	2.796	3.013	3.259	3.498	3.753	4.022	4.293	4.576	4.852	5.168	5.468	5.793	6.115	6.458	6.796	7.145
25	1.765	1.947	2.137	2.331	2.535	2.759	2.978	3.211	3.460	3.701	3.971	4.238	4.511	4.799	5.092	5.393	5.719	6.043	6.365	6.698	7.053
26	1.743	1.921	2.111	2.304	2.507	2.725	2.944	3.179	3.424	3.658	3.921	4.181	4.459	4.737	5.028	5.326	5.642	5.964	6.287	6.619	6.972
27	1.724	1.898	2.082	2.280	2.481	2.691	2.912	3.137	3.380	3.611	3.869	4.139	4.408	4.695	4.986	5.275	5.578	5.886	6.223	6.547	6.886
28	1.703	1.878	2.063	2.250	2.454	2.661	2.878	3.100	3.339	3.580	3.829	4.098	4.356	4.634	4.924	5.210	5.519	5.828	6.152	6.480	6.814
29	1.688	1.858	2.038	2.231	2.428	2.634	2.845	3.072	3.300	3.543	3.790	4.049	4.311	4.579	4.871	5.166	5.458	5.767	6.080	6.414	6.747
30	1.670	1.839	2.021	2.207	2.403	2.606	2.825	3.040	3.274	3.510	3.753	4.008	4.271	4.552	4.825	5.108	5.399	5.713	6.020	6.344	6.663
32	1.639	1.807	1.986	2.169	2.364	2.561	2.772	2.989	3.212	3.445	3.689	3.943	4.199	4.462	4.745	5.018	5.305	5.611	5.929	6.233	6.564
34	1.612	1.778	1.952	2.134	2.321	2.522	2.725	2.941	3.163	3.387	3.633	3.875	4.133	4.391	4.662	4.944	5.220	5.522	5.817	6.131	6.459
35	1.599	1.766	1.940	2.117	2.303	2.501	2.707	2.915	3.140	3.369	3.602	3.846	4.100	4.361	4.628	4.900	5.187	5.481	5.772	6.091	6.398
36	1.589	1.752	1.922	2.103	2.290	2.480	2.686	2.899	3.113	3.338	3.581	3.821	4.067	4.326	4.590	4.869	5.149	5.439	5.739	6.048	6.360
38	1.569	1.731	1.896	2.075	2.255	2.447	2.651	2.860	3.071	3.300	3.525	3.766	4.013	4.267	4.533	4.806	5.092	5.369	5.654	5.963	6.273
40	1.549	1.709	1.874	2.048	2.233	2.422	2.615	2.820	3.033	3.257	3.488	3.717	3.966	4.210	4.481	4.742	5.023	5.300	5.591	5.887	6.194
45	1.507	1.662	1.824	1.995	2.172	2.357	2.546	2.745	2.954	3.172	3.392	3.623	3.856	4.106	4.363	4.615	4.885	5.157	5.442	5.730	6.039
50	1.475	1.625	1.785	1.950	2.123	2.305	2.492	2.689	2.888	3.097	3.317	3.543	3.775	4.015	4.261	4.515	4.779	5.046	5.325	5.609	5.898
**R = 3 replicates**																					
20	1.507	1.660	1.822	1.992	2.171	2.356	2.547	2.749	2.953	3.170	3.387	3.620	3.857	4.098	4.353	4.614	4.880	5.151	5.435	5.723	6.027
21	1.491	1.643	1.804	1.970	2.145	2.333	2.522	2.717	2.922	3.137	3.359	3.581	3.820	4.060	4.312	4.573	4.833	5.102	5.384	5.671	5.963
22	1.477	1.628	1.786	1.957	2.128	2.308	2.496	2.692	2.892	3.108	3.322	3.548	3.783	4.022	4.263	4.521	4.786	5.055	5.323	5.617	5.911
23	1.463	1.613	1.772	1.935	2.108	2.286	2.473	2.668	2.867	3.074	3.295	3.514	3.747	3.982	4.231	4.480	4.741	5.012	5.287	5.570	5.854
24	1.452	1.601	1.755	1.918	2.090	2.268	2.452	2.644	2.842	3.055	3.262	3.490	3.715	3.949	4.193	4.445	4.701	4.970	5.240	5.518	5.810
25	1.440	1.588	1.742	1.906	2.073	2.251	2.434	2.624	2.820	3.028	3.240	3.459	3.685	3.921	4.164	4.410	4.664	4.929	5.196	5.476	5.760
26	1.429	1.576	1.728	1.891	2.059	2.235	2.416	2.605	2.803	3.007	3.217	3.437	3.657	3.896	4.132	4.378	4.631	4.896	5.163	5.435	5.716
27	1.421	1.566	1.717	1.877	2.044	2.219	2.397	2.588	2.782	2.985	3.196	3.412	3.635	3.871	4.105	4.348	4.602	4.859	5.123	5.402	5.679
28	1.411	1.555	1.708	1.865	2.032	2.203	2.385	2.571	2.763	2.964	3.175	3.391	3.607	3.845	4.074	4.324	4.572	4.830	5.097	5.360	5.643
29	1.402	1.545	1.696	1.855	2.018	2.190	2.370	2.555	2.750	2.948	3.156	3.368	3.592	3.818	4.054	4.294	4.544	4.798	5.061	5.327	5.609
30	1.394	1.538	1.686	1.842	2.008	2.177	2.357	2.543	2.733	2.932	3.136	3.351	3.571	3.795	4.030	4.269	4.518	4.773	5.030	5.302	5.577
32	1.380	1.521	1.671	1.825	1.986	2.156	2.331	2.513	2.704	2.900	3.104	3.316	3.530	3.754	3.987	4.224	4.470	4.722	4.977	5.247	5.516
34	1.366	1.506	1.653	1.804	1.967	2.135	2.307	2.490	2.680	2.872	3.074	3.282	3.499	3.719	3.949	4.182	4.425	4.675	4.930	5.191	5.463
35	1.359	1.499	1.646	1.796	1.957	2.126	2.299	2.479	2.665	2.858	3.060	3.270	3.481	3.704	3.928	4.163	4.404	4.654	4.905	5.178	5.440
36	1.354	1.494	1.641	1.790	1.949	2.118	2.288	2.468	2.654	2.847	3.047	3.252	3.468	3.686	3.911	4.149	4.387	4.632	4.887	5.151	5.417
38	1.343	1.481	1.626	1.776	1.934	2.099	2.271	2.448	2.633	2.827	3.024	3.230	3.440	3.655	3.881	4.114	4.355	4.597	4.848	5.113	5.367
40	1.333	1.470	1.614	1.765	1.921	2.083	2.253	2.429	2.616	2.804	3.002	3.202	3.413	3.629	3.855	4.086	4.323	4.564	4.815	5.074	5.336
45	1.313	1.447	1.589	1.735	1.890	2.049	2.219	2.391	2.571	2.758	2.954	3.154	3.361	3.571	3.792	4.020	4.251	4.494	4.740	4.986	5.251
50	1.295	1.428	1.568	1.713	1.866	2.025	2.190	2.361	2.539	2.723	2.915	3.112	3.316	3.529	3.742	3.965	4.196	4.435	4.677	4.926	5.176
**R = 4 replicates**																					
20	1.378	1.519	1.668	1.822	1.983	2.152	2.329	2.508	2.700	2.896	3.097	3.311	3.530	3.753	3.979	4.222	4.466	4.715	4.973	5.243	5.509
21	1.367	1.508	1.655	1.807	1.969	2.136	2.311	2.492	2.679	2.873	3.076	3.282	3.501	3.723	3.952	4.187	4.433	4.677	4.938	5.196	5.471
22	1.358	1.497	1.643	1.795	1.955	2.120	2.295	2.474	2.661	2.855	3.053	3.261	3.476	3.697	3.921	4.158	4.403	4.646	4.901	5.162	5.427
23	1.348	1.487	1.632	1.784	1.942	2.107	2.279	2.459	2.646	2.837	3.035	3.240	3.456	3.672	3.899	4.131	4.372	4.617	4.865	5.130	5.395
24	1.340	1.478	1.621	1.774	1.931	2.094	2.265	2.442	2.627	2.821	3.018	3.222	3.437	3.650	3.877	4.107	4.344	4.588	4.836	5.100	5.362
25	1.333	1.470	1.613	1.764	1.920	2.082	2.252	2.431	2.612	2.802	2.999	3.200	3.413	3.629	3.855	4.078	4.314	4.561	4.810	5.071	5.329
26	1.326	1.461	1.604	1.753	1.908	2.072	2.239	2.417	2.598	2.784	2.984	3.187	3.393	3.609	3.832	4.062	4.298	4.537	4.784	5.040	5.304
27	1.320	1.455	1.596	1.745	1.900	2.062	2.229	2.402	2.587	2.773	2.970	3.170	3.378	3.591	3.816	4.039	4.272	4.512	4.761	5.019	5.276
28	1.313	1.447	1.588	1.737	1.890	2.052	2.218	2.394	2.572	2.758	2.956	3.156	3.362	3.574	3.795	4.021	4.250	4.493	4.744	4.993	5.252
29	1.307	1.440	1.581	1.729	1.882	2.043	2.211	2.381	2.562	2.750	2.941	3.140	3.347	3.561	3.778	4.003	4.238	4.476	4.716	4.970	5.232
30	1.302	1.434	1.575	1.721	1.874	2.033	2.200	2.373	2.553	2.736	2.928	3.127	3.330	3.543	3.762	3.987	4.216	4.454	4.696	4.948	5.205
32	1.291	1.423	1.563	1.709	1.860	2.017	2.182	2.354	2.532	2.719	2.906	3.103	3.306	3.516	3.731	3.958	4.179	4.420	4.663	4.912	5.167
34	1.282	1.414	1.553	1.696	1.848	2.004	2.168	2.338	2.513	2.696	2.885	3.082	3.284	3.490	3.706	3.928	4.155	4.390	4.631	4.880	5.129
35	1.278	1.411	1.547	1.690	1.841	1.998	2.160	2.329	2.505	2.688	2.875	3.071	3.272	3.479	3.696	3.911	4.139	4.374	4.613	4.860	5.112
36	1.275	1.405	1.542	1.686	1.835	1.991	2.154	2.323	2.497	2.679	2.866	3.062	3.263	3.469	3.684	3.901	4.128	4.361	4.601	4.844	5.096
38	1.266	1.396	1.532	1.676	1.825	1.978	2.142	2.309	2.483	2.664	2.851	3.044	3.242	3.451	3.657	3.878	4.103	4.338	4.572	4.817	5.063
40	1.260	1.389	1.524	1.667	1.814	1.971	2.129	2.296	2.471	2.649	2.836	3.027	3.227	3.432	3.640	3.862	4.083	4.314	4.548	4.790	5.042
45	1.246	1.373	1.507	1.649	1.793	1.946	2.105	2.270	2.441	2.619	2.803	2.992	3.189	3.391	3.603	3.813	4.037	4.259	4.494	4.738	4.979
50	1.234	1.360	1.492	1.633	1.777	1.927	2.085	2.249	2.419	2.595	2.777	2.963	3.158	3.358	3.564	3.778	3.996	4.222	4.458	4.693	4.936
**R = 5 replicates**																					
20	1.314	1.448	1.590	1.738	1.893	2.053	2.222	2.393	2.574	2.763	2.957	3.155	3.363	3.576	3.798	4.023	4.255	4.497	4.744	4.994	5.253
21	1.306	1.440	1.582	1.727	1.880	2.040	2.208	2.382	2.560	2.745	2.939	3.136	3.342	3.554	3.774	3.999	4.228	4.469	4.716	4.966	5.220
22	1.299	1.431	1.571	1.718	1.870	2.029	2.195	2.365	2.544	2.729	2.923	3.119	3.322	3.534	3.752	3.979	4.206	4.446	4.692	4.940	5.194
23	1.291	1.424	1.564	1.708	1.859	2.019	2.182	2.353	2.531	2.715	2.908	3.102	3.308	3.515	3.735	3.953	4.184	4.422	4.663	4.909	5.166
24	1.286	1.417	1.555	1.698	1.851	2.008	2.174	2.342	2.518	2.704	2.891	3.086	3.291	3.499	3.715	3.936	4.163	4.398	4.644	4.887	5.140
25	1.279	1.411	1.548	1.691	1.842	1.998	2.162	2.332	2.506	2.690	2.879	3.073	3.277	3.480	3.697	3.919	4.147	4.379	4.619	4.866	5.119
26	1.274	1.404	1.541	1.684	1.835	1.992	2.154	2.321	2.496	2.679	2.864	3.060	3.260	3.466	3.680	3.900	4.130	4.361	4.597	4.847	5.094
27	1.268	1.400	1.535	1.677	1.827	1.983	2.143	2.312	2.486	2.666	2.855	3.047	3.246	3.453	3.668	3.886	4.111	4.342	4.581	4.824	5.073
28	1.264	1.394	1.528	1.671	1.820	1.975	2.135	2.304	2.477	2.659	2.844	3.036	3.237	3.441	3.652	3.870	4.097	4.325	4.565	4.807	5.054
29	1.259	1.388	1.523	1.665	1.814	1.968	2.129	2.296	2.470	2.649	2.834	3.027	3.225	3.428	3.640	3.857	4.081	4.308	4.547	4.786	5.038
30	1.254	1.384	1.519	1.660	1.807	1.961	2.120	2.286	2.461	2.640	2.822	3.015	3.213	3.416	3.626	3.845	4.067	4.298	4.531	4.771	5.020
32	1.247	1.375	1.510	1.648	1.795	1.949	2.108	2.274	2.445	2.623	2.806	2.998	3.192	3.398	3.608	3.819	4.041	4.267	4.503	4.743	4.989
34	1.241	1.368	1.501	1.641	1.786	1.940	2.096	2.261	2.430	2.607	2.791	2.982	3.175	3.376	3.584	3.802	4.017	4.246	4.478	4.717	4.963
35	1.237	1.365	1.497	1.636	1.781	1.934	2.092	2.255	2.426	2.601	2.782	2.972	3.166	3.368	3.576	3.790	4.009	4.233	4.466	4.704	4.948
36	1.234	1.361	1.494	1.632	1.777	1.929	2.088	2.250	2.418	2.594	2.779	2.966	3.161	3.361	3.566	3.780	4.001	4.226	4.453	4.696	4.935
38	1.228	1.354	1.486	1.624	1.770	1.918	2.077	2.239	2.406	2.583	2.766	2.953	3.143	3.346	3.550	3.763	3.981	4.204	4.437	4.671	4.916
40	1.224	1.348	1.480	1.618	1.762	1.910	2.067	2.229	2.398	2.571	2.753	2.939	3.134	3.331	3.537	3.748	3.965	4.187	4.419	4.653	4.894
45	1.212	1.336	1.467	1.603	1.745	1.895	2.049	2.209	2.377	2.548	2.727	2.911	3.101	3.300	3.502	3.710	3.929	4.148	4.374	4.608	4.851
50	1.203	1.327	1.455	1.592	1.733	1.881	2.034	2.192	2.357	2.530	2.706	2.891	3.081	3.274	3.477	3.684	3.900	4.117	4.343	4.577	4.812
