# Supplementary material for: Quantification of Difference in Nonselectivity Between In Vitro Diagnostic Medical Devices
Source: Biom J. 2025 Jan 2;67(1):e70032. doi: 10.1002/bimj.70032 (PMC11695778; doi:10.1002/bimj.70032)
Supplement: Supplementary file 1 — Supporting Information [file BIMJ-67-e70032-s001.zip › Reproducibility resubmission v2/results pkf 22 10 2024 15 cores/Reproducing-manuscript-results_files/figure-latex/percentiles-of-zeta-3-4-1.pdf]

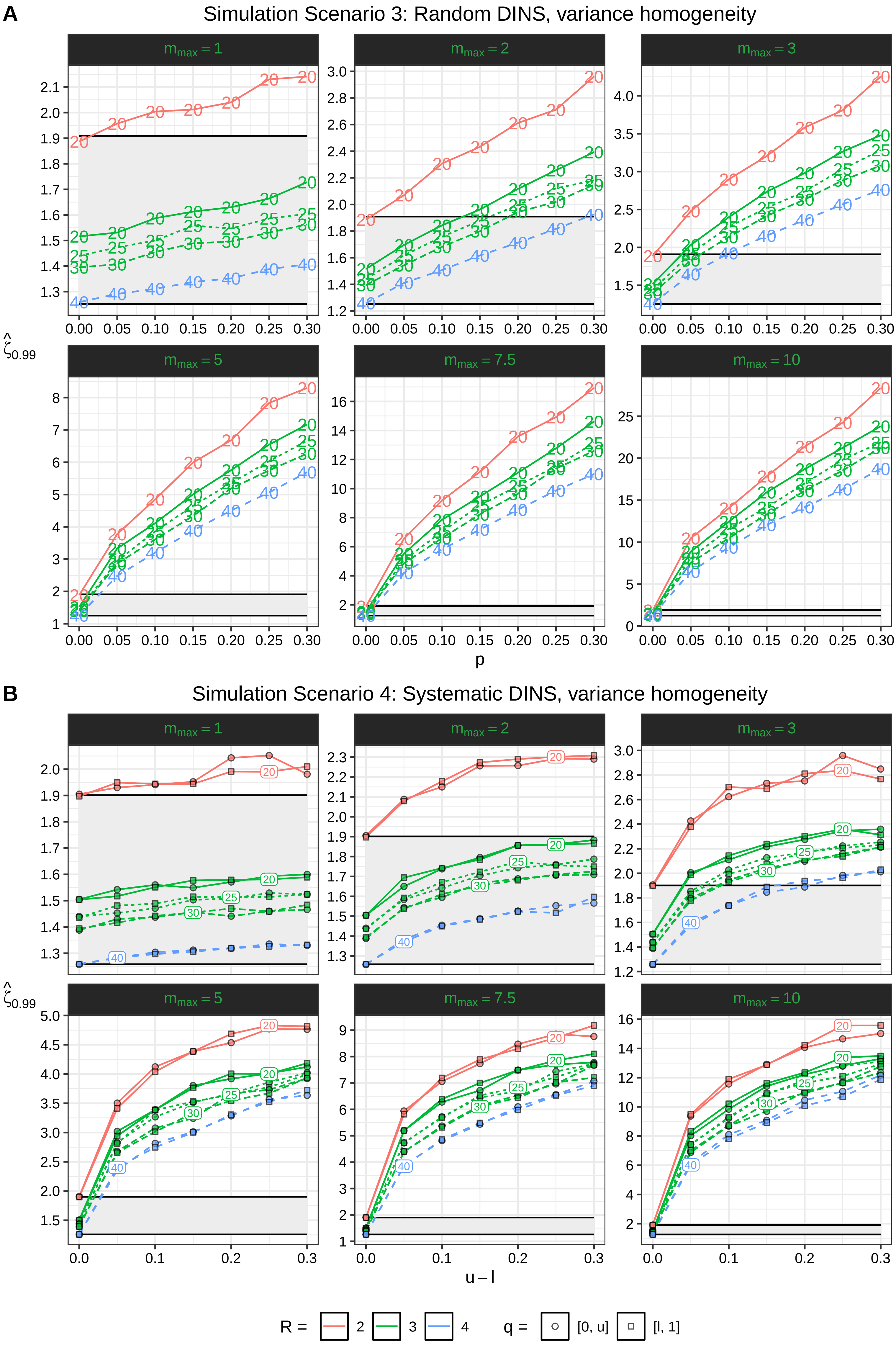

A
Simulation Scenario 3: Random DINS, variance homogeneity
$m_{max} = 1$
$m_{max} = 2$
$m_{max} = 3$
$m_{max} = 5$
$m_{max} = 7.5$
$m_{max} = 10$
$\hat{\zeta}_{0.99}$
p
B
Simulation Scenario 4: Systematic DINS, variance homogeneity
$m_{max} = 1$
$m_{max} = 2$
$m_{max} = 3$
$m_{max} = 5$
$m_{max} = 7.5$
$m_{max} = 10$
$\hat{\zeta}_{0.99}$
u – l
R = 2 3 4
q = [0, u] [l, 1]
