# Supplementary material for: Quantification of Difference in Nonselectivity Between In Vitro Diagnostic Medical Devices
Source: Biom J. 2025 Jan 2;67(1):e70032. doi: 10.1002/bimj.70032 (PMC11695778; doi:10.1002/bimj.70032)
Supplement: Supplementary file 1 — Supporting Information [file BIMJ-67-e70032-s001.zip › Reproducibility resubmission v2/results pkf 22 10 2024 15 cores/Reproducing-manuscript-results_files/figure-latex/simulation-1-to-4-illustration-1.pdf]

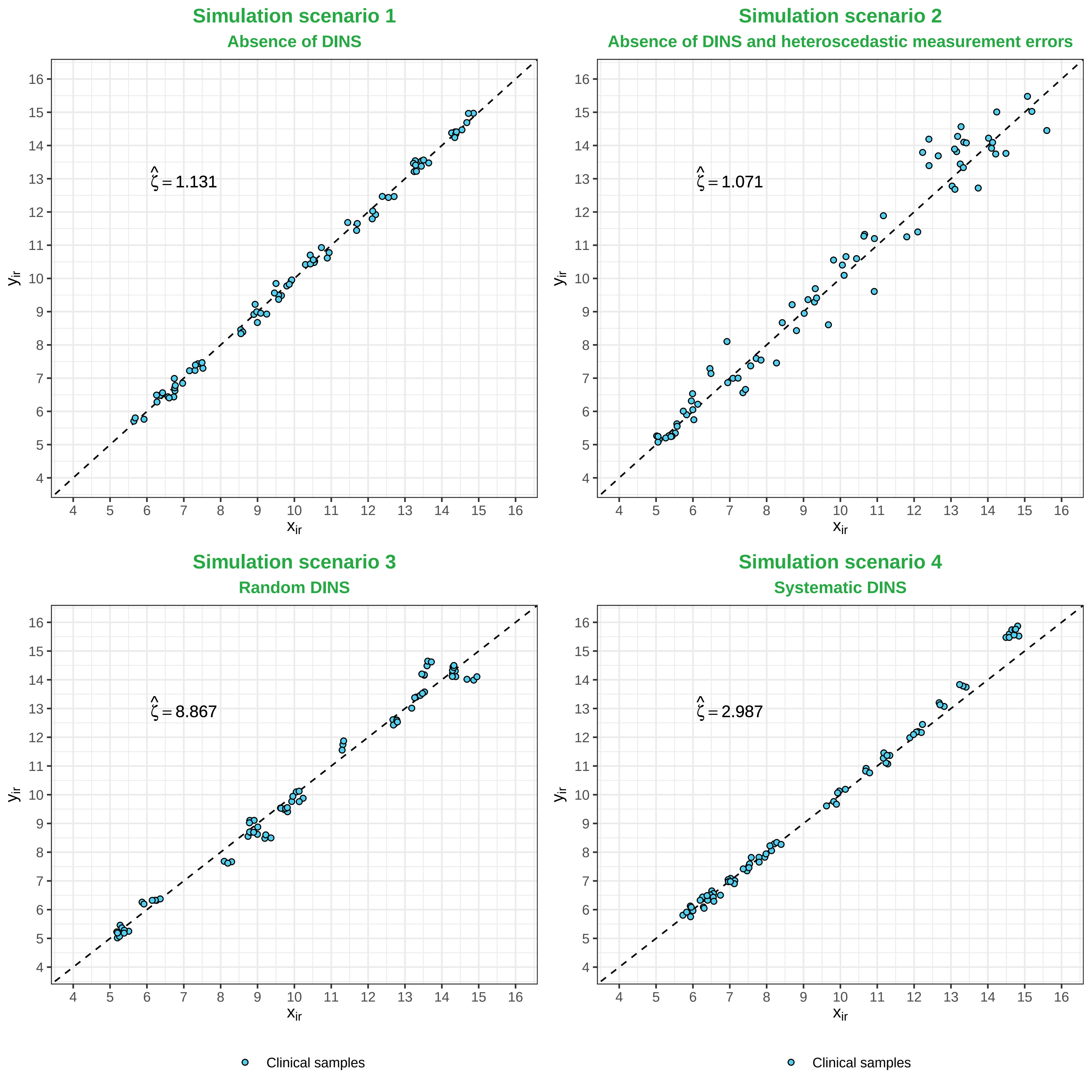

Simulation scenario 1
Absence of DINS
$\hat{\zeta} = 1.131$
Simulation scenario 2
Absence of DINS and heteroscedastic measurement errors
$\hat{\zeta} = 1.071$
Simulation scenario 3
Random DINS
$\hat{\zeta} = 8.867$
Simulation scenario 4
Systematic DINS
$\hat{\zeta} = 2.987$
x_{ir}
y_{ir}
Clinical samples
