# Supplementary material for: Quantification of Difference in Nonselectivity Between In Vitro Diagnostic Medical Devices
Source: Biom J. 2025 Jan 2;67(1):e70032. doi: 10.1002/bimj.70032 (PMC11695778; doi:10.1002/bimj.70032)
Supplement: Supplementary file 1 — Supporting Information [file BIMJ-67-e70032-s001.zip › Reproducibility resubmission v2/results pkf 22 10 2024 15 cores/Supplemental-file-repr.pdf]

Quantification of difference in non-selectivity between in vitro diagnostic medical devices

Supplemental files

Pernille Kjeilen Fauskanger[a,b*] Sverre Sandberg[a,c,d] Jesper Johansen[e]
Thomas Keller[f] Jeffrey Budd[g] W. Greg Miller[h] Anne Stavelin[a]
Vincent Delatour[i] Mauro Panteghini[j] Bård Støve[b]

[*] Corresponding author; E-mail: pernille.fauskanger@noklus.no

[a] Norwegian Organization for Quality Improvement of Laboratory Examinations (Noklus), Haraldsplass Deaconess Hospital, Bergen, Norway.

[b] Department of Mathematics, University of Bergen, Bergen, Norway.

[c] Department of Global Public Health and Primary Care, University of Bergen, Bergen, Norway.

[d] Department of Medical Biochemistry and Pharmacology, Haukeland University Hospital, Bergen, Norway.

[e] Radiometer Medical ApS, Copenhagen, Denmark.

[f] ACOMED Statistic, Leipzig, Germany.

[g] Jeff Budd Consulting, St. Paul, MN, United States.

[h] Department of Pathology, Virginia Commonwealth University, Richmond, VA, United States.

[i] Laboratoire national de métrologie et d'essais, Paris, France.

[j] Department of Laboratory Medicine, Ludwik Rydygier Collegium Medicum in Bydgoszcz, Nicolaus Copernicus University in Torun, Torun, Poland.

1 Simulations introduction

Differences in non-selectivity (DINS) are formally conceptualized as the observed discrepancies in clinical sample (CS) measurement results, specifically, those discrepancies that exceed anticipated random variations due to inherent analytical uncertainties of compared in vitro diagnostic medical devices (IVD-MDs).

In such a IVD-MD comparison, individual measurement results from one of the IVD-MDs, IVD-MD$_X$, are represented by $\{x_{ir} \ : \ (i, r) \in \{1, \dots, n\} \times \{1, \dots, R\}\}$. The corresponding measurement results from the other IVD-MD in the comparison, IVD-MD$_Y$, are denoted by $\{y_{ir} \ : \ (i, r) \in \{1, \dots, n\} \times \{1, \dots, R\}\}$. The indices i and r indicate the CS identifier (ID) and replicate measurement ID, respectively. In order to quantify the DINS magnitude of a IVD-MD pair, we regard the univariate ordinary least squares (OLS) regression model:

$$y_{ir} = \beta_0 + \beta_1 x_{ir} + e_{ir}; \ e_{ir} \sim \mathrm{N}(0, \sigma^2). \tag{1}$$

Within this model, β_0 and β_1 represent the intercept and slope coefficients respectively, while e_{ir} corresponds to the model error terms. Both x_{ir} and y_{ir} are assumed to be influenced my normally distributed measurement errors with mean 0, parameterized by variances $\sigma^2_{\text{IVD-MD}_X}$ and $\sigma^2_{\text{IVD-MD}_Y}$, respectively.

The goal of the simulations presented in this supplemental material is twofold: to validate our theoretical findings and to identify potential correlations between $\hat{\zeta}$—an estimator quantifying differences in non-selectivity (DINS)—and the considered simulation parameters. We define:

$$\hat{\zeta} = \frac{S^2_{\mathrm{P}}}{\hat{\sigma}^2_{\text{IVD-MD}_Y} + \hat{\beta}_1^2 \cdot \hat{\sigma}^2_{\text{IVD-MD}_X}} \tag{2}$$

Herein, $\hat{\beta}_1$ stands as the unbiased estimator of β_1. Both $\hat{\sigma}^2_{\text{IVD-MD}_X}$ and $\hat{\sigma}^2_{\text{IVD-MD}_Y}$ serve as the unbiased estimators of $\sigma^2_{\text{IVD-MD}_X}$ and $\sigma^2_{\text{IVD-MD}_Y}$, respectively. Moreover, $S^2_{\mathrm{P}} = S^2 \cdot \frac{nR+2}{nR}$, where S denotes the mean sum of squares error of the univariate OLS regression model:

$$S^2 = \frac{1}{nR - 2} \sum_{i=1}^{n} \sum_{r=1}^{R} (\hat{y}_{ir} - y_{ir})^2. \tag{3}$$

In this framework, S^2 is an estimator of σ^2, and $\hat{y}_{ir}$ is the OLS prediction of y_{ir} predicated on x_{ir}.

The forthcoming simulation study provides an exhaustive exploration of $\hat{\zeta}$ across a diverse range of simulation parameters, with the intent of discerning its correlations with these parameter combinations. Four primary simulation settings are contemplated, each endowed with a distinctive parameter set. The first duo of settings concentrates on IVD-MD comparisons devoid of DINS, with the second explicitly incorporating heteroscedastic measurement errors. The subsequent three settings integrates parameters pertinent to DINS. Settings 3 and 4 focus on two distinct modalities of DINS—one that exerts a random influence on CS measurements and another that systematically modulates them based on inherent analyte concentration values. The fifth setting scrutinizes the relationship between DINS and OLS point-wise prediction interval widths, predicated on a singular DINS parameter.

2 Simulation settings

Each simulation setting comprises a finite sequence of steps, with varying numbers of steps and their contents differing across simulation settings. However, the initial four steps remain consistent across all settings:

1. **Generating the number of CSs**, denoted by n
2. **Generating the number of measurement replicates**, represented by R.
3. **Generating the analytical imprecision of the two IVD-MDs under comparison**. IVD-MD analytical imprecision can be expressed using $\mathrm{CV}_{\text{IVD-MD}_X}$ and $\mathrm{CV}_{\text{IVD-MD}_Y}$ (IVD-MD coefficients of variation), $\sigma^2_{\text{IVD-MD}_X}$ and $\sigma^2_{\text{IVD-MD}_Y}$ (IVD-MD variances), or $\sigma_{\text{IVD-MD}_X}$ and $\sigma_{\text{IVD-MD}_Y}$ (IVD-MD standard deviations). However, $\mathrm{CV}_{\text{IVD-MD}_X}$ and $\mathrm{CV}_{\text{IVD-MD}_Y}$ are the raw simulation parameters.
4. **Generating the concentration interval's lower and upper limits**, U_1 and U_2. U_2 is generated by first generating $T > 0$, and then calculated using $U_2 = U_1(1 + T)$.

Each of these steps can either involve drawing the parameter from a specific probability distribution or manually setting it. When a parameter is set manually, the corresponding step is skipped. For instance:

- If we manually decide on 25 CSs measured in triplicate, then $n = 25$ and $R = 3$, skipping the first two steps.
- If we specify that $\mathrm{CV}_{\text{IVD-MD}_X}$ is 2% and $\mathrm{CV}_{\text{IVD-MD}_Y}$ is 1%, then the third step, which involves determining the analytical imprecision, is bypassed.
- If we specify that $U_1 = 100$ and $T = 1$, then $U_2 = 200$, skipping step four.

The probability distributions used for the random variables in the four initial simulation setting steps are derived from our observations in clinical sample data and from the reference intervals seen across various analytes.

2.1 Simulation setting 1: Identical non-selectivity profiles and homoscedastic measurement errors

Simulation setting 1: Simulates values of $\hat{\zeta}$ with IVD-MDs devoid of DINS, where the IVD-MD results are influenced by homoscedastic measurement errors:

1. Draw n (number of CSs) from a truncated Poisson distribution with limits at 20 and 30:

$$n \sim \max\Big[20, \min\big[\mathrm{Poisson}(25), 30\big]\Big].$$

2. Draw R (number of measurement replicates) from the set $\{2, 3, 4\}$ with point mass probabilities $(2/20, 17/20, 1/20)$, accounting for the commonality of three replicates over two or four.
3. Draw $\mathrm{CV}_{\text{IVD-MD}_X}$, $\mathrm{CV}_{\text{IVD-MD}_Y}$ from a beta distribution scaled by a factor of $1/10$:

$$\mathrm{CV}_{\text{IVD-MD}_X},\ \mathrm{CV}_{\text{IVD-MD}_Y} \sim \mathrm{Beta}(2, 5)/10.$$

4. Draw the lower limit of the concentration interval, U_1, from an F-distribution scaled by 44:

$$U_1 \sim F_{1.06, 8.15} \cdot 44.$$

Subsequently, determine T from a beta distribution (also scaled by 44) and compute the concentration interval upper limit, U_2, as:

$$T \sim \text{Beta}(0.78, 11) \cdot 44 \qquad\qquad U_2 = U_1(1 + T).$$

5. For each CS, draw latent concentration values, τ_i, uniformly within the defined concentration interval:

$$\tau_i \sim \text{Uniform}(U_1, U_2), \text{ for } i = 1, \ldots, n.$$

6. Derive IVD-MD standard deviations $\sigma_{\text{IVD-MD}_X}$ and $\sigma_{\text{IVD-MD}_Y}$ using the coefficients of variation, $\text{CV}_{\text{IVD-MD}_X}$ and $\text{CV}_{\text{IVD-MD}_Y}$, and the mid-point of the concentration interval:

$$\sigma_{\text{IVD-MD}_X} = \text{CV}_{\text{IVD-MD}_X} \cdot \frac{1}{2}[U_1 + U_2]$$
$$\sigma_{\text{IVD-MD}_Y} = \text{CV}_{\text{IVD-MD}_Y} \cdot \frac{1}{2}[U_1 + U_2].$$

7. Simulate observed IVD-MD measurements, x_{ir} and y_{ir}, by adding measurements errors to τ_i:

$$x_{ir} = \tau_i + \text{N}(0, \sigma^2_{\text{IVD-MD}_X})_r; \ r = 1, \ldots R$$
$$y_{ir} = \tau_i + \text{N}(0, \sigma^2_{\text{IVD-MD}_Y})_r; \ r = 1, \ldots R.$$

   The simulated values x_{ir} represents measurements based on IVD-MD_X, and y_{ir} signify measurements from IVD-MD_Y.

8. Calculate $\hat{\sigma}^2_{\text{IVD-MD}_X}$ and $\hat{\sigma}^2_{\text{IVD-MD}_Y}$ from the simulated observed measurements from step seven.
9. Calculate S^2_{P} and $\hat{\beta}_1$, using the OLS regression, with y_{ir} as responses and x_{ir} as predictors.
10. Calculate $\hat{\zeta}$ using the outputs from steps eight and nine.

2.2 Simulation setting 2: Identical non-selectivity profiles and heteroscedastic measurement errors

Simulation setting 2: Simulates values of $\hat{\zeta}$ with IVD-MDs devoid of DINS, where the IVD-MD results are influenced by heteroscedastic measurement errors:

We introduce two parameters, η_0 and η, to model heteroscedastic measurement errors in a pair of IVD-MDs. The parameter η_0 serves as a multiplier of the IVD-MD *base standard deviations* at the lower concentration interval, adjusting their magnitude. Meanwhile, η represent the *heteroscedasticity factor*. Specifically, it defines the scale of the standard deviations at the upper concentration interval limit relative to the adjusted standard deviations at the lower concentration interval limit. For a given concentration interval, if

- $\eta > 1$ and $\eta_0 \geq 1$, the IVD-MD standard deviations linearly increase across the interval.
- $\eta < 1$, they decrease linearly, irrespective of the value of η_0.

Mathematically, the j-th pair of IVD-MD standard deviations, denoted as $[\sigma_{\text{IVD-MD}_X}]_j$ and $[\sigma_{\text{IVD-MD}_Y}]_j$,

where j ranges from 1 to n, is calculated as:

$$[\sigma_{\text{IVD-MD}_X}]_j = \eta_0 \cdot [\text{base standard deviation}]_X + j \cdot \frac{\eta_0 \cdot [\text{base standard deviation}]_X \cdot (\eta - 1)}{n} \tag{4}$$

$$[\sigma_{\text{IVD-MD}_Y}]_j = \eta_0 \cdot [\text{base standard deviation}]_Y + j \cdot \frac{\eta_0 \cdot [\text{base standard deviation}]_Y \cdot (\eta - 1)}{n}. \tag{5}$$

In these simulations, [base standard deviation]$_X = \sigma_{\text{IVD-MD}_X}$ and [base standard deviation]$_Y = \sigma_{\text{IVD-MD}_Y}$. For instance, for $\eta = 2$ and $\eta_0 = 1$, IVD-MD standard deviations will double over the concentration interval, starting at their base values. Conversely, if $\eta = 1/2$ and $\eta_0 = 1/4$, they will decrease from a quarter of their base values to an eighth.

Notably, if $\eta = 1$, the pairs of IVD-MD standard deviations remain constant (homoscedastic IVD-MD measurement errors) across the concentration interval, irrespective of η_0. Particularly, when both η and η_0 equal 1, this simulation setting mirrors the first simulation setting with homoscedastic measurement errors.

With the principles of heteroscedasticity, as guided by, η and η_0, established, we can detail the second simulation setting for $\hat{\zeta}$:

1. Draw n from a truncated Poisson distribution with limits at 20 and 30:

$$n \sim \max\Big[20, \min\big[\text{Poisson}(25), 30\big]\Big].$$

2. Draw R from the set $\{2, 3, 4\}$ with point mass probabilities $(2/20, 17/20, 1/20)$, accounting for the commonality of three replicates over two or four.
3. Draw $\text{CV}_{\text{IVD-MD}_X}$, $\text{CV}_{\text{IVD-MD}_Y}$ from a beta distribution scaled by a factor of $1/10$:

$$\text{CV}_{\text{IVD-MD}_X},\ \text{CV}_{\text{IVD-MD}_Y} \sim \text{Beta}(2, 5)/10.$$

4. Draw the lower limit of the concentration interval, U_1, from an F-distribution scaled by 44:

$$U_1 \sim F_{1.06, 8.15} \cdot 44.$$

   Subsequently, determine T from a beta distribution (also scaled by 44) and compute the concentration interval upper limit, U_2, as:

$$T \sim \text{Beta}(0.78, 11) \cdot 44 \qquad\qquad U_2 = U_1(1 + T).$$

5. For each CS, draw latent concentration values, τ_i, uniformly within the defined concentration interval:

$$\tau_i \sim \text{Uniform}(U_1, U_2),\ \text{for } i = 1, \ldots, n.$$

   Determine the order statistics of τ_i values. Specifically, $\tau_{(i)}$ represents the i-th smallest value among the set $\{\tau_1, \ldots, \tau_n\}$.
6. Derive IVD-MD standard deviations $\sigma_{\text{IVD-MD}_X}$ and $\sigma_{\text{IVD-MD}_Y}$ using the coefficients of variation,

$\mathrm{CV}_{\text{IVD-MD}_X}$ and $\mathrm{CV}_{\text{IVD-MD}_Y}$, and the mid-point of the concentration interval:

$$\sigma_{\text{IVD-MD}_X} = \mathrm{CV}_{\text{IVD-MD}_X} \cdot \frac{1}{2}[U_1 + U_2]$$
$$\sigma_{\text{IVD-MD}_Y} = \mathrm{CV}_{\text{IVD-MD}_Y} \cdot \frac{1}{2}[U_1 + U_2].$$

7. After defining the base IVD-MD standard deviations as $[\text{base standard deviation}]_X = \sigma_{\text{IVD-MD}_X}$ and $[\text{base standard deviation}]_Y = \sigma_{\text{IVD-MD}_Y}$, compute the n equally spaced concentration dependent IVD-MD standard deviations for both IVD-MD$_X$ and IVD-MD$_Y$ standard deviation intervals:

$$[\eta_0 \cdot \sigma_{\text{IVD-MD}_X},\ \eta \cdot \eta_0 \cdot \sigma_{\text{IVD-MD}_X}]$$
$$[\eta_0 \cdot \sigma_{\text{IVD-MD}_Y},\ \eta \cdot \eta_0 \cdot \sigma_{\text{IVD-MD}_Y}].$$

This results in two sets, each of size n:

$$\big\{[\sigma_{\text{IVD-MD}_X}]_j :\ j \in \{1, \ldots, n\}\big\}$$
$$\big\{[\sigma_{\text{IVD-MD}_Y}]_j :\ j \in \{1, \ldots, n\}\big\}.$$

Here,

$$[\sigma_{\text{IVD-MD}_X}]_j = \eta_0 \cdot \sigma_{\text{IVD-MD}_X} + j \cdot \frac{\eta_0 \cdot \sigma_{\text{IVD-MD}_X} \cdot (\eta - 1)}{n}$$
$$[\sigma_{\text{IVD-MD}_Y}]_j = \eta_0 \cdot \sigma_{\text{IVD-MD}_Y} + j \cdot \frac{\eta_0 \cdot \sigma_{\text{IVD-MD}_Y} \cdot (\eta - 1)}{n}.$$

8. For each order statistic $\tau_{(i)}$ where $i = 1, \ldots, n$, apply measurement errors using the corresponding standard deviations $[\sigma_{\text{IVD-MD}_X}]_j$ and $[\sigma_{\text{IVD-MD}_Y}]_j$ where $j = i$:

$$x_{ir} = \tau_{(i)} + \mathcal{N}\Big(0, [\sigma_{\text{IVD-MD}_X}]_j^2\Big)_r,\ r = 1, \ldots R \tag{6}$$
$$y_{ir} = \tau_{(i)} + \mathcal{N}\Big(0, [\sigma_{\text{IVD-MD}_Y}]_j^2\Big)_r,\ r = 1, \ldots R \tag{7}$$

The simulated values x_{ir} represents measurements based on IVD-MD$_X$, and y_{ir} signify measurements from IVD-MD$_Y$.

9. Calculate $\hat{\sigma}^2_{\text{IVD-MD}_X}$ and $\hat{\sigma}^2_{\text{IVD-MD}_Y}$ from the simulated observed measurements from step eight.
10. Calculate S^2_{P} and $\hat{\beta}_1$, using the OLS regression, with y_{ir} as responses and x_{ir} as predictors.
11. Calculate $\hat{\zeta}$ using the outputs from steps nine and ten.

2.3 Simulation setting 3: Homoscedastic measurement errors with differences in non-selectivity randomly impacting CS results

Simulation setting 3: Simulates values of $\hat{\zeta}$ with IVD-MDs demonstrating *random DINS*, where the IVD-MD results are influenced by homoscedastic measurement errors:

In this context, p signifies the probability that a CS is impacted by *random differences in non-selectivity* (termed as random DINS) in an IVD-MD comparison. Such an occurrence of random DINS signify that replicated measurements for a designated CS drift away from the theoretical regression line on the XY-

plane. This line typifies the linear interrelationship between IVD-MD$_X$ and IVD-MD$_Y$. The extent of this drift ranges between 0 up to a predefined *maximum relocation magnitude multiplier*, symbolized by $m_{\max}$, multiplied by the *base standard deviation.* In this simulation setting,

$$\text{base standard deviation} = \sqrt{\sigma^2_{\text{IVD-MD}_X} + \sigma^2_{\text{IVD-MD}_Y}}. \tag{8}$$

The product $m_{\max} \cdot \sqrt{\sigma^2_{\text{IVD-MD}_X} + \sigma^2_{\text{IVD-MD}_Y}}$ therefore refers to the *maximum relocation magnitude.* Thus, the extent of the drift ranges between 0 and maximum relocation magnitude.

If both p and $m_{\max}$ are set to values greater than 0, this introduces a random DINS effect to the simulated data. The magnitudes of this effect is directly related to the values chosen for p and $m_{\max}$. For a given CS with identifier (ID) i, having replicated measurements $y^{\star}_{i1}, \ldots, y^{\star}_{iR}$, the corresponding random DINS-affected replicated measurements are determined:

$$y_{ir} = y^{\star}_{ir} + Y_i \cdot D_i, \; r = 1, \ldots, R. \tag{9}$$

The random variable Y_i, illustrating the relocation magnitude due to random DINS and has support on $[0, m_{\max} \cdot \sqrt{\sigma^2_{\text{IVD-MD}_X} + \sigma^2_{\text{IVD-MD}_Y}}]$. Y_i must therefore be generated from a probability distribution with this exact support. Accordingly, the beta distribution is apt for simulating Y_i. For instance,

$$Y_i \sim \text{Beta}(2, 2) \cdot m_{\max} \cdot \sqrt{\sigma^2_{\text{IVD-MD}_X} + \sigma^2_{\text{IVD-MD}_Y}} \tag{10}$$

is supported on $[0, m_{\max} \cdot \sqrt{\sigma^2_{\text{IVD-MD}_X} + \sigma^2_{\text{IVD-MD}_Y}}]$ and is symmetrical around $1/2 \cdot m_{\max} \cdot \sqrt{\sigma^2_{\text{IVD-MD}_X} + \sigma^2_{\text{IVD-MD}_Y}}$ making it an ideal choice. The random variable D_i represents the directional shift: a value of -1 suggests a downward shift in the replicated measurements, whereas a value of 1 indicates an upward shift. Because D_i is required to have support on $\{-1, 1\}$ where both outcomes have equal probability, we define

$$D_i \sim 1 - 2 \cdot \text{binomial}(1, 1/2). \tag{11}$$

With the term random DINS meticously defined, here is how we simulate values of $\hat{\zeta}$ when IVD-MD pairs exhibit random DINS:

1. Draw n from a truncated Poisson distribution with limits at 20 and 30:

$$n \sim \max\Big[20, \min\big[\text{Poisson}(25), 30\big]\Big].$$

2. Draw R from the set $\{2, 3, 4\}$ with point mass probabilities $(2/20, 17/20, 1/20)$, accounting for the commonality of three replicates over two or four.
3. Draw $\text{CV}_{\text{IVD-MD}_X}$, $\text{CV}_{\text{IVD-MD}_Y}$ from a beta distribution scaled by a factor of $1/10$:

$$\text{CV}_{\text{IVD-MD}_X}, \; \text{CV}_{\text{IVD-MD}_Y} \sim \text{Beta}(2, 5)/10.$$

4. Draw the lower limit of the concentration interval, U_1, from an F-distribution scaled by 44:

$$U_1 \sim F_{1.06, 8.15} \cdot 44.$$

Subsequently, determine T from a beta distribution (also scaled by 44) and compute the concentration interval upper limit, U_2, as:

$$T \sim \text{Beta}(0.78, 11) \cdot 44 \qquad\qquad U_2 = U_1(1 + T).$$

5. For each CS, draw latent concentration values, τ_i, uniformly within the defined concentration interval:

$$\tau_i \sim \text{Uniform}(U_1, U_2), \text{ for } i = 1, \ldots, n.$$

6. Derive IVD-MD standard deviations $\sigma_{\text{IVD-MD}_X}$ and $\sigma_{\text{IVD-MD}_Y}$ using the coefficients of variation, $\text{CV}_{\text{IVD-MD}_X}$ and $\text{CV}_{\text{IVD-MD}_Y}$, and the mid-point of the concentration interval:

$$\sigma_{\text{IVD-MD}_X} = \text{CV}_{\text{IVD-MD}_X} \cdot \frac{1}{2}[U_1 + U_2]$$
$$\sigma_{\text{IVD-MD}_Y} = \text{CV}_{\text{IVD-MD}_Y} \cdot \frac{1}{2}[U_1 + U_2].$$

7. Simulate IVD-MD measurements unaffected by random DINS, $x_{ir}^{\star}$ and $y_{ir}^{\star}$, by adding measurements errors to τ_i:

$$x_{ir}^{\star} = \tau_i + \text{N}(0, \sigma^2_{\text{IVD-MD}_X})_r;\ r = 1, \ldots R$$
$$y_{ir}^{\star} = \tau_i + \text{N}(0, \sigma^2_{\text{IVD-MD}_Y})_r;\ r = 1, \ldots R.$$

   The simulated values $x_{ir}^{\star}$ represents unaffected measurements based on IVD-MD_X, and $y_{ir}^{\star}$ signify unaffected measurements from IVD-MD_Y.
8. Relocate clusters of replicated measurements according to randomly selected CSs to be affected with random DINS:
   a. Draw $N \sim \text{binomial}(n, p)$. If $N = 0$, set $X = \emptyset$, $Y_j = 0$ and $D_j = 0$, and move to step nine. If $N > 0$, jump to sub step b.
   b. Draw N observations (without replacement) uniformly from $\{1, 2, \ldots, n\}$ and denote the set of drawn numbers by X.
   c. Draw N values from $\text{Beta}(2, 2) \cdot m_{\max} \cdot$ base standard deviation, referred to by $\{Y_j;\ j \in X\}$.
   d. Draw N direction observations from $1 - 2 \cdot \text{binomial}(1,\ 1/2)$, referred to by $\{D_j;\ j \in X\}$.
9. Add the relocation effects due to random DINS to the non-affected measurement results of IVD-MD_Y:

$$y_{ir} = y_{ir}^{\star} + Y_i \cdot D_i \cdot \mathbb{I}(i \in X);\ i = 1, \ldots, n \text{ and } r = 1, \ldots, R \tag{12}$$

10. Calculate $\hat{\sigma}^2_{\text{IVD-MD}_X}$, $\hat{\sigma}^2_{\text{IVD-MD}_Y}$ by using the simulated observed measurements from step seven and nine.
11. Calculate S^2_{P} and $\hat{\beta}_1$, using the OLS regression, with y_{ir} as responses and $x_{ir}^{\star}$ as predictors.
12. Calculate $\hat{\zeta}$ based on the computed quantities of steps ten and eleven.

2.4 Simulation setting 4: Homoscedastic measurement errors with differences in non-selectivity systematically impacting CS results

Simulation setting 4: Simulates values of $\hat{\zeta}$ with IVD-MDs demonstrating *systematic DINS*, where the IVD-MD results are influenced by homoscedastic measurement errors:

Let $q = [l, u]$ be the *quantile interval*, with a *quantile range* $u - l$, derived from the quantile sub-interval of $[0, 1]$. This interval maps to a specific concentration sub-interval of the full concentration interval. This means that q corresponds to a set of true analyte concentration values, represented by Q, within the defined concentration sub-interval. Considering the CSs influenced by systematic differences in non-selectivity (systematic DINS), these CSs possess true analyte concentration values that are defined by:

$$Q = \{\tau_i : l \leq \mathrm{P}(\tau \leq \tau_i) \leq u\}. \tag{13}$$

For instance, if $l = 0$ and $u = 0.25$, then $q = [0, 0.25]$. We then select the subset of $\{\tau_1, \ldots, \tau_n\}$ that satisfies $\mathrm{P}(\tau \leq \tau_i) \leq 0.25$. Thus, if $U_1 = 50$ and $U_2 = 100$, and the true analyte concentration values are uniformly distributed between these two limits, the values of $\{\tau_i, \ldots, \tau_n\}$ being smaller than or equal to 62.5 are those affected by systematic DINS. Conversely, for $l = 0.70$ and $u = 1$, the subset is such that $0.70 \leq \mathrm{P}(\tau \leq \tau_i) \leq 1$. The selected set based on u and l, represented as Q, will undergo relocation on the XY-plane. In fact, measurements from CSs affected by systematic DINS are relocated from the regression line that defines the relationship between IVD-MD$_X$ and IVD-MD$_Y$.

Differing from simulation setting 3, the relocation magnitudes here are deterministic, not random. Moreover, they vary and increase linearly towards the concentration interval edges when $\{l = 0, u > 0\}$ or $\{l < 1, u = 1\}$ – the most realistic scenarios. The former condition indicates systematic DINS in the lower range of the concentration interval, termed *lower systematic DINS*, while the latter indicates systematic DINS in the upper range of the concentration interval, termed *upper systematic DINS*. Here, we will only consider these two forms of systematic DINS, as they are the most prevalent.

The base standard deviation here matches that from simulation setting 3. Thus, the maximal relocation magnitude of systematic DINS-affected CSs is the same as in simulation setting 3: $m_{\max} \cdot \sqrt{\sigma^2_{\text{IVD-MD}_X} + \sigma^2_{\text{IVD-MD}_Y}}$. That is, $m_{\max}$ remains the maximal relocation magnitude multiplier. However, actual relocation magnitudes vary between the two simulation settings. In simulation setting 3, the relocation magnitudes were random variables. However, in this simulation setting, the relocation magnitudes, Y_i, are functions τ_i, q, and U_1 and U_2 (the concentration interval limits). Specifically,

$$Y_i = m_{\max} \cdot \sqrt{\sigma^2_{\text{IVD-MD}_X} + \sigma^2_{\text{IVD-MD}_Y}} \cdot \begin{cases} \dfrac{U_1 + u \cdot (U_2 - U_1) - \tau_i}{u \cdot (U_2 - U_1)}, & \text{for } l = 0, u > 0 \\ \dfrac{\tau_i - U_2 + (1 - l) \cdot (U_2 - U_1)}{(1 - l) \cdot (U_2 - U_1)}, & \text{for } l < 1, u = 1. \end{cases}$$

The direction of systematic relocations can be either upward or downward, determined by $D \sim 1 - 2 \cdot \text{binomial}(1, 1/2)$.

With the principles covered, we simulate $\hat{\zeta}$ values base on simulation setting 4 by following this sequence of steps:

1. Draw n (number of CSs) from a truncated Poisson distribution with limits at 20 and 30:

$$n \sim \max\Big[20, \min\big[\text{Poisson}(25), 30\big]\Big].$$

2. Draw R (number of measurement replicates) from the set $\{2, 3, 4\}$ with point mass probabilities ($^2/_{20}, {}^{17}/_{20}, {}^1/_{20}$), accounting for the commonality of three replicates over two or four.
3. Draw $\text{CV}_{\text{IVD-MD}_X}$, $\text{CV}_{\text{IVD-MD}_Y}$ from a beta distribution scaled by a factor of $^1/_{10}$:

$$\text{CV}_{\text{IVD-MD}_X},\ \text{CV}_{\text{IVD-MD}_Y} \sim \text{Beta}(2, 5)/10.$$

4. Draw the lower limit of the concentration interval, U_1, from an F-distribution scaled by 44:

$$U_1 \sim F_{1.06, 8.15} \cdot 44.$$

Subsequently, determine T from a beta distribution (also scaled by 44) and compute the concentration interval upper limit, U_2, as:

$$T \sim \text{Beta}(0.78, 11) \cdot 44 \qquad\qquad U_2 = U_1(1 + T).$$

5. For each CS, draw latent concentration values, τ_i, uniformly within the defined concentration interval:

$$\tau_i \sim \text{Uniform}(U_1, U_2),\ \text{for } i = 1, \ldots, n.$$

6. Derive IVD-MD standard deviations $\sigma_{\text{IVD-MD}_X}$ and $\sigma_{\text{IVD-MD}_Y}$ using the coefficients of variation, $\text{CV}_{\text{IVD-MD}_X}$ and $\text{CV}_{\text{IVD-MD}_Y}$, and the mid-point of the concentration interval:

$$\sigma_{\text{IVD-MD}_X} = \text{CV}_{\text{IVD-MD}_X} \cdot \frac{1}{2}[U_1 + U_2]$$
$$\sigma_{\text{IVD-MD}_Y} = \text{CV}_{\text{IVD-MD}_Y} \cdot \frac{1}{2}[U_1 + U_2].$$

7. Simulate IVD-MD measurements unaffected by systematic DINS, $x_{ir}^\star$ and $y_{ir}^\star$, by adding measurements errors to τ_i:

$$x_{ir}^\star = \tau_i + \text{N}(0, \sigma^2_{\text{IVD-MD}_X})_r;\ r = 1, \ldots R$$
$$y_{ir}^\star = \tau_i + \text{N}(0, \sigma^2_{\text{IVD-MD}_Y})_r;\ r = 1, \ldots R.$$

The simulated values $x_{ir}^\star$ represents unaffected measurements based on IVD-MD_X, and $y_{ir}^\star$ signify unaffected measurements from IVD-MD_Y.
8. Relocate clusters of replicated measurements according to selected CSs associated with true analyte concentration values inside Q:
   a. Obtain the subset $Q = \{\tau_i : l \leq \text{P}(\tau \leq \tau_i) \leq u\}$, where τ_i is observed values of τ, with $\tau \sim \text{uniform}(U_1, U_2)$. Compute the cardinality of Q and denote it by $0 \leq |Q| \leq n$.
   b. If $|Q| = 0$, set $Y_i = 0$ for $i = 1, \ldots, n$ and $D = 0$ and proceed to step nine. If $|Q| > 0$, proceed to sub-step c.
   c. Obtain the set of indices, $I = \{i_1, i_2, \ldots, i_{|Q|}\}$ with $i_j \in \{1, \ldots, n\}$ for all $j \in \{1, \ldots, |Q|\}$.

d. Calculate

$$Y_{i_j} = m_{\max} \cdot \sqrt{\sigma^2_{\text{IVD-MD}_X} + \sigma^2_{\text{IVD-MD}_Y}} \cdot \begin{cases} \dfrac{U_1 + u \cdot (U_2 - U_1) - \tau_{i_j}}{u \cdot (U_2 - U_1)}, & \text{for } l = 0, u > 0 \\ \dfrac{\tau_{i_j} - U_2 + (1 - l) \cdot (U_2 - U_1)}{(1 - l) \cdot (U_2 - U_1)}, & \text{for } l < 1, u = 1 \end{cases}$$

for all $j \in \{1, \ldots, |Q|\}$.

e. Draw D from $1 - 2 \cdot \text{binomial}(1, \ 1/2)$.

9. Add the relocation effects due to systematic DINS to the unaffected measurement results of IVD-MD$_Y$:

$$y_{ir} = y^\star_{ir} + Y_i \cdot D \cdot \mathbb{I}(i \in I); \ i = 1, \ldots, n. \tag{14}$$

10. Calculate $\hat{\sigma}^2_{\text{IVD-MD}_X}$, $\hat{\sigma}^2_{\text{IVD-MD}_Y}$ by using the simulated observed measurements from step seven and nine.
11. Calculate S^2_{P} and $\hat{\beta}_1$, using the OLS regression, with y_{ir} as responses and $x^\star_{ir}$ as predictors.
12. Calculate $\hat{\zeta}$ based on the computed quantities of steps ten and eleven.

2.5 Simulation setting 5: Point-wise prediction interval widths and differences in non-selectivity for homoscedastic measurement errors

Simulation setting 5: Simulates values of $\hat{\zeta}$ with IVD-MDs demonstrating any form of DINS contributing to the average relative increase of the point-wise prediction interval widths, where the IVD-MD results are influenced by homoscedastic measurement errors:

While settings 3 and 4 focused on specific forms of DINS (random and systematic), in simulation setting 5, we shift our attention to the broader relationship between OLS point-wise prediction interval widths and DINS.

Suppose we have two nearly identical IVD-MD comparisons. The only difference between them is the presence of DINS in the second comparison, while DINS is absent in the first. Apart from their difference in DINS, the two IVD-MD comparisons are identical. We estimate point-wise prediction intervals for y_{ir} based on x_{ir} for both IVD-MD comparisons using OLS regression, resulting in two sets of prediction interval widths.

Consider the i-th CS and r-th replicate measurement for both IVD-MD comparisons. The prediction interval lengths for these two nearly identical cases, denoted w_{ir0} and w_{ir}, with respect to these two pairs of measurements, are as follows:

$$w_{ir0} = 2t \cdot S_0 \sqrt{1 + \frac{1}{nR} + \frac{(x_{ir0} - \overline{\overline{x_0}})^2}{S_{XX0}}} \tag{15}$$

$$w_{ir} = 2t \cdot S \sqrt{1 + \frac{1}{nR} + \frac{(x_{ir} - \overline{\overline{x}})^2}{S_{XX}}}. \tag{16}$$

Here, t represents a quantile of the t_{nR-2} distribution (student t-distribution with $nR - 2$ degrees of freedom), determined by the chosen confidence level for the point-wise prediction intervals. The exact value of t is not of interest, because it will cancel later on. Thus, the chosen confidence level is irrelevant. Moreover, S_0 is the mean squared error of OLS regression model fitted by the IVD-MD comparison measurements devoid of DINS. S is the mean squared error of the OLS regression model fitted by the IVD-MD comparison

measurements exhibiting DINS. $\overline{\overline{x_0}}$ and $\overline{\overline{x}}$ are the grand means of the measurements of IVD-MD$_X$ in the two IVD-MD comparisons. Furthermore, we have that

$$S_{XX} = \sum_{i=1}^{n}\sum_{r=1}^{R}\left(x_{ir} - \overline{\overline{x}}\right)^2$$
$$S_{XX0} = \sum_{i=1}^{n}\sum_{r=1}^{R}\left(x_{ir0} - \overline{\overline{x_0}}\right)^2.$$

We can replace S_0 and S with $\hat{\zeta}$, $\hat{\zeta}_0$, $\hat{\sigma}^2_{\text{IVD-MD}_X}$, and $\hat{\sigma}^2_{\text{IVD-MD}_Y}$ by utilizing the definition of $\hat{\zeta}$:

$$w_{ir0} = 2t \cdot \sqrt{\hat{\zeta}_0(\hat{\sigma}^2_{\text{IVD-MD}_Y} + \hat{\beta}_1^2\hat{\sigma}^2_{\text{IVD-MD}_X}) \cdot \frac{nR}{nR+2}}\sqrt{1 + \frac{1}{nR} + \frac{(x_{ir0} - \overline{\overline{x_0}})^2}{S_{XX0}}} \tag{17}$$

$$w_{ir} = 2t \cdot \sqrt{\hat{\zeta}(\hat{\sigma}^2_{\text{IVD-MD}_Y} + \hat{\beta}_1^2\hat{\sigma}^2_{\text{IVD-MD}_X}) \cdot \frac{nR}{nR+2}}\sqrt{1 + \frac{1}{nR} + \frac{(x_{ir} - \overline{\overline{x}})^2}{S_{XX}}}. \tag{18}$$

We now define the ratio of w_{ir} to w_{ir0} as $M_{ir} + 1$, where M_{ir} represents the relative increase in prediction interval length for the prediction interval of y_{ir} due to DINS. After simplifying and canceling some terms, we obtain the following expression:

$$M_{ir} + 1 = \sqrt{\frac{\hat{\zeta}}{\hat{\zeta}_0}} \cdot \frac{\sqrt{1 + \frac{1}{nR} + \frac{(x_{ir} - \overline{\overline{x}})^2}{S_{XX}}}}{\sqrt{1 + \frac{1}{nR} + \frac{(x_{ir0} - \overline{\overline{x_0}})^2}{S_{XX0}}}} \tag{19}$$

Taking the average on both sides of the equation over all i and r, we derive that

$$M + 1 = \frac{1}{nR} \cdot \sqrt{\frac{\hat{\zeta}}{\hat{\zeta}_0}} \cdot \sum_{i=1}^{n}\sum_{r=1}^{R} \frac{\sqrt{1 + \frac{1}{nR} + \frac{(x_{ir} - \overline{\overline{x}})^2}{S_{XX}}}}{\sqrt{1 + \frac{1}{nR} + \frac{(x_{ir0} - \overline{\overline{x}})^2}{S_{XX0}}}} \tag{20}$$

provided that M is the mean of all individual M_{ir}. We define

$$\psi = \frac{1}{nR} \sum_{i=1}^{n}\sum_{r=1}^{R} \frac{\sqrt{1 + \frac{1}{nR} + \frac{(x_{ir} - \overline{\overline{x}})^2}{S_{XX}}}}{\sqrt{1 + \frac{1}{nR} + \frac{(x_{ir0} - \overline{\overline{x_0}})^2}{S_{XX0}}}}, \tag{21}$$

as a random variable when at least one pair (i, r) satisfies $x_{ir0} \neq x_{ir}$. If no such pair exists, all terms in the double sum become 1, leading to $\psi = 1$. By squaring both sides of the equation and performing some algebraic manipulations, we derive the closed-form relationship between M, $\hat{\zeta}_0$, and $\hat{\zeta}$:

$$\hat{\zeta} = \Big[\psi(M + 1)\Big]^2 \cdot \hat{\zeta}_0 \tag{22}$$

In this article, we will assume that $\psi = 1$, which implies that $x_{ir0} = x_{ir}$ for all $i = 1, \ldots, n$ and $r = 1, \ldots, R$. The general expression for $\hat{\zeta}$ based on M and $\psi \neq 1$ may be explored in a future article. Under the assumption of $\psi = 1$, we simplify the closed-form relationship to obtain the following expression:

$$\hat{\zeta} = (1 + M)^2\hat{\zeta}_0. \tag{23}$$

This relationship proves useful because the average relative increase in the prediction interval serves as an intuitive and general way to quantify the magnitude of DINS without having to manage multiple parameters, as required in simulation settings 3 and 4. Additionally, we can utilize the conditional distribution of $\hat{\zeta}$ given M and $\hat{\zeta}_0$. By fixing M, the distribution of $\hat{\zeta}$ can be easily simulated since $\hat{\zeta}_0$ is straightforward to simulate. Consequently, we can use this formula to construct rejection regions of $\hat{\zeta}$ that depend solely on M, n, and R, simplifying the DINS problem to the selection of an appropriate value for M. Keep in mind that M can be represented as either a percentage or a decimal number. For instance, when $M = 0.3$, its equivalent percentage form is 30%. To distinguish between the two, percentage values of M are usually denoted as $M(\%)$.

We can then simulate $\hat{\zeta}$ values affected by DINS between compared IVD-MDs by choosing $M > 0$. The algorithm defining the fifth simulation setting is:

1. Draw n from a truncated Poisson distribution with limits at 20 and 30:

$$n \sim \max\Big[20, \min\big[\text{Poisson}(25), 30\big]\Big].$$

2. Draw R from the set $\{2, 3, 4\}$ with point mass probabilities $(2/20, 17/20, 1/20)$, accounting for the commonality of three replicates over two or four.
3. Draw $\text{CV}_{\text{IVD-MD}_X}$, $\text{CV}_{\text{IVD-MD}_Y}$ from a beta distribution scaled by a factor of $1/10$:

$$\text{CV}_{\text{IVD-MD}_X},\ \text{CV}_{\text{IVD-MD}_Y} \sim \text{Beta}(2, 5)/10.$$

4. Draw the lower limit of the concentration interval, U_1, from an F-distribution scaled by 44:

$$U_1 \sim F_{1.06, 8.15} \cdot 44.$$

   Subsequently, determine T from a beta distribution (also scaled by 44) and compute the concentration interval upper limit, U_2, as:

$$T \sim \text{Beta}(0.78, 11) \cdot 44 \qquad\qquad U_2 = U_1(1 + T).$$

5. For each CS, draw latent concentration values, τ_i, uniformly within the defined concentration interval:

$$\tau_i \sim \text{Uniform}(U_1, U_2),\ \text{for } i = 1, \ldots, n.$$

6. Derive IVD-MD standard deviations $\sigma_{\text{IVD-MD}_X}$ and $\sigma_{\text{IVD-MD}_Y}$ using the coefficients of variation, $\text{CV}_{\text{IVD-MD}_X}$ and $\text{CV}_{\text{IVD-MD}_Y}$, and the mid-point of the concentration interval:

$$\sigma_{\text{IVD-MD}_X} = \text{CV}_{\text{IVD-MD}_X} \cdot \frac{1}{2}[U_1 + U_2]$$
$$\sigma_{\text{IVD-MD}_Y} = \text{CV}_{\text{IVD-MD}_Y} \cdot \frac{1}{2}[U_1 + U_2].$$

7. Simulate observed IVD-MD measurements, x_{ir0} and y_{ir0}, by adding measurements errors to τ_i:

$$x_{ir0} = \tau_i + \mathrm{N}(0, \sigma^2_{\text{IVD-MD}_X})_r;\; r = 1, \ldots R$$
$$y_{ir0} = \tau_i + \mathrm{N}(0, \sigma^2_{\text{IVD-MD}_Y})_r;\; r = 1, \ldots R.$$

The simulated values x_{ir0} represents measurements based on IVD-MD$_X$, and y_{ir0} signify measurements from IVD-MD$_Y$.

8. Calculate $\hat{\sigma}^2_{\text{IVD-MD}_X}$ and $\hat{\sigma}^2_{\text{IVD-MD}_Y}$ from the simulated observed measurements from step seven.
9. Calculate S^2_{P} and $\hat{\beta}_1$, using the OLS regression, with y_{ir0} as responses and x_{ir0} as predictors.
10. Calculate $\hat{\zeta}_0$ using the outputs from steps eight and nine.
11. Calculate $\hat{\zeta}$ using $\hat{\zeta}_0$ from step ten and $M \geq 0$:

$$\hat{\zeta} = (1 + M)^2 \cdot \hat{\zeta}_0 \tag{24}$$

2.6 Principle figures for the different simulation settings

For a comprehensive understanding of the first four simulation settings, it is imperative to visualize the corresponding simulated data. This objective is accomplished by presenting 12 scatter plots, depicting three examples for each of the four simulation settings. These three examples are labeled as a, b, and c. In example a, the IVD-MD CVs are 1% and 3%, while in example b, the IVD-MD CVs are established at 3% and 2%, and in example c, both CVs are uniformly set at 1%. The concentration interval, which is the same across all three examples, ranges between 5 and 10 units ($U_1 = 5$ and $U_2 = 10$). Moreover, each simulation engenders 100 CSs ($n = 100$), wherein each CS encompasses three replicated measurements ($R = 3$). For a detailed breakdown of the remaining parameters used in these examples for each simulation setting, one is directed to consult Table 1.

Figure 1 presents the simulated data for the three examples across all four simulation settings. Within this figure, the topmost row of scatter showcases examples a, b and c associated with simulation setting 1. The subsequent rows, in order, display scatter plots for examples a, b, and c related to simulation settings 2, 3, and 4 respectively.

3 Simulation parameters

We are interested in performing six sets of simulations. The first two use simulation setting 1. Simulation sets, three, four, five and six uses simulation settings 2, 3, 4 and 5.

3.1 Simulation parameters for the first set of simulations

In the first set of simulations we are simulating $\hat{\zeta}$ values, based on simulating setting 1, with all combinations of

1. Fixed concentration intervals:
   $\{U_1 = 5, U_2 = 10\}$, $\{U_1 = 2, U_2 = 10\}$, $\{U_1 = 500, U_2 = 750\}$ $\{U_1 = 70, U_2 = 1600\}$.
2. Fixed IVD-MD CVs for $\mathrm{CV}_{\text{IVD-MD}_X}$ and $\mathrm{CV}_{\text{IVD-MD}_Y}$:
   0.1%, 0.5%, 1.0%, 2.5%, 5.0%, 7.5%, and 10%.

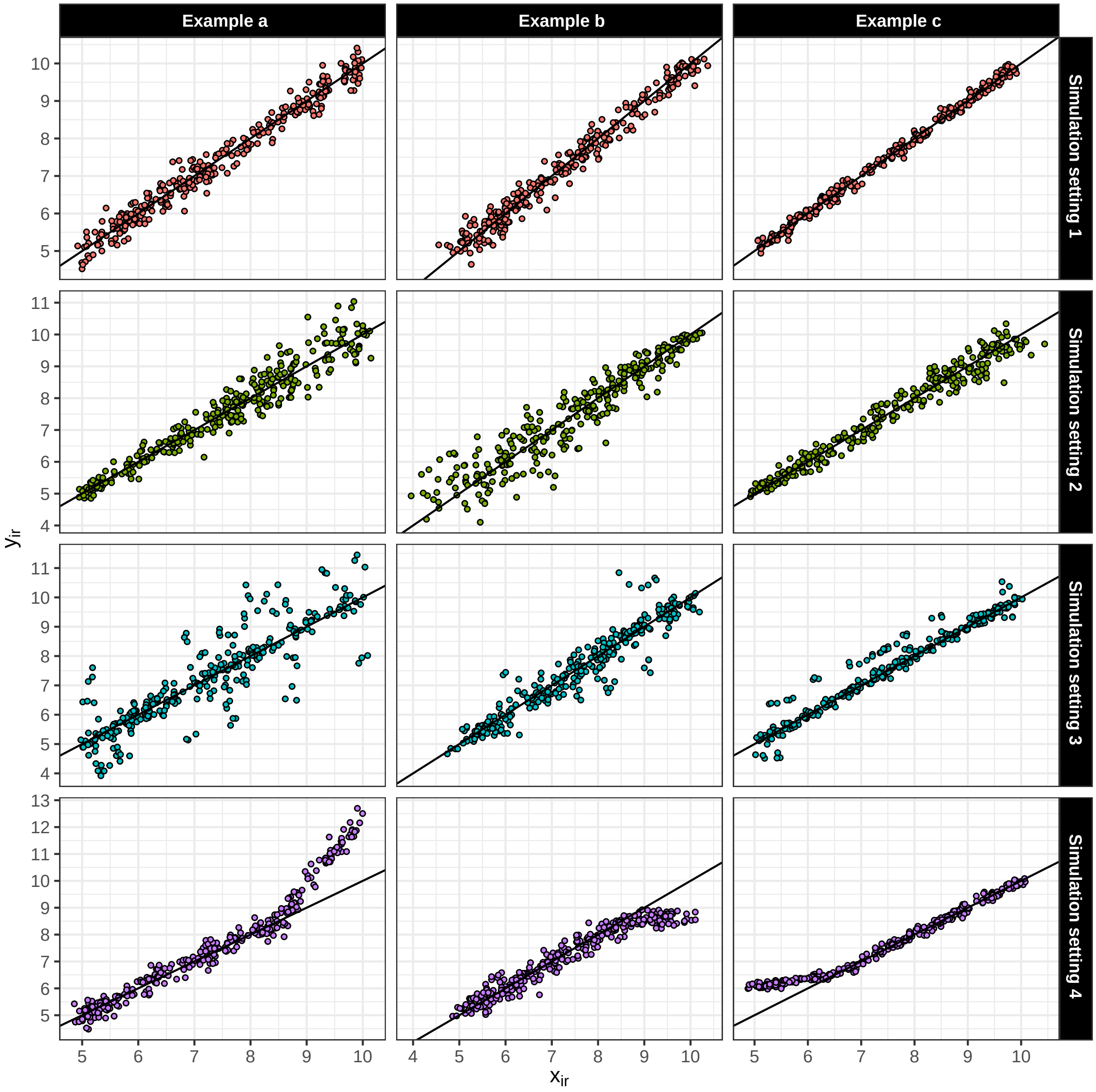

Figure 1. Examples of simulated data from simulation settings 1, 2, 3, and 4. Example a had in vitro diagnostic medical device coefficients of variation that measured 1% and 3%, example b 3% and 2% and example c both 1%. 100 clinical samples with three replicated measurements are simulated for each example within each simulation setting. The remaining simulation parameter specifications are listed in Table 1. The black lines refers to the lines of equivalence: lines with an intercept equal to 0 and a slope equal to 1.

Table 1. Simulation parameters used for each simulation setting and for each of the examples, a, b and c. The nine first cell values of q are missing because q has quantile range equal to 0 for the three first simulation settings.

Simulation setting	Example	η_0	η	p	$m_{\max}$	q
1	a	1.0	1.0	0.0	0	
1	b	1.0	1.0	0.0	0	
1	c	1.0	1.0	0.0	0	
2	a	0.5	5.0	0.0	0	
2	b	3.0	0.2	0.0	0	
2	c	1.0	4.0	0.0	0	
3	a	1.0	1.0	0.3	10	
3	b	1.0	1.0	0.1	10	
3	c	1.0	1.0	0.2	10	
4	a	1.0	1.0	0.0	10	[0.7, 1.0]
4	b	1.0	1.0	0.0	5	[0.7, 1.0]
4	c	1.0	1.0	0.0	10	[0, 0.3]

One million $\hat{\zeta}$ values are sampled for every combination of simulation parameters. There are $4 \cdot 7 \cdot 7 = 196$ unique combinations of simulation parameters, which means that 196 million $\hat{\zeta}$ values are simulated in total. The steps three and four of simulation setting 1 are skipped because the IVD-MD CVs and concentration intervals are fixed.

3.2 Simulation parameters in the second set of simulations

In the second set of simulations we are simulating $\hat{\zeta}$ values, based on simulation setting 1, with all combinations of

1. Fixed number of clinical samples: 20, 25, 30, 35, 40
2. Fixed Number of replicated measurements: 2, 3, 4

One million $\hat{\zeta}$ values are sampled for every combination of simulation parameters. There are $5 \cdot 3 = 15$ unique combinations of simulation parameters, which means that 15 million $\hat{\zeta}$ values are simulated in total. The two first steps of simulation setting 1 are skipped because n and R are fixed.

3.3 Simulation parameters in the third set of simulations

In the third set of simulations we are simulating $\hat{\zeta}$ values, based on simulation setting 2, utilizing all combinations of

1. Fixed number of clinical samples: 20, 25, 30, 35, 40
2. Fixed number of replicated measurements: 2, 3, 4
3. Heteroscedasticity factors, η: $1/10$, $1/4$, $1/2$, $3/4$, 1, $4/3$, 2, 4, 6, 10
4. Multiplier of base IVD-MD standard deviations: $\eta_0 = 1$

One million $\hat{\zeta}$ values are sampled for every combination of simulation parameters. There are $5 \cdot 3 \cdot 10 = 150$ unique combinations of simulation parameters, which means that 150 million $\hat{\zeta}$ values are simulated for these simulations. The two first steps of simulation setting 2 are skipped because n and R are fixed.

3.4 Simulation parameters in the fourth set of simulations

In the fourth set of simulations we are simulating measurements from two IVD-MDs in a comparison, having random DINS. This implies that we will simulate $\hat{\zeta}$ values based on simulation setting 3. We simulate values of $\hat{\zeta}$ using all combinations of

1. Fixed number of clinical samples: 20, 25, 30, 35, 40
2. Fixed number of replicated measurements: 2, 3, 4
3. Average proportion of affected CSs: 0.05, 0.10, 0.15, 0.20, 0.25, 0.30
4. Maximal relocation magnitudes: 0, 1, 2, 3, 5, 7.5, 10

One million $\hat{\zeta}$ values are sampled for every combination of simulation parameters. There are $5 \cdot 3 \cdot 6 \cdot 7 = 630$ unique combinations of simulation parameters, which means that 630 million $\hat{\zeta}$ values are simulated in this simulation setting. The two first steps of simulation setting 3 are skipped because n and R are fixed.

3.5 Simulation parameters in the fifth set of simulations

In the fourth set of simulations we are simulating measurements from two IVD-MDs in a comparison, that having systematic DINS. This implies that we will simulate $\hat{\zeta}$ values based on simulation setting 4. We simulate values of $\hat{\zeta}$ using all combinations of

1. Fixed number of clinical samples: 20, 25, 30, 35, 40
2. Fixed number of replicated measurements: 2, 3, 4
3. quantile intervals: (0, 0.05), (0, 0.10), (0, 0.15), (0, 0.20), (0, 0.25), (0, 0.30), (0, 0.95), (0.90, 1), (0.85, 1), (0.80, 1), (0.75, 1), (0.70, 1)
4. Maximal relocation magnitudes: 0, 1, 2, 3, 5, 7.5, 10

One million $\hat{\zeta}$ values are sampled for every combination of simulation parameters. There are $5 \cdot 3 \cdot 12 \cdot 7 = 1260$ unique combinations of simulation parameters, which means that 1.26 billion $\hat{\zeta}$ values are simulated in this simulation setting. The two first steps of simulation setting 4 are skipped because n and R are fixed.

3.6 Simulation parameters in the sixth set of simulations

In the sixth set of simulations, we are simulating $\hat{\zeta}$ values, based on simulation setting 5, which describes the more general approach to simulate $\hat{\zeta}$ values corresponding with effects caused by DINS. We simulate values of $\hat{\zeta}$ using all combinations of:

1. Fixed number of clinical samples: 20, 25, 30, 35, 40
2. Fixed number of replicated measurements: 2, 3, 4
3. M values: 0, 0.05, 0.10, 0.15, ..., 0.95, 1, 1.05, ..., 1.95, 2

One million $\hat{\zeta}$ values are sampled for every combinations of simulation parameters. There are $5 \cdot 3 \cdot 41 = 615$ unique combinations of simulation parameters, which means that 615 million $\hat{\zeta}$ values are simulated in this simulation setting. The two first steps of the initial four simulation steps are skipped because n and R are fixed.

4 Simulation results

The first four moments of a random variable are the *mean*, *variance*, *skewness*, and *kurtosis*. In combination with the inverse cumulative distribution function (percentiles), these moments provide a sufficient description of the distribution of $\hat{\zeta}$. Readers are assumed to be familiar with the first two moments, mean and variance, as well as percentiles. Skewness is an asymmetry statistic of a random variable, indicating whether its distribution is left-skewed or right-skewed. The magnitude of the skewness statistic reflects the degree of the asymmetry. On the other hand, kurtosis measures the thickness of the distribution's tails, indicating the potential presence of outliers. The mean, variance, skewness and kurtosis of $\hat{\zeta}$ are estimated by replicating $\hat{\zeta}$ sufficiently many times and the resulting set of replications are used to approximate these moments. Let $\{\hat{\zeta}_1, \ldots, \hat{\zeta}_N\}$ denote the set of N simulated $\hat{\zeta}$ values predicated upon a set of simulation parameters. Then, we use the following sample statistics to estimate the mean, variance, skewness and kurtosis, respectively:

$$\begin{aligned}
\overline{\hat{\zeta}} &= \frac{1}{N}\sum_{i=1}^{N}\hat{\zeta}_i \\
S^2_{\hat{\zeta}} &= \frac{1}{N-1}\sum_{i=1}^{N}(\hat{\zeta}_i - \overline{\hat{\zeta}})^2 \\
\gamma_{\hat{\zeta}} &= \frac{\frac{1}{N}\sum_{i=1}^{N}(\hat{\zeta}_i - \overline{\hat{\zeta}})^3}{[\frac{N-1}{N}S^2_{\hat{\zeta}}]^{3/2}} \\
K_{\hat{\zeta}} &= \frac{\frac{1}{N}\sum_{i=1}^{N}(\hat{\zeta}_i - \overline{\hat{\zeta}})^4}{[\frac{N-1}{N}S^2_{\hat{\zeta}}]^2}.
\end{aligned}$$

It is well known that $\overline{\hat{\zeta}}$ and $S^2_{\hat{\zeta}}$ are unbiased estimators of the true population moments, $\mathrm{E}[\hat{\zeta}]$ and $\mathrm{Var}[\hat{\zeta}]$, respectively. However, the skewness and kurtosis of $\hat{\zeta}$ are only unbiased of their corresponding population moments if $\hat{\zeta}$ is normally distributed. Based on the definition of $\hat{\zeta}$, it is not normal, so the skewness and kurtosis of $\hat{\zeta}$ will be biased estimators of the population skewness and kurtosis. However, they will still be documented in the simulation results, as they convey general information regarding the distribution of $\hat{\zeta}$.

Suppose that we want to obtain the $P\%$ percentile of $\hat{\zeta}$. Given that $\tilde{p} = P\%/100\%$, this percentile is per definition

$$P\% = \inf\{x \in \mathbb{R}^+ : \tilde{p} \leq \mathrm{P}(\hat{\zeta} \leq x)\}.$$

We can estimate the $P\%$ percentile of $\hat{\zeta}$ by again assuming N replications of $\hat{\zeta}$, $\{\hat{\zeta}_1, \ldots, \hat{\zeta}_N\}$, and use these to calculate the P-th percentile of $\hat{\zeta}$ in the following way:

$$P\% = (1-h)\hat{\zeta}_{(j)} + h\hat{\zeta}_{(j)};\ h = \tilde{p}(N-1) - (j-1).$$

Here, $\hat{\zeta}_{(j)}$ is the j-th smallest value among $\{\hat{\zeta}_1, \ldots, \hat{\zeta}_N\}$. Moreover, j is chosen so that

$$\begin{aligned}
\frac{j-1}{N-1} &< \tilde{p} \\
\frac{j}{N-1} &> \tilde{p}.
\end{aligned}$$

4.1 Simulation results: first set of simulations

In the first set of simulations, the exact values of the moments and percentiles of $\hat{\zeta}$ are not of primary concern, as the aim is to demonstrate the absence of a considerable relationship between $\hat{\zeta}$ and the IVD-MD CVs and concentration intervals. However, in the subsequent simulations, the specific values of these moments and percentiles become important.

Table 2. The 1%, 25%, 50%, 75% and 99% moment percentiles of the differences in non-selectivity estimator, $\hat{\zeta}$ for each of the four concentration intervals, within the three in vitro diagnostic medical device coefficients of variation groupings.

Grouping	U_1	U_2	Moment percentiles (1% - 25% - 50% - 75% - 99%)
**Mean**			
All	5	10	1.037 - 1.043 - 1.044 - 1.046 - 1.106
All	2	10	1.036 - 1.042 - 1.044 - 1.045 - 1.058
All	500	750	1.036 - 1.043 - 1.045 - 1.047 - 1.168
All	70	1600	1.036 - 1.042 - 1.044 - 1.046 - 1.05
$\lambda > 2$ or $\lambda < 1/2$	5	10	1.037 - 1.043 - 1.044 - 1.046 - 1.062
$\lambda > 2$ or $\lambda < 1/2$	2	10	1.038 - 1.043 - 1.044 - 1.045 - 1.048
$\lambda > 2$ or $\lambda < 1/2$	500	750	1.04 - 1.043 - 1.045 - 1.046 - 1.09
$\lambda > 2$ or $\lambda < 1/2$	70	1600	1.038 - 1.042 - 1.044 - 1.045 - 1.048
Both IVD-MD CVs < 5%	5	10	1.036 - 1.04 - 1.042 - 1.044 - 1.046
Both IVD-MD CVs < 5%	2	10	1.036 - 1.039 - 1.042 - 1.044 - 1.047
Both IVD-MD CVs < 5%	500	750	1.036 - 1.04 - 1.043 - 1.045 - 1.055
Both IVD-MD CVs < 5%	70	1600	1.036 - 1.038 - 1.043 - 1.044 - 1.047
**Variance**			
All	5	10	0.019 - 0.02 - 0.021 - 0.024 - 0.031
All	2	10	0.019 - 0.02 - 0.021 - 0.023 - 0.027
All	500	750	0.019 - 0.02 - 0.021 - 0.025 - 0.037
All	70	1600	0.019 - 0.02 - 0.021 - 0.024 - 0.026
$\lambda > 2$ or $\lambda < 1/2$	5	10	0.019 - 0.02 - 0.02 - 0.022 - 0.026
$\lambda > 2$ or $\lambda < 1/2$	2	10	0.019 - 0.02 - 0.021 - 0.021 - 0.025
$\lambda > 2$ or $\lambda < 1/2$	500	750	0.019 - 0.02 - 0.021 - 0.022 - 0.029
$\lambda > 2$ or $\lambda < 1/2$	70	1600	0.019 - 0.02 - 0.021 - 0.022 - 0.024
Both IVD-MD CVs < 5%	5	10	0.02 - 0.02 - 0.021 - 0.024 - 0.026
Both IVD-MD CVs < 5%	2	10	0.019 - 0.02 - 0.021 - 0.023 - 0.026
Both IVD-MD CVs < 5%	500	750	0.019 - 0.02 - 0.022 - 0.024 - 0.026
Both IVD-MD CVs < 5%	70	1600	0.019 - 0.021 - 0.021 - 0.024 - 0.025
**Skewness**			
All	5	10	0.487 - 0.736 - 1.336 - 1.501 - 1.832
All	2	10	0.528 - 0.73 - 1.317 - 1.55 - 1.856
All	500	750	0.459 - 0.831 - 1.296 - 1.466 - 1.763
All	70	1600	0.466 - 0.721 - 1.309 - 1.529 - 1.825
$\lambda > 2$ or $\lambda < 1/2$	5	10	0.645 - 1.042 - 1.377 - 1.527 - 1.836

$\lambda > 2$ or $\lambda < 1/2$	2	10	0.656 - 1.018 - 1.406 - 1.584 - 1.857
$\lambda > 2$ or $\lambda < 1/2$	500	750	0.686 - 1.054 - 1.368 - 1.476 - 1.767
$\lambda > 2$ or $\lambda < 1/2$	70	1600	0.654 - 0.989 - 1.384 - 1.549 - 1.826
Both IVD-MD CVs < 5%	5	10	0.525 - 0.702 - 1.153 - 1.463 - 1.843
Both IVD-MD CVs < 5%	2	10	0.524 - 0.696 - 1.087 - 1.421 - 1.549
Both IVD-MD CVs < 5%	500	750	0.469 - 0.677 - 1.092 - 1.429 - 1.6
Both IVD-MD CVs < 5%	70	1600	0.554 - 0.691 - 1.13 - 1.477 - 1.548
**Kurtosis**			
All	5	10	3.879 - 4.945 - 7.203 - 9.046 - 13.447
All	2	10	4.018 - 4.835 - 7.457 - 9.262 - 14.275
All	500	750	3.957 - 5.318 - 7.106 - 8.366 - 13.168
All	70	1600	4.048 - 4.71 - 7.352 - 9.331 - 12.885
$\lambda > 2$ or $\lambda < 1/2$	5	10	4.367 - 6.417 - 7.812 - 9.104 - 13.561
$\lambda > 2$ or $\lambda < 1/2$	2	10	4.521 - 6.262 - 8.165 - 9.802 - 14.399
$\lambda > 2$ or $\lambda < 1/2$	500	750	4.406 - 6.474 - 7.526 - 8.992 - 13.239
$\lambda > 2$ or $\lambda < 1/2$	70	1600	4.472 - 6.05 - 8.185 - 10.245 - 12.931
Both IVD-MD CVs < 5%	5	10	3.948 - 4.916 - 6.957 - 9.084 - 12.786
Both IVD-MD CVs < 5%	2	10	3.925 - 4.833 - 6.713 - 8.163 - 9.607
Both IVD-MD CVs < 5%	500	750	4.046 - 4.604 - 6.297 - 8.31 - 10.487
Both IVD-MD CVs < 5%	70	1600	4.288 - 4.665 - 6.671 - 8.363 - 11.365

The violin plots presented in Figure 2**A** suggests that the first four moments of $\hat{\zeta}$ are generally unaffected by concentration intervals. This observation stems from the overlapping of the black points atop the violin plots across the four concentration intervals under consideration. The red points represent the union of simulation parameter combinations associated with the ten most deviating moment values for each of the first four moments (40 in total). There are currently 25 such unique simulation parameter combinations. Generally, the simulation parameter combinations with the most deviating mean and variance values are what causing slight variations in the empirical moment distributions across the four regarded concentration intervals. From the scatter plots in Figure 2**B**, it is evident that the red points are generally associated with

$$\lambda = \frac{\sigma^2_{\text{IVD-MD}_Y}}{\sigma^2_{\text{IVD-MD}_X}} = \frac{\text{CV}^2_{\text{IVD-MD}_Y}}{\text{CV}^2_{\text{IVD-MD}_X}}$$

being close to one, zero, or a large value. We infer that $\frac{4}{9} \leq \lambda \leq \frac{9}{4}$ produces the most extreme mean and variance values, whereas either $\lambda \leq \frac{1}{100}$ or $\lambda \geq \frac{225}{4}$, correspond to the most extreme skewness and kurtosis values. Nevertheless, λ is not the sole factor; the actual values of $\text{CV}_{\text{IVD-MD}_X}$ and $\text{CV}_{\text{IVD-MD}_Y}$ contribute as well. In detail, large IVD-MD CVs ($\geq 5\%$) are usually associated with larger mean and variance of $\hat{\zeta}$, but this is only problematic when $\frac{4}{9} \leq \lambda \leq \frac{9}{4}$. Conversely, when one IVD-MD CV is large and the other is small, it results in increased skewness and kurtosis of $\hat{\zeta}$. However, this does not contribute to the slight variations of the moment distributions across the regarded concentration intervals. In essence, if λ is proximate to 1 and both IVD-MD CVs are at least 5%, the mean and variance of $\hat{\zeta}$ are slightly influenced by the concentration interval. In such scenarios, concentration intervals with U_2/U_1 close to 1 are problematic.

The implications of these findings are evident in the the empirical CDFs of $\hat{\zeta}$ shown in Figure 3**A**. Notably, for

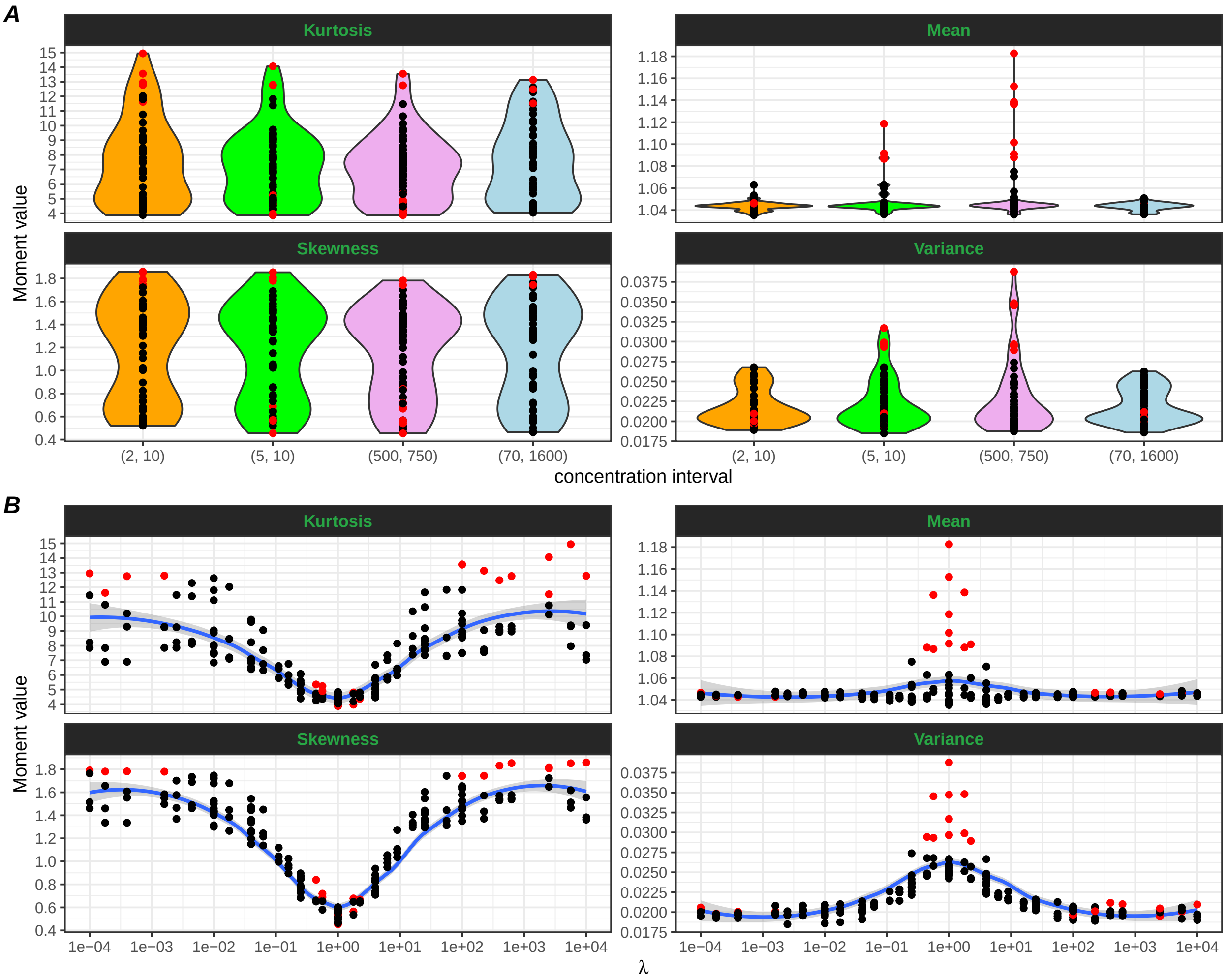

Figure 2. **A**: Violin plots showcasing the distribution of the mean, variance, skewness, and kurtosis of the differences in non-selectivity estimator, $\hat{\zeta}$, for each of the four considered concentration intervals over all pairs of in vitro diagnostic medical device (IVD-MD) coefficents of variation. **B**: Scatter plots illustrating the relationship between the mean, variance, skewness, and kurtosis of $\hat{\zeta}$ and the ratio of IVD-MD variances (λ). The red points (there are 25 of them) found in panels **A** and **B**, signify the union of simulation parameter combinations associated with the ten largest mean, variance, skewness and kurtosis values of $\hat{\zeta}$.

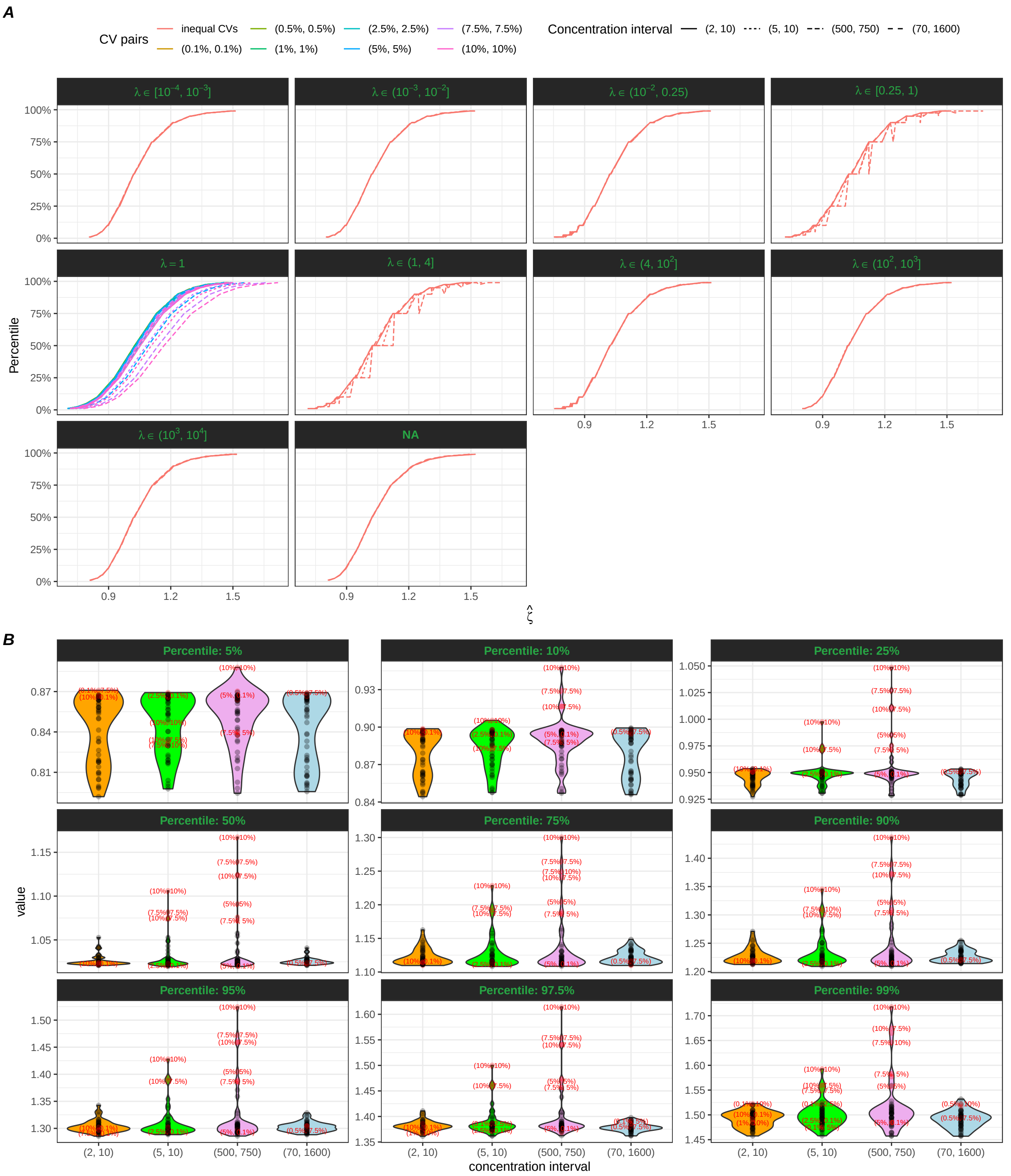

Figure 3. **A**: Empirical CDFs for the differences in non-selectivity estimator, $\hat{\zeta}$, across all considered coefficient of variation (CV) pairs, concentration intervals, and nine sub-intervals of the domain of the ratio of in vitro diagnostic medical device (IVD-MD) variances (λ). **B**: Violin plots for the 5%, 10%, 25%, 50%, 75%, 90%, 95%, 97.5% and 99% percentiles of $\hat{\zeta}$ for each of the four considered concentration intervals. The red points signify the percentile values, for each concentration interval, associated with the IVD-MD CV pairs resulting in an extreme value for at least one of the first four moments of $\hat{\zeta}$. The red text, layered above the red points, reveals the IVD-MD CV pair corresponding to each red point.

the $\lambda < \frac{1}{4}$ and $\lambda > 4$ panels, the CDFs of $\hat{\zeta}$ coincide for all four concentration intervals and all combinations of IVD-MD CV magnitudes. For $\frac{1}{4} \leq \lambda \leq 4$, minor discrepancies exists in the the CDFs concenrning concentration intervals and the magnitude of IVD-MD CVs. In the $\lambda = 1$ panel, less steep CDF curves align with larger IVD-MD CV magnitudes. This trend is also seen for the two adjacent panels, where larger IVD-MD CVs contribute to non-overlapping CDF curves. In Figure 3**B**, the empirical distributions of the 5%, 10%, 25%, 50%, 75%, 90%, 95%, 97.5% and 99% percentiles are examined. As noted in 2**A**, differences in the regarded percentiles of $\hat{\zeta}$ between concentration intervals are only noticeable when both IVD-MD CVs are large and λ proximate 1.

To summarize, $\hat{\zeta}$ is independent of both the concentration interval and IVD-MD CVs if either $\frac{4}{9} \leq \lambda \leq \frac{9}{4}$ with both IVD-MD CVs being smaller than 5%, or if $\lambda < \frac{4}{9}$ or $\lambda > \frac{9}{4}$, irrespective of the magnitudes of the IVD-MD CV magnitudes. Thus, if both IVD-MD CVs in a comparison are smaller than 5%, $\hat{\zeta}$ will be independent of both the concentration interval and IVD-MD CVs, independently of λ. However, the practical implications of IVD-MD CVs being at 10% are generally minimal. This means that $\hat{\zeta}$ is largely unaffected by both concentration interval and IVD-MD CVs if the IVD-MD CVs are less than or equal to 10%. However, it is important to note that if both IVD-MD CVs in a IVD-MD comparison approach 10% and the ratio U_2/U_1 is close to 1, the mean, variance, and percentiles of $\hat{\zeta}$ might be marginally larger than when U_2/U_1 is large.

4.2 Simulation results: second set of simulations

In the second set of simulations, we investigate the correlation between *study design* and the distribution of $\hat{\zeta}$ when compared IVD-MDs are devoid of DINS. Study design is defined as the number of CSs (n) and the number of replicates (R). We anticipate that smaller study designs will result in greater uncertainty of $\hat{\zeta}$, such as larger variance, compared to larger study designs. The mean of $\hat{\zeta}$ is expected to be close to 1 for all study designs. The mean of $\hat{\zeta}$ is also expected to converge to 1 as $nR \to \infty$. Thus, larger study designs will result in a mean value of $\hat{\zeta}$ closer to 1 compared to smaller study designs.

Table 3. Distribution summary statistics for the mean, variance, skewness and kurtosis of the differences in non-selectivity estimator, $\hat{\zeta}$, across all 15 combinations of the number of clinical samples (n) and replicated measurements (R). 'MSD' is short for moment study design.

Moment	Minimum	First quartile	Median	Third quartile	Maximum	Minimizing MSD	Maximizing MSD
mean	1.025	1.034	1.040	1.056	1.089	{n = 40, R = 4}	{n = 20, R = 2}
variance	0.008	0.013	0.017	0.033	0.069	{n = 40, R = 4}	{n = 20, R = 2}
skewness	0.407	0.479	0.529	0.785	1.310	{n = 40, R = 4}	{n = 20, R = 2}
kurtosis	3.587	3.661	3.863	4.310	8.398	{n = 25, R = 3}	{n = 20, R = 2}

Table 4. Distribution summary statistics for various $\hat{\zeta}$ (differences in non-selectivity estimator) percentiles across all 15 combinations of the number of clinical samples (n) and replicated measurements (R). 'PSD' is short for percentile study design.

Percentile	Minimum	First quartile	Median	Third quartile	Maximum	Minimizing PSD	Maximizing PSD
1%	0.645	0.721	0.772	0.801	0.834	{n = 20, R = 2}	{n = 40, R = 4}
2.5%	0.699	0.762	0.815	0.835	0.866	{n = 20, R = 2}	{n = 40, R = 4}
5%	0.743	0.799	0.847	0.865	0.890	{n = 20, R = 2}	{n = 40, R = 4}
10%	0.800	0.846	0.884	0.899	0.919	{n = 20, R = 2}	{n = 40, R = 4}
25%	0.909	0.927	0.949	0.958	0.965	{n = 20, R = 2}	{n = 40, R = 4}
**50%**	**1.019**	**1.025**	**1.031**	**1.036**	**1.050**	**{n = 35, R = 4}**	**{n = 20, R = 2}**
75%	1.079	1.103	1.116	1.160	1.224	{n = 40, R = 4}	{n = 20, R = 2}
90%	1.140	1.180	1.210	1.291	1.421	{n = 40, R = 4}	{n = 20, R = 2}
95%	1.179	1.231	1.268	1.382	1.561	{n = 40, R = 4}	{n = 20, R = 2}
97.5%	1.220	1.278	1.327	1.471	1.709	{n = 40, R = 4}	{n = 20, R = 2}
99%	1.263	1.345	1.392	1.580	1.922	{n = 40, R = 4}	{n = 20, R = 2}

By examining Table 3, we observe that the mean of $\hat{\zeta}$ falls within the range of 1.024 and 1.094, while the variance lies between 0.008 and 0.069. Further, the skewness ranges from 0.427 to 1.130, and the kurtosis is between 3.649 and 5.832. The maximum values for all four moments are associated with the smallest study design considered, where $n = 20$ and $R = 2$. Conversely, the minimum mean, variance and skewness values for $\hat{\zeta}$ are all linked to the largest considered study design, with $n = 40$ and $R = 4$. However, the minimum kurtosis value for $\hat{\zeta}$ correspond to $n = 40$ and $R = 3$. As we see in Figure 4**A**, a systematic relationship exists between the first four moments of $\hat{\zeta}$ and the product $n \cdot R$. Specifically, as the product $n \cdot R$ increases, the four moments tend to decrease. In this figure, the numbers represent the number of CSs (n), while the color of the numbers denotes the number of replicated measurements. In general, we observe that increasing R by 1 is more effective in reducing the mean, variance, skewness, and kurtosis of $\hat{\zeta}$ compared to increasing n by 5.

Table 4 illustrates that, the largest 1%, 2.5%, 5%, 10% and 25% percentile values of $\hat{\zeta}$ are associated with the largest study design ($n = 40$ and $R = 4$), while the smallest 1%, 2.5%, 5%, 10% and 25% percentiles are related to the smallest study design ($n = 20$ and $R = 2$). On the other hand, the largest 50%, 75%, 90%, 95%, 97.5% and 99% percentile values of $\hat{\zeta}$ correspond to the smallest study design and the smallest 50%, 75%, 90%, 95%, 97.5% and 99% percentile values to the largest study design. These relationships between the percentiles and the study designs result from the relationship between the first four moments of $\hat{\zeta}$ and the study designs: Larger study designs decrease variance, causing $\hat{\zeta}$ values to approach the mean of $\hat{\zeta}$, thus increasing the percentile values of 1%, 2.5%, 5%, 10% and 25% and decreasing percentile values of 50%, 75%, 90%, 95%, 97.5% and 99%.

Similar to the first four moments of $\hat{\zeta}$ and the product $n \cdot R$, a systematic relationship exists between the percentiles of $\hat{\zeta}$ and the product $n \cdot R$. Indeed, Figure 4**B** elucidates that 1%, 2.5%, 5%, 10% and 25%

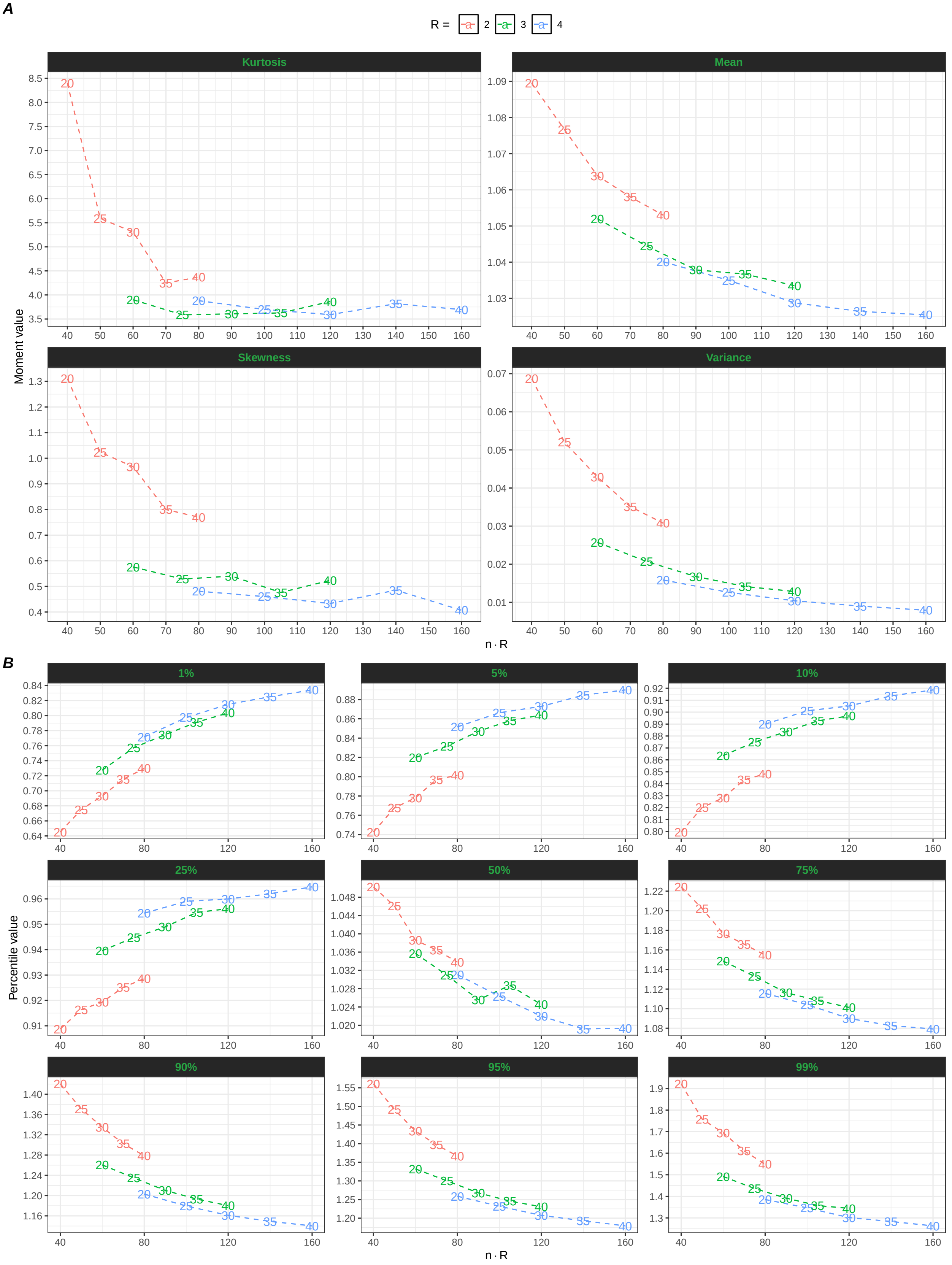

Figure 4. **A**: The mean, variance, skewness and kurtosis of the differences in non-selectivity estimator, $\hat{\zeta}$, for all combinations of $20, 25, 30, 35, 40$ clinical samples and $2, 3, 4$ measurement replicates (15 study designs in total). **B**: The 1%, 5%, 10%, 25%, 50%, 75%, 90%, 95%, and 99% percentiles of $\hat{\zeta}$, plotted against each of the 15 study designs. In vitro diagnostic medical device measurements are simulated with homoscedastic measurement errors and are not influenced by differences in non-selectivity. The numbers on top of the curves represent the number of clinical samples within each study design.

percentiles increase in value as the study design size increases, while 50%, 75%, 90%, 95%, 97.5% and 99% percentiles decrease in value.

In summary, the distribution of $\hat{\zeta}$ is approximately centered at 1 for all study designs. The gap between the mean of $\hat{\zeta}$, $\mathrm{E}[\hat{\zeta}]$, and 1 narrows as n and R increase. The skewness of $\hat{\zeta}$ is consistently positive, indicating a right-skewed distribution. For reference, the normal distribution has a skewness of 0. The kurtosis consistently exceeds 3, signifying a higher kurtosis than the normal distribution, which has a kurtosis of 3. Based on the 99% percentile of $\hat{\zeta}$, the probability of $\hat{\zeta} > 2$ is always less than 1% for $n \geq 20$ and $R \geq 2$. Thus, if we observe $\hat{\zeta} > 2$, it is less than 1% likely that the compared IVD-MDs are influenced by DINS for that set of possible study designs.

4.3 Simulation results: third set of simulations

In the third set of simulations, we will consider the same study designs as we did for the second set of simulations. However, we will implement heteroscedastic measurement errors. $\hat{\zeta}$ values will be generated considering both non-decreasing IVD-MD standard deviations across the concentration interval, $\{\eta \geq 1, \eta_0 = 1\}$, and decreasing, $\{\eta < 1, \eta_0 = 1\}$. Heteroscedasticity defined in this way are unlikely to affect the mean of $\hat{\zeta}$. Nevertheless, variance, skewness, kurtosis, and percentiles of $\hat{\zeta}$ may be influenced by η.

Table 6. Distribution summary statistics of the 1%, 2.5%, 5%, 10%, 25%, 50%, 75%, 90%, 95%, 97.5%, and 99% percentiles of the differences in non-selectivity estimator, $\hat{\zeta}$, for various heteroscedasticity factors, η, accross all 15 considered study designs. 'Min. PSD' is short for minimizing percentile study design and 'Max. PSD' is short for maximizing percentile study design. Furthermore, 'Min.' is short for minimum and 'Max.' is short for maximum.

η	Min.	First quartile	Median	Third quartile	Max.	Min. PSD	Max. PSD
**1%**							
0.100	0.566	0.649	0.710	0.746	0.792	{n = 20, R = 2}	{n = 40, R = 4}
0.250	0.593	0.677	0.733	0.764	0.804	{n = 20, R = 2}	{n = 40, R = 4}
0.500	0.635	0.696	0.760	0.785	0.828	{n = 20, R = 2}	{n = 40, R = 4}
0.750	0.638	0.722	0.776	0.798	0.838	{n = 20, R = 2}	{n = 40, R = 4}
1.000	0.644	0.718	0.775	0.800	0.840	{n = 20, R = 2}	{n = 40, R = 4}
1.333	0.634	0.718	0.774	0.800	0.836	{n = 20, R = 2}	{n = 40, R = 4}
2.000	0.629	0.702	0.767	0.791	0.826	{n = 20, R = 2}	{n = 40, R = 4}
4.000	0.597	0.681	0.739	0.770	0.815	{n = 20, R = 2}	{n = 40, R = 4}
6.000	0.591	0.673	0.738	0.765	0.818	{n = 20, R = 2}	{n = 40, R = 4}
10.000	0.572	0.672	0.740	0.766	0.814	{n = 20, R = 2}	{n = 40, R = 4}
**2.5%**							
0.100	0.633	0.705	0.763	0.786	0.828	{n = 20, R = 2}	{n = 40, R = 4}
0.250	0.647	0.723	0.778	0.802	0.838	{n = 20, R = 2}	{n = 40, R = 4}
0.500	0.688	0.744	0.797	0.822	0.857	{n = 20, R = 2}	{n = 40, R = 4}
0.750	0.692	0.761	0.809	0.833	0.865	{n = 20, R = 2}	{n = 40, R = 4}
1.000	0.697	0.759	0.815	0.834	0.865	{n = 20, R = 2}	{n = 40, R = 4}
1.333	0.691	0.758	0.810	0.834	0.866	{n = 20, R = 2}	{n = 40, R = 4}
2.000	0.686	0.743	0.807	0.827	0.859	{n = 20, R = 2}	{n = 40, R = 4}

4.000	0.658	0.727	0.783	0.810	0.848	{n = 20, R = 2}	{n = 40, R = 4}
6.000	0.649	0.719	0.783	0.808	0.848	{n = 20, R = 2}	{n = 40, R = 4}
10.000	0.637	0.715	0.781	0.812	0.852	{n = 20, R = 2}	{n = 40, R = 4}
**5%**							
0.100	0.688	0.748	0.804	0.823	0.857	{n = 20, R = 2}	{n = 40, R = 4}
0.250	0.706	0.765	0.816	0.837	0.866	{n = 20, R = 2}	{n = 40, R = 4}
0.500	0.739	0.783	0.833	0.855	0.884	{n = 20, R = 2}	{n = 40, R = 4}
0.750	0.738	0.796	0.842	0.863	0.889	{n = 20, R = 2}	{n = 40, R = 4}
1.000	0.744	0.799	0.848	0.865	0.889	{n = 20, R = 2}	{n = 40, R = 4}
1.333	0.741	0.796	0.846	0.863	0.891	{n = 20, R = 2}	{n = 40, R = 4}
2.000	0.733	0.786	0.840	0.856	0.886	{n = 20, R = 2}	{n = 40, R = 4}
4.000	0.709	0.770	0.823	0.845	0.877	{n = 20, R = 2}	{n = 40, R = 4}
6.000	0.702	0.765	0.821	0.846	0.877	{n = 20, R = 2}	{n = 40, R = 4}
10.000	0.691	0.759	0.820	0.847	0.880	{n = 20, R = 2}	{n = 40, R = 4}
**10%**							
0.100	0.756	0.800	0.851	0.866	0.893	{n = 20, R = 2}	{n = 40, R = 4}
0.250	0.770	0.815	0.858	0.877	0.900	{n = 20, R = 2}	{n = 40, R = 4}
0.500	0.796	0.831	0.872	0.889	0.912	{n = 20, R = 2}	{n = 40, R = 4}
0.750	0.796	0.841	0.881	0.896	0.916	{n = 20, R = 2}	{n = 40, R = 4}
1.000	0.803	0.845	0.883	0.898	0.917	{n = 20, R = 2}	{n = 40, R = 4}
1.333	0.798	0.843	0.883	0.898	0.918	{n = 20, R = 2}	{n = 40, R = 4}
2.000	0.792	0.833	0.879	0.892	0.914	{n = 20, R = 2}	{n = 40, R = 4}
4.000	0.773	0.820	0.869	0.885	0.911	{n = 20, R = 2}	{n = 40, R = 4}
6.000	0.763	0.817	0.864	0.885	0.909	{n = 20, R = 2}	{n = 40, R = 4}
10.000	0.754	0.813	0.866	0.886	0.913	{n = 20, R = 2}	{n = 40, R = 4}
**25%**							
0.100	0.878	0.899	0.928	0.936	0.949	{n = 20, R = 2}	{n = 40, R = 4}
0.250	0.890	0.908	0.931	0.942	0.954	{n = 20, R = 2}	{n = 40, R = 4}
0.500	0.904	0.917	0.941	0.950	0.960	{n = 20, R = 2}	{n = 40, R = 4}
0.750	0.906	0.924	0.946	0.954	0.963	{n = 20, R = 2}	{n = 40, R = 4}
1.000	0.913	0.926	0.947	0.955	0.964	{n = 20, R = 2}	{n = 40, R = 4}
1.333	0.909	0.925	0.946	0.956	0.964	{n = 20, R = 2}	{n = 40, R = 4}
2.000	0.904	0.922	0.947	0.954	0.964	{n = 20, R = 2}	{n = 40, R = 4}
4.000	0.890	0.915	0.943	0.950	0.963	{n = 20, R = 2}	{n = 40, R = 4}
6.000	0.890	0.915	0.941	0.951	0.963	{n = 20, R = 2}	{n = 40, R = 4}
10.000	0.885	0.916	0.944	0.955	0.969	{n = 20, R = 2}	{n = 40, R = 4}
**50%**							
0.100	1.015	1.021	1.025	1.030	1.046	{n = 35, R = 4}	{n = 20, R = 2}
0.250	1.017	1.021	1.027	1.033	1.051	{n = 40, R = 4}	{n = 20, R = 2}
0.500	1.015	1.022	1.026	1.032	1.054	{n = 40, R = 4}	{n = 20, R = 2}
0.750	1.018	1.021	1.029	1.033	1.047	{n = 35, R = 4}	{n = 20, R = 2}
1.000	1.018	1.022	1.030	1.037	1.056	{n = 35, R = 4}	{n = 20, R = 2}

1.333	1.019	1.025	1.030	1.036	1.054	{n = 40, R = 4}	{n = 20, R = 2}
2.000	1.022	1.027	1.032	1.036	1.050	{n = 40, R = 4}	{n = 20, R = 2}
4.000	1.025	1.031	1.036	1.043	1.056	{n = 40, R = 4}	{n = 20, R = 2}
6.000	1.030	1.035	1.037	1.046	1.058	{n = 40, R = 4}	{n = 20, R = 2}
10.000	1.039	1.043	1.044	1.055	1.061	{n = 40, R = 4}	{n = 20, R = 2}
**75%**							
0.100	1.090	1.116	1.134	1.185	1.263	{n = 40, R = 4}	{n = 20, R = 2}
0.250	1.083	1.108	1.126	1.175	1.250	{n = 40, R = 4}	{n = 20, R = 2}
0.500	1.079	1.103	1.119	1.162	1.244	{n = 40, R = 4}	{n = 20, R = 2}
0.750	1.078	1.100	1.117	1.157	1.228	{n = 40, R = 4}	{n = 20, R = 2}
1.000	1.077	1.100	1.117	1.161	1.236	{n = 40, R = 4}	{n = 20, R = 2}
1.333	1.080	1.104	1.120	1.158	1.236	{n = 40, R = 4}	{n = 20, R = 2}
2.000	1.085	1.110	1.127	1.167	1.246	{n = 40, R = 4}	{n = 20, R = 2}
4.000	1.100	1.122	1.144	1.193	1.269	{n = 40, R = 4}	{n = 20, R = 2}
6.000	1.108	1.135	1.151	1.207	1.280	{n = 40, R = 4}	{n = 20, R = 2}
10.000	1.123	1.147	1.167	1.222	1.311	{n = 40, R = 4}	{n = 20, R = 2}
**90%**							
0.100	1.167	1.213	1.252	1.364	1.509	{n = 40, R = 4}	{n = 20, R = 2}
0.250	1.152	1.202	1.236	1.333	1.489	{n = 40, R = 4}	{n = 20, R = 2}
0.500	1.143	1.186	1.213	1.303	1.456	{n = 40, R = 4}	{n = 20, R = 2}
0.750	1.139	1.178	1.205	1.293	1.423	{n = 40, R = 4}	{n = 20, R = 2}
1.000	1.139	1.177	1.205	1.295	1.433	{n = 40, R = 4}	{n = 20, R = 2}
1.333	1.140	1.180	1.209	1.295	1.435	{n = 40, R = 4}	{n = 20, R = 2}
2.000	1.151	1.194	1.228	1.309	1.458	{n = 40, R = 4}	{n = 20, R = 2}
4.000	1.176	1.219	1.254	1.365	1.520	{n = 40, R = 4}	{n = 20, R = 2}
6.000	1.189	1.236	1.272	1.382	1.556	{n = 40, R = 4}	{n = 20, R = 2}
10.000	1.213	1.261	1.300	1.416	1.608	{n = 40, R = 4}	{n = 20, R = 2}
**95%**							
0.100	1.214	1.279	1.335	1.482	1.713	{n = 40, R = 4}	{n = 20, R = 2}
0.250	1.195	1.264	1.304	1.440	1.676	{n = 40, R = 4}	{n = 20, R = 2}
0.500	1.187	1.239	1.279	1.397	1.604	{n = 40, R = 4}	{n = 20, R = 2}
0.750	1.178	1.231	1.264	1.388	1.572	{n = 40, R = 4}	{n = 20, R = 2}
1.000	1.183	1.229	1.269	1.382	1.571	{n = 40, R = 4}	{n = 20, R = 2}
1.333	1.180	1.233	1.270	1.385	1.589	{n = 40, R = 4}	{n = 20, R = 2}
2.000	1.193	1.248	1.290	1.410	1.610	{n = 40, R = 4}	{n = 20, R = 2}
4.000	1.228	1.281	1.335	1.481	1.698	{n = 40, R = 4}	{n = 20, R = 2}
6.000	1.242	1.307	1.357	1.500	1.764	{n = 40, R = 4}	{n = 20, R = 2}
10.000	1.270	1.335	1.394	1.555	1.827	{n = 40, R = 4}	{n = 20, R = 2}
**97.5%**							
0.100	1.264	1.340	1.403	1.601	1.895	{n = 40, R = 4}	{n = 20, R = 2}
0.250	1.238	1.324	1.372	1.545	1.859	{n = 40, R = 4}	{n = 20, R = 2}
0.500	1.227	1.288	1.347	1.484	1.767	{n = 40, R = 4}	{n = 20, R = 2}

0.750	1.214	1.277	1.323	1.471	1.710	{n = 40, R = 4}	{n = 20, R = 2}
1.000	1.222	1.275	1.326	1.468	1.717	{n = 40, R = 4}	{n = 20, R = 2}
1.333	1.219	1.281	1.325	1.476	1.729	{n = 40, R = 4}	{n = 20, R = 2}
2.000	1.236	1.304	1.358	1.504	1.771	{n = 40, R = 4}	{n = 20, R = 2}
4.000	1.271	1.341	1.407	1.594	1.898	{n = 40, R = 4}	{n = 20, R = 2}
6.000	1.298	1.374	1.437	1.610	1.962	{n = 40, R = 4}	{n = 20, R = 2}
10.000	1.322	1.408	1.483	1.684	2.053	{n = 40, R = 4}	{n = 20, R = 2}
**99%**							
0.100	1.324	1.417	1.489	1.753	2.199	{n = 40, R = 4}	{n = 20, R = 2}
0.250	1.284	1.394	1.466	1.681	2.099	{n = 40, R = 4}	{n = 20, R = 2}
0.500	1.277	1.347	1.418	1.613	1.965	{n = 40, R = 4}	{n = 20, R = 2}
0.750	1.259	1.340	1.400	1.584	1.944	{n = 40, R = 4}	{n = 20, R = 2}
1.000	1.265	1.335	1.399	1.570	1.924	{n = 40, R = 4}	{n = 20, R = 2}
1.333	1.267	1.335	1.393	1.597	1.931	{n = 40, R = 4}	{n = 20, R = 2}
2.000	1.290	1.370	1.435	1.612	1.973	{n = 40, R = 4}	{n = 20, R = 2}
4.000	1.322	1.417	1.500	1.733	2.146	{n = 40, R = 4}	{n = 20, R = 2}
6.000	1.353	1.460	1.535	1.773	2.218	{n = 40, R = 4}	{n = 20, R = 2}
10.000	1.389	1.508	1.608	1.855	2.386	{n = 40, R = 4}	{n = 20, R = 2}

As Table 5 suggests, the mean of $\hat{\zeta}$ is merely affected by η. In detail, the mean range of $\hat{\zeta}$ over all regarded study designs are [1.021 - 1.106] and [1.053 - 1.131] for $\eta = 1/10$ and $\eta = 10$, respectively, as opposed to [1.024 - 1.094] for $\eta = 1$ (i.e., homoscedastic measurement errors). Furthermore, skewness and kurtosis are moderately affected by the heteroscedasticity factor, η. The values of skewness and kurtosis for $\eta = 1/10$ range from 0.433 to 1.486 and 3.671 to 8.051, respectively. This is as opposed to the values of skewness ranging from 0.427 to 1.140 and kurtosis ranging from 3.629 to 5.990 when $\eta = 1$. For $\eta = 10$, skewness and kurtosis range from 0.677 to 1.611 and 3.952 to 8.769, respectively. The variance is undoubtedly the moment most affected by introducing heteroscedasticity. For $\eta = 1/10$, the variance of $\hat{\zeta}$ is ranging from 0.012 to 0.112, as opposed to 0.008 to 0.069 – which is the case for $\eta = 1$. An especially dramatic change in the variance of $\hat{\zeta}$ is observed when $\eta = 10$, where it fall between 0.015 and 0.130.

Due to the considerable increase in the variance of $\hat{\zeta}$ when $\eta \to 1/10$ and $\eta \to 10$, the percentiles of $\hat{\zeta}$ are expected to be influenced accordingly. However, as Table 6 illustrates, only the upper percentiles, 90%, 95%, 97.5% and 99% are affected notably.

In addition to the effects of $\eta \neq 1$, various study designs exhibit a significant relationship with the first four moments and the percentiles of $\hat{\zeta}$. Figure 5**A** demonstrates that the influence of $\eta \to 1/10$ or $\eta \to 10$ on the first four moments is reduced when increasing either n or R. To achieve the vastest reduction in the impact of $\eta \to 1/10$ or $\eta \to 10$ on the first four moments, one ought to increase R (the number of replicated measurements). Although increasing n is also an option, doing so results in a less pronounced impact decrease compared to increasing R. The same observation is discerned by considering Figure 5**B**, which shows that the undesirable changes in the percentiles of $\hat{\zeta}$ can be partially mitigated by increasing either n or R.

Another observation based on Figures 5**A** and 5 **B** is that $\eta > 1$ (which represents increasing IVD-MD SDs with concentration) has a more significant impact on the first four moments and percentiles of $\hat{\zeta}$ compared

Table 5. Distribution summary statistics of the mean, variance, skewness and kurtosis of the differences in non-selectivity estimator, $\hat{\zeta}$, for various heteroscedasticity factors, η, accross all 15 regarded study designs. 'MSD' is short for moment study design.

η	**Minimum**	**First quartile**	**Median**	**Third quartile**	**Maximum**	**Minimizing MSD**	**Maximizing MSD**
**Mean**							
0.100	1.024	1.032	1.039	1.061	1.104	{n = 40, R = 4}	{n = 20, R = 2}
0.250	1.022	1.031	1.038	1.056	1.102	{n = 40, R = 4}	{n = 20, R = 2}
0.500	1.023	1.031	1.040	1.052	1.098	{n = 40, R = 4}	{n = 20, R = 2}
0.750	1.024	1.031	1.040	1.053	1.089	{n = 40, R = 4}	{n = 20, R = 2}
1.000	1.024	1.031	1.039	1.056	1.096	{n = 35, R = 4}	{n = 20, R = 2}
1.333	1.025	1.034	1.040	1.055	1.095	{n = 40, R = 4}	{n = 20, R = 2}
2.000	1.029	1.037	1.044	1.058	1.098	{n = 40, R = 4}	{n = 20, R = 2}
4.000	1.036	1.043	1.052	1.072	1.112	{n = 40, R = 4}	{n = 20, R = 2}
6.000	1.041	1.051	1.056	1.079	1.121	{n = 40, R = 4}	{n = 20, R = 2}
10.000	1.052	1.060	1.068	1.090	1.138	{n = 40, R = 4}	{n = 20, R = 2}
**Variance**							
0.100	0.012	0.020	0.027	0.053	0.115	{n = 40, R = 4}	{n = 20, R = 2}
0.250	0.010	0.017	0.023	0.045	0.096	{n = 40, R = 4}	{n = 20, R = 2}
0.500	0.009	0.014	0.019	0.037	0.076	{n = 40, R = 4}	{n = 20, R = 2}
0.750	0.008	0.013	0.017	0.033	0.071	{n = 40, R = 4}	{n = 20, R = 2}
1.000	0.008	0.013	0.017	0.033	0.070	{n = 40, R = 4}	{n = 20, R = 2}
1.333	0.008	0.013	0.017	0.034	0.072	{n = 40, R = 4}	{n = 20, R = 2}
2.000	0.009	0.015	0.020	0.038	0.078	{n = 40, R = 4}	{n = 20, R = 2}
4.000	0.012	0.018	0.025	0.049	0.104	{n = 40, R = 4}	{n = 20, R = 2}
6.000	0.013	0.021	0.027	0.053	0.115	{n = 40, R = 4}	{n = 20, R = 2}
10.000	0.015	0.023	0.032	0.062	0.139	{n = 40, R = 4}	{n = 20, R = 2}
**Skewness**							
0.100	0.482	0.536	0.690	1.002	2.547	{n = 40, R = 4}	{n = 20, R = 2}
0.250	0.364	0.495	0.603	0.862	1.458	{n = 40, R = 4}	{n = 20, R = 2}
0.500	0.428	0.484	0.557	0.784	1.118	{n = 40, R = 4}	{n = 20, R = 2}
0.750	0.398	0.498	0.539	0.815	1.272	{n = 30, R = 4}	{n = 20, R = 2}
1.000	0.424	0.478	0.580	0.711	1.210	{n = 35, R = 4}	{n = 20, R = 2}
1.333	0.435	0.471	0.560	0.777	1.147	{n = 25, R = 4}	{n = 20, R = 2}
2.000	0.460	0.510	0.629	0.837	1.115	{n = 25, R = 4}	{n = 20, R = 2}
4.000	0.551	0.588	0.676	0.934	1.562	{n = 40, R = 4}	{n = 20, R = 2}
6.000	0.575	0.661	0.735	0.951	1.486	{n = 40, R = 4}	{n = 20, R = 2}
10.000	0.614	0.741	0.868	1.099	1.670	{n = 35, R = 4}	{n = 20, R = 2}
**Kurtosis**							
0.100	3.696	3.934	4.184	5.251	33.481	{n = 30, R = 4}	{n = 20, R = 2}
0.250	3.510	3.703	4.014	4.475	8.675	{n = 35, R = 3}	{n = 20, R = 2}
0.500	3.496	3.713	3.740	4.270	5.394	{n = 40, R = 4}	{n = 20, R = 2}
0.750	3.536	3.697	3.821	4.471	6.981	{n = 30, R = 4}	{n = 20, R = 2}
1.000	3.527	3.704	3.860	4.061	6.715	{n = 35, R = 3}	{n = 20, R = 2}
1.333	3.537	3.646	3.796	4.255	5.804	{n = 40, R = 3}	{n = 20, R = 2}
2.000	3.629	3.813	4.052	4.599	5.748	{n = 25, R = 4}	{n = 35, R = 2}
4.000	3.692	3.942	4.003	4.745	9.630	{n = 35, R = 4}	{n = 20, R = 2}
6.000	3.692	4.053	4.269	4.872	7.963	{n = 40, R = 4}	{n = 20, R = 2}
10.000	3.830	4.270	4.641	5.533	8.594	{n = 35, R = 4}	{n = 20, R = 2}

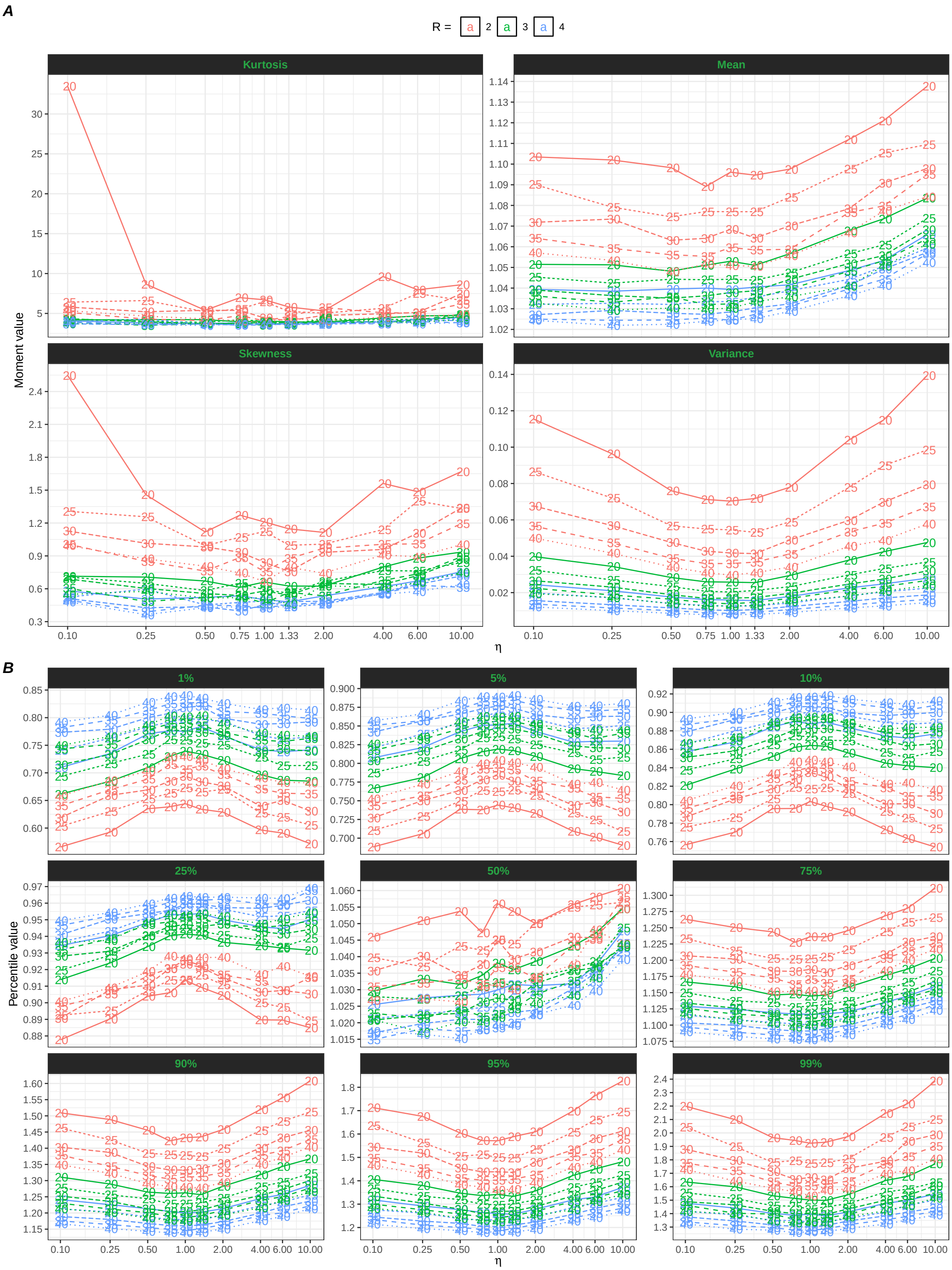

Figure 5. **A**: The mean, variance, skewness, and kurtosis of the differences in non-selectivity estimator, $\hat{\zeta}$, against the heteroscedasticity factor, η, and the 15 regarded study designs. **B**: The 1%, 5%, 10%, 25%, 50%, 75%, 90%, 95%, 97.5%, and 99% percentiles of $\hat{\zeta}$ against η and the 15 regarded study designs. The numbers on top of the curves represent the number of clinical samples within each study design.

to $\eta < 1$ (corresponding to decreasing IVD-MD SDs with concentration). Although the differences between the outcomes of $\eta > 1$ and $\eta < 1$ are not substantial, they remain consistent regarding the moments and percentiles of $\hat{\zeta}$.

4.4 Simulation results: fourth set of simulations

In the fourth set of simulations, we will investigate the same study designs as previously, but this time in combination of random DINS. We expect larger values of p and $m_{\max}$ to yield a higher mean value for $\hat{\zeta}$. Nevertheless, we also expect that random DINS will influence the variance, skewness, and kurtosis of $\hat{\zeta}$. By the assumed relationship between $\hat{\zeta}$ and the first four moments, we expect that random DINS will also impact the percentiles of $\hat{\zeta}$. The primary question of interest is to what extent the first four moments and the percentiles of $\hat{\zeta}$ will be affected by various combinations of p and $m_{\max}$ on top of the 15 considered study designs.

```
## [[1]]
## NULL
##
## [[2]]
## NULL
##
## [[3]]
## NULL
##
## [[4]]
## NULL
##
## [[5]]
## NULL
##
## [[6]]
## NULL
##
## [[7]]
## NULL
##
## [[8]]
## NULL
##
## [[9]]
## NULL
##
## [[10]]
## NULL
##
```

```
## [[11]]
## NULL
##
## [[12]]
## NULL
##
## [[13]]
## NULL
##
## [[14]]
## NULL
##
## [[15]]
## NULL
```

Table 7. Distribution summary statistics of the mean, variance, skewness, and kurtosis of the differences in non-selectivity (DINS) estimator, $\hat{\zeta}$, for all combinations of the average proportion of clinical samples influenced by DINS, p, and the maximum relocation magnitude multiplier, $m_{\max}$, accross all 15 considered study designs. 'Min. MSD' is short for minimizing moment study design and 'Max. PSD' is short for maximizing moment study design. Furthermore, 'Min.' 'Q1', 'Q3', and 'Max.' are short for minimum, first quartile, third quartile, and maximum, respectively.

p	$m_{\max}$	**Min.**	**Q1**	**Median**	**Q3**	**Max.**	**Min. MSD**	**Max. MSD**
**Mean**								
0.00	0.0	1.024	1.032	1.039	1.056	1.093	{n = 40, R = 4}	{n = 20, R = 2}
0.05	1.0	1.039	1.046	1.055	1.070	1.113	{n = 40, R = 4}	{n = 20, R = 2}
0.05	2.0	1.082	1.091	1.097	1.115	1.159	{n = 40, R = 4}	{n = 20, R = 2}
0.05	3.0	1.158	1.164	1.171	1.193	1.235	{n = 35, R = 4}	{n = 20, R = 2}
0.05	5.0	1.382	1.389	1.402	1.427	1.482	{n = 35, R = 4}	{n = 20, R = 2}
0.05	7.5	1.802	1.821	1.838	1.889	1.962	{n = 30, R = 4}	{n = 20, R = 2}
0.05	10.0	2.389	2.407	2.433	2.514	2.592	{n = 20, R = 4}	{n = 20, R = 2}
0.10	1.0	1.054	1.060	1.070	1.084	1.127	{n = 40, R = 4}	{n = 20, R = 2}
0.10	2.0	1.141	1.151	1.162	1.179	1.221	{n = 40, R = 4}	{n = 20, R = 2}
0.10	3.0	1.288	1.296	1.301	1.332	1.378	{n = 30, R = 4}	{n = 20, R = 2}
0.10	5.0	1.731	1.746	1.758	1.809	1.887	{n = 40, R = 4}	{n = 20, R = 2}
0.10	7.5	2.568	2.607	2.631	2.701	2.767	{n = 20, R = 4}	{n = 20, R = 2}
0.10	10.0	3.717	3.767	3.806	3.940	4.015	{n = 30, R = 4}	{n = 20, R = 2}
0.15	1.0	1.069	1.077	1.085	1.100	1.144	{n = 40, R = 4}	{n = 20, R = 2}
0.15	2.0	1.199	1.208	1.215	1.233	1.287	{n = 40, R = 4}	{n = 20, R = 2}
0.15	3.0	1.414	1.423	1.433	1.464	1.516	{n = 40, R = 4}	{n = 20, R = 2}
0.15	5.0	2.075	2.098	2.120	2.179	2.265	{n = 20, R = 4}	{n = 20, R = 2}
0.15	7.5	3.305	3.378	3.407	3.516	3.628	{n = 20, R = 4}	{n = 20, R = 2}
0.15	10.0	4.992	5.096	5.151	5.275	5.418	{n = 20, R = 4}	{n = 20, R = 2}
0.20	1.0	1.083	1.092	1.098	1.114	1.155	{n = 40, R = 4}	{n = 20, R = 2}

0.20	2.0	1.255	1.265	1.278	1.300	1.345	{n = 40, R = 4}	{n = 20, R = 2}
0.20	3.0	1.538	1.548	1.564	1.600	1.645	{n = 25, R = 4}	{n = 20, R = 2}
0.20	5.0	2.427	2.435	2.480	2.538	2.609	{n = 30, R = 4}	{n = 20, R = 2}
0.20	7.5	4.073	4.103	4.163	4.304	4.455	{n = 20, R = 4}	{n = 20, R = 2}
0.20	10.0	6.184	6.347	6.437	6.683	6.822	{n = 20, R = 4}	{n = 20, R = 2}
0.25	1.0	1.098	1.106	1.113	1.132	1.176	{n = 40, R = 4}	{n = 20, R = 2}
0.25	2.0	1.314	1.323	1.334	1.361	1.400	{n = 40, R = 4}	{n = 20, R = 2}
0.25	3.0	1.669	1.675	1.697	1.734	1.792	{n = 35, R = 4}	{n = 20, R = 2}
0.25	5.0	2.754	2.782	2.814	2.909	2.980	{n = 20, R = 4}	{n = 20, R = 2}
0.25	7.5	4.810	4.856	4.915	5.066	5.278	{n = 20, R = 4}	{n = 20, R = 2}
0.25	10.0	7.442	7.593	7.684	7.980	8.132	{n = 20, R = 4}	{n = 20, R = 2}
0.30	1.0	1.112	1.121	1.130	1.146	1.191	{n = 40, R = 4}	{n = 20, R = 2}
0.30	2.0	1.371	1.382	1.393	1.422	1.473	{n = 40, R = 4}	{n = 20, R = 2}
0.30	3.0	1.795	1.800	1.823	1.870	1.931	{n = 25, R = 4}	{n = 20, R = 2}
0.30	5.0	3.089	3.123	3.168	3.260	3.371	{n = 25, R = 4}	{n = 20, R = 2}
0.30	7.5	5.499	5.580	5.661	5.828	6.037	{n = 20, R = 4}	{n = 20, R = 2}
0.30	10.0	8.741	8.863	8.965	9.293	9.654	{n = 20, R = 4}	{n = 20, R = 2}
**Variance**								
0.00	0.0	0.008	0.012	0.017	0.032	0.068	{n = 40, R = 4}	{n = 20, R = 2}
0.05	1.0	0.009	0.013	0.018	0.035	0.074	{n = 40, R = 4}	{n = 20, R = 2}
0.05	2.0	0.013	0.019	0.024	0.042	0.089	{n = 40, R = 4}	{n = 20, R = 2}
0.05	3.0	0.027	0.037	0.051	0.070	0.138	{n = 40, R = 4}	{n = 20, R = 2}
0.05	5.0	0.130	0.167	0.203	0.253	0.421	{n = 40, R = 4}	{n = 20, R = 2}
0.05	7.5	0.574	0.692	0.853	1.067	1.633	{n = 40, R = 4}	{n = 20, R = 2}
0.05	10.0	1.706	2.076	2.465	3.139	4.599	{n = 40, R = 4}	{n = 20, R = 2}
0.10	1.0	0.009	0.014	0.020	0.036	0.076	{n = 40, R = 4}	{n = 20, R = 2}
0.10	2.0	0.018	0.026	0.033	0.055	0.113	{n = 40, R = 4}	{n = 20, R = 2}
0.10	3.0	0.048	0.061	0.089	0.111	0.202	{n = 40, R = 4}	{n = 20, R = 2}
0.10	5.0	0.250	0.330	0.391	0.499	0.807	{n = 40, R = 4}	{n = 20, R = 2}
0.10	7.5	1.200	1.438	1.738	2.155	3.269	{n = 40, R = 4}	{n = 20, R = 2}
0.10	10.0	3.616	4.353	4.996	6.338	9.082	{n = 40, R = 4}	{n = 20, R = 2}
0.15	1.0	0.010	0.016	0.020	0.038	0.082	{n = 40, R = 4}	{n = 20, R = 2}
0.15	2.0	0.022	0.033	0.044	0.063	0.128	{n = 40, R = 4}	{n = 20, R = 2}
0.15	3.0	0.065	0.088	0.124	0.150	0.278	{n = 40, R = 4}	{n = 20, R = 2}
0.15	5.0	0.392	0.491	0.595	0.724	1.236	{n = 40, R = 4}	{n = 20, R = 2}
0.15	7.5	1.846	2.275	2.699	3.310	5.045	{n = 40, R = 4}	{n = 20, R = 2}
0.15	10.0	5.703	6.935	7.849	9.652	13.965	{n = 40, R = 4}	{n = 20, R = 2}
0.20	1.0	0.010	0.017	0.022	0.039	0.084	{n = 40, R = 4}	{n = 20, R = 2}
0.20	2.0	0.027	0.039	0.055	0.078	0.156	{n = 40, R = 4}	{n = 20, R = 2}
0.20	3.0	0.085	0.113	0.156	0.198	0.338	{n = 40, R = 4}	{n = 20, R = 2}
0.20	5.0	0.541	0.667	0.784	1.003	1.567	{n = 40, R = 4}	{n = 20, R = 2}
0.20	7.5	2.598	3.132	3.791	4.439	7.237	{n = 40, R = 4}	{n = 20, R = 2}
0.20	10.0	8.042	9.727	11.512	13.407	20.290	{n = 40, R = 4}	{n = 20, R = 2}

0.25	1.0	0.011	0.017	0.023	0.042	0.092	{n = 40, R = 4}	{n = 20, R = 2}
0.25	2.0	0.033	0.046	0.061	0.091	0.171	{n = 40, R = 4}	{n = 20, R = 2}
0.25	3.0	0.108	0.143	0.197	0.239	0.416	{n = 40, R = 4}	{n = 20, R = 2}
0.25	5.0	0.672	0.865	1.020	1.274	2.093	{n = 40, R = 4}	{n = 20, R = 2}
0.25	7.5	3.432	4.195	4.854	5.822	9.319	{n = 40, R = 4}	{n = 20, R = 2}
0.25	10.0	10.553	13.024	14.940	17.292	25.779	{n = 40, R = 4}	{n = 20, R = 2}
0.30	1.0	0.012	0.018	0.024	0.044	0.093	{n = 40, R = 4}	{n = 20, R = 2}
0.30	2.0	0.039	0.053	0.074	0.103	0.209	{n = 40, R = 4}	{n = 20, R = 2}
0.30	3.0	0.128	0.171	0.237	0.286	0.523	{n = 40, R = 4}	{n = 20, R = 2}
0.30	5.0	0.879	1.051	1.253	1.609	2.477	{n = 40, R = 4}	{n = 20, R = 2}
0.30	7.5	4.154	5.164	5.975	7.128	11.227	{n = 40, R = 4}	{n = 20, R = 2}
0.30	10.0	13.658	16.256	18.776	22.029	33.731	{n = 40, R = 4}	{n = 20, R = 2}
**Skewness**								
0.00	0.0	0.420	0.462	0.576	0.763	1.116	{n = 30, R = 4}	{n = 20, R = 2}
0.05	1.0	0.444	0.507	0.550	0.790	1.229	{n = 35, R = 4}	{n = 20, R = 2}
0.05	2.0	0.665	0.712	0.802	0.894	1.260	{n = 40, R = 3}	{n = 20, R = 2}
0.05	3.0	0.966	1.069	1.170	1.301	1.745	{n = 40, R = 3}	{n = 20, R = 2}
0.05	5.0	1.299	1.394	1.487	1.652	1.879	{n = 40, R = 3}	{n = 20, R = 2}
0.05	7.5	1.213	1.408	1.576	1.804	2.103	{n = 40, R = 4}	{n = 20, R = 2}
0.05	10.0	1.245	1.482	1.549	1.872	2.227	{n = 40, R = 4}	{n = 20, R = 2}
0.10	1.0	0.442	0.509	0.579	0.746	1.230	{n = 40, R = 4}	{n = 25, R = 2}
0.10	2.0	0.687	0.767	0.883	0.999	1.537	{n = 40, R = 3}	{n = 20, R = 2}
0.10	3.0	0.885	0.985	1.087	1.208	1.460	{n = 40, R = 3}	{n = 20, R = 2}
0.10	5.0	0.951	1.077	1.220	1.407	1.747	{n = 40, R = 4}	{n = 20, R = 2}
0.10	7.5	0.947	1.116	1.258	1.394	1.839	{n = 40, R = 4}	{n = 20, R = 2}
0.10	10.0	0.987	1.110	1.218	1.385	1.684	{n = 40, R = 3}	{n = 20, R = 2}
0.15	1.0	0.487	0.564	0.616	0.817	1.230	{n = 40, R = 4}	{n = 20, R = 2}
0.15	2.0	0.665	0.751	0.869	0.963	1.204	{n = 40, R = 4}	{n = 20, R = 2}
0.15	3.0	0.829	0.935	1.002	1.161	1.496	{n = 40, R = 4}	{n = 20, R = 2}
0.15	5.0	0.759	0.909	1.047	1.151	1.673	{n = 40, R = 4}	{n = 20, R = 2}
0.15	7.5	0.844	0.953	1.065	1.268	1.630	{n = 40, R = 4}	{n = 20, R = 2}
0.15	10.0	0.792	0.967	1.049	1.219	1.465	{n = 40, R = 4}	{n = 20, R = 2}
0.20	1.0	0.435	0.565	0.617	0.787	1.247	{n = 40, R = 4}	{n = 20, R = 2}
0.20	2.0	0.684	0.792	0.910	0.994	1.391	{n = 40, R = 4}	{n = 20, R = 2}
0.20	3.0	0.702	0.885	0.999	1.086	1.435	{n = 40, R = 4}	{n = 20, R = 2}
0.20	5.0	0.715	0.825	0.940	1.115	1.347	{n = 40, R = 4}	{n = 20, R = 2}
0.20	7.5	0.681	0.799	0.976	1.087	1.751	{n = 40, R = 3}	{n = 20, R = 2}
0.20	10.0	0.581	0.798	1.002	1.093	1.502	{n = 40, R = 4}	{n = 20, R = 2}
0.25	1.0	0.531	0.579	0.654	0.766	1.278	{n = 30, R = 4}	{n = 20, R = 2}
0.25	2.0	0.703	0.748	0.828	1.002	1.264	{n = 30, R = 4}	{n = 20, R = 2}
0.25	3.0	0.564	0.783	0.894	0.995	1.336	{n = 40, R = 4}	{n = 20, R = 2}
0.25	5.0	0.563	0.711	0.810	1.037	1.515	{n = 40, R = 4}	{n = 20, R = 2}
0.25	7.5	0.515	0.730	0.859	1.019	1.591	{n = 40, R = 3}	{n = 20, R = 2}

0.25	10.0	0.479	0.681	0.790	1.013	1.400	{n = 40, R = 4}	{n = 20, R = 2}
0.30	1.0	0.510	0.571	0.618	0.875	1.187	{n = 35, R = 4}	{n = 20, R = 2}
0.30	2.0	0.617	0.726	0.859	0.982	1.407	{n = 40, R = 4}	{n = 20, R = 2}
0.30	3.0	0.513	0.745	0.888	0.970	1.510	{n = 40, R = 4}	{n = 20, R = 2}
0.30	5.0	0.477	0.601	0.808	1.018	1.202	{n = 35, R = 4}	{n = 20, R = 2}
0.30	7.5	0.397	0.568	0.714	0.870	1.295	{n = 40, R = 4}	{n = 20, R = 2}
0.30	10.0	0.401	0.541	0.752	0.853	1.272	{n = 40, R = 4}	{n = 20, R = 2}
**Kurtosis**								
0.00	0.0	3.617	3.722	3.819	4.277	5.880	{n = 30, R = 4}	{n = 20, R = 2}
0.05	1.0	3.564	3.660	3.908	4.304	6.575	{n = 40, R = 4}	{n = 20, R = 2}
0.05	2.0	3.868	4.062	4.407	4.828	6.678	{n = 40, R = 3}	{n = 20, R = 2}
0.05	3.0	4.747	5.154	5.530	6.327	11.133	{n = 40, R = 3}	{n = 20, R = 2}
0.05	5.0	5.565	6.147	6.390	7.292	8.383	{n = 35, R = 4}	{n = 20, R = 2}
0.05	7.5	4.715	5.739	6.646	7.490	9.572	{n = 40, R = 4}	{n = 20, R = 2}
0.05	10.0	4.891	6.140	6.592	8.130	11.077	{n = 40, R = 4}	{n = 20, R = 2}
0.10	1.0	3.537	3.687	3.866	4.182	8.167	{n = 30, R = 4}	{n = 25, R = 2}
0.10	2.0	3.846	4.174	4.603	5.088	9.151	{n = 40, R = 3}	{n = 20, R = 2}
0.10	3.0	4.241	4.746	5.141	5.848	6.564	{n = 40, R = 3}	{n = 20, R = 2}
0.10	5.0	4.408	4.891	5.426	6.024	9.047	{n = 40, R = 4}	{n = 20, R = 2}
0.10	7.5	4.307	5.026	5.384	5.983	8.566	{n = 40, R = 4}	{n = 20, R = 2}
0.10	10.0	4.343	4.820	5.359	5.975	7.671	{n = 40, R = 3}	{n = 20, R = 2}
0.15	1.0	3.593	3.845	4.066	4.467	6.399	{n = 20, R = 4}	{n = 20, R = 2}
0.15	2.0	3.836	3.997	4.598	5.042	5.861	{n = 40, R = 3}	{n = 30, R = 2}
0.15	3.0	4.131	4.500	4.905	5.248	7.431	{n = 35, R = 4}	{n = 20, R = 2}
0.15	5.0	3.924	4.333	4.783	5.260	8.404	{n = 40, R = 4}	{n = 20, R = 2}
0.15	7.5	4.153	4.744	4.907	5.654	7.905	{n = 35, R = 3}	{n = 20, R = 2}
0.15	10.0	4.032	4.632	5.036	5.520	6.513	{n = 40, R = 4}	{n = 25, R = 2}
0.20	1.0	3.590	3.803	4.086	4.276	6.692	{n = 35, R = 4}	{n = 20, R = 2}
0.20	2.0	3.825	4.195	4.769	5.258	6.903	{n = 40, R = 3}	{n = 20, R = 3}
0.20	3.0	4.028	4.398	4.765	5.503	6.870	{n = 35, R = 4}	{n = 20, R = 2}
0.20	5.0	4.127	4.497	4.645	5.077	5.927	{n = 30, R = 4}	{n = 30, R = 2}
0.20	7.5	3.872	4.300	4.475	5.326	10.723	{n = 35, R = 4}	{n = 20, R = 2}
0.20	10.0	3.623	4.287	4.711	5.377	7.427	{n = 40, R = 4}	{n = 20, R = 2}
0.25	1.0	3.644	3.825	4.066	4.318	6.627	{n = 35, R = 3}	{n = 20, R = 2}
0.25	2.0	3.767	4.114	4.390	5.519	6.166	{n = 30, R = 4}	{n = 20, R = 2}
0.25	3.0	3.582	4.175	4.518	4.751	6.159	{n = 40, R = 4}	{n = 20, R = 2}
0.25	5.0	3.743	4.049	4.209	5.134	7.853	{n = 40, R = 4}	{n = 20, R = 2}
0.25	7.5	3.836	4.271	4.626	5.019	8.883	{n = 40, R = 3}	{n = 20, R = 2}
0.25	10.0	3.839	4.157	4.360	5.382	7.376	{n = 40, R = 4}	{n = 20, R = 2}
0.30	1.0	3.602	3.679	3.844	4.827	6.316	{n = 35, R = 3}	{n = 20, R = 2}
0.30	2.0	3.739	4.133	4.571	4.883	7.065	{n = 40, R = 3}	{n = 20, R = 2}
0.30	3.0	3.888	4.162	4.474	4.848	8.359	{n = 40, R = 4}	{n = 20, R = 2}
0.30	5.0	3.708	4.020	4.323	5.254	5.483	{n = 35, R = 4}	{n = 25, R = 2}

0.30	7.5	3.952	4.053	4.295	5.006	6.321	{n = 35, R = 4}	{n = 20, R = 3}
0.30	10.0	3.698	4.112	4.279	4.648	6.134	{n = 40, R = 3}	{n = 25, R = 2}

Table 8. Distribution summary statistics of the 1%, 2.5%, 5%, 10%, 25%, 50%, 75%, 90%, 95%, 97.5%, and 99% percentiles of the differences in non-selectivity (DINS) estimator, $\hat{\zeta}$, for all combinations of p = 0, 0.05, 0.15, and 0.30 (average proportions of clinical samples influenced by DINS) and $m_{\max}$ = 0, 5, 7.5, and10 (maximum relocation magnitude multipliers), accross all 15 considered study designs. 'Min. PSD' is short for minimizing percentile study design and 'Max. PSD' is short for maximizing percentile study design. Furthermore, 'Min.' 'Q1', 'Q3', and 'Max.' are short for minimum, first quartile, third quartile, and maximum, respectively.

p	$m_{\max}$	**Min.**	**Q1**	**Median**	**Q3**	**Max.**	**Min. PSD**	**Max. PSD**
**1%**								
0.00	0.0	0.648	0.720	0.778	0.799	0.839	{n = 20, R = 2}	{n = 40, R = 4}
0.05	5.0	0.677	0.781	0.817	0.854	0.899	{n = 20, R = 2}	{n = 40, R = 4}
0.05	7.5	0.701	0.785	0.823	0.866	0.895	{n = 20, R = 2}	{n = 40, R = 4}
0.05	10.0	0.695	0.778	0.825	0.862	0.905	{n = 20, R = 2}	{n = 40, R = 4}
0.15	5.0	0.801	0.903	0.953	0.970	1.011	{n = 20, R = 2}	{n = 40, R = 4}
0.15	7.5	0.828	0.926	0.981	1.000	1.041	{n = 20, R = 2}	{n = 40, R = 4}
0.15	10.0	0.843	0.940	0.984	1.010	1.035	{n = 20, R = 2}	{n = 40, R = 4}
0.30	5.0	0.952	1.025	1.044	1.051	1.069	{n = 20, R = 2}	{n = 40, R = 4}
0.30	7.5	0.989	1.038	1.048	1.053	1.088	{n = 20, R = 2}	{n = 40, R = 4}
0.30	10.0	0.998	1.039	1.046	1.056	1.094	{n = 20, R = 2}	{n = 35, R = 3}
**2.5%**								
0.00	0.0	0.698	0.761	0.814	0.834	0.867	{n = 20, R = 2}	{n = 40, R = 4}
0.05	5.0	0.750	0.830	0.863	0.897	0.930	{n = 20, R = 2}	{n = 40, R = 4}
0.05	7.5	0.762	0.846	0.871	0.910	0.934	{n = 20, R = 2}	{n = 40, R = 4}
0.05	10.0	0.762	0.841	0.884	0.906	0.942	{n = 20, R = 2}	{n = 40, R = 4}
0.15	5.0	0.895	0.972	1.020	1.053	1.092	{n = 20, R = 2}	{n = 40, R = 4}
0.15	7.5	0.926	1.021	1.092	1.144	1.193	{n = 20, R = 2}	{n = 40, R = 3}
0.15	10.0	0.965	1.043	1.117	1.165	1.225	{n = 20, R = 2}	{n = 40, R = 3}
0.30	5.0	1.126	1.196	1.230	1.266	1.314	{n = 20, R = 2}	{n = 40, R = 4}
0.30	7.5	1.238	1.284	1.336	1.357	1.504	{n = 20, R = 2}	{n = 40, R = 4}
0.30	10.0	1.247	1.277	1.339	1.368	1.447	{n = 30, R = 4}	{n = 35, R = 2}
**5%**								
0.00	0.0	0.747	0.799	0.846	0.864	0.891	{n = 20, R = 2}	{n = 40, R = 4}
0.05	5.0	0.808	0.878	0.904	0.934	0.960	{n = 20, R = 2}	{n = 40, R = 4}
0.05	7.5	0.823	0.894	0.919	0.947	0.973	{n = 20, R = 2}	{n = 40, R = 4}
0.05	10.0	0.824	0.899	0.932	0.948	0.975	{n = 20, R = 2}	{n = 40, R = 4}
0.15	5.0	0.992	1.051	1.108	1.154	1.200	{n = 20, R = 2}	{n = 40, R = 4}
0.15	7.5	1.073	1.172	1.258	1.354	1.444	{n = 20, R = 2}	{n = 40, R = 3}
0.15	10.0	1.095	1.223	1.394	1.529	1.635	{n = 20, R = 4}	{n = 40, R = 2}
0.30	5.0	1.336	1.445	1.549	1.591	1.690	{n = 20, R = 2}	{n = 40, R = 4}

0.30	7.5	1.678	1.853	1.978	2.114	2.271	{n = 20, R = 2}	{n = 40, R = 4}
0.30	10.0	1.919	2.103	2.179	2.490	2.593	{n = 20, R = 2}	{n = 40, R = 4}
**10%**								
0.00	0.0	0.803	0.844	0.883	0.898	0.918	{n = 20, R = 2}	{n = 40, R = 4}
0.05	5.0	0.882	0.940	0.962	0.984	1.004	{n = 20, R = 2}	{n = 40, R = 4}
0.05	7.5	0.901	0.957	0.985	1.010	1.027	{n = 20, R = 2}	{n = 40, R = 4}
0.05	10.0	0.907	0.960	0.995	1.018	1.041	{n = 20, R = 2}	{n = 40, R = 3}
0.15	5.0	1.132	1.198	1.250	1.307	1.359	{n = 20, R = 2}	{n = 40, R = 4}
0.15	7.5	1.255	1.439	1.568	1.677	1.777	{n = 20, R = 4}	{n = 40, R = 3}
0.15	10.0	1.425	1.693	1.925	2.099	2.232	{n = 20, R = 4}	{n = 40, R = 3}
0.30	5.0	1.643	1.752	1.889	1.948	2.019	{n = 20, R = 3}	{n = 40, R = 4}
0.30	7.5	2.346	2.589	2.822	2.979	3.122	{n = 20, R = 4}	{n = 40, R = 4}
0.30	10.0	3.185	3.529	3.826	4.202	4.426	{n = 20, R = 4}	{n = 40, R = 4}
**25%**								
0.00	0.0	0.911	0.927	0.948	0.956	0.964	{n = 20, R = 2}	{n = 40, R = 4}
0.05	5.0	1.043	1.059	1.081	1.097	1.109	{n = 20, R = 2}	{n = 40, R = 3}
0.05	7.5	1.062	1.090	1.142	1.195	1.225	{n = 20, R = 4}	{n = 40, R = 2}
0.05	10.0	1.070	1.113	1.185	1.281	1.368	{n = 20, R = 4}	{n = 40, R = 2}
0.15	5.0	1.440	1.513	1.567	1.612	1.646	{n = 20, R = 4}	{n = 40, R = 4}
0.15	7.5	1.923	2.134	2.243	2.335	2.417	{n = 20, R = 4}	{n = 40, R = 3}
0.15	10.0	2.589	2.920	3.102	3.268	3.420	{n = 20, R = 4}	{n = 40, R = 3}
0.30	5.0	2.203	2.318	2.416	2.467	2.506	{n = 20, R = 3}	{n = 40, R = 4}
0.30	7.5	3.593	3.849	4.036	4.152	4.275	{n = 20, R = 3}	{n = 40, R = 3}
0.30	10.0	5.377	5.734	6.098	6.357	6.506	{n = 20, R = 4}	{n = 40, R = 3}
**50%**								
0.00	0.0	1.018	1.023	1.029	1.037	1.054	{n = 40, R = 4}	{n = 20, R = 2}
0.05	5.0	1.223	1.273	1.299	1.319	1.333	{n = 20, R = 4}	{n = 35, R = 2}
0.05	7.5	1.380	1.498	1.606	1.652	1.669	{n = 20, R = 4}	{n = 40, R = 2}
0.05	10.0	1.596	1.852	2.010	2.104	2.149	{n = 20, R = 3}	{n = 40, R = 2}
0.15	5.0	1.926	1.985	2.020	2.026	2.065	{n = 20, R = 4}	{n = 35, R = 2}
0.15	7.5	2.955	3.128	3.191	3.209	3.263	{n = 20, R = 4}	{n = 35, R = 2}
0.15	10.0	4.391	4.653	4.752	4.811	4.915	{n = 20, R = 4}	{n = 40, R = 2}
0.30	5.0	2.916	3.040	3.065	3.103	3.138	{n = 20, R = 4}	{n = 40, R = 2}
0.30	7.5	5.175	5.377	5.437	5.531	5.628	{n = 20, R = 4}	{n = 40, R = 2}
0.30	10.0	8.202	8.460	8.656	8.789	8.931	{n = 20, R = 4}	{n = 35, R = 2}
**75%**								
0.00	0.0	1.077	1.100	1.116	1.157	1.231	{n = 40, R = 4}	{n = 20, R = 2}
0.05	5.0	1.573	1.603	1.626	1.666	1.734	{n = 40, R = 4}	{n = 20, R = 2}
0.05	7.5	2.230	2.257	2.301	2.328	2.450	{n = 40, R = 3}	{n = 20, R = 2}
0.05	10.0	3.088	3.183	3.213	3.268	3.456	{n = 40, R = 4}	{n = 20, R = 2}
0.15	5.0	2.466	2.497	2.543	2.615	2.783	{n = 40, R = 4}	{n = 20, R = 2}
0.15	7.5	4.155	4.230	4.318	4.407	4.638	{n = 30, R = 4}	{n = 20, R = 2}

0.15	10.0	6.437	6.643	6.747	6.868	7.243	{n = 40, R = 4}	{n = 20, R = 2}
0.30	5.0	3.687	3.724	3.793	3.921	4.158	{n = 40, R = 4}	{n = 20, R = 2}
0.30	7.5	6.834	6.955	7.035	7.235	7.713	{n = 40, R = 4}	{n = 20, R = 2}
0.30	10.0	11.130	11.297	11.447	11.816	12.551	{n = 40, R = 4}	{n = 20, R = 2}
**90%**								
0.00	0.0	1.138	1.176	1.208	1.289	1.427	{n = 40, R = 4}	{n = 20, R = 2}
0.05	5.0	1.865	1.946	1.997	2.100	2.322	{n = 40, R = 4}	{n = 20, R = 2}
0.05	7.5	2.860	2.958	3.121	3.231	3.648	{n = 40, R = 4}	{n = 20, R = 2}
0.05	10.0	4.179	4.380	4.610	4.835	5.421	{n = 40, R = 4}	{n = 20, R = 2}
0.15	5.0	2.923	3.043	3.172	3.289	3.684	{n = 40, R = 4}	{n = 20, R = 2}
0.15	7.5	5.159	5.353	5.633	5.823	6.604	{n = 40, R = 4}	{n = 20, R = 2}
0.15	10.0	8.186	8.564	8.897	9.289	10.273	{n = 40, R = 4}	{n = 20, R = 2}
0.30	5.0	4.328	4.441	4.692	4.863	5.452	{n = 40, R = 4}	{n = 20, R = 2}
0.30	7.5	8.178	8.547	8.869	9.218	10.371	{n = 40, R = 4}	{n = 20, R = 2}
0.30	10.0	13.432	14.080	14.568	15.231	17.124	{n = 40, R = 4}	{n = 20, R = 2}
**95%**								
0.00	0.0	1.177	1.226	1.269	1.377	1.570	{n = 40, R = 4}	{n = 20, R = 2}
0.05	5.0	2.070	2.187	2.286	2.415	2.762	{n = 40, R = 4}	{n = 20, R = 2}
0.05	7.5	3.280	3.433	3.687	3.887	4.517	{n = 40, R = 4}	{n = 20, R = 2}
0.05	10.0	4.935	5.225	5.615	5.993	6.860	{n = 40, R = 4}	{n = 20, R = 2}
0.15	5.0	3.233	3.394	3.575	3.768	4.371	{n = 40, R = 4}	{n = 20, R = 2}
0.15	7.5	5.821	6.136	6.450	6.850	8.004	{n = 40, R = 4}	{n = 20, R = 2}
0.15	10.0	9.451	9.930	10.378	11.017	12.741	{n = 40, R = 4}	{n = 20, R = 2}
0.30	5.0	4.745	4.944	5.220	5.523	6.368	{n = 40, R = 4}	{n = 20, R = 2}
0.30	7.5	9.017	9.583	10.022	10.573	12.374	{n = 40, R = 4}	{n = 20, R = 2}
0.30	10.0	15.030	15.900	16.717	17.644	20.542	{n = 40, R = 4}	{n = 20, R = 2}
**97.5%**								
0.00	0.0	1.213	1.272	1.324	1.462	1.713	{n = 40, R = 4}	{n = 20, R = 2}
0.05	5.0	2.272	2.414	2.534	2.707	3.234	{n = 40, R = 4}	{n = 20, R = 2}
0.05	7.5	3.690	3.899	4.231	4.563	5.422	{n = 40, R = 4}	{n = 20, R = 2}
0.05	10.0	5.622	6.086	6.491	7.074	8.306	{n = 40, R = 4}	{n = 20, R = 2}
0.15	5.0	3.535	3.733	3.951	4.224	5.024	{n = 40, R = 4}	{n = 20, R = 2}
0.15	7.5	6.433	6.904	7.202	7.871	9.298	{n = 40, R = 4}	{n = 20, R = 2}
0.15	10.0	10.618	11.270	11.728	12.658	14.793	{n = 40, R = 4}	{n = 20, R = 2}
0.30	5.0	5.105	5.423	5.793	6.141	7.281	{n = 40, R = 4}	{n = 20, R = 2}
0.30	7.5	9.866	10.582	11.121	11.907	14.210	{n = 40, R = 4}	{n = 20, R = 2}
0.30	10.0	16.749	17.760	18.647	19.809	24.042	{n = 40, R = 4}	{n = 20, R = 2}
**99%**								
0.00	0.0	1.259	1.330	1.395	1.571	1.887	{n = 40, R = 4}	{n = 20, R = 2}
0.05	5.0	2.482	2.697	2.885	3.123	3.774	{n = 40, R = 4}	{n = 20, R = 2}
0.05	7.5	4.191	4.540	4.986	5.505	6.554	{n = 40, R = 4}	{n = 20, R = 2}
0.05	10.0	6.493	7.223	7.641	8.445	10.461	{n = 40, R = 4}	{n = 20, R = 2}

0.15	5.0	3.890	4.150	4.451	4.767	5.992	{n = 40, R = 4}	{n = 20, R = 2}
0.15	7.5	7.181	7.807	8.308	9.232	11.152	{n = 40, R = 4}	{n = 20, R = 2}
0.15	10.0	12.058	12.749	13.698	14.835	17.843	{n = 40, R = 4}	{n = 20, R = 2}
0.30	5.0	5.692	5.983	6.581	6.941	8.298	{n = 40, R = 4}	{n = 20, R = 2}
0.30	7.5	11.000	11.924	12.672	13.532	16.927	{n = 40, R = 4}	{n = 20, R = 2}
0.30	10.0	18.744	19.907	21.250	22.678	28.365	{n = 40, R = 4}	{n = 20, R = 2}

From Table 7, we observe that $p = 3/10$ and $m_{\max} = 10$ produces the largest mean and variance values of $\hat{\zeta}$ for every study design. In contrast, $p = 0$ and $m_{\max} = 0$ produces the smallest mean and variance values of $\hat{\zeta}$ for each study design. In general, the skewness and kurtosis $\hat{\zeta}$ are smallest for $p = 0$ and $m_{\max} = 0$ and largest when $p = 1/20$ and $m_{\max} = 10$.

The mean and variance of $\hat{\zeta}$ are both deduced both positively correlated with both p and $m_{\max}$, as illustrated in Figure 6. For example, in a typical study design with $n = 25$ and $R = 3$, setting $p = 1/10$ and $m_{\max} = 5$ results in a mean and variance of $\hat{\zeta}$ of 1.768 and 0.448, respectively. Comparatively, for IVD-MDs with no DINS and the same study design, the mean and variance of $\hat{\zeta}$ are 1.044 and 0.020, respectively. Therefore, by increasing p from 0 to $1/10$ and $m_{\max}$ from 0 to 5, the resulting mean and variance of $\hat{\zeta}$ are 1.693 and 21.998 times larger than in the absence of DINS.

The skewness and kurtosis of $\hat{\zeta}$ exhibit a somewhat different relationship with p and $m_{\max}$ compared to its mean and variance. Figure 6 elucidates that, for $m_{\max}$ above a certain threshold, the skewness and kurtosis of $\hat{\zeta}$ decrease as p increases. Indeed, while increasing $m_{\max}$ raises the skewness and kurtosis of $\hat{\zeta}$, increasing p reduces them, except when $m_{\max} < 3$. Consequently, a suitably small p coupled with a large $m_{\max}$ would indeed yield the highest skewness and kurtosis of $\hat{\zeta}$.

Among the four moments, variance is the most significantly affected by increasing p or $m_{\max}$. As a result, the percentiles of $\hat{\zeta}$ are expected to be accordingly impacted. Due to the potential substantial increase in variance, the smallest percentiles of $\hat{\zeta}$ are postulated to become smaller, while the largest percentiles of $\hat{\zeta}$ should increase. However, Table 8 specifies that the smallest percentiles of $\hat{\zeta}$ do not decrease with increasing p and $m_{\max}$; instead, they increase. This could be put down to the mean of $\hat{\zeta}$ also increasing as p and $m_{\max}$ increment. The largest percentiles are hypothesized to increase and indeed do so as p and $m_{\max}$ increment.

4.5 Simulation results: fifth set of simulations

In the fifth set of simulations, we will examine the same study designs as before, but this time implementing DINS as defined in simulation setting 4: systematic DINS. We will investigate the relationship between the distribution of $\hat{\zeta}$, the quantile intervals $q = [l, u]$, and $m_{\max}$. The quantile intervals, $q = [l, u]$, with quantile range $u - l$ and $m_{\max}$, are postulated to influence the mean, variance, skewness and kurtosis $\hat{\zeta}$, but also the percentiles of $\hat{\zeta}$.

Specifically, the mean and variance of $\hat{\zeta}$ are hypothesized to increase with both $u - l$ and $m_{\max}$. This is because $u - l$ is a parameter similar to the random DINS parameter p from simulation setting 3, and in the fourth set of simulations, we observed that both the mean and variance of $\hat{\zeta}$ increased with p. We anticipate a similar pattern for $u - l$ in the current set of simulations.

We also observed that the percentiles of $\hat{\zeta}$ increased in value with both p and $m_{\max}$. Therefore, we believe

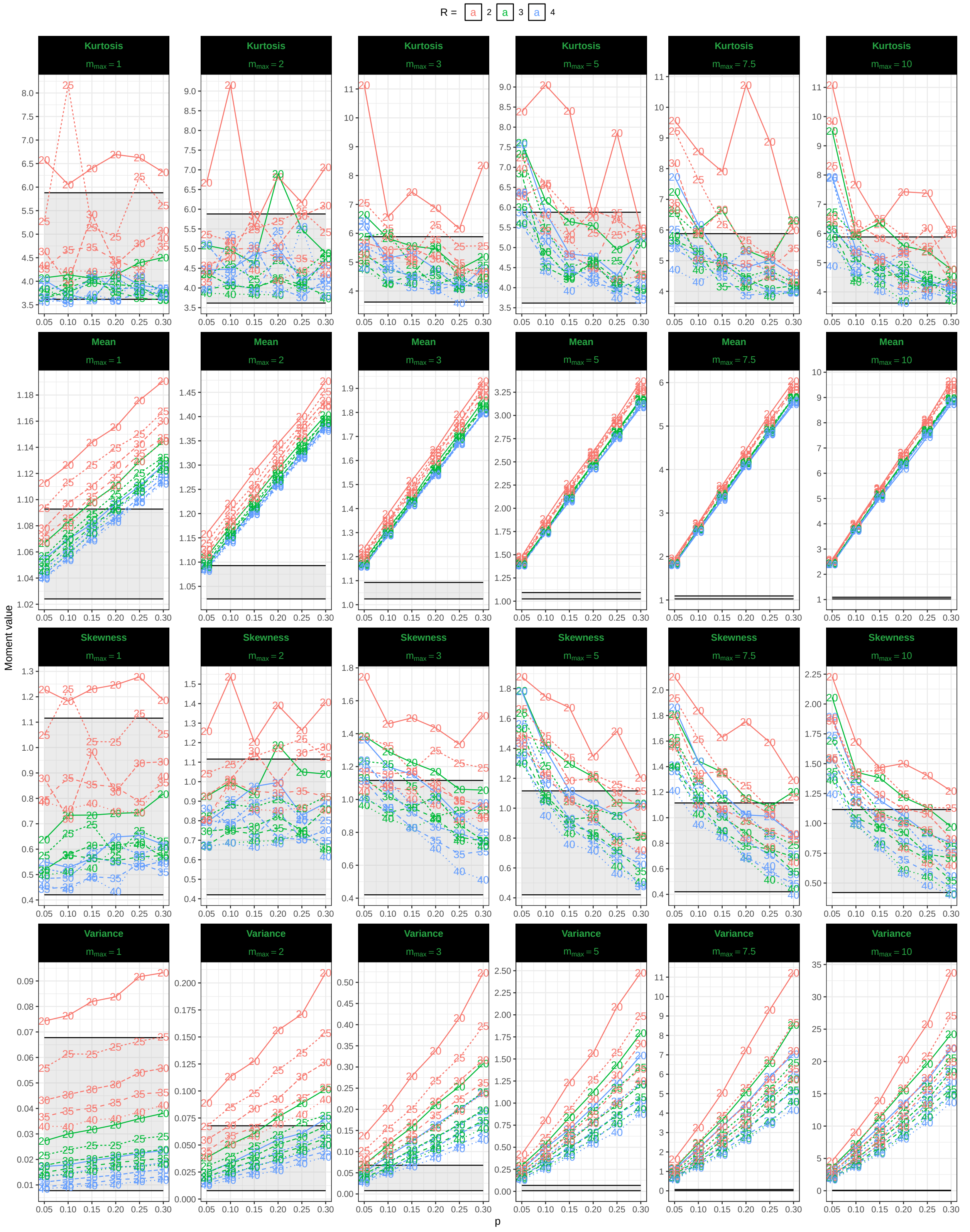

Figure 6. The mean, variance, skewness and kurtosis of the differences in non-selectivity estimator, $\hat{\zeta}$, for all combinations of the average proportion of clinical samples affected by differences in non-selectivity, p, the maximum relocation magnitude multiplier, $m_{\max}$, and 15 regarded study designs. The gray ribbons represent the ranges of moment values when there are no differences in non-selectivity (i.e., $p = 0$, $m_{\max} = 0$ or both). The numbers on top of the curves represent the number of clinical samples within each study design.

the percentiles to also increase with both $u - l$ and $m_{\max}$.

```
## [[1]]
## NULL
##
## [[2]]
## NULL
##
## [[3]]
## NULL
##
## [[4]]
## NULL
##
## [[5]]
## NULL
##
## [[6]]
## NULL
##
## [[7]]
## NULL
##
## [[8]]
## NULL
##
## [[9]]
## NULL
##
## [[10]]
## NULL
##
## [[11]]
## NULL
##
## [[12]]
## NULL
##
## [[13]]
## NULL
##
## [[14]]
## NULL
##
```

```
## [[15]]
## NULL
```

Table 9. Distribution summary statistics of the mean, variance, skewness and kurtosis of the differences in non-selectivity estimator, $\hat{\zeta}$, for all combinations of quantile intervals, q, and maximum relocation magnitude multipliers, $m_{\max}$, across all 15 considered study designs. 'Min. MSD' and 'Max. MSD' are short for 'Minimize moment study design' and 'Maximize moment study design', respectively. Furthermore, 'Min.', 'Q1', 'Med.', 'Q3' and 'Max.' refers to Minimum, First quartile, Median, Third quartile and Maximum, respectively.

$u-l$	$m_{\max}$	q	**Min.**	**Q1**	**Med.**	**Q3**	**Max.**	**Min. MSD**	**Max. MSD**
**Mean**									
0.00	0.0		1.024	1.032	1.040	1.056	1.093	{n = 40, R = 4}	{n = 20, R = 2}
0.00	0.0		1.024	1.032	1.039	1.055	1.095	{n = 40, R = 4}	{n = 20, R = 2}
0.05	1.0	[0, 0.05]	1.039	1.047	1.052	1.068	1.110	{n = 40, R = 4}	{n = 20, R = 2}
0.05	1.0	[0.95, 1]	1.038	1.045	1.052	1.068	1.108	{n = 40, R = 4}	{n = 20, R = 2}
0.05	2.0	[0, 0.05]	1.078	1.086	1.093	1.110	1.151	{n = 40, R = 4}	{n = 20, R = 2}
0.05	2.0	[0.95, 1]	1.075	1.084	1.095	1.112	1.148	{n = 40, R = 4}	{n = 20, R = 2}
0.05	3.0	[0, 0.05]	1.145	1.152	1.158	1.181	1.216	{n = 40, R = 4}	{n = 20, R = 2}
0.05	3.0	[0.95, 1]	1.146	1.150	1.160	1.180	1.226	{n = 35, R = 4}	{n = 20, R = 2}
0.05	5.0	[0, 0.05]	1.343	1.354	1.368	1.397	1.445	{n = 20, R = 4}	{n = 20, R = 2}
0.05	5.0	[0.95, 1]	1.347	1.361	1.370	1.400	1.442	{n = 30, R = 4}	{n = 20, R = 2}
0.05	7.5	[0, 0.05]	1.712	1.729	1.744	1.795	1.826	{n = 20, R = 4}	{n = 20, R = 2}
0.05	7.5	[0.95, 1]	1.741	1.761	1.782	1.836	1.866	{n = 25, R = 4}	{n = 20, R = 2}
0.05	10.0	[0, 0.05]	2.168	2.215	2.239	2.285	2.351	{n = 20, R = 4}	{n = 25, R = 2}
0.05	10.0	[0.95, 1]	2.246	2.301	2.342	2.412	2.431	{n = 20, R = 4}	{n = 40, R = 2}
0.10	1.0	[0, 0.1]	1.047	1.055	1.062	1.078	1.118	{n = 40, R = 4}	{n = 20, R = 2}
0.10	1.0	[0.9, 1]	1.048	1.055	1.063	1.080	1.115	{n = 40, R = 4}	{n = 20, R = 2}
0.10	2.0	[0, 0.1]	1.116	1.123	1.132	1.150	1.184	{n = 40, R = 4}	{n = 20, R = 2}
0.10	2.0	[0.9, 1]	1.117	1.124	1.132	1.150	1.192	{n = 40, R = 4}	{n = 20, R = 2}
0.10	3.0	[0, 0.1]	1.233	1.235	1.244	1.272	1.311	{n = 40, R = 4}	{n = 20, R = 2}
0.10	3.0	[0.9, 1]	1.231	1.239	1.247	1.272	1.317	{n = 35, R = 4}	{n = 20, R = 2}
0.10	5.0	[0, 0.1]	1.574	1.589	1.604	1.644	1.680	{n = 20, R = 4}	{n = 20, R = 2}
0.10	5.0	[0.9, 1]	1.589	1.595	1.618	1.648	1.689	{n = 25, R = 4}	{n = 20, R = 2}
0.10	7.5	[0, 0.1]	2.199	2.243	2.268	2.333	2.370	{n = 20, R = 4}	{n = 30, R = 2}
0.10	7.5	[0.9, 1]	2.246	2.296	2.320	2.394	2.423	{n = 20, R = 4}	{n = 20, R = 2}
0.10	10.0	[0, 0.1]	3.022	3.085	3.100	3.236	3.263	{n = 20, R = 4}	{n = 25, R = 2}
0.10	10.0	[0.9, 1]	3.156	3.224	3.285	3.392	3.437	{n = 20, R = 4}	{n = 20, R = 2}
0.15	1.0	[0, 0.15]	1.054	1.062	1.070	1.083	1.123	{n = 40, R = 4}	{n = 20, R = 2}
0.15	1.0	[0.85, 1]	1.053	1.062	1.069	1.085	1.121	{n = 40, R = 4}	{n = 20, R = 2}
0.15	2.0	[0, 0.15]	1.141	1.150	1.161	1.183	1.221	{n = 40, R = 4}	{n = 20, R = 2}
0.15	2.0	[0.85, 1]	1.143	1.150	1.160	1.181	1.215	{n = 40, R = 4}	{n = 20, R = 2}
0.15	3.0	[0, 0.15]	1.288	1.292	1.308	1.330	1.367	{n = 30, R = 4}	{n = 20, R = 2}
0.15	3.0	[0.85, 1]	1.289	1.295	1.310	1.334	1.380	{n = 25, R = 4}	{n = 20, R = 2}
0.15	5.0	[0, 0.15]	1.735	1.747	1.767	1.817	1.849	{n = 25, R = 4}	{n = 20, R = 2}

0.15	5.0	[0.85, 1]	1.744	1.757	1.778	1.830	1.858	{n = 20, R = 4}	{n = 20, R = 2}
0.15	7.5	[0, 0.15]	2.535	2.595	2.627	2.706	2.729	{n = 20, R = 4}	{n = 25, R = 2}
0.15	7.5	[0.85, 1]	2.582	2.652	2.675	2.767	2.819	{n = 20, R = 4}	{n = 20, R = 2}
0.15	10.0	[0, 0.15]	3.577	3.706	3.743	3.872	3.966	{n = 20, R = 4}	{n = 20, R = 2}
0.15	10.0	[0.85, 1]	3.760	3.868	3.906	4.049	4.095	{n = 20, R = 4}	{n = 20, R = 2}
0.20	1.0	[0, 0.2]	1.058	1.064	1.072	1.089	1.127	{n = 40, R = 4}	{n = 20, R = 2}
0.20	1.0	[0.8, 1]	1.059	1.065	1.074	1.089	1.134	{n = 35, R = 4}	{n = 20, R = 2}
0.20	2.0	[0, 0.2]	1.158	1.165	1.173	1.198	1.241	{n = 40, R = 4}	{n = 20, R = 2}
0.20	2.0	[0.8, 1]	1.158	1.167	1.174	1.196	1.235	{n = 40, R = 4}	{n = 20, R = 2}
0.20	3.0	[0, 0.2]	1.322	1.327	1.338	1.364	1.408	{n = 20, R = 4}	{n = 20, R = 2}
0.20	3.0	[0.8, 1]	1.326	1.328	1.342	1.367	1.404	{n = 40, R = 4}	{n = 20, R = 2}
0.20	5.0	[0, 0.2]	1.819	1.845	1.870	1.909	1.967	{n = 20, R = 4}	{n = 20, R = 2}
0.20	5.0	[0.8, 1]	1.834	1.853	1.871	1.925	1.974	{n = 20, R = 4}	{n = 20, R = 2}
0.20	7.5	[0, 0.2]	2.767	2.821	2.842	2.929	2.986	{n = 20, R = 4}	{n = 25, R = 2}
0.20	7.5	[0.8, 1]	2.827	2.854	2.897	2.975	3.043	{n = 25, R = 4}	{n = 20, R = 2}
0.20	10.0	[0, 0.2]	3.953	4.103	4.136	4.279	4.356	{n = 20, R = 4}	{n = 20, R = 2}
0.20	10.0	[0.8, 1]	4.150	4.261	4.280	4.427	4.521	{n = 20, R = 4}	{n = 20, R = 2}
0.25	1.0	[0, 0.25]	1.059	1.067	1.074	1.093	1.132	{n = 40, R = 4}	{n = 20, R = 2}
0.25	1.0	[0.75, 1]	1.060	1.066	1.072	1.091	1.129	{n = 40, R = 4}	{n = 20, R = 2}
0.25	2.0	[0, 0.25]	1.163	1.173	1.179	1.200	1.242	{n = 35, R = 4}	{n = 20, R = 2}
0.25	2.0	[0.75, 1]	1.165	1.174	1.183	1.199	1.243	{n = 40, R = 4}	{n = 20, R = 2}
0.25	3.0	[0, 0.25]	1.339	1.340	1.354	1.383	1.439	{n = 30, R = 4}	{n = 20, R = 2}
0.25	3.0	[0.75, 1]	1.339	1.344	1.357	1.385	1.427	{n = 35, R = 4}	{n = 20, R = 2}
0.25	5.0	[0, 0.25]	1.876	1.888	1.908	1.968	2.022	{n = 20, R = 4}	{n = 20, R = 2}
0.25	5.0	[0.75, 1]	1.881	1.891	1.917	1.963	2.022	{n = 20, R = 4}	{n = 20, R = 2}
0.25	7.5	[0, 0.25]	2.874	2.920	2.952	3.036	3.111	{n = 20, R = 4}	{n = 20, R = 2}
0.25	7.5	[0.75, 1]	2.898	2.965	3.002	3.084	3.146	{n = 20, R = 4}	{n = 20, R = 2}
0.25	10.0	[0, 0.25]	4.188	4.264	4.319	4.496	4.530	{n = 20, R = 4}	{n = 30, R = 2}
0.25	10.0	[0.75, 1]	4.311	4.400	4.458	4.623	4.748	{n = 25, R = 4}	{n = 20, R = 2}
0.30	1.0	[0, 0.3]	1.058	1.067	1.075	1.092	1.134	{n = 40, R = 4}	{n = 20, R = 2}
0.30	1.0	[0.7, 1]	1.059	1.067	1.075	1.090	1.130	{n = 40, R = 4}	{n = 20, R = 2}
0.30	2.0	[0, 0.3]	1.162	1.170	1.178	1.200	1.242	{n = 40, R = 4}	{n = 20, R = 2}
0.30	2.0	[0.7, 1]	1.166	1.171	1.180	1.202	1.243	{n = 35, R = 4}	{n = 20, R = 2}
0.30	3.0	[0, 0.3]	1.335	1.342	1.356	1.381	1.425	{n = 40, R = 4}	{n = 20, R = 2}
0.30	3.0	[0.7, 1]	1.336	1.343	1.352	1.385	1.420	{n = 30, R = 4}	{n = 20, R = 2}
0.30	5.0	[0, 0.3]	1.870	1.894	1.908	1.958	2.023	{n = 20, R = 4}	{n = 20, R = 2}
0.30	5.0	[0.7, 1]	1.868	1.891	1.912	1.965	2.011	{n = 20, R = 4}	{n = 20, R = 2}
0.30	7.5	[0, 0.3]	2.879	2.934	2.956	3.048	3.126	{n = 20, R = 4}	{n = 20, R = 2}
0.30	7.5	[0.7, 1]	2.918	2.956	2.999	3.079	3.163	{n = 20, R = 4}	{n = 20, R = 2}
0.30	10.0	[0, 0.3]	4.207	4.343	4.363	4.542	4.636	{n = 20, R = 4}	{n = 20, R = 2}
0.30	10.0	[0.7, 1]	4.347	4.430	4.493	4.618	4.737	{n = 20, R = 4}	{n = 20, R = 2}
**Variance**									
0.00	0.0		0.008	0.012	0.017	0.032	0.068	{n = 40, R = 4}	{n = 20, R = 2}

0.00	0.0		0.008	0.012	0.017	0.033	0.069	{n = 40, R = 4}	{n = 20, R = 2}
0.05	1.0	[0, 0.05]	0.009	0.014	0.018	0.035	0.074	{n = 40, R = 4}	{n = 20, R = 2}
0.05	1.0	[0.95, 1]	0.008	0.013	0.018	0.034	0.073	{n = 40, R = 4}	{n = 20, R = 2}
0.05	2.0	[0, 0.05]	0.012	0.019	0.024	0.042	0.086	{n = 40, R = 4}	{n = 20, R = 2}
0.05	2.0	[0.95, 1]	0.011	0.018	0.025	0.041	0.085	{n = 40, R = 4}	{n = 20, R = 2}
0.05	3.0	[0, 0.05]	0.023	0.034	0.046	0.065	0.127	{n = 40, R = 4}	{n = 20, R = 2}
0.05	3.0	[0.95, 1]	0.024	0.034	0.046	0.065	0.125	{n = 40, R = 4}	{n = 20, R = 2}
0.05	5.0	[0, 0.05]	0.114	0.144	0.170	0.220	0.378	{n = 40, R = 4}	{n = 20, R = 2}
0.05	5.0	[0.95, 1]	0.114	0.144	0.173	0.224	0.358	{n = 40, R = 4}	{n = 20, R = 2}
0.05	7.5	[0, 0.05]	0.495	0.615	0.736	0.874	1.318	{n = 40, R = 4}	{n = 20, R = 2}
0.05	7.5	[0.95, 1]	0.499	0.634	0.755	0.913	1.367	{n = 40, R = 4}	{n = 20, R = 2}
0.05	10.0	[0, 0.05]	1.500	1.796	2.076	2.532	3.790	{n = 40, R = 4}	{n = 20, R = 2}
0.05	10.0	[0.95, 1]	1.504	1.886	2.175	2.655	3.934	{n = 40, R = 4}	{n = 20, R = 2}
0.10	1.0	[0, 0.1]	0.009	0.014	0.019	0.035	0.073	{n = 40, R = 4}	{n = 20, R = 2}
0.10	1.0	[0.9, 1]	0.009	0.014	0.019	0.036	0.073	{n = 40, R = 4}	{n = 20, R = 2}
0.10	2.0	[0, 0.1]	0.014	0.021	0.028	0.046	0.098	{n = 40, R = 4}	{n = 20, R = 2}
0.10	2.0	[0.9, 1]	0.014	0.022	0.029	0.047	0.099	{n = 40, R = 4}	{n = 20, R = 2}
0.10	3.0	[0, 0.1]	0.032	0.045	0.061	0.080	0.159	{n = 40, R = 4}	{n = 20, R = 2}
0.10	3.0	[0.9, 1]	0.031	0.044	0.062	0.084	0.164	{n = 40, R = 4}	{n = 20, R = 2}
0.10	5.0	[0, 0.1]	0.169	0.208	0.256	0.303	0.521	{n = 40, R = 4}	{n = 20, R = 2}
0.10	5.0	[0.9, 1]	0.161	0.204	0.248	0.329	0.527	{n = 40, R = 4}	{n = 20, R = 2}
0.10	7.5	[0, 0.1]	0.746	0.940	1.135	1.351	1.946	{n = 40, R = 4}	{n = 20, R = 2}
0.10	7.5	[0.9, 1]	0.757	0.932	1.101	1.336	2.097	{n = 40, R = 4}	{n = 20, R = 2}
0.10	10.0	[0, 0.1]	2.498	2.892	3.387	3.982	5.764	{n = 40, R = 4}	{n = 20, R = 2}
0.10	10.0	[0.9, 1]	2.333	2.857	3.373	4.020	6.080	{n = 40, R = 4}	{n = 20, R = 2}
0.15	1.0	[0, 0.15]	0.009	0.014	0.019	0.037	0.075	{n = 40, R = 4}	{n = 20, R = 2}
0.15	1.0	[0.85, 1]	0.009	0.014	0.019	0.037	0.073	{n = 40, R = 4}	{n = 20, R = 2}
0.15	2.0	[0, 0.15]	0.015	0.023	0.030	0.052	0.106	{n = 40, R = 4}	{n = 20, R = 2}
0.15	2.0	[0.85, 1]	0.015	0.023	0.030	0.050	0.105	{n = 40, R = 4}	{n = 20, R = 2}
0.15	3.0	[0, 0.15]	0.037	0.050	0.067	0.090	0.177	{n = 40, R = 4}	{n = 20, R = 2}
0.15	3.0	[0.85, 1]	0.038	0.051	0.067	0.093	0.176	{n = 40, R = 4}	{n = 20, R = 2}
0.15	5.0	[0, 0.15]	0.181	0.233	0.297	0.361	0.582	{n = 40, R = 4}	{n = 20, R = 2}
0.15	5.0	[0.85, 1]	0.184	0.235	0.294	0.361	0.612	{n = 40, R = 4}	{n = 20, R = 2}
0.15	7.5	[0, 0.15]	0.918	1.106	1.307	1.565	2.306	{n = 40, R = 4}	{n = 20, R = 2}
0.15	7.5	[0.85, 1]	0.870	1.087	1.231	1.553	2.453	{n = 40, R = 4}	{n = 20, R = 2}
0.15	10.0	[0, 0.15]	3.075	3.634	4.003	4.784	7.160	{n = 40, R = 4}	{n = 20, R = 2}
0.15	10.0	[0.85, 1]	2.873	3.537	4.033	4.776	7.117	{n = 40, R = 4}	{n = 20, R = 2}
0.20	1.0	[0, 0.2]	0.009	0.015	0.019	0.037	0.081	{n = 40, R = 4}	{n = 20, R = 2}
0.20	1.0	[0.8, 1]	0.009	0.015	0.020	0.037	0.076	{n = 40, R = 4}	{n = 20, R = 2}
0.20	2.0	[0, 0.2]	0.017	0.024	0.031	0.052	0.110	{n = 40, R = 4}	{n = 20, R = 2}
0.20	2.0	[0.8, 1]	0.017	0.025	0.032	0.054	0.110	{n = 40, R = 4}	{n = 20, R = 2}
0.20	3.0	[0, 0.2]	0.037	0.051	0.069	0.095	0.182	{n = 40, R = 4}	{n = 20, R = 2}
0.20	3.0	[0.8, 1]	0.039	0.052	0.071	0.095	0.183	{n = 40, R = 4}	{n = 20, R = 2}

0.20	5.0	[0, 0.2]	0.207	0.255	0.312	0.388	0.620	{n = 40, R = 4}	{n = 20, R = 2}
0.20	5.0	[0.8, 1]	0.203	0.252	0.327	0.391	0.646	{n = 40, R = 4}	{n = 20, R = 2}
0.20	7.5	[0, 0.2]	1.046	1.279	1.431	1.669	2.508	{n = 40, R = 4}	{n = 20, R = 2}
0.20	7.5	[0.8, 1]	0.987	1.258	1.340	1.747	2.583	{n = 40, R = 4}	{n = 20, R = 2}
0.20	10.0	[0, 0.2]	3.837	4.147	4.775	5.415	8.147	{n = 40, R = 4}	{n = 20, R = 2}
0.20	10.0	[0.8, 1]	3.435	3.944	4.567	5.368	8.017	{n = 40, R = 4}	{n = 20, R = 2}
0.25	1.0	[0, 0.25]	0.010	0.015	0.019	0.037	0.079	{n = 40, R = 4}	{n = 20, R = 2}
0.25	1.0	[0.75, 1]	0.009	0.015	0.020	0.037	0.076	{n = 40, R = 4}	{n = 20, R = 2}
0.25	2.0	[0, 0.25]	0.017	0.026	0.032	0.055	0.109	{n = 40, R = 4}	{n = 20, R = 2}
0.25	2.0	[0.75, 1]	0.016	0.026	0.033	0.054	0.110	{n = 40, R = 4}	{n = 20, R = 2}
0.25	3.0	[0, 0.25]	0.041	0.054	0.070	0.097	0.194	{n = 40, R = 4}	{n = 20, R = 2}
0.25	3.0	[0.75, 1]	0.040	0.055	0.069	0.101	0.191	{n = 40, R = 4}	{n = 20, R = 2}
0.25	5.0	[0, 0.25]	0.224	0.262	0.343	0.406	0.650	{n = 35, R = 4}	{n = 20, R = 2}
0.25	5.0	[0.75, 1]	0.219	0.265	0.337	0.393	0.659	{n = 40, R = 4}	{n = 20, R = 2}
0.25	7.5	[0, 0.25]	1.186	1.338	1.527	1.759	2.686	{n = 40, R = 4}	{n = 20, R = 2}
0.25	7.5	[0.75, 1]	1.150	1.322	1.503	1.791	2.657	{n = 40, R = 4}	{n = 20, R = 2}
0.25	10.0	[0, 0.25]	4.217	4.599	5.360	5.961	8.081	{n = 40, R = 4}	{n = 20, R = 2}
0.25	10.0	[0.75, 1]	3.865	4.524	4.891	5.908	9.012	{n = 40, R = 4}	{n = 20, R = 2}
0.30	1.0	[0, 0.3]	0.009	0.015	0.020	0.037	0.075	{n = 40, R = 4}	{n = 20, R = 2}
0.30	1.0	[0.7, 1]	0.010	0.015	0.020	0.037	0.081	{n = 40, R = 4}	{n = 20, R = 2}
0.30	2.0	[0, 0.3]	0.017	0.025	0.033	0.055	0.110	{n = 40, R = 4}	{n = 20, R = 2}
0.30	2.0	[0.7, 1]	0.018	0.026	0.032	0.056	0.109	{n = 40, R = 4}	{n = 20, R = 2}
0.30	3.0	[0, 0.3]	0.040	0.057	0.069	0.099	0.189	{n = 40, R = 4}	{n = 20, R = 2}
0.30	3.0	[0.7, 1]	0.042	0.055	0.070	0.099	0.180	{n = 40, R = 4}	{n = 20, R = 2}
0.30	5.0	[0, 0.3]	0.233	0.291	0.323	0.405	0.655	{n = 40, R = 4}	{n = 20, R = 2}
0.30	5.0	[0.7, 1]	0.240	0.283	0.326	0.408	0.637	{n = 40, R = 4}	{n = 20, R = 2}
0.30	7.5	[0, 0.3]	1.280	1.460	1.704	1.825	2.591	{n = 40, R = 4}	{n = 20, R = 2}
0.30	7.5	[0.7, 1]	1.262	1.411	1.594	1.806	2.784	{n = 40, R = 4}	{n = 20, R = 2}
0.30	10.0	[0, 0.3]	4.856	5.154	5.512	6.306	8.915	{n = 35, R = 4}	{n = 20, R = 2}
0.30	10.0	[0.7, 1]	4.530	4.990	5.460	6.111	8.748	{n = 35, R = 4}	{n = 20, R = 2}
**Skewness**									
0.00	0.0		0.396	0.462	0.538	0.758	1.138	{n = 40, R = 4}	{n = 20, R = 2}
0.00	0.0		0.410	0.460	0.529	0.795	1.185	{n = 35, R = 4}	{n = 20, R = 2}
0.05	1.0	[0, 0.05]	0.354	0.532	0.550	0.826	1.321	{n = 35, R = 4}	{n = 20, R = 2}
0.05	1.0	[0.95, 1]	0.402	0.513	0.563	0.783	1.189	{n = 40, R = 4}	{n = 20, R = 2}
0.05	2.0	[0, 0.05]	0.627	0.686	0.785	0.878	1.211	{n = 40, R = 4}	{n = 20, R = 2}
0.05	2.0	[0.95, 1]	0.571	0.681	0.769	0.879	1.238	{n = 40, R = 4}	{n = 20, R = 2}
0.05	3.0	[0, 0.05]	0.798	0.945	1.060	1.292	1.574	{n = 40, R = 4}	{n = 20, R = 2}
0.05	3.0	[0.95, 1]	0.849	0.998	1.066	1.235	1.499	{n = 40, R = 4}	{n = 20, R = 2}
0.05	5.0	[0, 0.05]	1.081	1.213	1.418	1.632	1.981	{n = 40, R = 3}	{n = 20, R = 2}
0.05	5.0	[0.95, 1]	1.057	1.243	1.348	1.486	1.726	{n = 40, R = 4}	{n = 20, R = 2}
0.05	7.5	[0, 0.05]	1.264	1.328	1.502	1.697	2.098	{n = 35, R = 4}	{n = 20, R = 2}
0.05	7.5	[0.95, 1]	1.104	1.270	1.446	1.581	1.966	{n = 40, R = 4}	{n = 20, R = 2}

0.05	10.0	[0, 0.05]	1.372	1.473	1.643	1.849	2.418	{n = 40, R = 4}	{n = 20, R = 2}
0.05	10.0	[0.95, 1]	1.151	1.314	1.447	1.672	2.082	{n = 35, R = 3}	{n = 20, R = 2}
0.10	1.0	[0, 0.1]	0.467	0.517	0.559	0.845	1.083	{n = 35, R = 4}	{n = 20, R = 2}
0.10	1.0	[0.9, 1]	0.463	0.541	0.593	0.808	1.125	{n = 40, R = 4}	{n = 20, R = 2}
0.10	2.0	[0, 0.1]	0.674	0.721	0.803	0.887	1.296	{n = 25, R = 4}	{n = 20, R = 2}
0.10	2.0	[0.9, 1]	0.650	0.720	0.812	0.861	1.348	{n = 40, R = 4}	{n = 20, R = 2}
0.10	3.0	[0, 0.1]	0.737	0.923	1.000	1.100	1.425	{n = 35, R = 4}	{n = 20, R = 2}
0.10	3.0	[0.9, 1]	0.705	0.896	1.003	1.091	1.520	{n = 40, R = 3}	{n = 20, R = 2}
0.10	5.0	[0, 0.1]	0.912	1.089	1.182	1.251	1.582	{n = 35, R = 4}	{n = 20, R = 2}
0.10	5.0	[0.9, 1]	0.887	1.005	1.151	1.374	1.653	{n = 40, R = 4}	{n = 20, R = 2}
0.10	7.5	[0, 0.1]	1.012	1.091	1.236	1.437	1.731	{n = 35, R = 4}	{n = 20, R = 2}
0.10	7.5	[0.9, 1]	0.954	0.995	1.067	1.225	1.802	{n = 40, R = 4}	{n = 20, R = 2}
0.10	10.0	[0, 0.1]	1.139	1.233	1.287	1.475	1.959	{n = 40, R = 3}	{n = 20, R = 2}
0.10	10.0	[0.9, 1]	0.934	1.050	1.246	1.349	1.823	{n = 40, R = 4}	{n = 20, R = 2}
0.15	1.0	[0, 0.15]	0.507	0.539	0.597	0.781	1.231	{n = 25, R = 4}	{n = 20, R = 2}
0.15	1.0	[0.85, 1]	0.419	0.536	0.642	0.763	1.074	{n = 40, R = 4}	{n = 20, R = 2}
0.15	2.0	[0, 0.15]	0.708	0.760	0.833	0.897	1.423	{n = 35, R = 3}	{n = 20, R = 2}
0.15	2.0	[0.85, 1]	0.666	0.760	0.840	0.917	1.386	{n = 40, R = 4}	{n = 20, R = 2}
0.15	3.0	[0, 0.15]	0.874	0.965	1.060	1.121	1.409	{n = 30, R = 4}	{n = 20, R = 2}
0.15	3.0	[0.85, 1]	0.796	0.925	0.999	1.142	1.482	{n = 35, R = 4}	{n = 20, R = 2}
0.15	5.0	[0, 0.15]	0.936	1.056	1.188	1.266	1.471	{n = 30, R = 4}	{n = 20, R = 3}
0.15	5.0	[0.85, 1]	0.945	1.026	1.100	1.261	1.481	{n = 40, R = 3}	{n = 20, R = 2}
0.15	7.5	[0, 0.15]	1.010	1.120	1.206	1.314	1.714	{n = 40, R = 4}	{n = 20, R = 2}
0.15	7.5	[0.85, 1]	0.893	1.050	1.077	1.342	1.615	{n = 35, R = 4}	{n = 20, R = 2}
0.15	10.0	[0, 0.15]	1.053	1.142	1.262	1.411	1.663	{n = 40, R = 4}	{n = 20, R = 2}
0.15	10.0	[0.85, 1]	1.020	1.150	1.271	1.386	1.569	{n = 40, R = 4}	{n = 20, R = 2}
0.20	1.0	[0, 0.2]	0.549	0.577	0.626	0.828	1.605	{n = 30, R = 4}	{n = 20, R = 2}
0.20	1.0	[0.8, 1]	0.545	0.580	0.597	0.828	1.131	{n = 40, R = 4}	{n = 25, R = 2}
0.20	2.0	[0, 0.2]	0.744	0.812	0.893	0.991	1.182	{n = 30, R = 3}	{n = 20, R = 2}
0.20	2.0	[0.8, 1]	0.787	0.857	0.909	0.981	1.303	{n = 30, R = 4}	{n = 20, R = 2}
0.20	3.0	[0, 0.2]	0.994	1.043	1.064	1.310	1.478	{n = 35, R = 3}	{n = 25, R = 2}
0.20	3.0	[0.8, 1]	1.013	1.094	1.150	1.217	1.450	{n = 30, R = 4}	{n = 20, R = 2}
0.20	5.0	[0, 0.2]	1.150	1.244	1.321	1.390	1.599	{n = 30, R = 4}	{n = 25, R = 2}
0.20	5.0	[0.8, 1]	1.141	1.200	1.307	1.362	1.816	{n = 40, R = 4}	{n = 20, R = 3}
0.20	7.5	[0, 0.2]	1.095	1.280	1.436	1.506	1.638	{n = 40, R = 4}	{n = 20, R = 2}
0.20	7.5	[0.8, 1]	1.080	1.181	1.255	1.485	1.737	{n = 40, R = 4}	{n = 20, R = 2}
0.20	10.0	[0, 0.2]	1.225	1.277	1.416	1.514	1.934	{n = 35, R = 4}	{n = 20, R = 2}
0.20	10.0	[0.8, 1]	1.099	1.265	1.382	1.503	1.875	{n = 40, R = 4}	{n = 20, R = 3}
0.25	1.0	[0, 0.25]	0.478	0.596	0.650	0.790	1.162	{n = 25, R = 4}	{n = 20, R = 2}
0.25	1.0	[0.75, 1]	0.555	0.586	0.619	0.803	1.097	{n = 25, R = 4}	{n = 20, R = 2}
0.25	2.0	[0, 0.25]	0.865	0.951	1.026	1.056	1.294	{n = 30, R = 4}	{n = 20, R = 2}
0.25	2.0	[0.75, 1]	0.791	0.929	1.003	1.088	1.375	{n = 35, R = 3}	{n = 20, R = 2}
0.25	3.0	[0, 0.25]	1.133	1.214	1.369	1.431	1.633	{n = 35, R = 2}	{n = 25, R = 4}

0.25	3.0	[0.75, 1]	1.159	1.278	1.342	1.412	1.614	{n = 40, R = 2}	{n = 20, R = 2}
0.25	5.0	[0, 0.25]	1.295	1.554	1.631	1.673	1.828	{n = 40, R = 3}	{n = 20, R = 2}
0.25	5.0	[0.75, 1]	1.370	1.470	1.602	1.701	1.920	{n = 20, R = 3}	{n = 20, R = 4}
0.25	7.5	[0, 0.25]	1.343	1.483	1.545	1.618	1.915	{n = 40, R = 3}	{n = 25, R = 2}
0.25	7.5	[0.75, 1]	1.417	1.442	1.551	1.703	1.912	{n = 25, R = 3}	{n = 20, R = 2}
0.25	10.0	[0, 0.25]	1.272	1.412	1.501	1.598	2.012	{n = 35, R = 3}	{n = 20, R = 2}
0.25	10.0	[0.75, 1]	1.294	1.440	1.520	1.668	2.093	{n = 40, R = 4}	{n = 20, R = 2}
0.30	1.0	[0, 0.3]	0.611	0.654	0.696	0.839	1.026	{n = 40, R = 4}	{n = 20, R = 2}
0.30	1.0	[0.7, 1]	0.519	0.602	0.678	0.803	1.405	{n = 40, R = 3}	{n = 20, R = 2}
0.30	2.0	[0, 0.3]	0.969	1.025	1.074	1.157	1.305	{n = 35, R = 3}	{n = 35, R = 4}
0.30	2.0	[0.7, 1]	0.947	1.048	1.112	1.170	1.321	{n = 25, R = 3}	{n = 40, R = 4}
0.30	3.0	[0, 0.3]	1.324	1.417	1.492	1.624	1.804	{n = 35, R = 2}	{n = 35, R = 4}
0.30	3.0	[0.7, 1]	1.246	1.433	1.522	1.613	2.048	{n = 30, R = 2}	{n = 35, R = 4}
0.30	5.0	[0, 0.3]	1.721	1.810	1.830	1.897	2.335	{n = 40, R = 2}	{n = 30, R = 4}
0.30	5.0	[0.7, 1]	1.634	1.832	1.883	1.930	2.203	{n = 30, R = 2}	{n = 20, R = 2}
0.30	7.5	[0, 0.3]	1.612	1.791	1.889	1.936	2.094	{n = 35, R = 4}	{n = 40, R = 2}
0.30	7.5	[0.7, 1]	1.545	1.709	1.833	1.958	2.146	{n = 40, R = 3}	{n = 20, R = 3}
0.30	10.0	[0, 0.3]	1.467	1.644	1.772	1.828	2.507	{n = 40, R = 2}	{n = 20, R = 2}
0.30	10.0	[0.7, 1]	1.569	1.706	1.842	1.926	2.143	{n = 35, R = 4}	{n = 20, R = 2}
**Kurtosis**									
0.00	0.0		3.588	3.687	3.760	4.284	6.043	{n = 40, R = 4}	{n = 20, R = 2}
0.00	0.0		3.529	3.680	3.719	4.386	6.464	{n = 35, R = 4}	{n = 20, R = 2}
0.05	1.0	[0, 0.05]	3.537	3.764	3.900	4.632	7.755	{n = 35, R = 4}	{n = 20, R = 2}
0.05	1.0	[0.95, 1]	3.553	3.685	3.840	4.295	6.257	{n = 40, R = 4}	{n = 20, R = 2}
0.05	2.0	[0, 0.05]	3.823	4.122	4.387	4.724	6.050	{n = 40, R = 3}	{n = 20, R = 2}
0.05	2.0	[0.95, 1]	3.703	4.059	4.354	4.682	6.513	{n = 40, R = 4}	{n = 20, R = 2}
0.05	3.0	[0, 0.05]	4.086	4.561	4.916	6.069	9.475	{n = 40, R = 4}	{n = 30, R = 2}
0.05	3.0	[0.95, 1]	4.212	4.875	5.286	6.099	7.873	{n = 40, R = 4}	{n = 20, R = 2}
0.05	5.0	[0, 0.05]	4.599	5.259	5.755	7.058	10.284	{n = 40, R = 3}	{n = 20, R = 2}
0.05	5.0	[0.95, 1]	4.399	5.419	5.745	6.179	7.934	{n = 40, R = 4}	{n = 20, R = 2}
0.05	7.5	[0, 0.05]	4.792	5.660	6.197	6.744	9.428	{n = 30, R = 4}	{n = 20, R = 2}
0.05	7.5	[0.95, 1]	4.521	5.170	5.820	6.614	8.411	{n = 40, R = 4}	{n = 20, R = 2}
0.05	10.0	[0, 0.05]	5.514	5.744	6.596	7.851	12.764	{n = 40, R = 4}	{n = 20, R = 2}
0.05	10.0	[0.95, 1]	4.301	5.387	6.014	6.857	9.227	{n = 35, R = 3}	{n = 20, R = 2}
0.10	1.0	[0, 0.1]	3.602	3.752	3.922	4.812	5.609	{n = 25, R = 3}	{n = 20, R = 2}
0.10	1.0	[0.9, 1]	3.712	3.865	4.027	4.450	5.665	{n = 40, R = 4}	{n = 20, R = 2}
0.10	2.0	[0, 0.1]	4.004	4.249	4.549	5.020	6.821	{n = 25, R = 4}	{n = 20, R = 2}
0.10	2.0	[0.9, 1]	4.008	4.190	4.469	4.618	7.063	{n = 35, R = 3}	{n = 20, R = 2}
0.10	3.0	[0, 0.1]	4.033	4.790	5.072	5.409	6.743	{n = 35, R = 4}	{n = 20, R = 2}
0.10	3.0	[0.9, 1]	3.780	4.569	5.112	5.191	7.300	{n = 40, R = 3}	{n = 20, R = 2}
0.10	5.0	[0, 0.1]	4.598	5.330	5.538	6.206	6.955	{n = 35, R = 4}	{n = 20, R = 2}
0.10	5.0	[0.9, 1]	4.420	5.167	5.662	6.722	8.480	{n = 30, R = 3}	{n = 20, R = 4}
0.10	7.5	[0, 0.1]	4.641	5.107	5.423	7.139	9.278	{n = 35, R = 3}	{n = 25, R = 2}

0.10	7.5	[0.9, 1]	4.483	4.800	4.899	5.586	9.777	{n = 30, R = 4}	{n = 20, R = 2}
0.10	10.0	[0, 0.1]	4.894	5.287	5.744	6.662	10.180	{n = 35, R = 3}	{n = 20, R = 2}
0.10	10.0	[0.9, 1]	4.495	4.954	5.698	6.093	10.028	{n = 40, R = 3}	{n = 20, R = 2}
0.15	1.0	[0, 0.15]	3.711	3.881	3.962	4.267	6.617	{n = 20, R = 4}	{n = 20, R = 2}
0.15	1.0	[0.85, 1]	3.574	3.882	4.019	4.538	5.389	{n = 40, R = 4}	{n = 20, R = 2}
0.15	2.0	[0, 0.15]	4.119	4.349	4.710	4.800	8.309	{n = 35, R = 3}	{n = 20, R = 2}
0.15	2.0	[0.85, 1]	4.236	4.341	4.543	5.436	7.569	{n = 40, R = 3}	{n = 20, R = 2}
0.15	3.0	[0, 0.15]	4.493	5.206	5.768	6.024	6.603	{n = 30, R = 3}	{n = 20, R = 2}
0.15	3.0	[0.85, 1]	4.382	5.055	5.336	6.285	8.170	{n = 35, R = 4}	{n = 20, R = 2}
0.15	5.0	[0, 0.15]	4.780	5.730	6.230	6.755	7.987	{n = 30, R = 4}	{n = 20, R = 3}
0.15	5.0	[0.85, 1]	5.185	5.400	6.043	6.676	7.603	{n = 40, R = 2}	{n = 25, R = 2}
0.15	7.5	[0, 0.15]	5.010	5.629	5.961	6.232	8.633	{n = 40, R = 3}	{n = 20, R = 2}
0.15	7.5	[0.85, 1]	4.833	5.313	5.671	6.861	8.567	{n = 35, R = 4}	{n = 25, R = 3}
0.15	10.0	[0, 0.15]	4.849	5.245	5.844	6.322	8.320	{n = 30, R = 3}	{n = 25, R = 2}
0.15	10.0	[0.85, 1]	5.196	5.855	6.340	7.054	7.387	{n = 25, R = 4}	{n = 20, R = 2}
0.20	1.0	[0, 0.2]	3.654	4.006	4.191	4.605	10.756	{n = 35, R = 3}	{n = 20, R = 2}
0.20	1.0	[0.8, 1]	3.691	3.933	4.098	4.704	6.270	{n = 25, R = 3}	{n = 25, R = 2}
0.20	2.0	[0, 0.2]	4.029	4.686	5.199	5.531	6.315	{n = 40, R = 2}	{n = 20, R = 3}
0.20	2.0	[0.8, 1]	4.450	4.767	5.093	5.717	6.716	{n = 35, R = 2}	{n = 40, R = 4}
0.20	3.0	[0, 0.2]	5.036	5.918	6.168	6.876	8.725	{n = 35, R = 2}	{n = 25, R = 2}
0.20	3.0	[0.8, 1]	5.449	5.754	6.825	6.963	7.746	{n = 25, R = 3}	{n = 20, R = 4}
0.20	5.0	[0, 0.2]	6.341	6.900	7.342	7.548	8.792	{n = 30, R = 4}	{n = 25, R = 2}
0.20	5.0	[0.8, 1]	6.171	6.611	7.205	7.563	13.046	{n = 30, R = 4}	{n = 20, R = 3}
0.20	7.5	[0, 0.2]	5.840	6.431	7.379	7.904	9.927	{n = 40, R = 4}	{n = 40, R = 3}
0.20	7.5	[0.8, 1]	5.966	6.257	7.038	8.065	10.445	{n = 40, R = 2}	{n = 35, R = 3}
0.20	10.0	[0, 0.2]	5.627	6.251	6.539	7.778	10.440	{n = 30, R = 3}	{n = 20, R = 2}
0.20	10.0	[0.8, 1]	5.514	6.463	6.824	8.120	12.649	{n = 35, R = 3}	{n = 20, R = 3}
0.25	1.0	[0, 0.25]	3.618	4.165	4.271	4.625	5.624	{n = 25, R = 4}	{n = 20, R = 2}
0.25	1.0	[0.75, 1]	3.882	4.029	4.111	4.471	5.553	{n = 40, R = 3}	{n = 25, R = 2}
0.25	2.0	[0, 0.25]	5.047	5.217	5.372	6.367	7.012	{n = 30, R = 4}	{n = 40, R = 4}
0.25	2.0	[0.75, 1]	4.460	5.100	5.260	6.144	7.642	{n = 35, R = 3}	{n = 20, R = 2}
0.25	3.0	[0, 0.25]	5.858	6.159	7.548	8.612	11.370	{n = 20, R = 3}	{n = 25, R = 4}
0.25	3.0	[0.75, 1]	5.845	7.335	7.746	8.475	10.345	{n = 30, R = 2}	{n = 35, R = 4}
0.25	5.0	[0, 0.25]	7.239	8.708	9.473	10.349	12.707	{n = 40, R = 3}	{n = 30, R = 3}
0.25	5.0	[0.75, 1]	6.779	8.498	9.538	10.427	13.132	{n = 20, R = 3}	{n = 30, R = 4}
0.25	7.5	[0, 0.25]	6.931	7.542	8.003	8.925	11.013	{n = 40, R = 3}	{n = 25, R = 2}
0.25	7.5	[0.75, 1]	7.294	7.769	8.840	9.978	11.199	{n = 25, R = 3}	{n = 20, R = 3}
0.25	10.0	[0, 0.25]	5.968	6.694	7.201	7.709	11.319	{n = 35, R = 3}	{n = 20, R = 2}
0.25	10.0	[0.75, 1]	6.413	7.099	8.053	9.084	11.892	{n = 30, R = 3}	{n = 20, R = 2}
0.30	1.0	[0, 0.3]	4.173	4.252	4.443	4.650	5.264	{n = 40, R = 2}	{n = 20, R = 3}
0.30	1.0	[0.7, 1]	3.580	4.079	4.210	4.463	9.547	{n = 40, R = 3}	{n = 20, R = 2}
0.30	2.0	[0, 0.3]	5.046	5.635	5.888	6.555	7.914	{n = 35, R = 2}	{n = 35, R = 4}
0.30	2.0	[0.7, 1]	5.134	5.826	6.066	6.866	7.909	{n = 40, R = 2}	{n = 40, R = 4}

0.30	3.0	[0, 0.3]	6.974	7.837	8.323	9.811	11.930	{n = 35, R = 2}	{n = 35, R = 4}
0.30	3.0	[0.7, 1]	6.312	7.807	9.006	10.744	14.304	{n = 30, R = 2}	{n = 35, R = 4}
0.30	5.0	[0, 0.3]	9.488	10.261	10.773	11.308	15.960	{n = 25, R = 4}	{n = 35, R = 4}
0.30	5.0	[0.7, 1]	8.820	10.470	11.317	11.739	16.514	{n = 30, R = 2}	{n = 20, R = 2}
0.30	7.5	[0, 0.3]	8.370	9.353	10.227	11.165	13.637	{n = 35, R = 4}	{n = 40, R = 2}
0.30	7.5	[0.7, 1]	8.173	9.213	10.001	11.409	12.459	{n = 40, R = 3}	{n = 20, R = 3}
0.30	10.0	[0, 0.3]	6.760	7.790	8.622	9.205	17.723	{n = 40, R = 2}	{n = 20, R = 2}
0.30	10.0	[0.7, 1]	7.431	9.010	9.561	10.332	11.535	{n = 35, R = 4}	{n = 25, R = 3}

Table 10. Regarding the distribution summary statistics (minimum, first quartile, median, third quartile and maximum, respectively) of the mean, variance, skewness and kurtosis of the differences in non-selectivity (DINS) estimator, $\hat{\zeta}$: what is the empirical probability of lower systematic DINS producing larger distribution summary statistic values than upper systematic DINS across all combinations quantile interval ranges, $u - l$, and maximum relocation magnitude multipliers, $m_{\max}$? Probabilities in this table are given in percent.

	Distribution summary statistics				
**Moment**	**Min. %**	**First quartile %**	**Median %**	**Third quartile %**	**Max. %**
mean	21.622	24.324	18.919	21.622	37.838
variance	56.757	62.162	45.946	51.351	51.351
skewness	67.568	62.162	56.757	56.757	48.649
kurtosis	56.757	64.865	56.757	40.541	48.649

Table 11. Regarding the distribution summary statistics (minimum, first quartile, median, third quartile and maximum, respectively) of the mean, variance, skewness and kurtosis of the differences in non-selectivity (DINS) estimator, $\hat{\zeta}$: what is the mean relative difference between the distribution summary statistic values produced by lower and upper systematic DINS, across all combinations of quantile interval ranges, $u - l$, and maximum relocation magnitude multipliers, $m_{\max}$? Mean relative differences in this table are given in percent.

	Distribution summary statistics				
**Moment**	**Min. %**	**First quartile %**	**Median %**	**Third quartile %**	**Max. %**
mean	-1.000	-0.900	-1.034	-0.964	-0.921
variance	1.456	0.524	0.443	-0.649	-0.880
skewness	3.643	2.037	1.948	1.417	1.350
kurtosis	1.769	0.343	-0.166	-0.259	3.342

Table 12. Distribution summary statistics of the 1%, 2.5%, 5%, 10%, 25%, 50%, 75%, 90%, 95%, 97.5%, and 99% percentiles of the differences in non-selectivity (DINS) estimator, $\hat{\zeta}$, for all combinations of quantile interval ranges, $u - l = 0,\ 0.05,\ 0.15,$ and 0.30, and maximum relocation magnitude multipliers, $m_{\max} = 0,\ 5,\ 7.5,$ and 10, across all 15 considered study designs. 'Min. PSD' and 'Max. PSD' are short for 'Minimize percentile study design' and 'Maximize percentile study design', respectively. Furthermore, 'Min.', 'Q1', 'Med.', 'Q3' and 'Max.' refers to Minimum, First quartile, Median, Third quartile and Maximum, respectively.

$u - l$	$m_{\max}$	**q**	**Min.**	**Q1**	**Med.**	**Q3**	**Max.**	**Min. PSD**	**Max. PSD**

**1%**									
0.00	0.0		0.649	0.720	0.775	0.800	0.834	{n = 20, R = 2}	{n = 40, R = 4}
0.00	0.0		0.646	0.720	0.773	0.800	0.837	{n = 20, R = 2}	{n = 40, R = 4}
0.05	5.0	[0, 0.05]	0.679	0.773	0.813	0.849	0.888	{n = 20, R = 2}	{n = 40, R = 4}
0.05	5.0	[0.95, 1]	0.687	0.773	0.818	0.850	0.891	{n = 20, R = 2}	{n = 40, R = 4}
0.05	7.5	[0, 0.05]	0.683	0.771	0.817	0.857	0.894	{n = 20, R = 2}	{n = 40, R = 4}
0.05	7.5	[0.95, 1]	0.701	0.781	0.815	0.854	0.895	{n = 20, R = 2}	{n = 40, R = 4}
0.05	10.0	[0, 0.05]	0.696	0.785	0.821	0.855	0.893	{n = 20, R = 2}	{n = 40, R = 4}
0.05	10.0	[0.95, 1]	0.704	0.787	0.822	0.859	0.900	{n = 20, R = 2}	{n = 40, R = 4}
0.15	5.0	[0, 0.15]	0.771	0.879	0.911	0.947	0.991	{n = 20, R = 2}	{n = 40, R = 4}
0.15	5.0	[0.85, 1]	0.764	0.877	0.912	0.952	0.984	{n = 20, R = 2}	{n = 40, R = 4}
0.15	7.5	[0, 0.15]	0.807	0.902	0.938	0.966	1.006	{n = 20, R = 2}	{n = 40, R = 4}
0.15	7.5	[0.85, 1]	0.794	0.906	0.948	0.980	1.014	{n = 20, R = 2}	{n = 40, R = 4}
0.15	10.0	[0, 0.15]	0.818	0.917	0.957	0.981	1.019	{n = 20, R = 2}	{n = 40, R = 4}
0.15	10.0	[0.85, 1]	0.822	0.927	0.955	0.990	1.024	{n = 20, R = 2}	{n = 40, R = 3}
0.30	5.0	[0, 0.3]	0.872	0.946	0.986	1.007	1.027	{n = 20, R = 2}	{n = 30, R = 4}
0.30	5.0	[0.7, 1]	0.879	0.956	0.984	1.002	1.020	{n = 20, R = 2}	{n = 40, R = 4}
0.30	7.5	[0, 0.3]	0.923	0.989	1.001	1.014	1.039	{n = 20, R = 2}	{n = 40, R = 4}
0.30	7.5	[0.7, 1]	0.922	0.984	0.999	1.016	1.033	{n = 20, R = 2}	{n = 40, R = 4}
0.30	10.0	[0, 0.3]	0.950	0.994	1.007	1.019	1.038	{n = 20, R = 2}	{n = 30, R = 3}
0.30	10.0	[0.7, 1]	0.965	0.987	1.017	1.020	1.032	{n = 20, R = 2}	{n = 35, R = 4}
**2.5%**									
0.00	0.0		0.701	0.762	0.813	0.835	0.865	{n = 20, R = 2}	{n = 40, R = 4}
0.00	0.0		0.701	0.762	0.811	0.835	0.866	{n = 20, R = 2}	{n = 40, R = 4}
0.05	5.0	[0, 0.05]	0.742	0.821	0.854	0.890	0.923	{n = 20, R = 2}	{n = 40, R = 4}
0.05	5.0	[0.95, 1]	0.748	0.823	0.860	0.890	0.924	{n = 20, R = 2}	{n = 40, R = 4}
0.05	7.5	[0, 0.05]	0.749	0.830	0.860	0.897	0.930	{n = 20, R = 2}	{n = 40, R = 4}
0.05	7.5	[0.95, 1]	0.762	0.838	0.868	0.898	0.931	{n = 20, R = 2}	{n = 40, R = 4}
0.05	10.0	[0, 0.05]	0.765	0.836	0.866	0.900	0.928	{n = 20, R = 2}	{n = 40, R = 4}
0.05	10.0	[0.95, 1]	0.757	0.839	0.864	0.902	0.938	{n = 20, R = 2}	{n = 40, R = 4}
0.15	5.0	[0, 0.15]	0.855	0.945	0.978	1.016	1.050	{n = 20, R = 2}	{n = 40, R = 4}
0.15	5.0	[0.85, 1]	0.835	0.947	0.979	1.017	1.045	{n = 20, R = 2}	{n = 40, R = 4}
0.15	7.5	[0, 0.15]	0.891	0.978	1.024	1.071	1.097	{n = 20, R = 2}	{n = 40, R = 4}
0.15	7.5	[0.85, 1]	0.886	0.983	1.043	1.076	1.110	{n = 20, R = 2}	{n = 40, R = 4}
0.15	10.0	[0, 0.15]	0.918	1.001	1.057	1.088	1.139	{n = 20, R = 2}	{n = 40, R = 2}
0.15	10.0	[0.85, 1]	0.929	1.004	1.064	1.109	1.158	{n = 20, R = 2}	{n = 40, R = 3}
0.30	5.0	[0, 0.3]	0.962	1.043	1.062	1.106	1.130	{n = 20, R = 2}	{n = 40, R = 3}
0.30	5.0	[0.7, 1]	0.967	1.045	1.079	1.093	1.123	{n = 20, R = 2}	{n = 40, R = 4}
0.30	7.5	[0, 0.3]	1.048	1.121	1.142	1.164	1.196	{n = 20, R = 2}	{n = 40, R = 2}
0.30	7.5	[0.7, 1]	1.065	1.128	1.143	1.162	1.194	{n = 20, R = 2}	{n = 40, R = 3}
0.30	10.0	[0, 0.3]	1.107	1.144	1.168	1.182	1.201	{n = 20, R = 4}	{n = 35, R = 2}
0.30	10.0	[0.7, 1]	1.123	1.145	1.163	1.178	1.213	{n = 20, R = 3}	{n = 35, R = 2}

**5%**									
0.00	0.0		0.746	0.799	0.846	0.865	0.890	{n = 20, R = 2}	{n = 40, R = 4}
0.00	0.0		0.750	0.799	0.844	0.864	0.891	{n = 20, R = 2}	{n = 40, R = 4}
0.05	5.0	[0, 0.05]	0.801	0.869	0.892	0.927	0.955	{n = 20, R = 2}	{n = 40, R = 4}
0.05	5.0	[0.95, 1]	0.803	0.868	0.898	0.923	0.954	{n = 20, R = 2}	{n = 40, R = 4}
0.05	7.5	[0, 0.05]	0.810	0.882	0.911	0.934	0.964	{n = 20, R = 2}	{n = 40, R = 4}
0.05	7.5	[0.95, 1]	0.818	0.887	0.918	0.933	0.963	{n = 20, R = 2}	{n = 40, R = 4}
0.05	10.0	[0, 0.05]	0.816	0.889	0.921	0.938	0.966	{n = 20, R = 2}	{n = 40, R = 4}
0.05	10.0	[0.95, 1]	0.821	0.894	0.922	0.941	0.971	{n = 20, R = 2}	{n = 40, R = 4}
0.15	5.0	[0, 0.15]	0.934	1.005	1.046	1.088	1.133	{n = 20, R = 2}	{n = 40, R = 4}
0.15	5.0	[0.85, 1]	0.919	1.014	1.064	1.091	1.126	{n = 20, R = 2}	{n = 40, R = 4}
0.15	7.5	[0, 0.15]	0.993	1.067	1.135	1.190	1.246	{n = 20, R = 2}	{n = 40, R = 4}
0.15	7.5	[0.85, 1]	0.992	1.088	1.149	1.214	1.283	{n = 20, R = 2}	{n = 40, R = 4}
0.15	10.0	[0, 0.15]	1.031	1.120	1.202	1.275	1.349	{n = 20, R = 2}	{n = 40, R = 4}
0.15	10.0	[0.85, 1]	1.046	1.124	1.235	1.320	1.409	{n = 20, R = 2}	{n = 40, R = 3}
0.30	5.0	[0, 0.3]	1.068	1.139	1.178	1.221	1.266	{n = 20, R = 2}	{n = 40, R = 4}
0.30	5.0	[0.7, 1]	1.067	1.145	1.187	1.208	1.258	{n = 20, R = 2}	{n = 40, R = 4}
0.30	7.5	[0, 0.3]	1.225	1.336	1.413	1.425	1.518	{n = 20, R = 2}	{n = 40, R = 4}
0.30	7.5	[0.7, 1]	1.257	1.339	1.389	1.434	1.494	{n = 20, R = 2}	{n = 40, R = 3}
0.30	10.0	[0, 0.3]	1.352	1.476	1.533	1.551	1.581	{n = 20, R = 4}	{n = 40, R = 2}
0.30	10.0	[0.7, 1]	1.394	1.495	1.543	1.588	1.647	{n = 20, R = 3}	{n = 40, R = 4}
**10%**									
0.00	0.0		0.804	0.845	0.884	0.898	0.918	{n = 20, R = 2}	{n = 40, R = 4}
0.00	0.0		0.805	0.844	0.882	0.898	0.918	{n = 20, R = 2}	{n = 40, R = 4}
0.05	5.0	[0, 0.05]	0.875	0.929	0.956	0.971	0.996	{n = 20, R = 2}	{n = 40, R = 4}
0.05	5.0	[0.95, 1]	0.875	0.932	0.951	0.970	0.995	{n = 20, R = 2}	{n = 40, R = 4}
0.05	7.5	[0, 0.05]	0.888	0.947	0.966	0.989	1.010	{n = 20, R = 2}	{n = 40, R = 4}
0.05	7.5	[0.95, 1]	0.895	0.947	0.974	0.992	1.010	{n = 20, R = 2}	{n = 40, R = 4}
0.05	10.0	[0, 0.05]	0.892	0.950	0.974	0.998	1.016	{n = 20, R = 2}	{n = 40, R = 4}
0.05	10.0	[0.95, 1]	0.900	0.955	0.983	1.004	1.024	{n = 20, R = 2}	{n = 40, R = 3}
0.15	5.0	[0, 0.15]	1.043	1.102	1.163	1.205	1.247	{n = 20, R = 2}	{n = 40, R = 4}
0.15	5.0	[0.85, 1]	1.031	1.110	1.175	1.211	1.247	{n = 20, R = 2}	{n = 40, R = 4}
0.15	7.5	[0, 0.15]	1.141	1.235	1.342	1.415	1.480	{n = 20, R = 3}	{n = 40, R = 3}
0.15	7.5	[0.85, 1]	1.148	1.270	1.365	1.462	1.546	{n = 20, R = 4}	{n = 40, R = 4}
0.15	10.0	[0, 0.15]	1.185	1.374	1.493	1.625	1.704	{n = 20, R = 4}	{n = 40, R = 2}
0.15	10.0	[0.85, 1]	1.212	1.425	1.619	1.762	1.871	{n = 20, R = 4}	{n = 40, R = 3}
0.30	5.0	[0, 0.3]	1.204	1.269	1.316	1.352	1.406	{n = 20, R = 2}	{n = 40, R = 4}
0.30	5.0	[0.7, 1]	1.205	1.273	1.324	1.348	1.401	{n = 20, R = 2}	{n = 40, R = 4}
0.30	7.5	[0, 0.3]	1.495	1.623	1.697	1.742	1.811	{n = 20, R = 2}	{n = 40, R = 4}
0.30	7.5	[0.7, 1]	1.530	1.634	1.714	1.784	1.839	{n = 20, R = 2}	{n = 40, R = 3}
0.30	10.0	[0, 0.3]	1.809	1.952	2.020	2.090	2.133	{n = 20, R = 4}	{n = 40, R = 3}
0.30	10.0	[0.7, 1]	1.885	2.040	2.158	2.209	2.325	{n = 20, R = 2}	{n = 40, R = 4}

**25%**									
0.00	0.0		0.911	0.928	0.948	0.957	0.964	{n = 20, R = 2}	{n = 40, R = 4}
0.00	0.0		0.912	0.927	0.947	0.956	0.964	{n = 20, R = 2}	{n = 40, R = 4}
0.05	5.0	[0, 0.05]	1.023	1.044	1.065	1.080	1.094	{n = 20, R = 2}	{n = 40, R = 2}
0.05	5.0	[0.95, 1]	1.028	1.045	1.060	1.077	1.100	{n = 20, R = 2}	{n = 40, R = 2}
0.05	7.5	[0, 0.05]	1.040	1.070	1.094	1.133	1.180	{n = 20, R = 4}	{n = 40, R = 2}
0.05	7.5	[0.95, 1]	1.045	1.070	1.101	1.137	1.192	{n = 20, R = 4}	{n = 40, R = 2}
0.05	10.0	[0, 0.05]	1.052	1.086	1.124	1.174	1.243	{n = 20, R = 4}	{n = 40, R = 2}
0.05	10.0	[0.95, 1]	1.052	1.088	1.132	1.206	1.285	{n = 20, R = 4}	{n = 40, R = 2}
0.15	5.0	[0, 0.15]	1.306	1.348	1.392	1.432	1.457	{n = 20, R = 4}	{n = 40, R = 4}
0.15	5.0	[0.85, 1]	1.302	1.361	1.405	1.436	1.466	{n = 20, R = 2}	{n = 40, R = 4}
0.15	7.5	[0, 0.15]	1.591	1.731	1.821	1.881	1.930	{n = 20, R = 4}	{n = 40, R = 3}
0.15	7.5	[0.85, 1]	1.631	1.800	1.892	1.965	2.030	{n = 20, R = 4}	{n = 40, R = 4}
0.15	10.0	[0, 0.15]	1.910	2.155	2.274	2.379	2.474	{n = 20, R = 4}	{n = 40, R = 2}
0.15	10.0	[0.85, 1]	2.095	2.356	2.516	2.650	2.741	{n = 20, R = 2}	{n = 40, R = 3}
0.30	5.0	[0, 0.3]	1.476	1.524	1.548	1.564	1.596	{n = 20, R = 2}	{n = 40, R = 4}
0.30	5.0	[0.7, 1]	1.484	1.518	1.554	1.577	1.600	{n = 20, R = 2}	{n = 40, R = 4}
0.30	7.5	[0, 0.3]	2.032	2.102	2.152	2.196	2.232	{n = 20, R = 4}	{n = 40, R = 4}
0.30	7.5	[0.7, 1]	2.066	2.159	2.202	2.253	2.296	{n = 20, R = 2}	{n = 40, R = 3}
0.30	10.0	[0, 0.3]	2.600	2.760	2.821	2.891	2.933	{n = 20, R = 4}	{n = 40, R = 3}
0.30	10.0	[0.7, 1]	2.796	2.926	3.033	3.081	3.128	{n = 20, R = 4}	{n = 40, R = 3}
**50%**									
0.00	0.0		1.018	1.024	1.030	1.037	1.055	{n = 40, R = 4}	{n = 20, R = 2}
0.00	0.0		1.018	1.024	1.029	1.036	1.056	{n = 40, R = 4}	{n = 20, R = 2}
0.05	5.0	[0, 0.05]	1.188	1.239	1.278	1.286	1.303	{n = 20, R = 4}	{n = 35, R = 2}
0.05	5.0	[0.95, 1]	1.191	1.245	1.277	1.299	1.318	{n = 20, R = 4}	{n = 40, R = 2}
0.05	7.5	[0, 0.05]	1.265	1.391	1.473	1.551	1.602	{n = 20, R = 4}	{n = 40, R = 2}
0.05	7.5	[0.95, 1]	1.284	1.427	1.513	1.610	1.657	{n = 20, R = 4}	{n = 40, R = 2}
0.05	10.0	[0, 0.05]	1.353	1.588	1.720	1.862	1.935	{n = 20, R = 4}	{n = 40, R = 3}
0.05	10.0	[0.95, 1]	1.407	1.664	1.895	2.047	2.131	{n = 20, R = 4}	{n = 40, R = 2}
0.15	5.0	[0, 0.15]	1.655	1.687	1.702	1.711	1.741	{n = 20, R = 4}	{n = 35, R = 2}
0.15	5.0	[0.85, 1]	1.666	1.699	1.712	1.734	1.760	{n = 20, R = 3}	{n = 40, R = 2}
0.15	7.5	[0, 0.15]	2.336	2.428	2.468	2.493	2.538	{n = 20, R = 4}	{n = 35, R = 2}
0.15	7.5	[0.85, 1]	2.409	2.515	2.547	2.563	2.617	{n = 20, R = 4}	{n = 40, R = 2}
0.15	10.0	[0, 0.15]	3.160	3.345	3.382	3.467	3.550	{n = 20, R = 4}	{n = 40, R = 2}
0.15	10.0	[0.85, 1]	3.447	3.577	3.635	3.717	3.766	{n = 20, R = 4}	{n = 40, R = 2}
0.30	5.0	[0, 0.3]	1.787	1.811	1.824	1.856	1.885	{n = 20, R = 4}	{n = 25, R = 2}
0.30	5.0	[0.7, 1]	1.778	1.816	1.826	1.861	1.874	{n = 20, R = 4}	{n = 40, R = 2}
0.30	7.5	[0, 0.3]	2.633	2.704	2.748	2.808	2.827	{n = 20, R = 4}	{n = 35, R = 2}
0.30	7.5	[0.7, 1]	2.695	2.763	2.790	2.843	2.879	{n = 20, R = 4}	{n = 35, R = 2}
0.30	10.0	[0, 0.3]	3.703	3.871	3.928	3.990	4.048	{n = 20, R = 4}	{n = 40, R = 2}
0.30	10.0	[0.7, 1]	3.852	4.011	4.059	4.095	4.187	{n = 20, R = 4}	{n = 40, R = 2}

**75%**									
0.00	0.0		1.078	1.101	1.116	1.159	1.230	{n = 40, R = 4}	{n = 20, R = 2}
0.00	0.0		1.077	1.100	1.116	1.158	1.234	{n = 40, R = 4}	{n = 20, R = 2}
0.05	5.0	[0, 0.05]	1.552	1.566	1.583	1.615	1.702	{n = 35, R = 4}	{n = 20, R = 2}
0.05	5.0	[0.95, 1]	1.557	1.574	1.585	1.622	1.704	{n = 40, R = 4}	{n = 20, R = 2}
0.05	7.5	[0, 0.05]	2.129	2.177	2.195	2.232	2.278	{n = 40, R = 4}	{n = 20, R = 2}
0.05	7.5	[0.95, 1]	2.174	2.232	2.258	2.302	2.342	{n = 40, R = 4}	{n = 25, R = 2}
0.05	10.0	[0, 0.05]	2.855	2.923	2.959	2.983	3.125	{n = 20, R = 4}	{n = 25, R = 2}
0.05	10.0	[0.95, 1]	3.043	3.123	3.179	3.198	3.260	{n = 40, R = 4}	{n = 25, R = 2}
0.15	5.0	[0, 0.15]	1.986	2.023	2.057	2.113	2.214	{n = 35, R = 4}	{n = 20, R = 2}
0.15	5.0	[0.85, 1]	1.997	2.039	2.064	2.134	2.241	{n = 40, R = 4}	{n = 20, R = 2}
0.15	7.5	[0, 0.15]	3.139	3.178	3.227	3.296	3.426	{n = 30, R = 4}	{n = 25, R = 2}
0.15	7.5	[0.85, 1]	3.147	3.226	3.254	3.361	3.574	{n = 40, R = 4}	{n = 20, R = 2}
0.15	10.0	[0, 0.15]	4.636	4.697	4.781	4.928	5.190	{n = 25, R = 4}	{n = 20, R = 2}
0.15	10.0	[0.85, 1]	4.798	4.838	4.935	5.081	5.347	{n = 40, R = 4}	{n = 20, R = 2}
0.30	5.0	[0, 0.3]	2.088	2.117	2.146	2.233	2.371	{n = 35, R = 4}	{n = 20, R = 2}
0.30	5.0	[0.7, 1]	2.091	2.121	2.157	2.236	2.355	{n = 40, R = 4}	{n = 20, R = 2}
0.30	7.5	[0, 0.3]	3.404	3.431	3.483	3.620	3.837	{n = 40, R = 4}	{n = 20, R = 2}
0.30	7.5	[0.7, 1]	3.408	3.452	3.507	3.636	3.818	{n = 35, R = 4}	{n = 20, R = 2}
0.30	10.0	[0, 0.3]	5.207	5.332	5.386	5.626	5.741	{n = 20, R = 4}	{n = 20, R = 2}
0.30	10.0	[0.7, 1]	5.296	5.337	5.420	5.644	5.847	{n = 35, R = 4}	{n = 20, R = 2}
**90%**									
0.00	0.0		1.137	1.177	1.208	1.290	1.429	{n = 40, R = 4}	{n = 20, R = 2}
0.00	0.0		1.138	1.176	1.207	1.292	1.432	{n = 40, R = 4}	{n = 20, R = 2}
0.05	5.0	[0, 0.05]	1.812	1.882	1.949	2.022	2.237	{n = 40, R = 4}	{n = 20, R = 2}
0.05	5.0	[0.95, 1]	1.823	1.872	1.959	2.029	2.242	{n = 40, R = 4}	{n = 20, R = 2}
0.05	7.5	[0, 0.05]	2.671	2.827	2.914	3.082	3.384	{n = 40, R = 4}	{n = 20, R = 2}
0.05	7.5	[0.95, 1]	2.713	2.860	2.963	3.138	3.468	{n = 40, R = 4}	{n = 20, R = 2}
0.05	10.0	[0, 0.05]	3.873	4.074	4.236	4.460	4.882	{n = 40, R = 4}	{n = 20, R = 2}
0.05	10.0	[0.95, 1]	3.945	4.232	4.357	4.621	5.157	{n = 40, R = 4}	{n = 20, R = 2}
0.15	5.0	[0, 0.15]	2.286	2.375	2.482	2.563	2.832	{n = 40, R = 4}	{n = 20, R = 2}
0.15	5.0	[0.85, 1]	2.289	2.380	2.465	2.568	2.868	{n = 40, R = 4}	{n = 20, R = 2}
0.15	7.5	[0, 0.15]	3.824	3.968	4.116	4.242	4.667	{n = 40, R = 4}	{n = 20, R = 2}
0.15	7.5	[0.85, 1]	3.814	3.994	4.164	4.303	4.790	{n = 40, R = 4}	{n = 20, R = 2}
0.15	10.0	[0, 0.15]	6.007	6.206	6.392	6.642	7.462	{n = 40, R = 4}	{n = 20, R = 2}
0.15	10.0	[0.85, 1]	6.012	6.268	6.465	6.755	7.481	{n = 40, R = 4}	{n = 20, R = 2}
0.30	5.0	[0, 0.3]	2.424	2.495	2.541	2.697	3.003	{n = 40, R = 4}	{n = 20, R = 2}
0.30	5.0	[0.7, 1]	2.414	2.500	2.565	2.692	2.960	{n = 40, R = 4}	{n = 20, R = 2}
0.30	7.5	[0, 0.3]	4.258	4.344	4.412	4.637	5.070	{n = 40, R = 4}	{n = 20, R = 2}
0.30	7.5	[0.7, 1]	4.240	4.346	4.440	4.622	5.197	{n = 40, R = 4}	{n = 20, R = 2}
0.30	10.0	[0, 0.3]	7.015	7.102	7.142	7.598	8.230	{n = 25, R = 4}	{n = 20, R = 2}
0.30	10.0	[0.7, 1]	6.970	7.156	7.229	7.522	8.245	{n = 40, R = 4}	{n = 20, R = 2}

**95%**									
0.00	0.0		1.176	1.229	1.268	1.379	1.571	{n = 40, R = 4}	{n = 20, R = 2}
0.00	0.0		1.177	1.226	1.269	1.382	1.576	{n = 40, R = 4}	{n = 20, R = 2}
0.05	5.0	[0, 0.05]	2.000	2.090	2.183	2.296	2.644	{n = 40, R = 4}	{n = 20, R = 2}
0.05	5.0	[0.95, 1]	1.998	2.084	2.205	2.316	2.650	{n = 40, R = 4}	{n = 20, R = 2}
0.05	7.5	[0, 0.05]	3.058	3.264	3.402	3.642	4.134	{n = 40, R = 4}	{n = 20, R = 2}
0.05	7.5	[0.95, 1]	3.084	3.299	3.418	3.682	4.249	{n = 40, R = 4}	{n = 20, R = 2}
0.05	10.0	[0, 0.05]	4.541	4.859	5.085	5.416	6.258	{n = 40, R = 4}	{n = 20, R = 2}
0.05	10.0	[0.95, 1]	4.633	4.986	5.234	5.538	6.545	{n = 40, R = 4}	{n = 20, R = 2}
0.15	5.0	[0, 0.15]	2.508	2.620	2.779	2.893	3.291	{n = 40, R = 4}	{n = 20, R = 2}
0.15	5.0	[0.85, 1]	2.499	2.632	2.783	2.900	3.334	{n = 40, R = 4}	{n = 20, R = 2}
0.15	7.5	[0, 0.15]	4.314	4.512	4.700	4.955	5.580	{n = 40, R = 4}	{n = 20, R = 2}
0.15	7.5	[0.85, 1]	4.318	4.518	4.795	4.975	5.699	{n = 40, R = 4}	{n = 20, R = 2}
0.15	10.0	[0, 0.15]	6.972	7.280	7.454	7.874	9.141	{n = 40, R = 4}	{n = 20, R = 2}
0.15	10.0	[0.85, 1]	6.877	7.331	7.588	7.966	9.194	{n = 40, R = 4}	{n = 20, R = 2}
0.30	5.0	[0, 0.3]	2.707	2.806	2.867	3.080	3.479	{n = 40, R = 4}	{n = 20, R = 2}
0.30	5.0	[0.7, 1]	2.726	2.806	2.902	3.066	3.412	{n = 40, R = 4}	{n = 20, R = 2}
0.30	7.5	[0, 0.3]	4.992	5.108	5.257	5.464	6.053	{n = 40, R = 4}	{n = 20, R = 2}
0.30	7.5	[0.7, 1]	4.973	5.075	5.239	5.492	6.276	{n = 40, R = 4}	{n = 20, R = 2}
0.30	10.0	[0, 0.3]	8.464	8.575	8.778	9.174	10.087	{n = 35, R = 4}	{n = 20, R = 2}
0.30	10.0	[0.7, 1]	8.362	8.591	8.749	9.202	10.260	{n = 40, R = 4}	{n = 20, R = 2}
**97.5%**									
0.00	0.0		1.213	1.275	1.326	1.464	1.715	{n = 40, R = 4}	{n = 20, R = 2}
0.00	0.0		1.213	1.274	1.325	1.466	1.715	{n = 40, R = 4}	{n = 20, R = 2}
0.05	5.0	[0, 0.05]	2.175	2.288	2.394	2.559	3.035	{n = 40, R = 4}	{n = 20, R = 2}
0.05	5.0	[0.95, 1]	2.169	2.273	2.418	2.565	2.996	{n = 40, R = 4}	{n = 20, R = 2}
0.05	7.5	[0, 0.05]	3.423	3.662	3.848	4.170	4.830	{n = 40, R = 4}	{n = 20, R = 2}
0.05	7.5	[0.95, 1]	3.423	3.698	3.894	4.206	5.022	{n = 40, R = 4}	{n = 20, R = 2}
0.05	10.0	[0, 0.05]	5.229	5.571	6.050	6.364	7.634	{n = 40, R = 4}	{n = 20, R = 2}
0.05	10.0	[0.95, 1]	5.220	5.694	6.025	6.413	7.678	{n = 40, R = 4}	{n = 20, R = 2}
0.15	5.0	[0, 0.15]	2.704	2.867	3.067	3.233	3.777	{n = 40, R = 4}	{n = 20, R = 2}
0.15	5.0	[0.85, 1]	2.696	2.889	3.095	3.232	3.784	{n = 40, R = 4}	{n = 20, R = 2}
0.15	7.5	[0, 0.15]	4.786	5.091	5.281	5.640	6.571	{n = 40, R = 4}	{n = 20, R = 2}
0.15	7.5	[0.85, 1]	4.809	5.035	5.398	5.662	6.669	{n = 40, R = 4}	{n = 20, R = 2}
0.15	10.0	[0, 0.15]	7.869	8.336	8.535	9.199	10.692	{n = 40, R = 4}	{n = 20, R = 2}
0.15	10.0	[0.85, 1]	7.730	8.348	8.770	9.225	10.856	{n = 40, R = 4}	{n = 20, R = 2}
0.30	5.0	[0, 0.3]	3.067	3.202	3.295	3.503	3.992	{n = 40, R = 4}	{n = 20, R = 2}
0.30	5.0	[0.7, 1]	3.111	3.189	3.258	3.457	3.929	{n = 40, R = 4}	{n = 20, R = 2}
0.30	7.5	[0, 0.3]	5.806	5.929	6.201	6.370	7.148	{n = 35, R = 4}	{n = 20, R = 2}
0.30	7.5	[0.7, 1]	5.811	5.904	6.227	6.384	7.444	{n = 35, R = 4}	{n = 20, R = 2}
0.30	10.0	[0, 0.3]	9.931	10.142	10.411	10.855	12.258	{n = 25, R = 4}	{n = 20, R = 2}
0.30	10.0	[0.7, 1]	9.858	10.082	10.528	10.847	12.745	{n = 35, R = 4}	{n = 20, R = 2}

**99%**									
0.00	0.0		1.257	1.332	1.388	1.574	1.905	{n = 40, R = 4}	{n = 20, R = 2}
0.00	0.0		1.259	1.334	1.394	1.577	1.897	{n = 40, R = 4}	{n = 20, R = 2}
0.05	5.0	[0, 0.05]	2.357	2.512	2.678	2.931	3.503	{n = 40, R = 4}	{n = 20, R = 2}
0.05	5.0	[0.95, 1]	2.403	2.527	2.713	2.916	3.412	{n = 40, R = 4}	{n = 20, R = 2}
0.05	7.5	[0, 0.05]	3.880	4.161	4.549	4.805	5.939	{n = 40, R = 4}	{n = 20, R = 2}
0.05	7.5	[0.95, 1]	3.847	4.189	4.538	4.924	5.816	{n = 40, R = 4}	{n = 20, R = 2}
0.05	10.0	[0, 0.05]	6.169	6.585	7.152	7.715	9.372	{n = 40, R = 4}	{n = 20, R = 2}
0.05	10.0	[0.95, 1]	6.027	6.656	7.104	7.628	9.486	{n = 40, R = 4}	{n = 20, R = 2}
0.15	5.0	[0, 0.15]	3.012	3.202	3.498	3.688	4.385	{n = 40, R = 4}	{n = 20, R = 2}
0.15	5.0	[0.85, 1]	3.002	3.207	3.496	3.676	4.385	{n = 40, R = 4}	{n = 20, R = 2}
0.15	7.5	[0, 0.15]	5.438	5.803	6.238	6.644	7.730	{n = 40, R = 4}	{n = 20, R = 2}
0.15	7.5	[0.85, 1]	5.486	5.819	6.135	6.601	7.888	{n = 40, R = 4}	{n = 20, R = 2}
0.15	10.0	[0, 0.15]	9.085	9.670	10.155	10.959	12.912	{n = 40, R = 4}	{n = 20, R = 2}
0.15	10.0	[0.85, 1]	8.924	9.773	10.254	10.856	12.875	{n = 40, R = 4}	{n = 20, R = 2}
0.30	5.0	[0, 0.3]	3.636	3.841	3.950	4.108	4.763	{n = 40, R = 4}	{n = 20, R = 2}
0.30	5.0	[0.7, 1]	3.718	3.800	3.930	4.136	4.815	{n = 30, R = 4}	{n = 20, R = 2}
0.30	7.5	[0, 0.3]	6.856	7.172	7.626	7.736	8.762	{n = 35, R = 4}	{n = 20, R = 2}
0.30	7.5	[0.7, 1]	6.897	7.266	7.517	7.798	9.180	{n = 40, R = 4}	{n = 20, R = 2}
0.30	10.0	[0, 0.3]	12.032	12.181	12.528	13.262	15.020	{n = 35, R = 4}	{n = 20, R = 2}
0.30	10.0	[0.7, 1]	11.872	12.306	12.769	13.448	15.576	{n = 40, R = 4}	{n = 20, R = 2}

Table 9 reveals that the mean and variance of $\hat{\zeta}$ are smallest when $u - l = 0$ and $m_{\max} = 0$ and largest when $u - l = 0.30$ and $m_{\max} = 10$. Furthermore, the skewness and kurtosis of $\hat{\zeta}$ are smallest when $u - l = 0$ and $m_{\max} = 0$ and largest when $u - l = 0.30$ and $m_{\max} = 5$.

For a fixed $m_{\max} > 0$, we deduce from Figure 7 that both the mean and variance of $\hat{\zeta}$ are positively correlated with $u - l$ up till some particular values of $u - l$, and are negatively correlated with $u - l$ larger than those values. Thus, if we include values $u - l > 0.3$, it is expected that relationship between the mean and variance of $\hat{\zeta}$ and $u - l$, given a fixed $m_{\max} > 0$, is according to an inverted U-curve. For a fixed $m_{\max} \geq 3$, the skewness and kurtosis of $\hat{\zeta}$ is negatively correlated with $u - l$ up till some particular values of $u - l$, and are positively correlated with $u - l$ larger than those values.

From the same figure, we observe that lower and upper systematic DINS produce very similar moment values: the circles, that refers to lower systematic DINS, overlaps very well with the squares, that symbolize upper systematic DINS. We delve deeper into the effect contributions of lower and upper systematic DINS in Tables 10 and 11. The results found in Table 10 reveal that only the mean and skewness of $\hat{\zeta}$ are consistent pointing out differences between the two forms of systematic DINS. Lower systematic produces smaller mean of $\hat{\zeta}$ on average, whereas upper systematic DINS produce larger skewness of $\hat{\zeta}$ on average. We do not observe these consistent results for the variance and kurtosis of $\hat{\zeta}$.

We deduce from Table 11, that the actual mean relative differences in the mean, variance, skewness and kurtosis of $\hat{\zeta}$ between lower and upper systematic DINS is minor. Indeed, the largest absolute mean relative difference is 2.246% (associated with minimum observed kurtosis of $\hat{\zeta}$), which is very small. Thus, we can

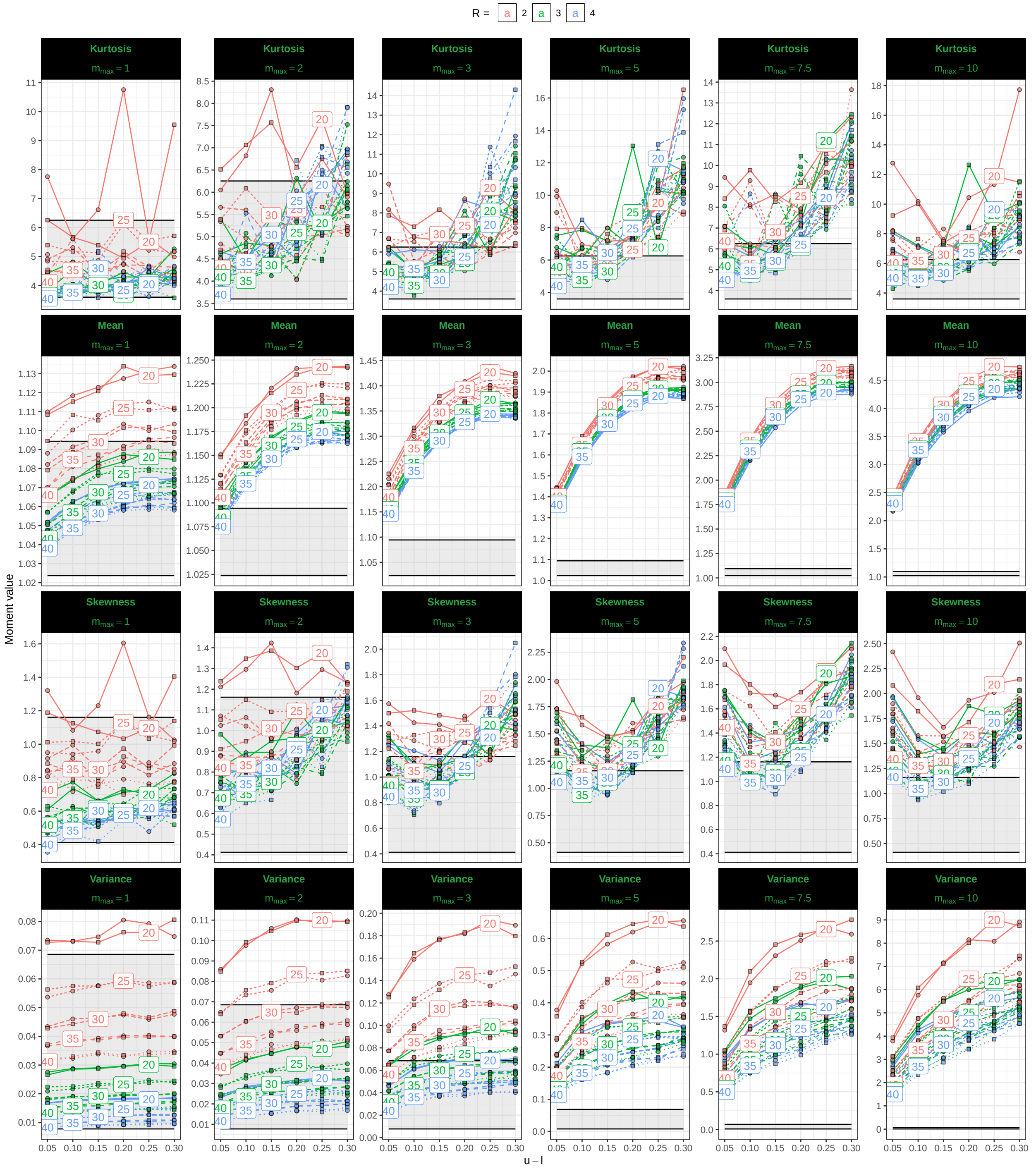

Figure 7. The mean, variance, skewness and kurtosis of the differences in non-selectivity (DINS) estimator, $\hat{\zeta}$, for all combinations of quantile intervals, q, maximum relocation magnitude multipliers, $m_{\max}$, and the 15 regarded study designs. Lines marked with circles represent systematic DINS in the lower range of the concentration interval, while lines marked with squares correspond to systematic DINS in the upper range of the concentration interval. The gray ribbons represent the ranges of moment values when there are no differences in non-selectivity (i.e., $u - l = 0$, $m_{\max} = 0$ or both). The numbers on top of the curves represent the number of clinical samples within each study design.

Table 13. Regarding the distribution summary statistics (minimum, first quartile, median, third quartile and maximum, respectively) of the 1%, 2.5%, 5%, 10%, 25%, 50%, 75%, 90%, 95%, 97.5%, and 99% percentiles of the differences in non-selectivity (DINS) estimator, $\hat{\zeta}$: what is the empirical probability of lower systematic DINS producing larger distribution summary statistic values than upper systematic DINS across all combinations quantile interval ranges, $u - l$, and maximum relocation magnitude multipliers, $m_{\max}$? Probabilities in this table are given in percent.

	Distribution summary statistics				
**Percentile**	**Min. %**	**First quartile %**	**Median %**	**Third quartile %**	**Max. %**
1%	32.432	43.243	43.243	29.730	40.541
2.5%	35.135	27.027	27.027	43.243	40.541
5%	35.135	21.622	24.324	40.541	43.243
10%	29.730	32.432	27.027	29.730	32.432
25%	35.135	27.027	32.432	24.324	13.514
50%	21.622	18.919	32.432	18.919	32.432
75%	24.324	18.919	16.216	27.027	32.432
90%	48.649	37.838	18.919	29.730	35.135
95%	40.541	45.946	27.027	32.432	29.730
97.5%	48.649	54.054	48.649	32.432	37.838
99%	59.459	37.838	62.162	40.541	40.541

conclude that the location of systematic DINS is practically irrelevant. Hence, the important part of q is its quantile range, $u - l$, and not the location of the interval.

In similarity to what we observed for random DINS, the variance is the most affected among the four considered moments. Thus, the percentiles of $\hat{\zeta}$ are expected to be affected due to this, in a manner where the smallest considered percentiles (1%, 2.5%, 5%, 10% and 25%) will decline, and the largest percentiles (50%, 75%, 90%, 95%, 97.5% and 99%) will increase as $u - l$ and $m_{\max}$ increase. However, this is not what we observe. Indeed, provided a fixed $m_{\max} > 0$, Table 12 illustrates that both the smallest and largest percentiles of $\hat{\zeta}$ increase with $u - l$ up till some particular points of $u - l$, and then starts decreasing with $u - l$ being larger than these points. This is similar to what we noted for the mean and variance of $\hat{\zeta}$ given a fixed $m_{\max} > 0$.

4.6 Simulation results: sixth set of simulations

In the sixth set of simulations, we examine the relationship between $\hat{\zeta}$ and M, across the same 15 study designs as before. Specifically, we will investigate how the first four moments of $\hat{\zeta}$, as well as selected percentiles of $\hat{\zeta}$ are influenced as M increases from 0 to 2 (i.e., 0% to 200%). Based on how $\hat{\zeta}$ is related to $\hat{\zeta}_0$ through M, we expect that the mean, variance, and the percentiles of $\hat{\zeta}$ will form a parabolic relationship with M.

```
## [[1]]
## NULL
##
## [[2]]
## NULL
##
## [[3]]
```

Table 14. Regarding the distribution summary statistics (minimum, first quartile, median, third quartile and maximum, respectively) of the 1%, 2.5%, 5%, 10%, 25%, 50%, 75%, 90%, 95%, 97.5%, and 99% percentiles of the differences in non-selectivity (DINS) estimator, $\hat{\zeta}$: what is the mean relative difference between the distribution summary statistic values produced by lower and upper systematic DINS, across all combinations of quantile interval ranges, $u - l$, and maximum relocation magnitude multipliers, $m_{\max}$? Mean relative differences in this table are given in percent.

	Distribution summary statistics				
**Percentile**	**Min. %**	**First quartile %**	**Median %**	**Third quartile %**	**Max. %**
1%	-0.275	-0.007	-0.130	-0.217	-0.090
2.5%	-0.056	-0.151	-0.363	-0.118	-0.311
5%	-0.495	-0.467	-0.643	-0.549	-0.779
10%	-0.676	-0.953	-1.313	-1.390	-1.554
25%	-1.256	-1.455	-1.685	-1.793	-1.889
50%	-1.591	-1.473	-1.486	-1.530	-1.466
75%	-0.805	-0.757	-0.961	-0.813	-0.997
90%	-0.169	-0.276	-0.541	-0.433	-0.799
95%	-0.104	-0.199	-0.453	-0.279	-0.850
97.5%	0.057	-0.062	-0.071	-0.244	-0.556
99%	0.239	-0.169	0.133	-0.385	-0.327

```
## NULL
##
## [[4]]
## NULL
##
## [[5]]
## NULL
##
## [[6]]
## NULL
##
## [[7]]
## NULL
##
## [[8]]
## NULL
##
## [[9]]
## NULL
##
## [[10]]
## NULL
##
## [[11]]
```

```
## NULL
##
## [[12]]
## NULL
##
## [[13]]
## NULL
##
## [[14]]
## NULL
##
## [[15]]
## NULL
```

Table 16. Distribution summary statistics of the 1%, 2.5%, 5%, 10%, 25%, 50%, 75%, 90%, 95%, 97.5%, and 99% percentiles of the differences in non-selectivity (DINS) estimator, $\hat{\zeta}$, for average relative increases in point-wise prediction interval widths due to DINS, $M(\%)$ = 0%, 25%, 50%, 75%, 100%, 125%, 150%, 175%, and 200%, across all 15 considered study designs. 'Min. PSD' and 'Max. PSD' are short for 'Minimize percentile study design' and 'Maximize percentile study design', respectively. Furthermore, 'Min.' and 'Max.' refers to Minimum and Maximum, respectively.

$M(\%)$	**Min.**	**First quartile**	**Median**	**Third quartile**	**Max.**	**Min. PSD**	**Max. PSD**
**1%**							
0	0.645	0.721	0.772	0.801	0.834	{n = 20, R = 2}	{n = 40, R = 4}
25	1.004	1.124	1.214	1.252	1.308	{n = 20, R = 2}	{n = 40, R = 4}
50	1.464	1.615	1.738	1.800	1.887	{n = 20, R = 2}	{n = 40, R = 4}
75	1.947	2.222	2.363	2.448	2.576	{n = 20, R = 2}	{n = 40, R = 4}
100	2.598	2.874	3.096	3.200	3.358	{n = 20, R = 2}	{n = 40, R = 4}
125	3.243	3.639	3.897	4.049	4.238	{n = 20, R = 2}	{n = 40, R = 4}
150	3.991	4.518	4.873	4.984	5.235	{n = 20, R = 2}	{n = 40, R = 4}
175	4.891	5.426	5.856	6.056	6.290	{n = 20, R = 2}	{n = 40, R = 4}
200	5.699	6.465	6.978	7.186	7.536	{n = 20, R = 2}	{n = 40, R = 4}
**2.5%**							
0	0.699	0.762	0.815	0.835	0.866	{n = 20, R = 2}	{n = 40, R = 4}
25	1.088	1.184	1.270	1.307	1.353	{n = 20, R = 2}	{n = 40, R = 4}
50	1.577	1.709	1.828	1.879	1.951	{n = 20, R = 2}	{n = 40, R = 4}
75	2.128	2.333	2.494	2.564	2.660	{n = 20, R = 2}	{n = 40, R = 4}
100	2.800	3.046	3.258	3.344	3.468	{n = 20, R = 2}	{n = 40, R = 4}
125	3.527	3.856	4.113	4.227	4.371	{n = 20, R = 2}	{n = 40, R = 4}
150	4.320	4.781	5.107	5.197	5.424	{n = 20, R = 2}	{n = 40, R = 4}
175	5.312	5.749	6.159	6.306	6.552	{n = 20, R = 2}	{n = 40, R = 4}
200	6.197	6.844	7.316	7.533	7.806	{n = 20, R = 2}	{n = 40, R = 4}
**5%**							
0	0.743	0.799	0.847	0.865	0.890	{n = 20, R = 2}	{n = 40, R = 4}

25	1.161	1.248	1.322	1.351	1.394	{n = 20, R = 2}	{n = 40, R = 4}
50	1.691	1.793	1.908	1.944	2.004	{n = 20, R = 2}	{n = 40, R = 4}
75	2.284	2.440	2.585	2.651	2.730	{n = 20, R = 2}	{n = 40, R = 4}
100	2.983	3.190	3.394	3.460	3.561	{n = 20, R = 2}	{n = 40, R = 4}
125	3.789	4.038	4.288	4.380	4.502	{n = 20, R = 2}	{n = 40, R = 4}
150	4.651	5.000	5.301	5.388	5.573	{n = 20, R = 2}	{n = 40, R = 4}
175	5.684	6.034	6.387	6.534	6.737	{n = 20, R = 2}	{n = 40, R = 4}
200	6.682	7.187	7.600	7.803	8.016	{n = 20, R = 2}	{n = 40, R = 4}
**10%**							
0	0.800	0.846	0.884	0.899	0.919	{n = 20, R = 2}	{n = 40, R = 4}
25	1.251	1.319	1.380	1.403	1.435	{n = 20, R = 2}	{n = 40, R = 4}
50	1.821	1.899	1.993	2.016	2.066	{n = 20, R = 2}	{n = 40, R = 4}
75	2.455	2.583	2.698	2.752	2.816	{n = 20, R = 2}	{n = 40, R = 4}
100	3.197	3.375	3.545	3.585	3.668	{n = 20, R = 2}	{n = 40, R = 4}
125	4.079	4.263	4.486	4.548	4.636	{n = 20, R = 2}	{n = 35, R = 4}
150	5.055	5.287	5.528	5.613	5.736	{n = 20, R = 2}	{n = 40, R = 4}
175	6.078	6.375	6.662	6.782	6.934	{n = 20, R = 2}	{n = 40, R = 4}
200	7.218	7.595	7.946	8.096	8.266	{n = 20, R = 2}	{n = 40, R = 4}
**25%**							
0	0.909	0.927	0.949	0.958	0.965	{n = 20, R = 2}	{n = 40, R = 4}
25	1.422	1.448	1.479	1.494	1.506	{n = 20, R = 2}	{n = 40, R = 4}
50	2.055	2.089	2.136	2.149	2.170	{n = 20, R = 2}	{n = 40, R = 4}
75	2.789	2.837	2.900	2.928	2.953	{n = 20, R = 2}	{n = 40, R = 4}
100	3.628	3.710	3.787	3.826	3.850	{n = 20, R = 2}	{n = 40, R = 4}
125	4.605	4.687	4.803	4.840	4.878	{n = 20, R = 2}	{n = 35, R = 4}
150	5.708	5.803	5.934	5.979	6.019	{n = 20, R = 2}	{n = 40, R = 4}
175	6.887	7.010	7.177	7.223	7.293	{n = 20, R = 2}	{n = 40, R = 4}
200	8.201	8.350	8.531	8.606	8.688	{n = 20, R = 2}	{n = 40, R = 4}
**50%**							
0	1.019	1.025	1.031	1.036	1.050	{n = 35, R = 4}	{n = 20, R = 2}
25	1.590	1.598	1.607	1.617	1.648	{n = 40, R = 4}	{n = 20, R = 2}
50	2.294	2.304	2.317	2.335	2.381	{n = 40, R = 4}	{n = 20, R = 2}
75	3.118	3.136	3.155	3.178	3.241	{n = 40, R = 4}	{n = 20, R = 2}
100	4.069	4.095	4.125	4.145	4.188	{n = 40, R = 4}	{n = 20, R = 2}
125	5.147	5.183	5.211	5.245	5.313	{n = 40, R = 4}	{n = 20, R = 2}
150	6.361	6.403	6.438	6.473	6.584	{n = 35, R = 4}	{n = 20, R = 2}
175	7.694	7.736	7.781	7.851	8.006	{n = 40, R = 4}	{n = 20, R = 2}
200	9.186	9.206	9.247	9.335	9.518	{n = 40, R = 4}	{n = 20, R = 2}
**75%**							
0	1.079	1.103	1.116	1.160	1.224	{n = 40, R = 4}	{n = 20, R = 2}
25	1.684	1.721	1.747	1.805	1.922	{n = 40, R = 4}	{n = 20, R = 2}
50	2.431	2.472	2.511	2.602	2.783	{n = 40, R = 4}	{n = 20, R = 2}

75	3.298	3.370	3.411	3.544	3.788	{n = 40, R = 4}	{n = 20, R = 2}
100	4.307	4.400	4.470	4.642	4.899	{n = 40, R = 4}	{n = 20, R = 2}
125	5.452	5.569	5.660	5.863	6.206	{n = 40, R = 4}	{n = 20, R = 2}
150	6.725	6.860	7.010	7.268	7.717	{n = 40, R = 4}	{n = 20, R = 2}
175	8.147	8.320	8.441	8.770	9.359	{n = 40, R = 4}	{n = 20, R = 2}
200	9.721	9.886	10.039	10.421	11.150	{n = 40, R = 4}	{n = 20, R = 2}
**90%**							
0	1.140	1.180	1.210	1.291	1.421	{n = 40, R = 4}	{n = 20, R = 2}
25	1.779	1.839	1.886	2.013	2.231	{n = 40, R = 4}	{n = 20, R = 2}
50	2.559	2.649	2.713	2.903	3.228	{n = 40, R = 4}	{n = 20, R = 2}
75	3.474	3.607	3.687	3.946	4.378	{n = 40, R = 4}	{n = 20, R = 2}
100	4.548	4.707	4.815	5.179	5.661	{n = 40, R = 4}	{n = 20, R = 2}
125	5.762	5.966	6.105	6.531	7.183	{n = 40, R = 4}	{n = 20, R = 2}
150	7.108	7.337	7.576	8.090	8.959	{n = 40, R = 4}	{n = 20, R = 2}
175	8.600	8.888	9.127	9.785	10.882	{n = 40, R = 4}	{n = 20, R = 2}
200	10.265	10.568	10.862	11.597	12.926	{n = 40, R = 4}	{n = 20, R = 2}
**95%**							
0	1.179	1.231	1.268	1.382	1.561	{n = 40, R = 4}	{n = 20, R = 2}
25	1.840	1.918	1.975	2.157	2.462	{n = 40, R = 4}	{n = 20, R = 2}
50	2.647	2.755	2.849	3.100	3.535	{n = 40, R = 4}	{n = 20, R = 2}
75	3.590	3.774	3.872	4.207	4.793	{n = 40, R = 4}	{n = 20, R = 2}
100	4.713	4.910	5.067	5.537	6.218	{n = 40, R = 4}	{n = 20, R = 2}
125	5.970	6.225	6.396	6.974	7.933	{n = 40, R = 4}	{n = 20, R = 2}
150	7.335	7.624	7.960	8.640	9.878	{n = 40, R = 4}	{n = 20, R = 2}
175	8.883	9.280	9.591	10.468	11.946	{n = 40, R = 4}	{n = 20, R = 2}
200	10.596	11.005	11.385	12.389	14.154	{n = 40, R = 4}	{n = 20, R = 2}
**97.5%**							
0	1.220	1.278	1.327	1.471	1.709	{n = 40, R = 4}	{n = 20, R = 2}
25	1.898	1.989	2.053	2.288	2.683	{n = 40, R = 4}	{n = 20, R = 2}
50	2.731	2.865	2.980	3.291	3.851	{n = 40, R = 4}	{n = 20, R = 2}
75	3.710	3.907	4.050	4.471	5.223	{n = 40, R = 4}	{n = 20, R = 2}
100	4.868	5.097	5.305	5.890	6.691	{n = 40, R = 4}	{n = 20, R = 2}
125	6.150	6.461	6.658	7.386	8.572	{n = 40, R = 4}	{n = 20, R = 2}
150	7.569	7.925	8.286	9.197	10.762	{n = 40, R = 4}	{n = 20, R = 2}
175	9.157	9.636	10.006	11.123	13.003	{n = 40, R = 4}	{n = 20, R = 2}
200	10.914	11.426	11.892	13.135	15.370	{n = 40, R = 4}	{n = 20, R = 2}
**99%**							
0	1.263	1.345	1.392	1.580	1.922	{n = 40, R = 4}	{n = 20, R = 2}
25	1.969	2.078	2.169	2.469	3.005	{n = 40, R = 4}	{n = 20, R = 2}
50	2.840	2.996	3.111	3.535	4.281	{n = 40, R = 4}	{n = 20, R = 2}
75	3.881	4.079	4.251	4.815	5.713	{n = 40, R = 4}	{n = 20, R = 2}
100	5.045	5.321	5.577	6.370	7.497	{n = 40, R = 4}	{n = 20, R = 2}

125	6.364	6.716	7.032	7.873	9.532	{n = 40, R = 4}	{n = 20, R = 2}
150	7.886	8.303	8.769	9.857	11.953	{n = 40, R = 4}	{n = 20, R = 2}
175	9.453	10.085	10.497	12.014	14.446	{n = 40, R = 4}	{n = 20, R = 2}
200	11.320	11.921	12.469	14.210	17.132	{n = 40, R = 4}	{n = 20, R = 2}

Table 15 reveals that the mean and variance of $\hat{\zeta}$ reaches their lowest values at $M = 0$ and their highest values at $M = 2$. Figure 8 and Table 15 demonstrate that the skewness and kurtosis of $\hat{\zeta}$ are both independent of M. In fact, the curves for skewness and kurtosis in Figure 8 are nearly horizontal across $M = 0$ to $M = 2$. All examined percentiles of $\hat{\zeta}$ increase with M, as evidenced by the curves in Figure 9 and the data in Table 16.

In summary, the mean and variance of $\hat{\zeta}$ exhibit a parabolic relationship with M. The percentiles of $\hat{\zeta}$ also exhibit a parabolic relationship with M. Although increasing the variance of $\hat{\zeta}$ tend to decrease its lower tail percentiles, they actually increase due to the increase of the mean of $\hat{\zeta}$. In consequence, both lower- and upper-tail percentiles increase as M increases. An important difference between the distribution of $\hat{\zeta}$ here compared to the distribution of $\hat{\zeta}$ observed for random and systematic DINS, is that the 1%, 2.5%, and 5% percentiles of $\hat{\zeta}$ are considerably more influenced. This is likely a product of random and systematic DINS producing a bimodal distribution for $\hat{\zeta}$, for small p and $u - l$ values.

4.7 Sensitivity analysis

The purpose of this sensitivity analysis is to examine the robustness of $\hat{\zeta}$ in the context of missing data and violating certain distributional assumptions in the model.

The model is based on the following equations:

$$
\begin{aligned}
\xi_i &= \beta_0 + \beta_1 \cdot \tau_i \\
x_{ir} &= \tau_i + h_{ir};\ \tau_i \sim \text{unif}(U_1, U_2),\ h_{ir} \sim \text{N}(0, \sigma^2_{\text{IVD-MD}_X}) \\
y_{ir} &= \xi_i + v_{ir};\ v_{ir} \sim \text{N}(0, \sigma^2_{\text{IVD-MD}_Y})
\end{aligned}
$$

This sensitivity study aims to investigate the impact of the following factors on $\hat{\zeta}$:

1. Missing Data Structures: How do Missing at Random (MAR) and Missing Not at Random (MNAR) data structures affect $\hat{\zeta}$?
2. Alternative Distributional Assumptions on τ_i: What is the impact of alternative distributional assumptions on τ_i on $\hat{\zeta}$?
3. Alternative Distributional Assumptions on h_{ir} and v_{ir}: How do alternative distributional assumptions on h_{ir} and v_{ir} affect $\hat{\zeta}$?

By exploring these questions, this simulation study aims to contribute to a deeper understanding of the robustness of $\hat{\zeta}$ in various important contexts.

4.7.1 Missing data structures

In the context of missing data, two structures are considered: Missing at Random (MAR) and Missing Not at Random (MNAR).

Table 15. Distribution summary statistics of the mean, variance, skewness and kurtosis of differences in non-selectivity estimator, $\hat{\zeta}$, for average relative increases in point-wise prediction interval widths due to DINS, $M(\%) = 0\%$, 25%, 50%, 75%, 100%, 125%, 150%, 175%, and 200%, across all 15 considered study designs. 'Min. MSD' and 'Max. MSD' are short for 'Minimizing moment study design' and 'Maximizing moment study design', respectively. Furthermore, 'Min.' and 'Max.' refers to Minimum and Maximum, respectively.

$M(\%)$	**Min.**	**First quartile**	**Median**	**Third quartile**	**Max.**	**Min. MSD**	**Max. MSD**
**Mean**							
0	1.025	1.034	1.040	1.056	1.089	{n = 40, R = 4}	{n = 20, R = 2}
25	1.600	1.613	1.623	1.647	1.709	{n = 40, R = 4}	{n = 20, R = 2}
50	2.306	2.322	2.339	2.374	2.468	{n = 40, R = 4}	{n = 20, R = 2}
75	3.135	3.162	3.182	3.233	3.352	{n = 40, R = 4}	{n = 20, R = 2}
100	4.093	4.127	4.160	4.231	4.346	{n = 40, R = 4}	{n = 20, R = 2}
125	5.179	5.224	5.265	5.336	5.517	{n = 40, R = 4}	{n = 20, R = 2}
150	6.396	6.443	6.508	6.606	6.847	{n = 40, R = 4}	{n = 20, R = 2}
175	7.737	7.802	7.861	7.995	8.305	{n = 40, R = 4}	{n = 20, R = 2}
200	9.229	9.284	9.338	9.508	9.860	{n = 40, R = 4}	{n = 20, R = 2}
**Variance**							
0	0.008	0.013	0.017	0.033	0.069	{n = 40, R = 4}	{n = 20, R = 2}
25	0.019	0.030	0.041	0.080	0.171	{n = 40, R = 4}	{n = 20, R = 2}
50	0.040	0.063	0.084	0.163	0.347	{n = 40, R = 4}	{n = 20, R = 2}
75	0.071	0.118	0.158	0.303	0.636	{n = 40, R = 4}	{n = 20, R = 2}
100	0.124	0.199	0.266	0.538	1.056	{n = 40, R = 4}	{n = 20, R = 2}
125	0.200	0.319	0.423	0.839	1.686	{n = 40, R = 4}	{n = 20, R = 2}
150	0.301	0.476	0.671	1.286	2.725	{n = 40, R = 4}	{n = 20, R = 2}
175	0.434	0.713	0.965	1.919	4.060	{n = 40, R = 4}	{n = 20, R = 2}
200	0.633	0.978	1.354	2.651	5.606	{n = 40, R = 4}	{n = 20, R = 2}
**Skewness**							
0	0.407	0.479	0.529	0.785	1.310	{n = 40, R = 4}	{n = 20, R = 2}
25	0.400	0.446	0.548	0.754	1.150	{n = 25, R = 4}	{n = 20, R = 2}
50	0.392	0.482	0.524	0.713	1.159	{n = 35, R = 4}	{n = 20, R = 2}
75	0.416	0.468	0.526	0.782	1.062	{n = 25, R = 4}	{n = 20, R = 2}
100	0.433	0.459	0.547	0.834	1.108	{n = 30, R = 4}	{n = 20, R = 2}
125	0.405	0.452	0.501	0.779	1.016	{n = 30, R = 4}	{n = 20, R = 2}
150	0.422	0.458	0.553	0.755	1.149	{n = 40, R = 3}	{n = 20, R = 2}
175	0.362	0.474	0.514	0.789	1.254	{n = 40, R = 4}	{n = 20, R = 2}
200	0.373	0.476	0.548	0.792	1.078	{n = 25, R = 4}	{n = 20, R = 2}
**Kurtosis**							
0	3.587	3.661	3.863	4.310	8.398	{n = 25, R = 3}	{n = 20, R = 2}
25	3.533	3.569	3.728	4.181	5.852	{n = 40, R = 3}	{n = 20, R = 2}
50	3.468	3.623	3.791	4.231	6.363	{n = 20, R = 4}	{n = 20, R = 2}
75	3.503	3.585	3.782	4.377	5.580	{n = 20, R = 4}	{n = 20, R = 2}
100	3.474	3.573	4.043	4.511	6.657	{n = 40, R = 3}	{n = 35, R = 2}
125	3.464	3.618	3.720	4.253	5.272	{n = 40, R = 4}	{n = 35, R = 2}
150	3.542	3.710	3.776	4.150	6.046	{n = 40, R = 3}	{n = 20, R = 2}
175	3.411	3.510	3.850	4.192	7.438	{n = 35, R = 4}	{n = 20, R = 2}
200	3.451	3.686	3.834	4.355	6.067	{n = 25, R = 4}	{n = 20, R = 2}

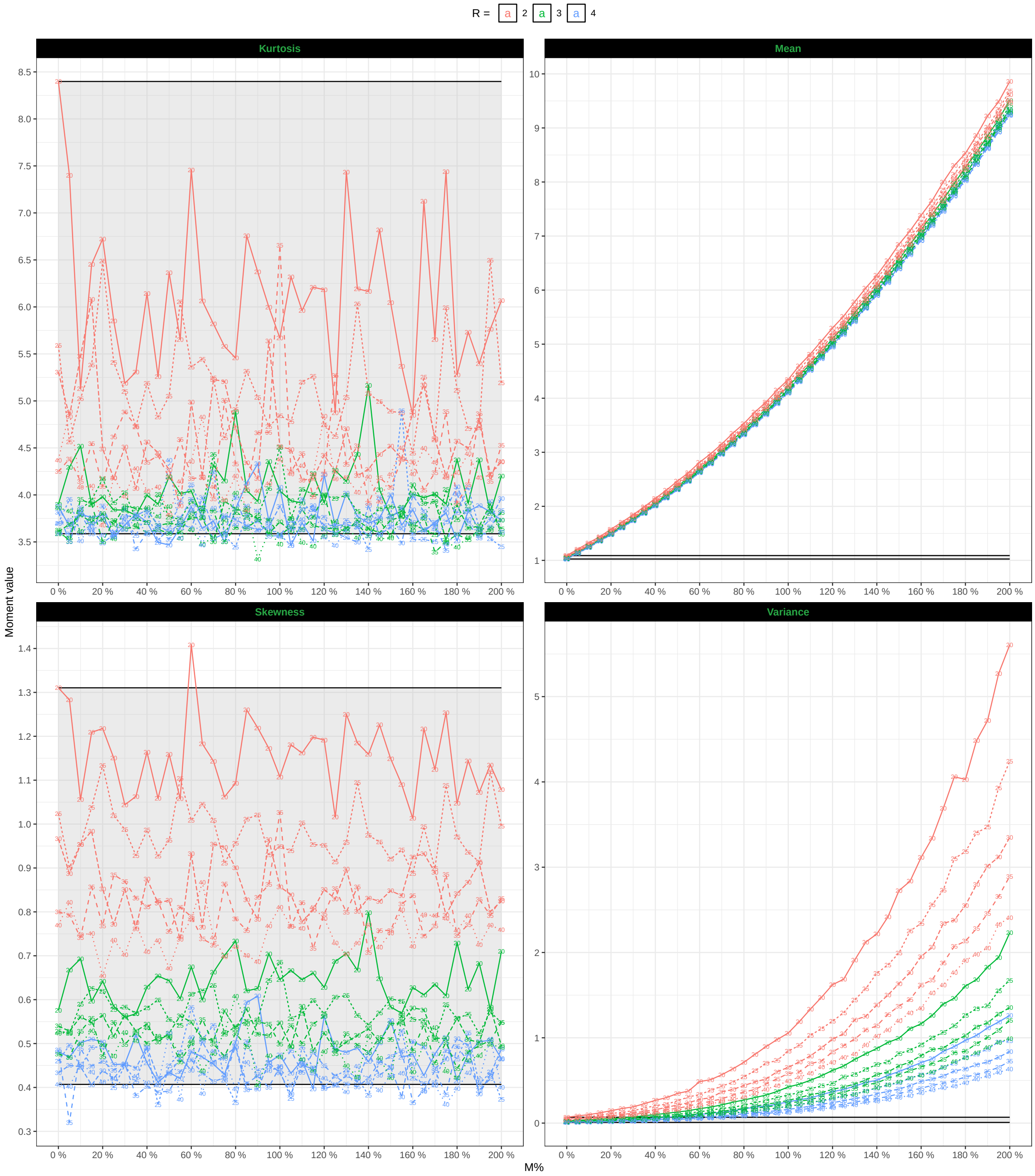

Figure 8. The mean, variance, skewness, and kurtosis of the differences in non-selectivity (DINS) estimator, $\hat{\zeta}$, for all considered average relative increases in point-wise prediction interval widths due to DINS, $M(\%)$, across the 15 regarded study designs. The gray ribbons represent the ranges of moment values when there are no DINS (i.e., $M(\%) = 0\%$). The numbers on top of the curves represent the number of clinical samples within each study design.

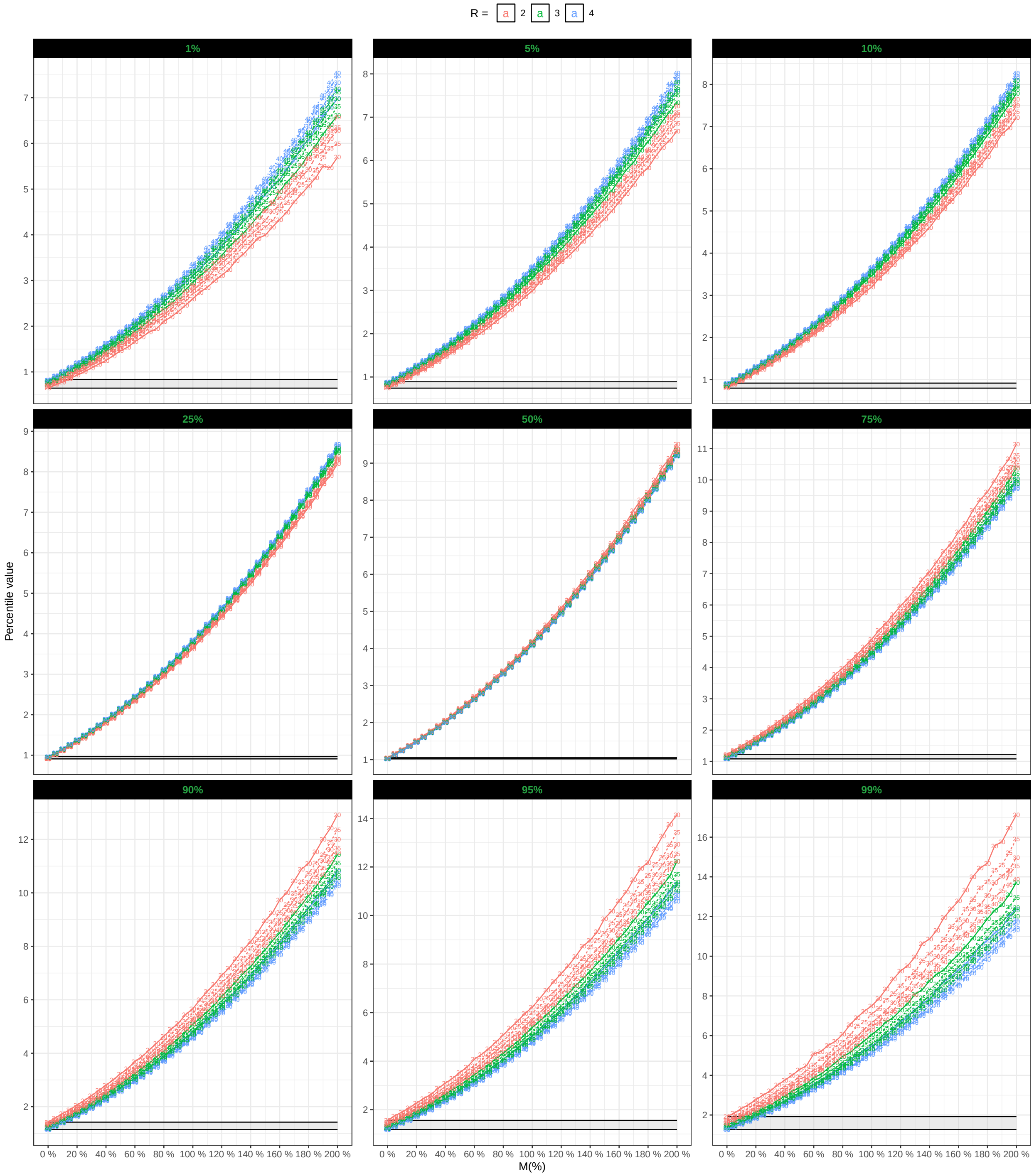

Figure 9. The 1%, 2.5%, 5%, 10%, 90%, 95%, 97.5% and 99% percentiles of the differences in non-selectivity (DINS) estimator, $\hat{\zeta}$, for all considered average relative increases in point-wise prediction interval widths due to DINS, $M(\%)$, across the 15 regarded study designs. The gray ribbons represent the ranges of percentiles when there are no DINS (i.e., $M(\%) = 0\%$). The numbers on top of the curves represent the number of clinical samples within each study design.

For MAR, a probability of a given measurement missing is considered. Since it is rare for more than 10% of measurements to be missing, two cases of MAR are examined:

1. Each observation has a 5% probability of being missing.
2. Each observation has a 10% probability of being missing.

For MNAR, measurements falling below a certain threshold are regarded as missing. We set this threshold to be U_1.

The simulations involve the following parameters: - Number of clinical samples (n): 25 and 40 - Number of replicates R: 3

In total we end up with 8 simulation parameter combinations.

```
## [[1]]
## NULL
##
## [[2]]
## NULL
##
## [[3]]
## NULL
##
## [[4]]
## NULL
##
## [[5]]
## NULL
##
## [[6]]
## NULL
##
## [[7]]
## NULL
##
## [[8]]
## NULL
```

Table 17. Some distribution measures of $\hat{\zeta}$ for data with missing measurements (MAR and MNAR), and corresponding distribution measures for complete data as reference.

Simulation parameters				Moments				Percentiles				
n	R	Structure	Probability	Mean	Variance	Skewness	Kurtosis	1%	25%	50%	75%	99%
25	3	mar	0.05	1.051	0.026	0.636	4.053	0.733	0.939	1.036	1.146	1.508
25	3	mar	0.10	1.058	0.033	0.666	4.217	0.705	0.930	1.043	1.166	1.570
40	3	mar	0.05	1.034	0.016	0.495	3.644	0.773	0.947	1.024	1.111	1.382
40	3	mar	0.10	1.038	0.020	0.507	3.637	0.751	0.941	1.027	1.123	1.437
25	3	mnar		1.059	0.028	1.388	11.082	0.743	0.949	1.040	1.147	1.560
40	3	mnar		1.043	0.017	0.796	6.121	0.785	0.959	1.032	1.115	1.419
25	3	none		1.044	0.021	0.529	3.587	0.757	0.945	1.031	1.133	1.436
40	3	none		1.033	0.013	0.524	3.863	0.804	0.956	1.025	1.101	1.344

Table 18 reveals that data missing according to a MAR model, increase the mean, variance, 50%, 75% and 99% percentiles of $\hat{\zeta}$, but decreases the 1% and 25% percentiles of $\hat{\zeta}$. Moreover, larger probability of missing data amplify these effects. The MNAR model signify similar effects on $\hat{\zeta}$, but in contrast to MAR, it also increase skewness and kurtosis of $\hat{\zeta}$ considerably. This increase in skewness and kurtosis is a consequence of how we define the MNAR model. In the MNAR model, measurements falling below U_1 are considered missing, so if $\mathrm{Var}[\tau_i]$ is small compared to $\sigma^2_{\text{IVD-MD}_X}$ and $\sigma^2_{\text{IVD-MD}_Y}$, it is quite likely that the effective study designs become so small that the estimator $\hat{\zeta}$ becomes unstable. Consequently, we can rule out that the increased skewness and kurtosis is a caused by the the MNAR model itself, but rather a consequence of $\sigma^2_{\text{IVD-MD}_X}$ and $\sigma^2_{\text{IVD-MD}_Y}$ being larger than $\mathrm{Var}[\tau_i]$ in a handful of the simulations, which we have discussed at an earlier point.

4.7.2 Alternative distributions of latent values

The default assumption is that τ_i follows a uniform distribution, $\mathrm{unif}(U_1, U_2)$. To examine the robustness of $\hat{\zeta}$, it is essential to investigate the impact of alternative distributional assumptions on τ_i.

1. Normal Distribution: $\tau_i \sim \mathrm{N}(\mu, \sigma^2)$, where τ_i has the same 0.1% and 99.9% percentiles as $\mathrm{Uniform}(U_1, U_2)$.
2. Location-Scale t-Distribution: $\tau_i \sim \mathrm{lst}(\nu, \mu, \sigma^2)$, where τ_i has the same 0.1% and 99.9% percentiles as $\mathrm{Uniform}(U_1, U_2)$, with $\nu = 5$ and $\nu = 15$.
3. Log-Normal Distribution: $\tau_i \sim \mathrm{lognormal}(\mu, \sigma^2)$, where τ_i has the same 0.1% and 99.9% percentiles as $\mathrm{Uniform}(U_1, U_2)$.

In these simulations, $n = 25; 40$ and $R = 3$. In total, this results in 10 simulation parameter combinations.

```
## [[1]]
## NULL
##
## [[2]]
## NULL
##
```

```
## [[3]]
## NULL
##
## [[4]]
## NULL
##
## [[5]]
## NULL
##
## [[6]]
## NULL
##
## [[7]]
## NULL
##
## [[8]]
## NULL
##
## [[9]]
## NULL
##
## [[10]]
## NULL
```

Table 18. Some distribution measures of $\hat{\zeta}$ for data with alternative distributional assumptions on τ_i, and corresponding distribution measures for $\tau_i \sim \text{Uniform}(U_1, U_2)$ as reference.

Simulation parameters				Moments				Percentiles				
n	R	Distribution	Degrees of freedom	Mean	Variance	Skewness	Kurtosis	1%	25%	50%	75%	99%
25	3	lnorm		1.048	0.021	0.612	4.053	0.753	0.947	1.034	1.135	1.450
40	3	lnorm		1.035	0.013	0.547	3.853	0.807	0.955	1.025	1.104	1.342
25	3	lst	5	1.049	0.021	0.601	3.798	0.759	0.949	1.034	1.136	1.453
25	3	lst	15	1.047	0.021	0.649	4.053	0.756	0.948	1.032	1.132	1.469
40	3	lst	5	1.037	0.013	0.505	3.656	0.808	0.959	1.028	1.106	1.340
40	3	lst	15	1.036	0.013	0.536	3.805	0.805	0.957	1.027	1.103	1.344
25	3	norm		1.049	0.021	0.611	4.053	0.755	0.947	1.035	1.135	1.449
40	3	norm		1.035	0.013	0.546	3.829	0.807	0.956	1.025	1.104	1.342
25	3	unif		1.044	0.021	0.529	3.587	0.757	0.945	1.031	1.133	1.436
40	3	unif		1.033	0.013	0.524	3.863	0.804	0.956	1.025	1.101	1.344

From Table 18, we observe that all considered alternative distributional assumptions on τ_i have minimal effect on $\hat{\zeta}$. There is a slight, albeit unimportant increase in the the four considered moments and the five considered percentiles. Thus, $\hat{\zeta}$ is indeed robust to the distribution of τ_i.

4.7.3 Alternative distribution of measurement error components

The default assumption is that $h_{ir} \sim \mathrm{N}(0, \sigma^2_{\text{IVD-MD}_X})$ and $v_{ir} \sim \mathrm{N}(0, \sigma^2_{\text{IVD-MD}_Y})$. It is of interest to check whether the distribution of $\hat{\zeta}$ changes drastically if

$$h_{ir} \sim \mathrm{lst}(\nu_h, 0, \sigma^2_{\text{IVD-MD}_X})$$
$$v_{ir} \sim \mathrm{lst}(\nu_v, 0, \sigma^2_{\text{IVD-MD}_Y})$$

for $\nu_h = 5; 15$, $\nu_v = 5; 15$, $n = 25; 40$ and $R = 3$. This results in 10 simulation parameter combinations.

```
## [[1]]
## NULL
##
## [[2]]
## NULL
##
## [[3]]
## NULL
##
## [[4]]
## NULL
##
## [[5]]
## NULL
##
## [[6]]
## NULL
##
## [[7]]
## NULL
##
## [[8]]
## NULL
##
## [[9]]
## NULL
##
## [[10]]
## NULL
```

Table 19. Some distribution measures of $\hat{\zeta}$ for data with alternative distributional assumptions on h_{ir}, v_{ir}, and corresponding distribution measures for h_{ir}, v_{ir} being normally distributed as reference.

Simulation parameters					Moments				Percentiles				
n	R	Distribution	ν_h	ν_v	Mean	Variance	Skewness	Kurtosis	1%	25%	50%	75%	99%
25	3	lst	5	5	1.043	0.020	0.581	4.112	0.752	0.947	1.030	1.127	1.425
25	3	lst	5	15	1.045	0.020	0.591	4.011	0.756	0.947	1.032	1.129	1.450
25	3	lst	15	5	1.046	0.020	0.565	3.783	0.761	0.948	1.033	1.129	1.442
25	3	lst	15	15	1.046	0.020	0.597	3.751	0.767	0.948	1.034	1.131	1.439
40	3	lst	5	5	1.034	0.013	0.494	3.760	0.798	0.958	1.024	1.102	1.340
40	3	lst	5	15	1.034	0.013	0.450	3.465	0.805	0.956	1.026	1.104	1.323
40	3	lst	15	5	1.033	0.013	0.500	3.582	0.804	0.955	1.024	1.102	1.335
40	3	lst	15	15	1.031	0.012	0.463	3.450	0.802	0.953	1.022	1.100	1.330
25	3	norm			1.044	0.021	0.529	3.587	0.757	0.945	1.031	1.133	1.436
40	3	norm			1.033	0.013	0.524	3.863	0.804	0.956	1.025	1.101	1.344

As is evident from Table 19, using the location-scale t-distribution instead of the normal distribution to model the measurement error terms have close to zero effect. Thus, $\hat{\zeta}$ is not sensitive to the distributional assumption of normality of the measurement error terms.

4.7.4 Sensitivity analysis conclusion

To summarize, $\hat{\zeta}$ is impacted by missing data structures such as the variations of MAR and MNAR structures considered here. However, the impact is a direct consequence of MAR and MNAR structures resulting in a smaller effective study design (smaller effective n and R), and mimics the effects on $\hat{\zeta}$ by decreasing the study design. No wild or unexpected behavior for $\hat{\zeta}$ is observed for these missing data structures.

Furthermore, $\hat{\zeta}$ is not sensitive to any of the alternative distributions we modeled τ_i, h_{ir} and v_{ir} with. The only thing to notice is that $\tau_i \sim \text{Uniform}(U_1, U_2)$ results in a slightly more conservative rejection region for what is considered acceptable magnitudes of DINS between a pair of IVD-MDs. For example, for $n = 25$, $R = 3$ and $M(\%) = 50\%$, the rejection region using the uniform distribution would be approximately $(3.24, \infty)$, but for the log-normal distribution it would be approximately $(3.26, \infty)$.
