# Supplementary figures and images for: Quantification of Difference in Nonselectivity Between In Vitro Diagnostic Medical Devices
Source: Biom J. 2025 Jan 2;67(1):e70032. doi: 10.1002/bimj.70032 (PMC11695778; doi:10.1002/bimj.70032)

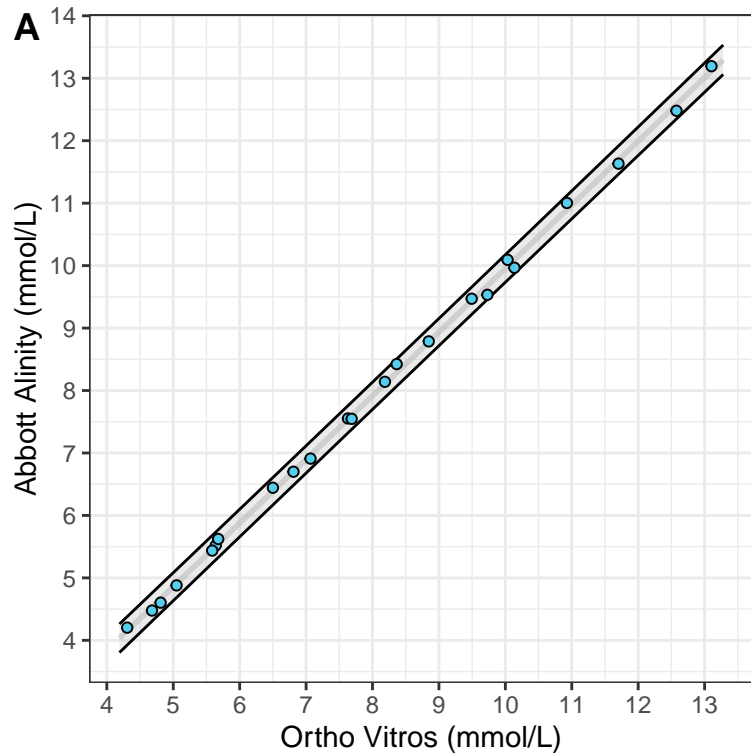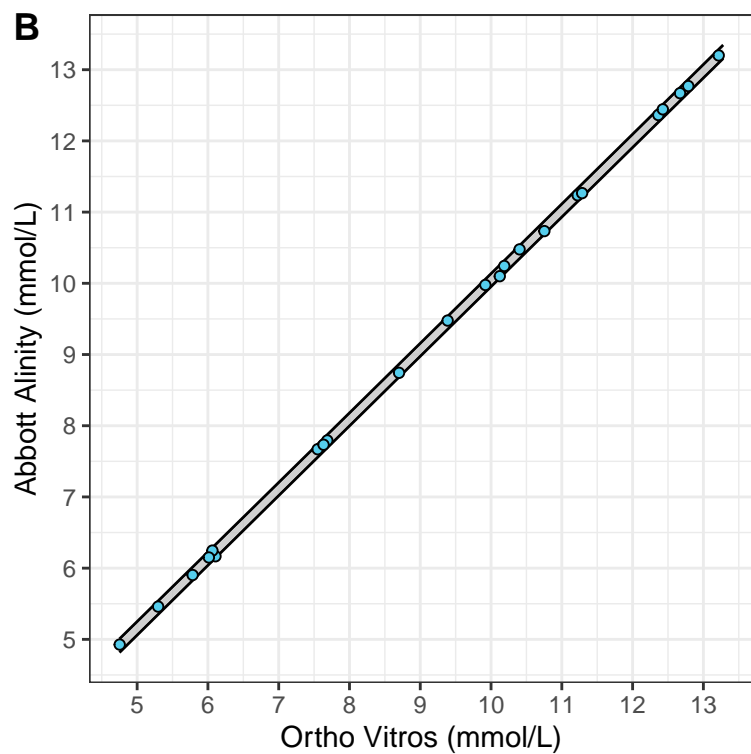

Supplement: Supplementary file 1 — Supporting Information [file BIMJ-67-e70032-s001.zip › Reproducibility resubmission v2/results pkf 22 10 2024 15 cores/Reproducing-manuscript-results_files/figure-latex/alinity-vs-vitros-1.pdf]

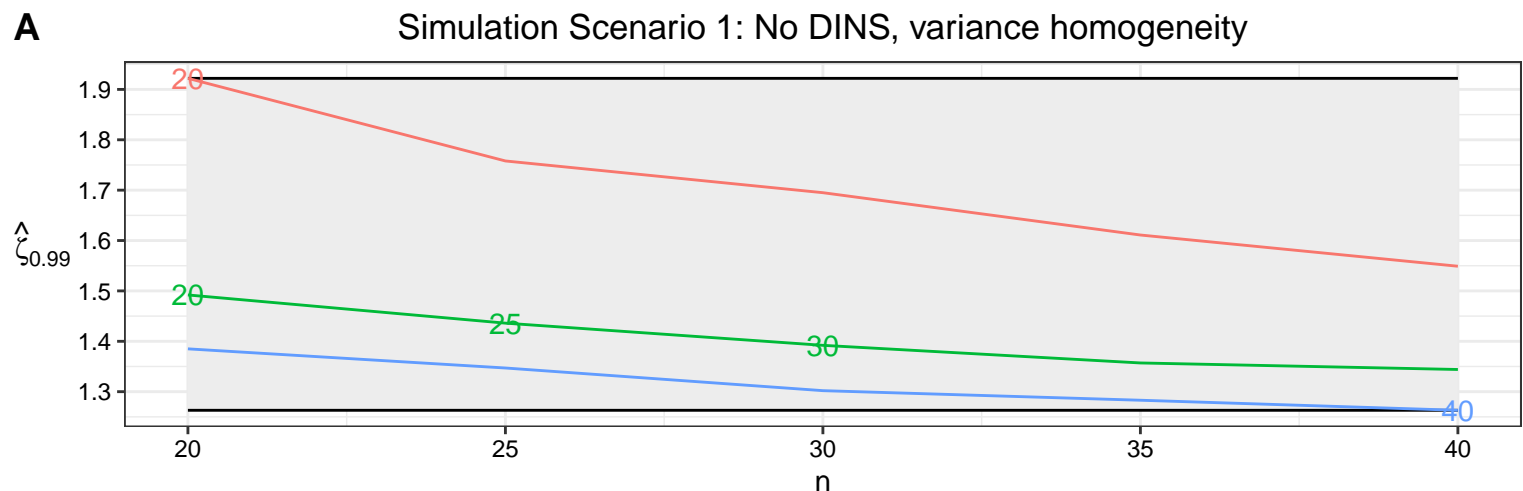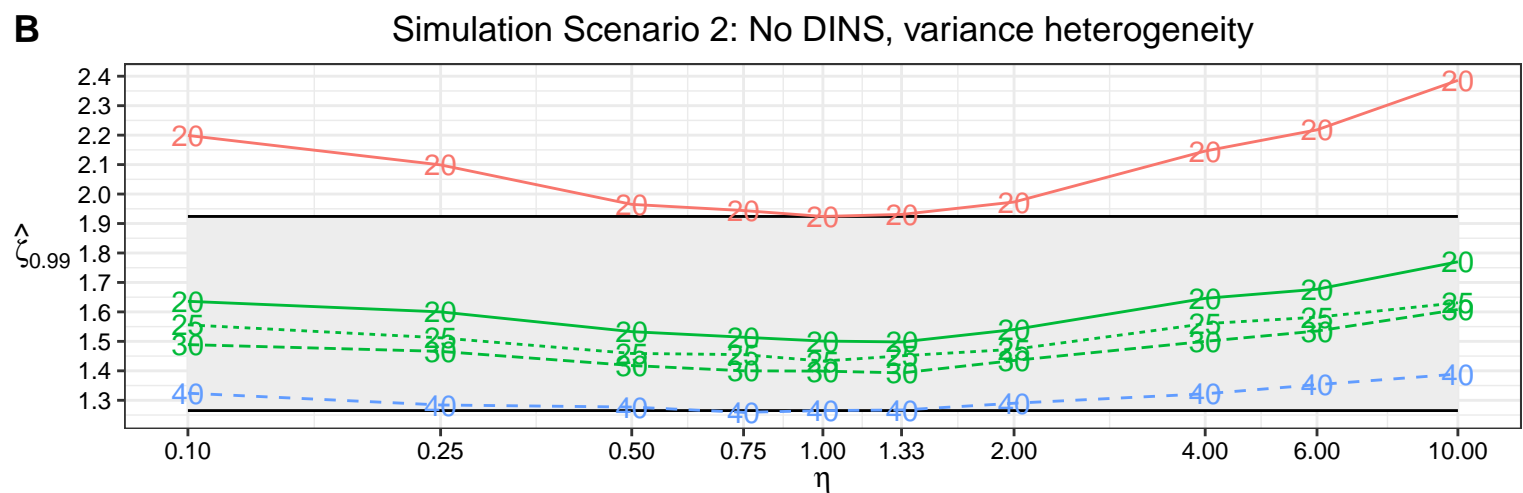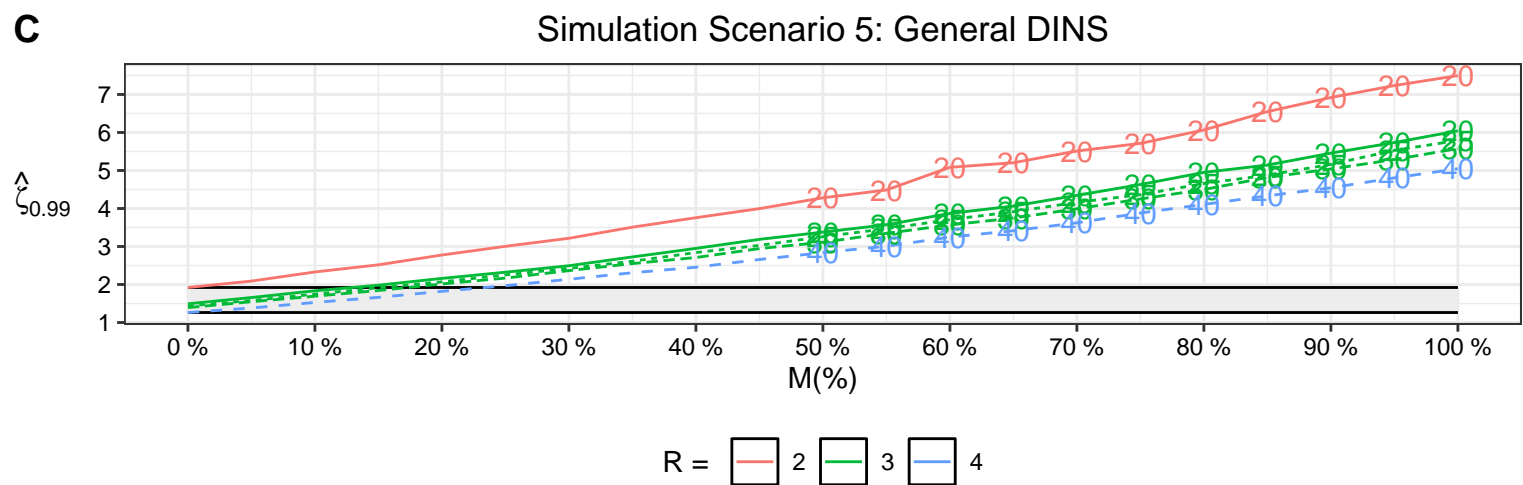

Supplement: Supplementary file 1 — Supporting Information [file BIMJ-67-e70032-s001.zip › Reproducibility resubmission v2/results pkf 22 10 2024 15 cores/Reproducing-manuscript-results_files/figure-latex/percentiles-of-zeta-1-2-5-1.pdf]

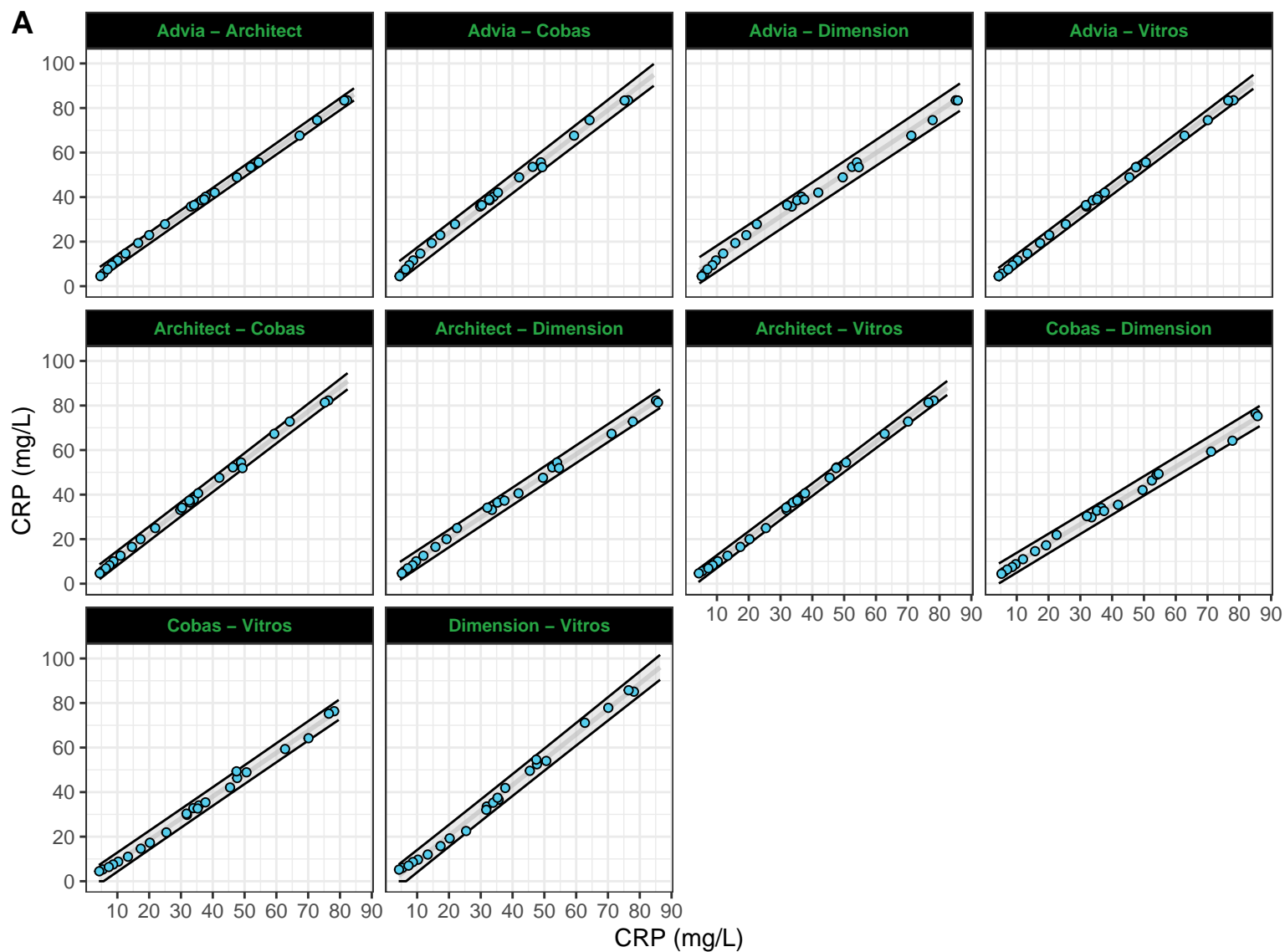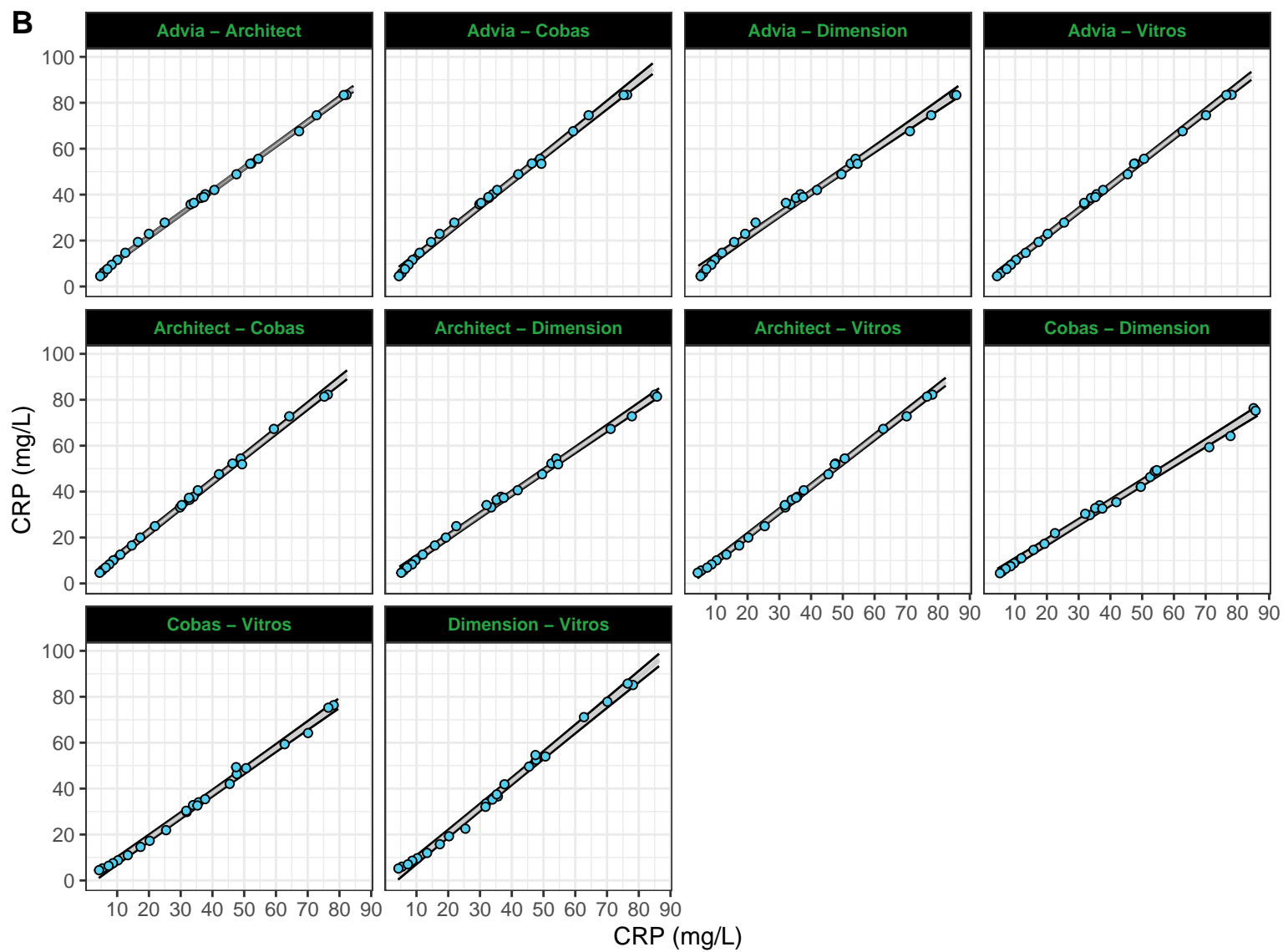

Supplement: Supplementary file 1 — Supporting Information [file BIMJ-67-e70032-s001.zip › Reproducibility resubmission v2/results pkf 22 10 2024 15 cores/Reproducing-manuscript-results_files/figure-latex/scatter-plots-for-crp-1.pdf]

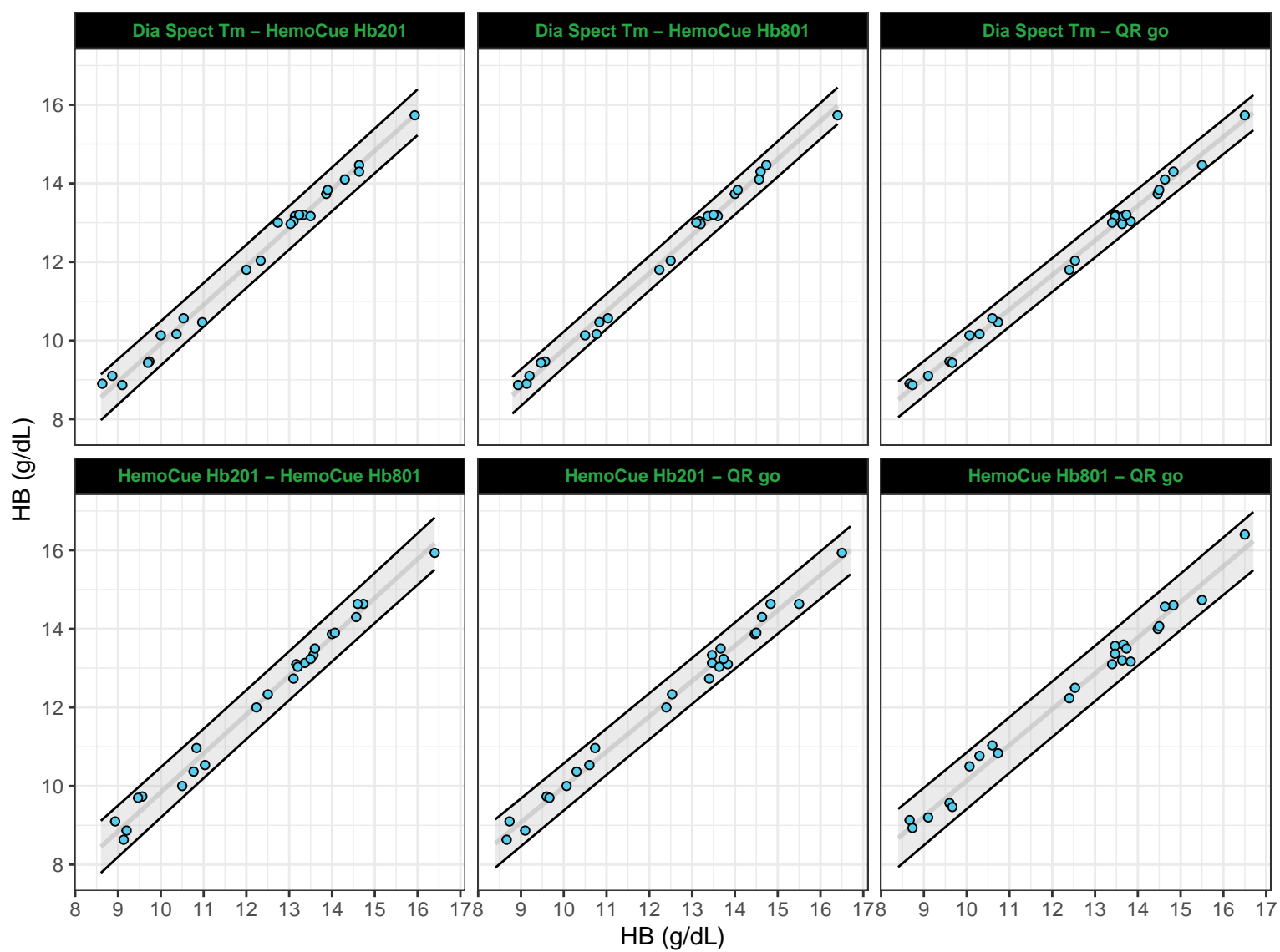

Supplement: Supplementary file 1 — Supporting Information [file BIMJ-67-e70032-s001.zip › Reproducibility resubmission v2/results pkf 22 10 2024 15 cores/Reproducing-manuscript-results_files/figure-latex/scatter-plots-for-hb-1.pdf]

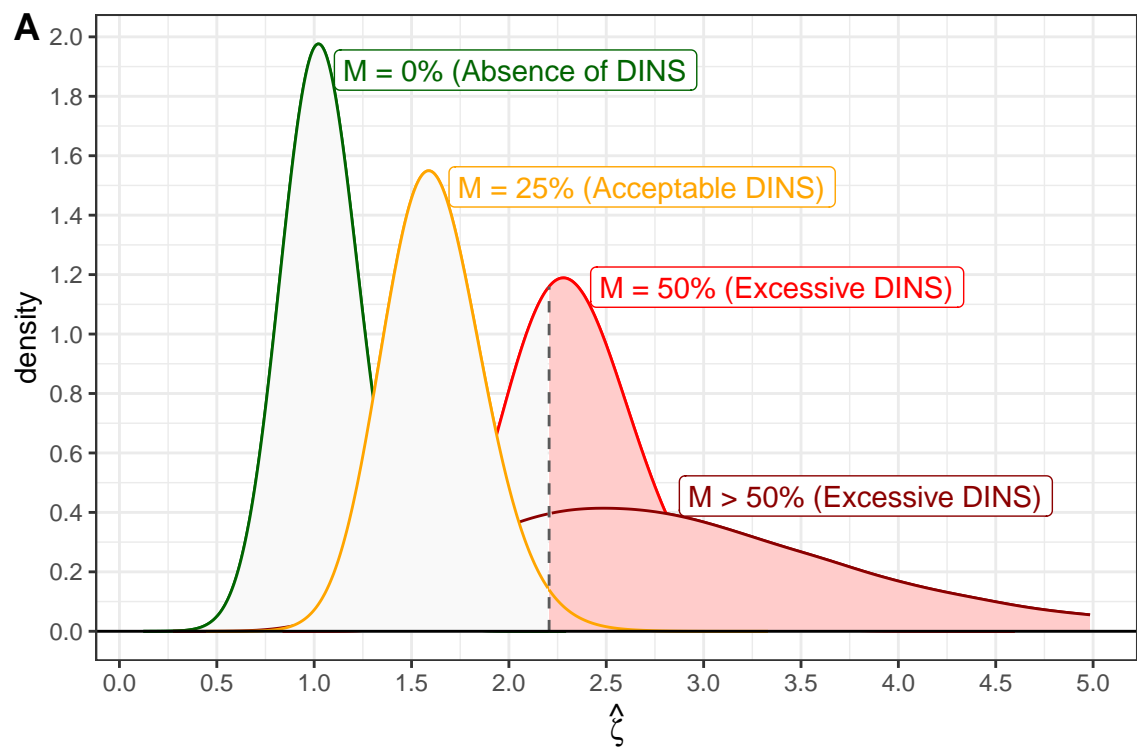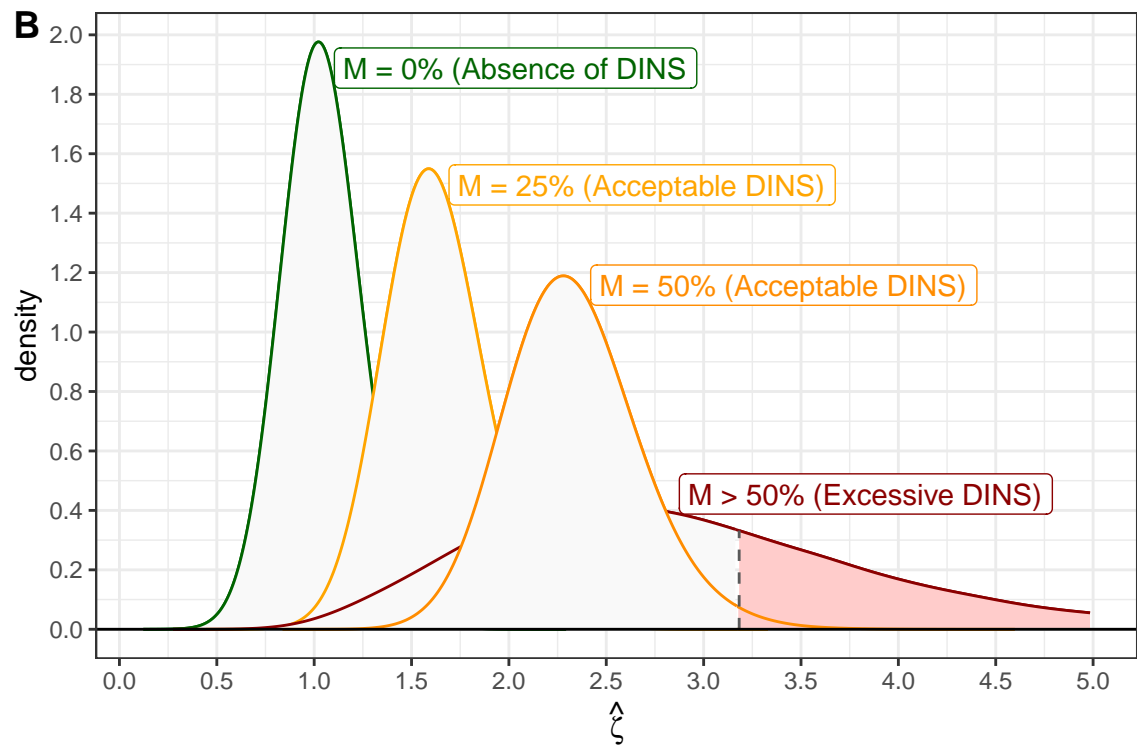

Supplement: Supplementary file 1 — Supporting Information [file BIMJ-67-e70032-s001.zip › Reproducibility resubmission v2/results pkf 22 10 2024 15 cores/Reproducing-manuscript-results_files/figure-latex/zeta-0-distribution-1.pdf]

R = a 2 a 3 a 4

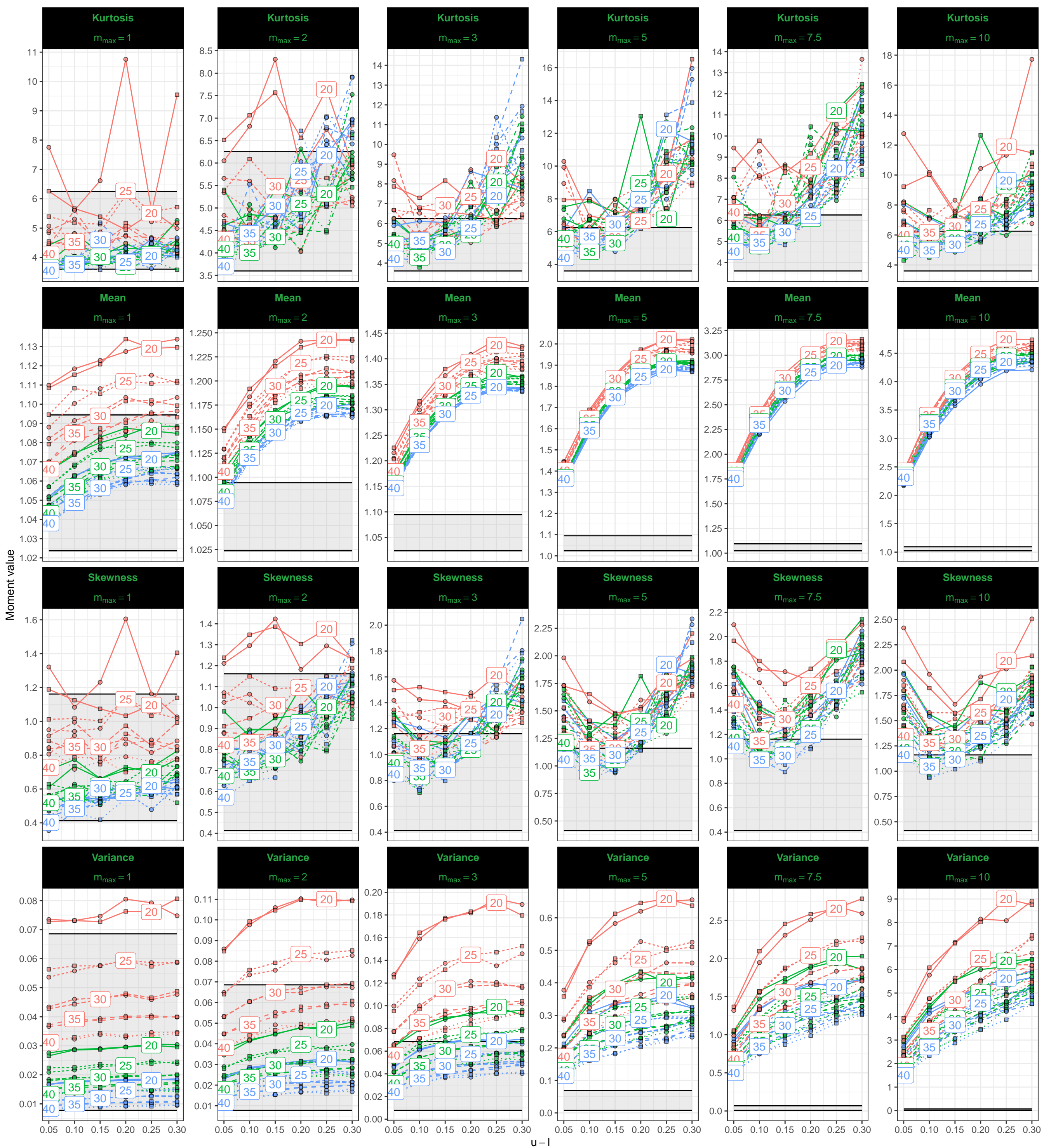

Supplement: Supplementary file 1 — Supporting Information [file BIMJ-67-e70032-s001.zip › Reproducibility resubmission v2/results pkf 22 10 2024 15 cores/Supplemental-file-repr_files/figure-latex/fifth-set-of-simulations-results-1.pdf]

**A**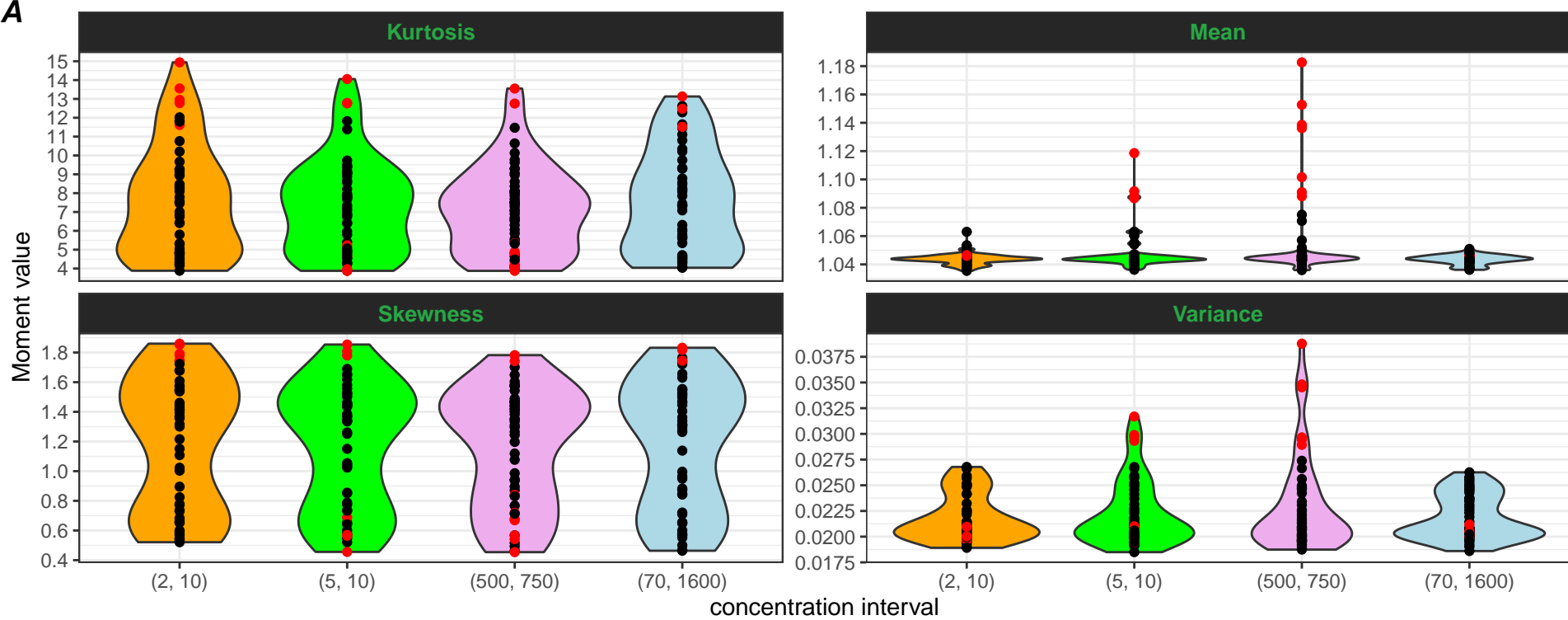**B**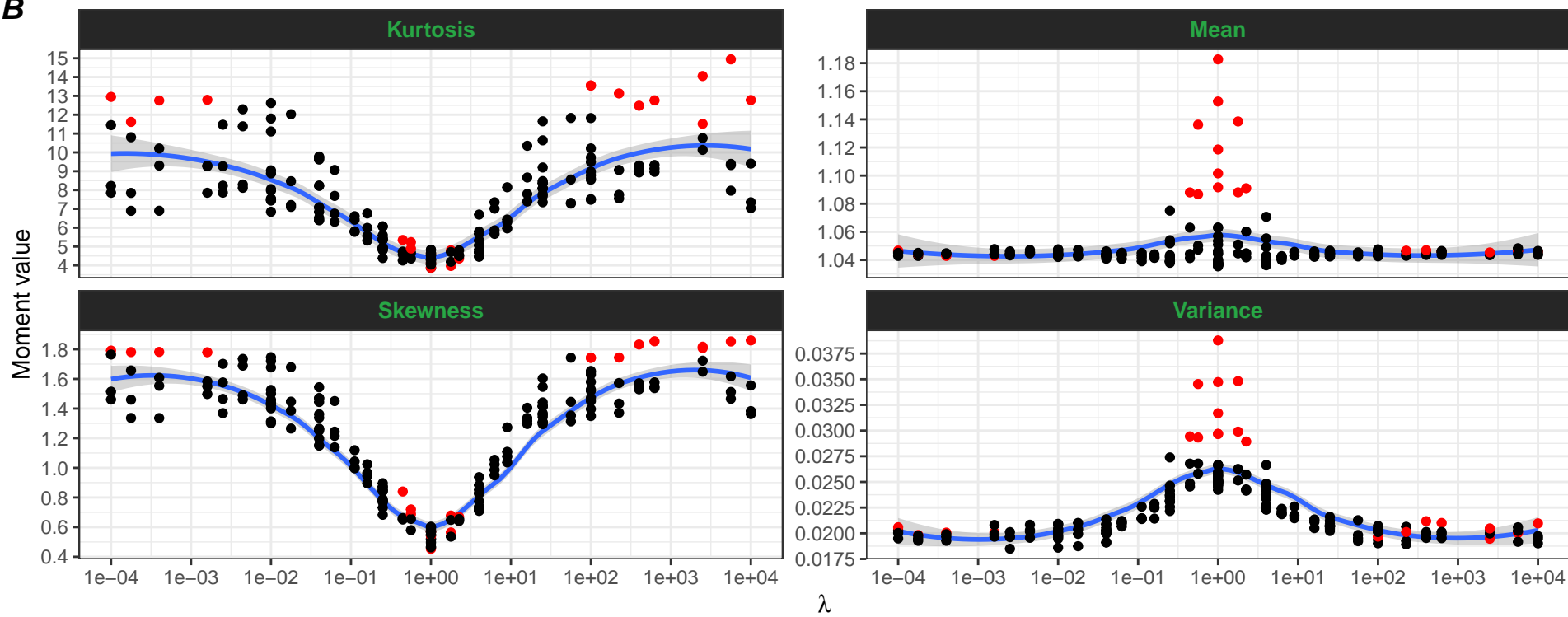

Supplement: Supplementary file 1 — Supporting Information [file BIMJ-67-e70032-s001.zip › Reproducibility resubmission v2/results pkf 22 10 2024 15 cores/Supplemental-file-repr_files/figure-latex/first-set-simulation-results-1.pdf]

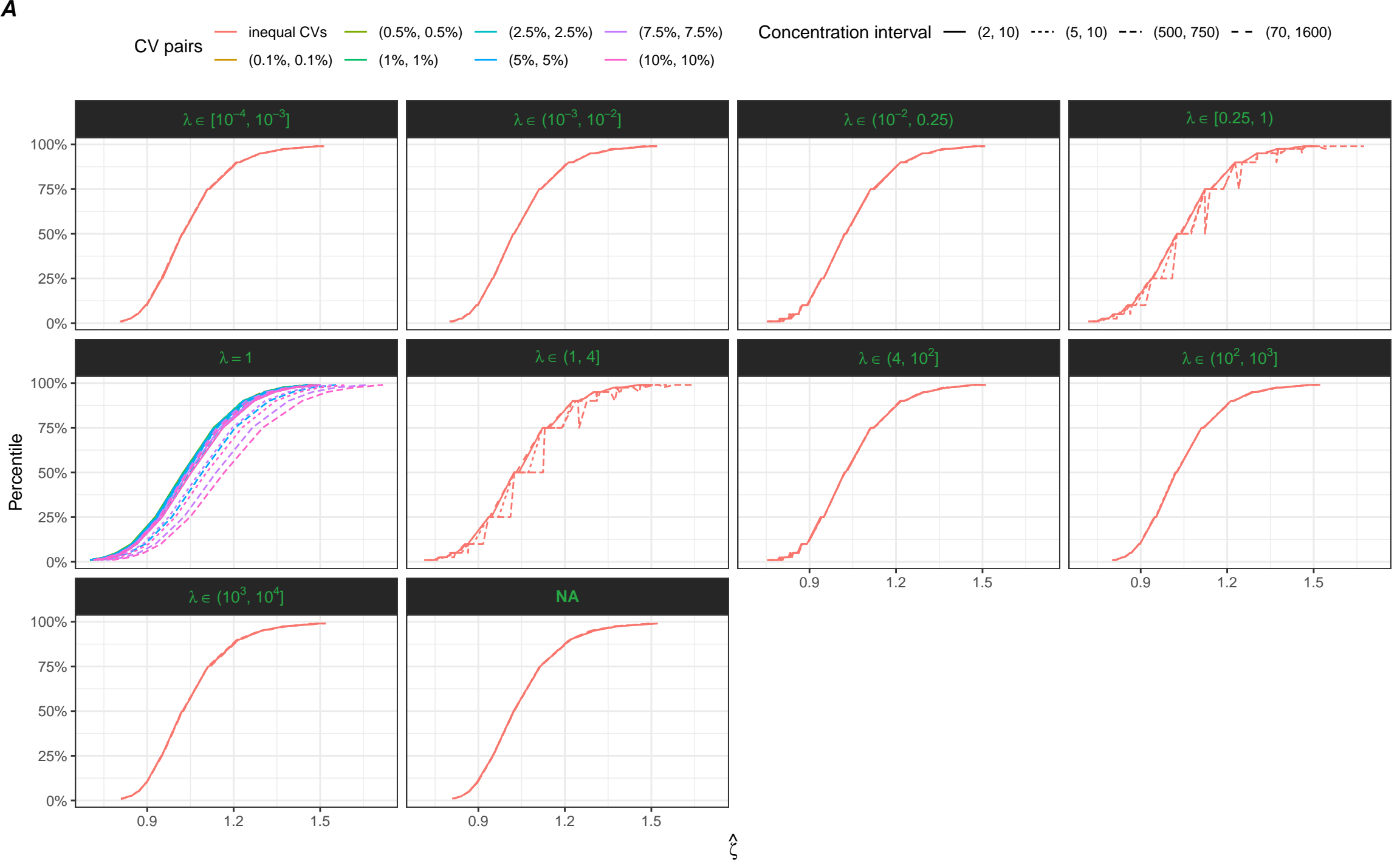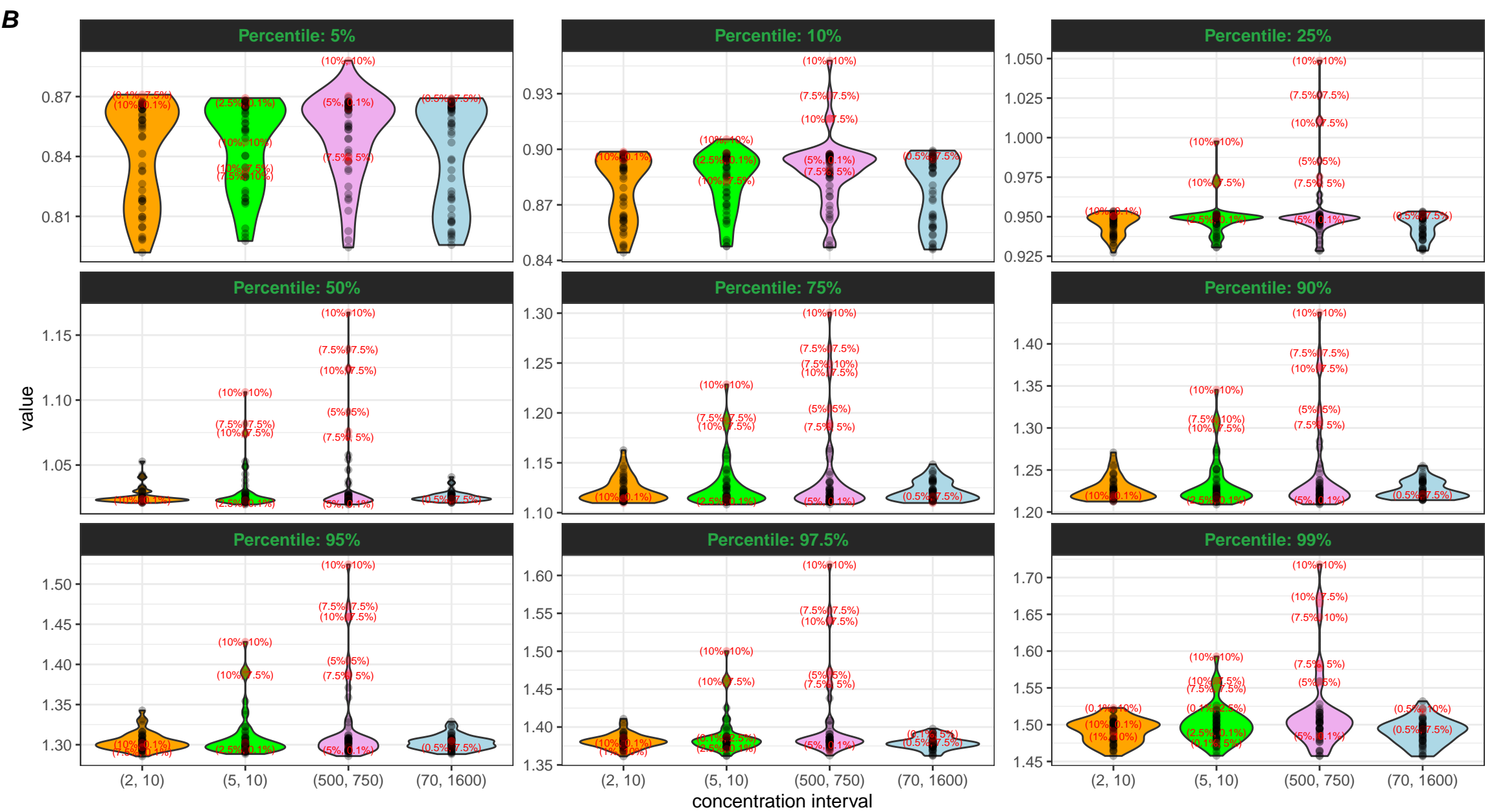

Supplement: Supplementary file 1 — Supporting Information [file BIMJ-67-e70032-s001.zip › Reproducibility resubmission v2/results pkf 22 10 2024 15 cores/Supplemental-file-repr_files/figure-latex/first-set-simulation-results-extra-1.pdf]

R = a 2 a 3 a 4

Moment value

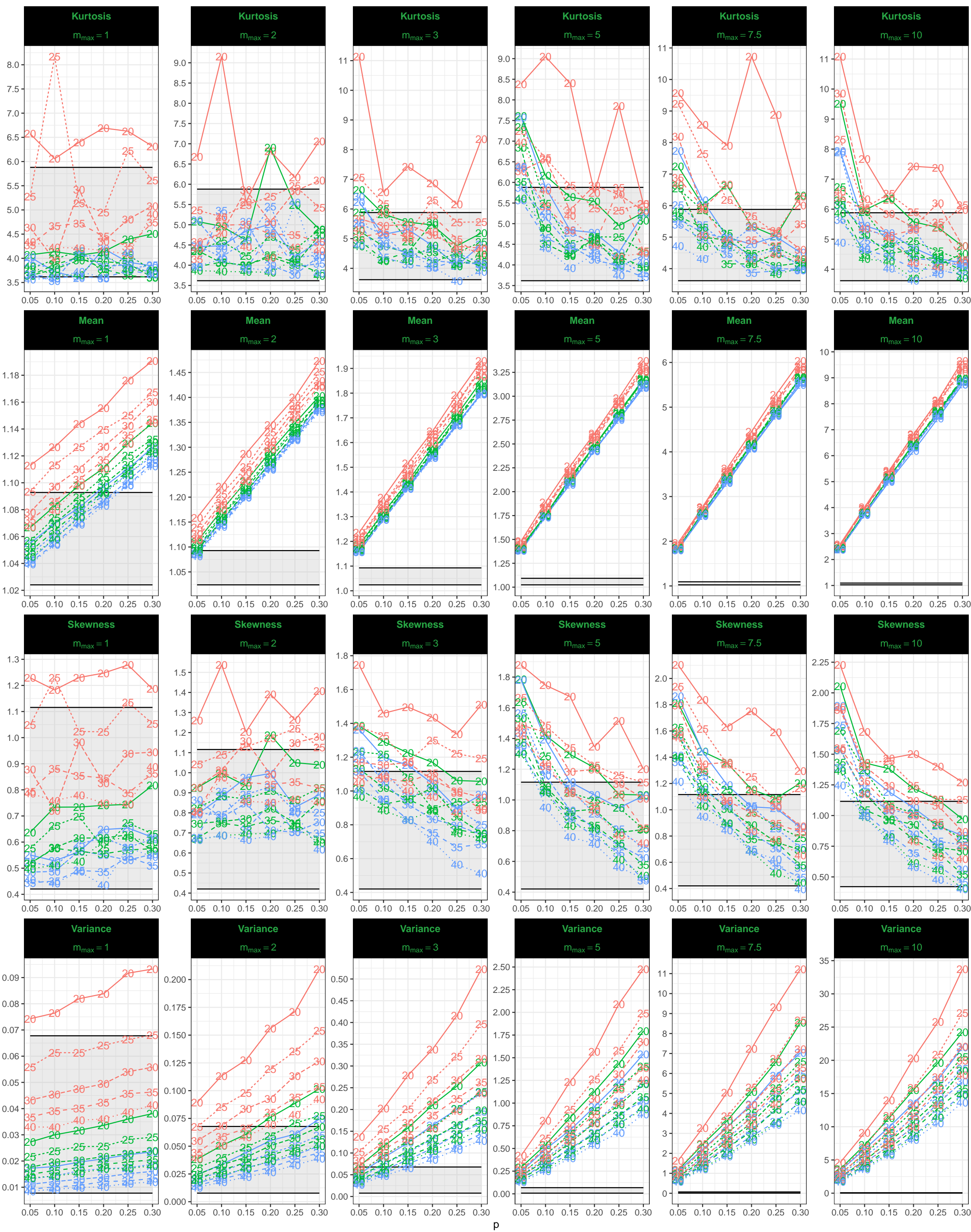

p

Supplement: Supplementary file 1 — Supporting Information [file BIMJ-67-e70032-s001.zip › Reproducibility resubmission v2/results pkf 22 10 2024 15 cores/Supplemental-file-repr_files/figure-latex/fourth-set-of-simulations-results-1.pdf]

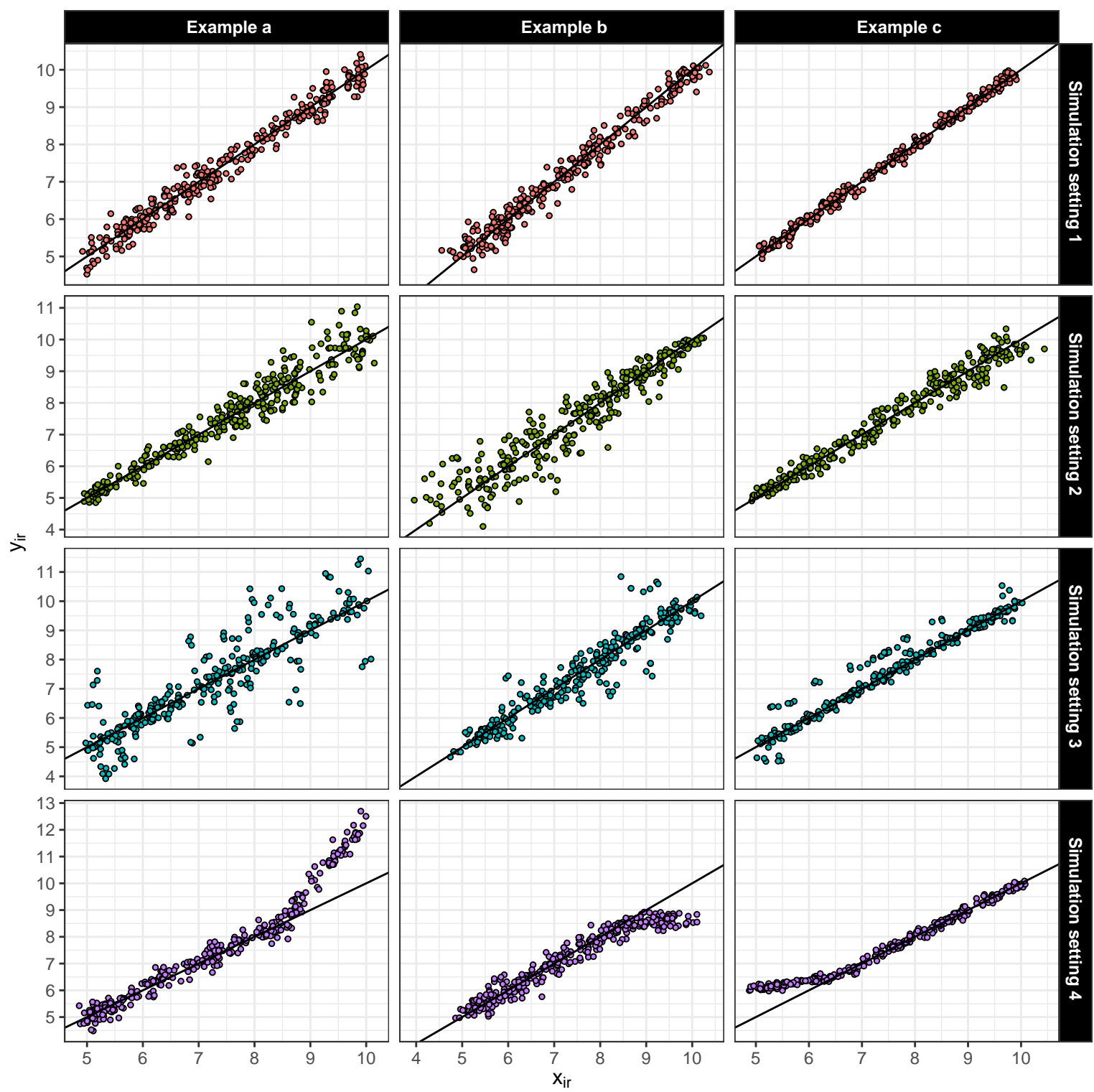

Supplement: Supplementary file 1 — Supporting Information [file BIMJ-67-e70032-s001.zip › Reproducibility resubmission v2/results pkf 22 10 2024 15 cores/Supplemental-file-repr_files/figure-latex/principle-figures-settings-1.pdf]

R = -a 2 -a 3 -a 4

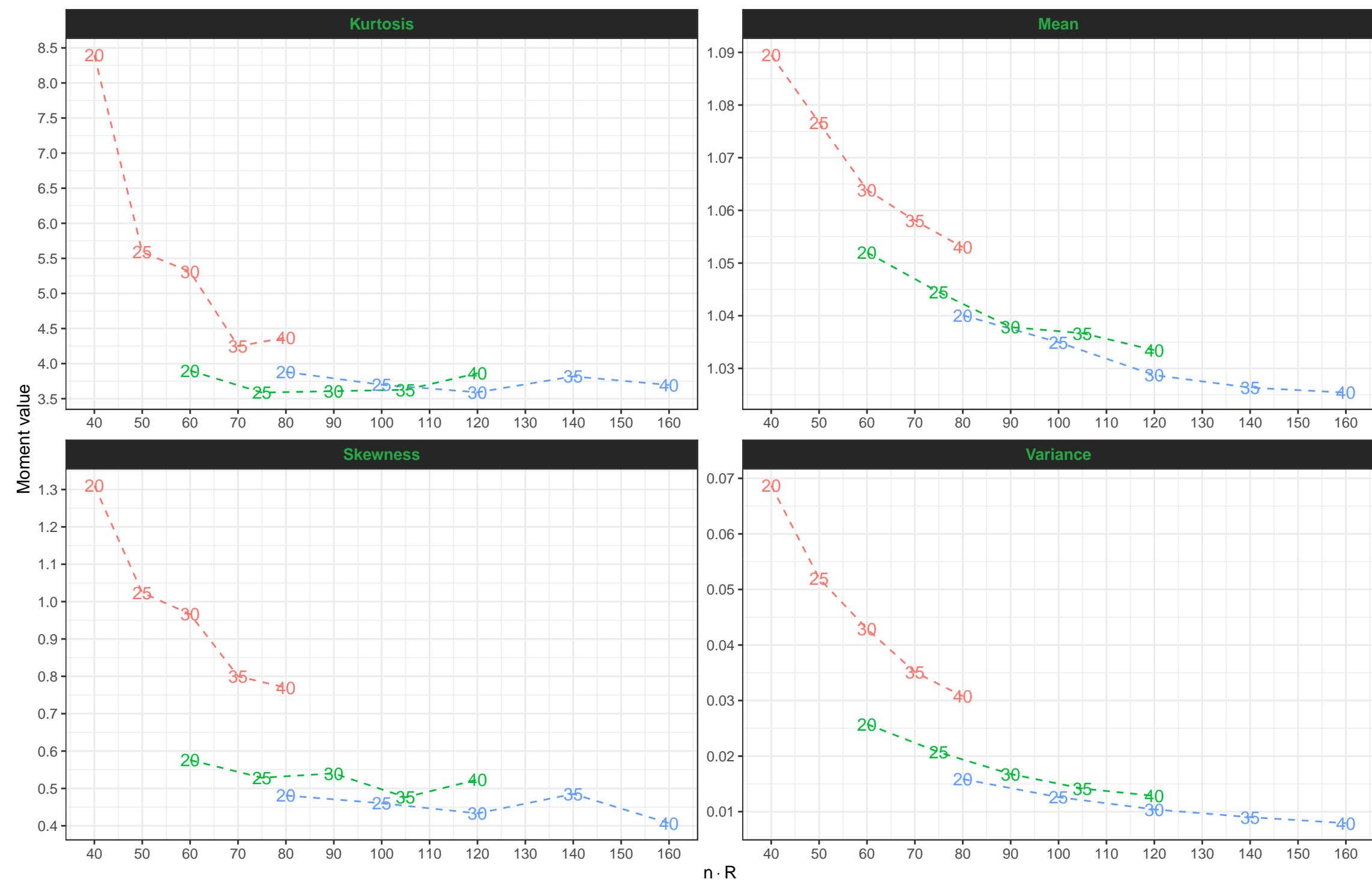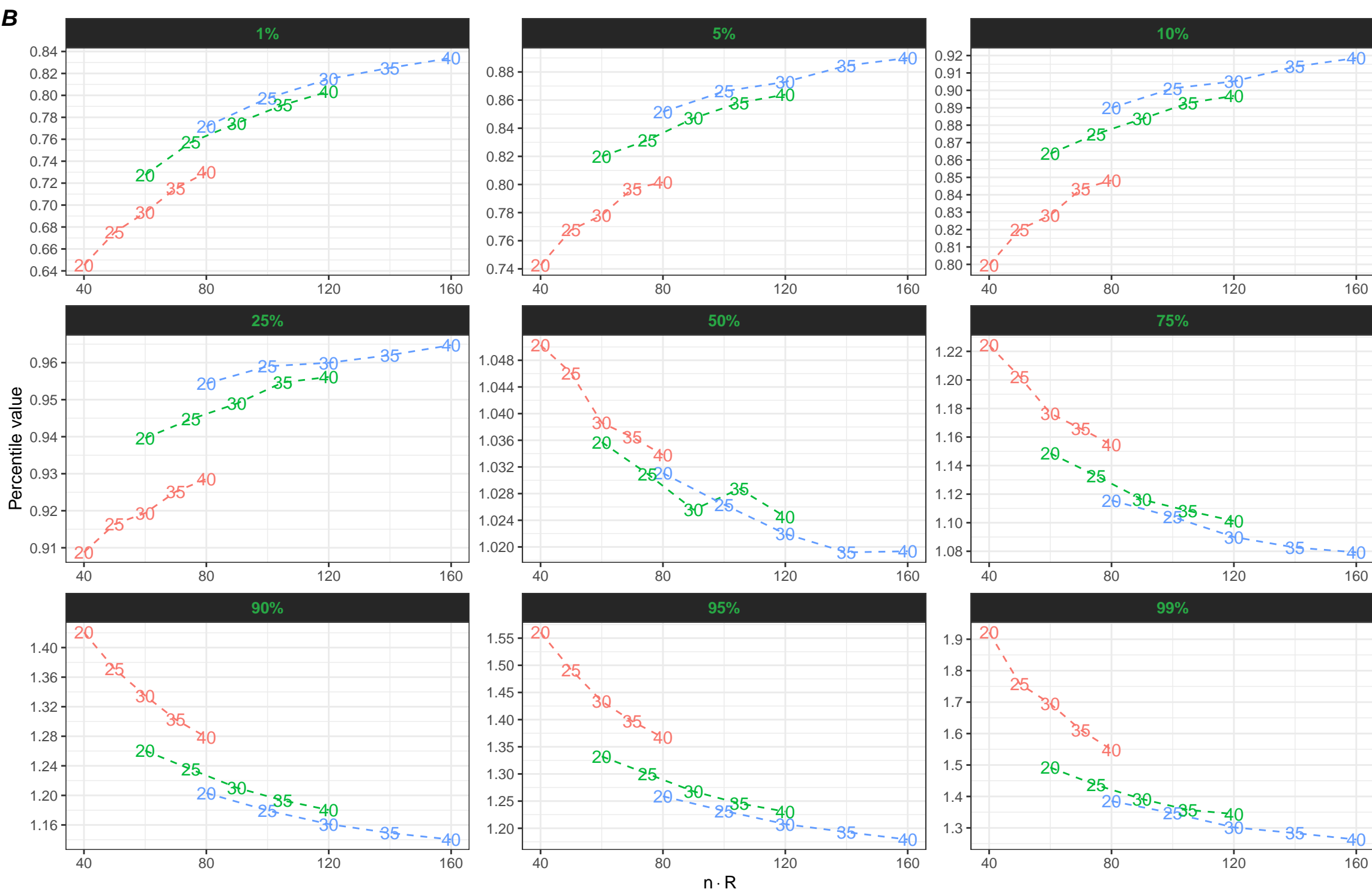

Supplement: Supplementary file 1 — Supporting Information [file BIMJ-67-e70032-s001.zip › Reproducibility resubmission v2/results pkf 22 10 2024 15 cores/Supplemental-file-repr_files/figure-latex/second-set-simulation-results-1.pdf]

R = a 2 a 3 a 4

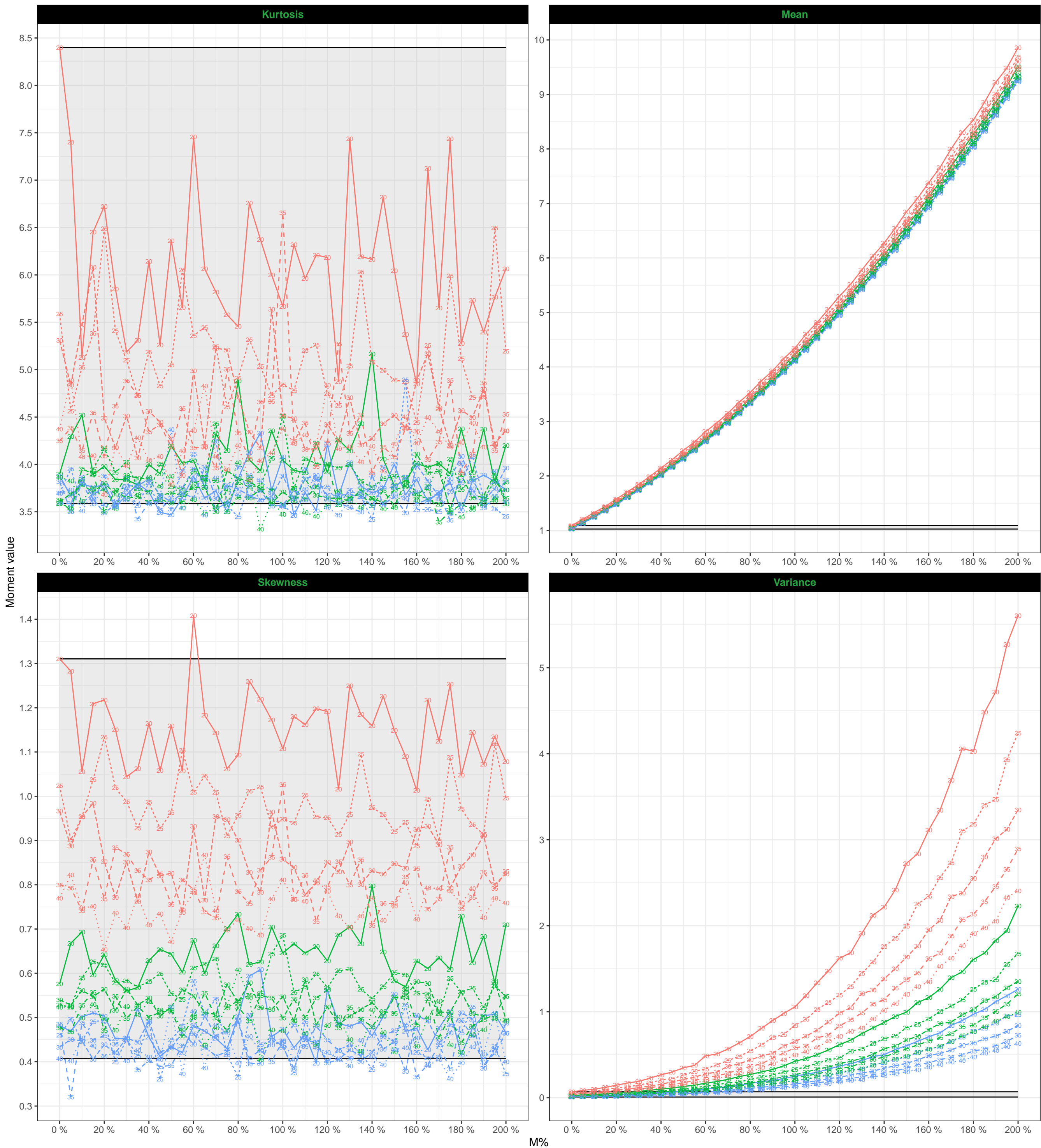

Supplement: Supplementary file 1 — Supporting Information [file BIMJ-67-e70032-s001.zip › Reproducibility resubmission v2/results pkf 22 10 2024 15 cores/Supplemental-file-repr_files/figure-latex/sixth-set-of-simulations-results-1.pdf]

R = a 2 a 3 a 4

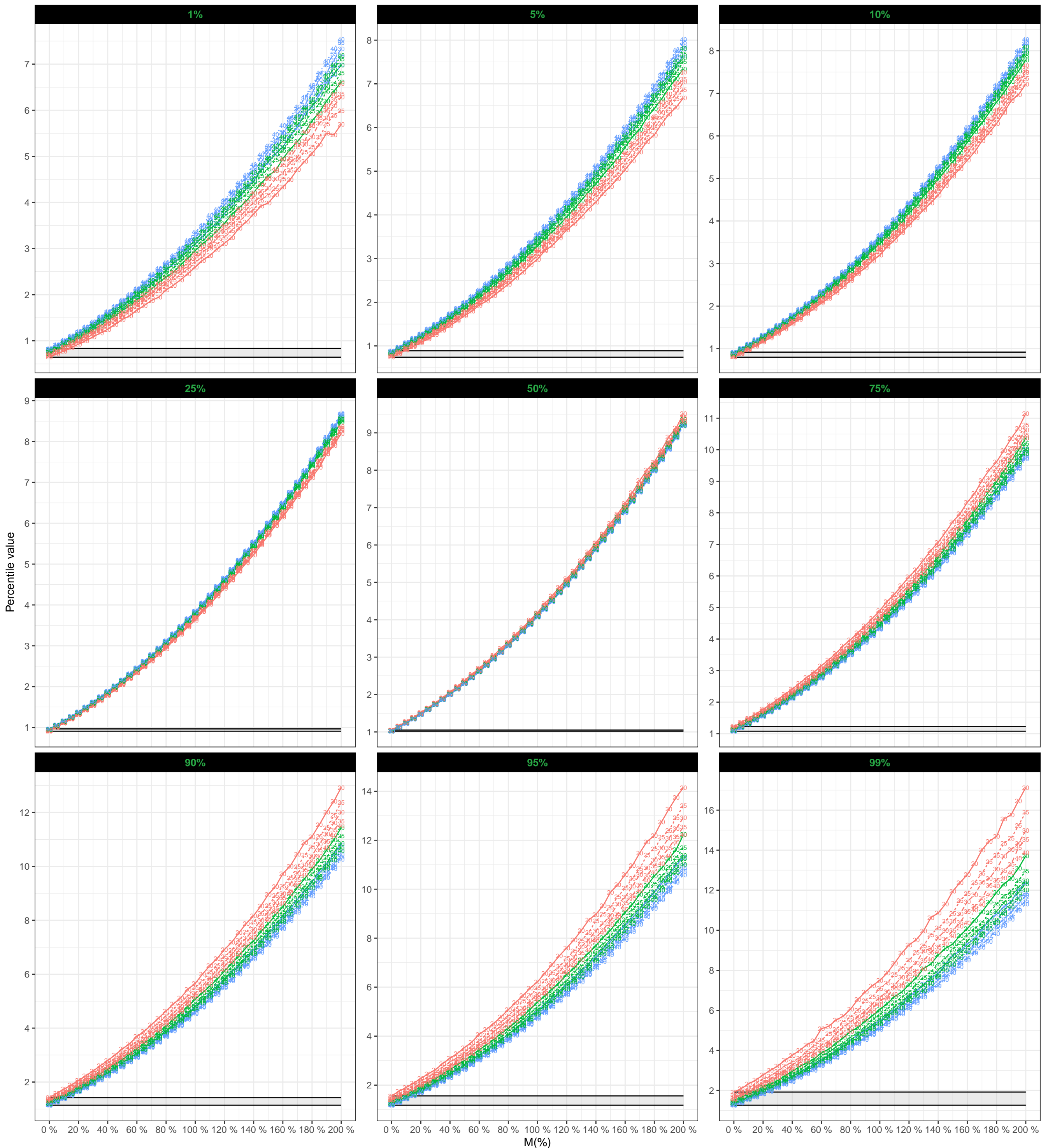

Supplement: Supplementary file 1 — Supporting Information [file BIMJ-67-e70032-s001.zip › Reproducibility resubmission v2/results pkf 22 10 2024 15 cores/Supplemental-file-repr_files/figure-latex/sixth-set-of-simulations-results-2-1.pdf]

R = a 2 a 3 a 4

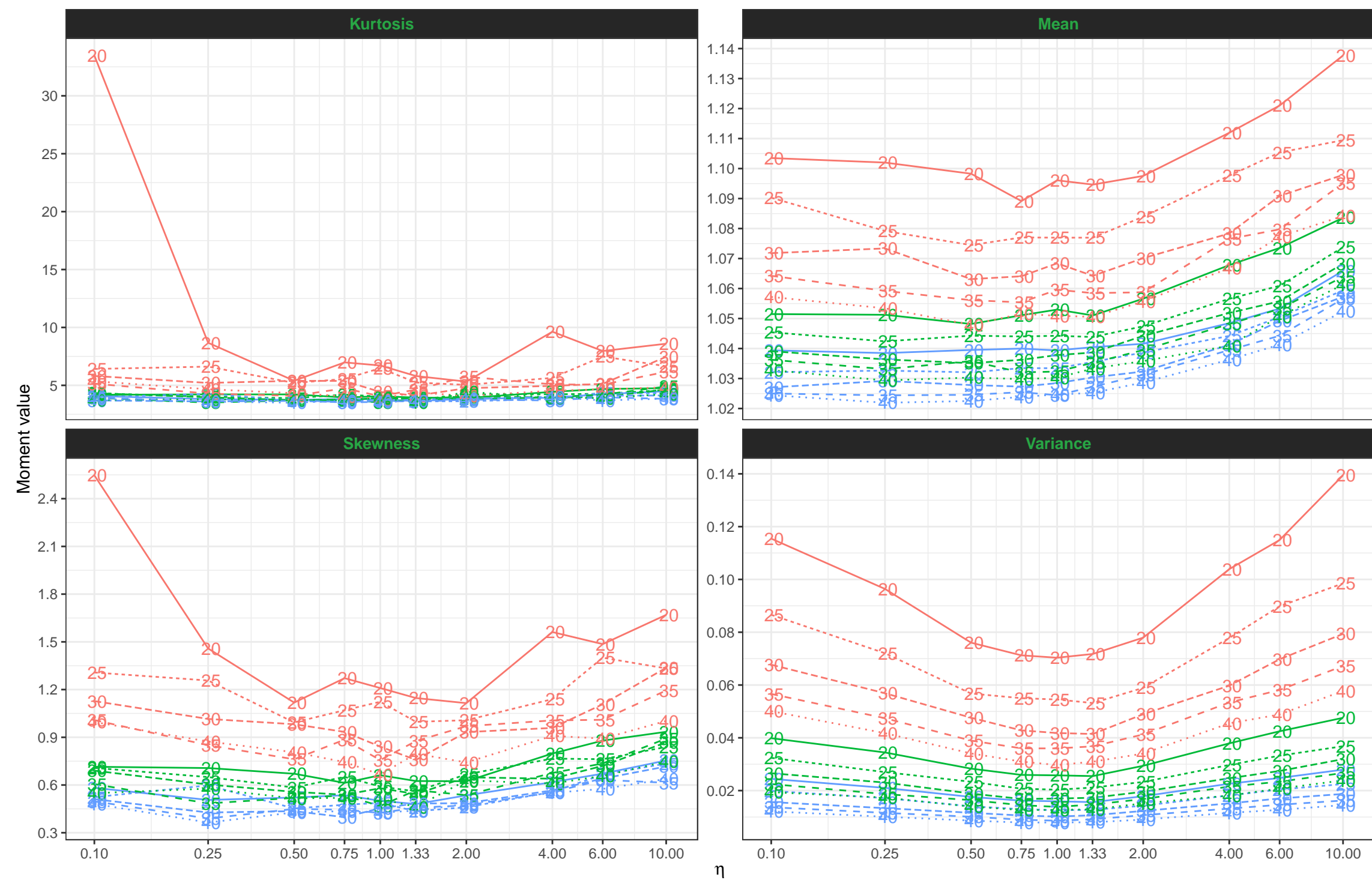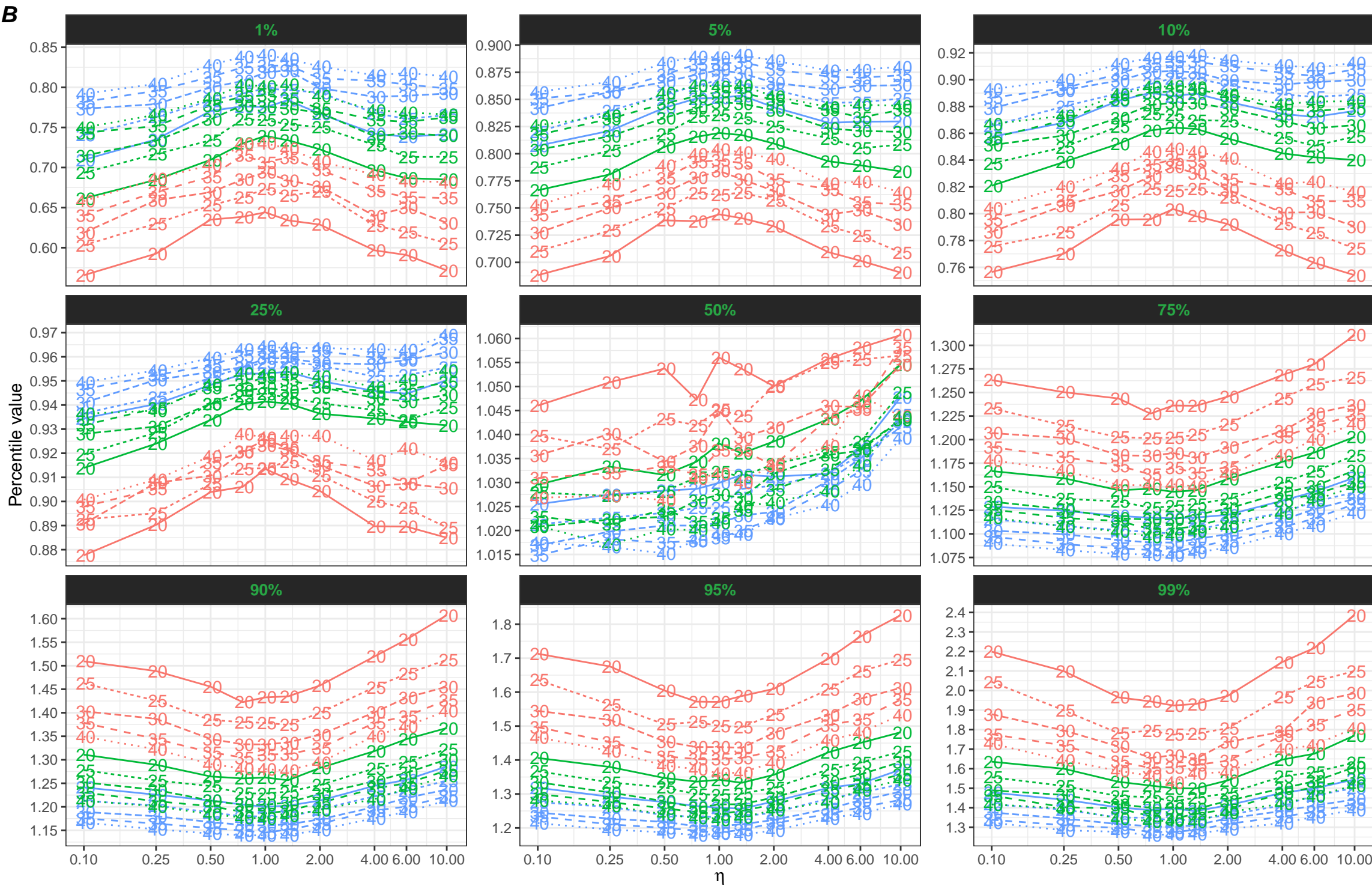

Supplement: Supplementary file 1 — Supporting Information [file BIMJ-67-e70032-s001.zip › Reproducibility resubmission v2/results pkf 22 10 2024 15 cores/Supplemental-file-repr_files/figure-latex/third-set-simulation-results-1.pdf]

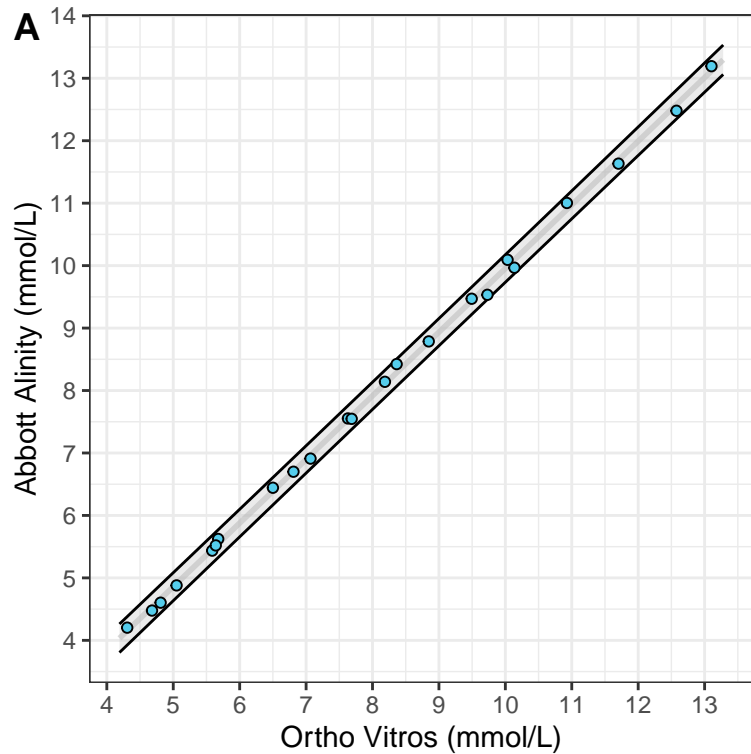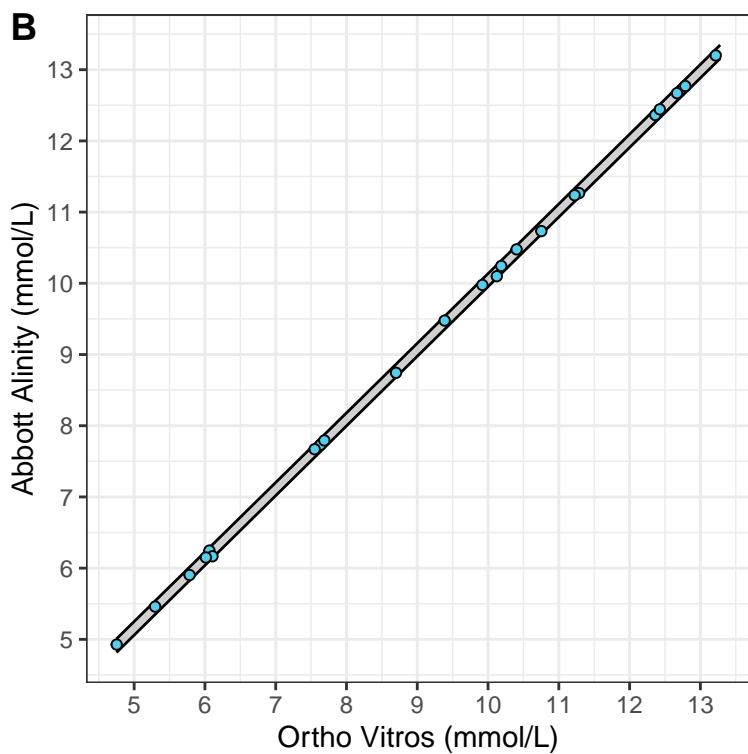

Supplement: Supplementary file 1 — Supporting Information [file BIMJ-67-e70032-s001.zip › Reproducibility resubmission v2/results pkf 22 10 2024 7 cores/Reproducing-manuscript-results_files/figure-latex/alinity-vs-vitros-1.pdf]

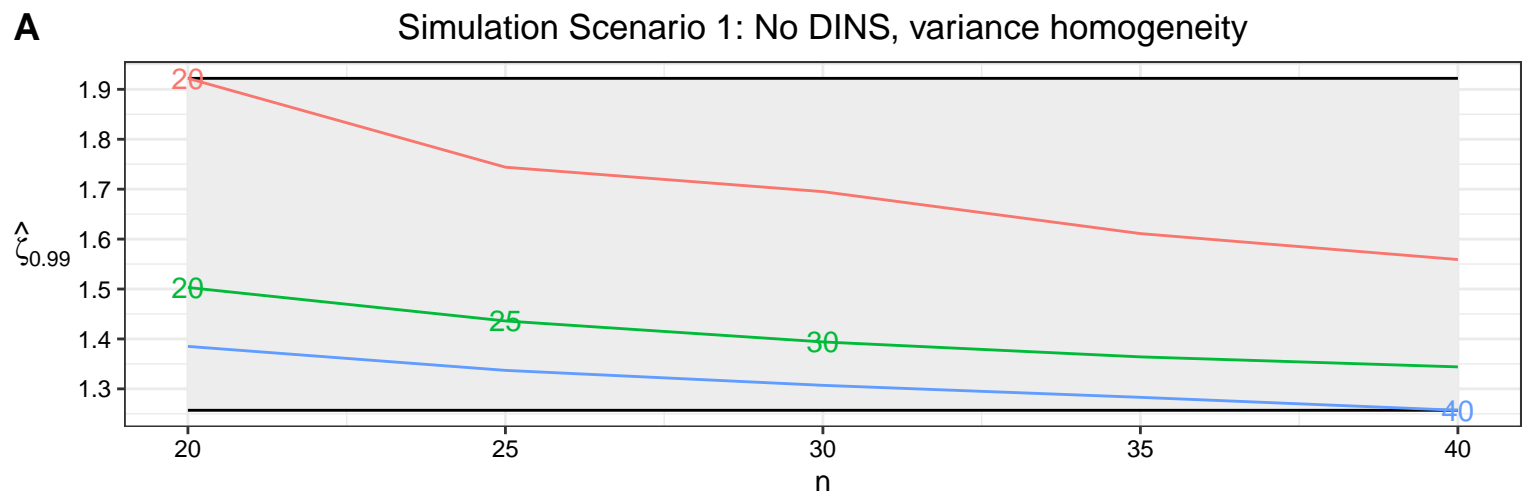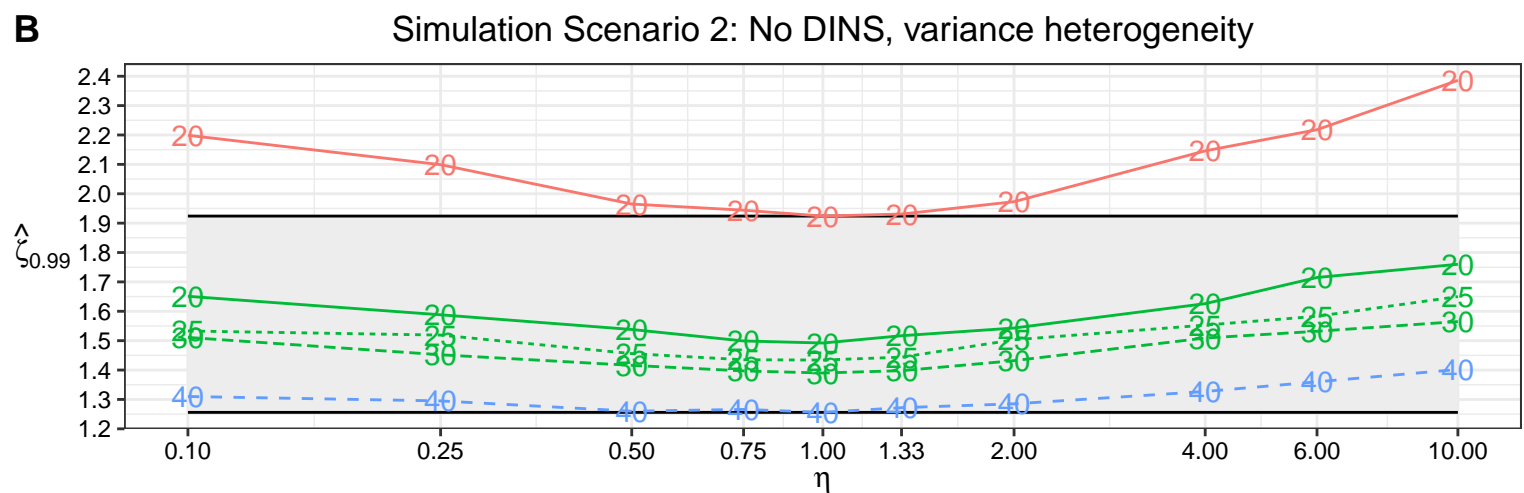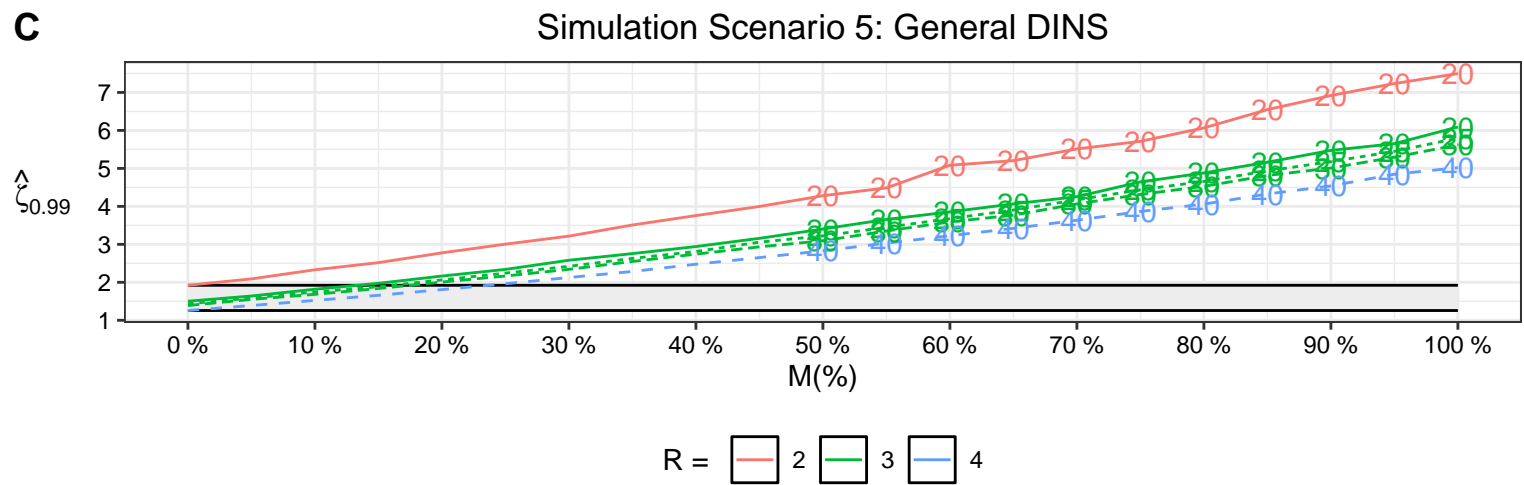

Supplement: Supplementary file 1 — Supporting Information [file BIMJ-67-e70032-s001.zip › Reproducibility resubmission v2/results pkf 22 10 2024 7 cores/Reproducing-manuscript-results_files/figure-latex/percentiles-of-zeta-1-2-5-1.pdf]

**A**

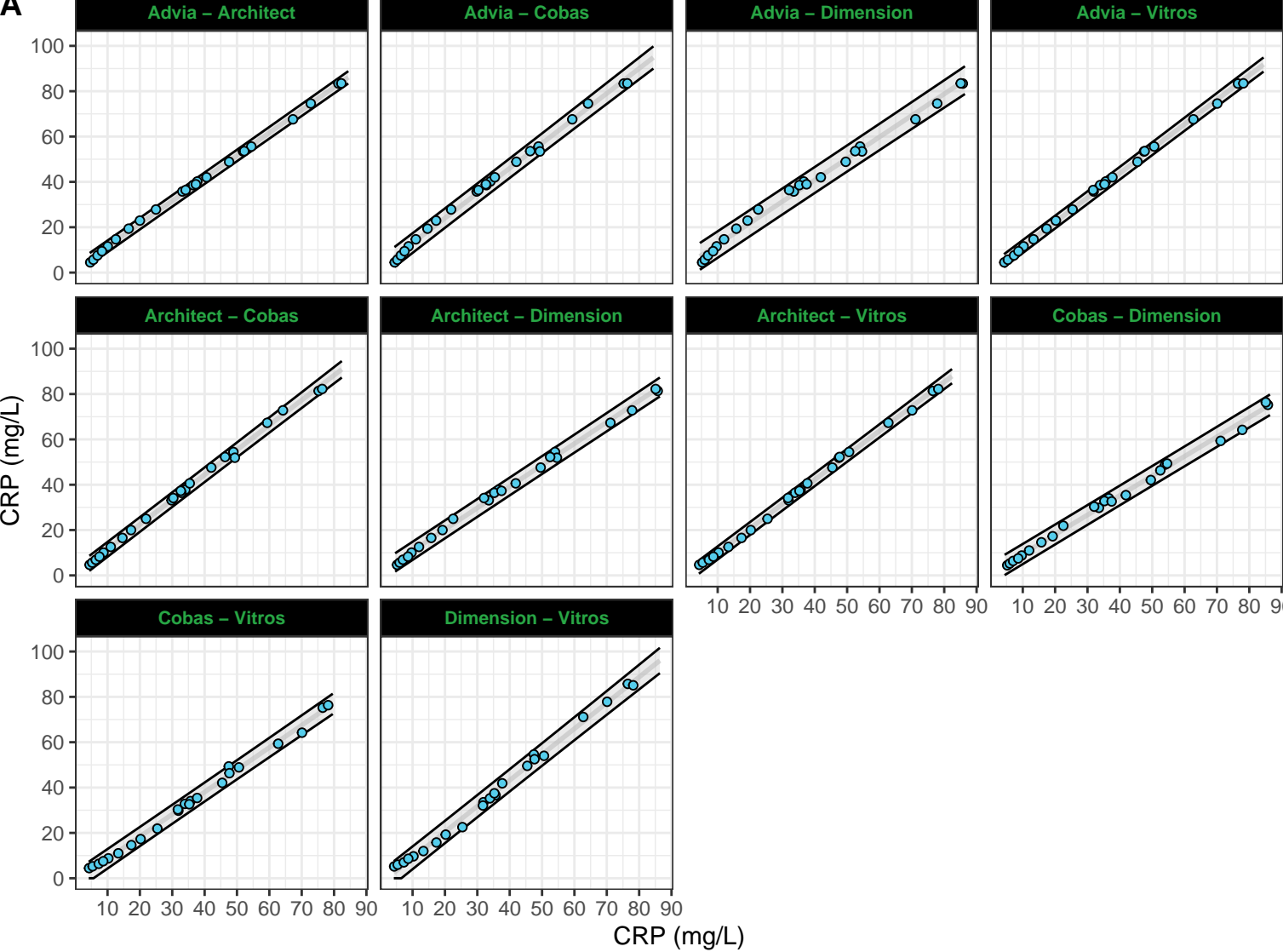

**B**

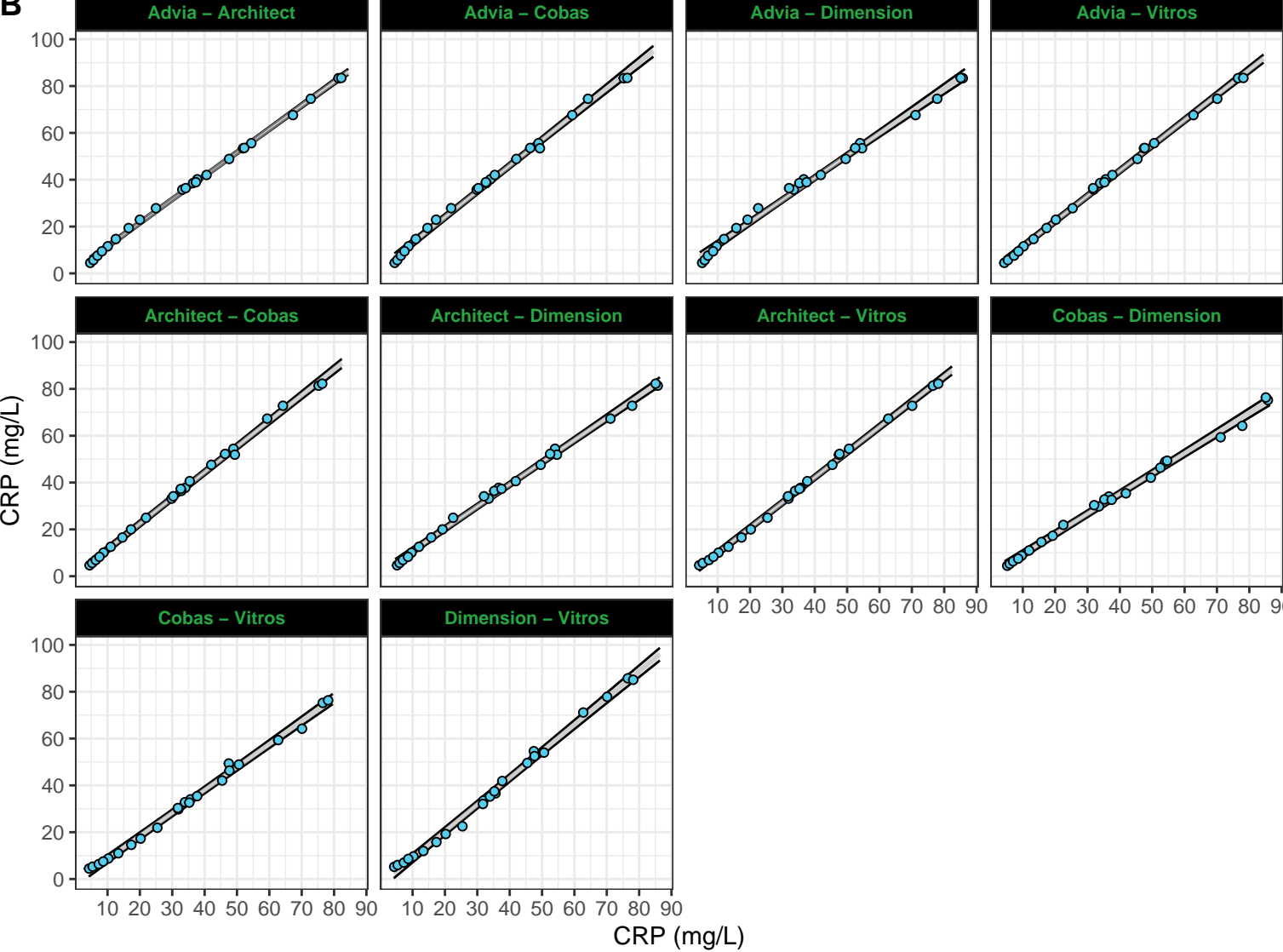

Supplement: Supplementary file 1 — Supporting Information [file BIMJ-67-e70032-s001.zip › Reproducibility resubmission v2/results pkf 22 10 2024 7 cores/Reproducing-manuscript-results_files/figure-latex/scatter-plots-for-crp-1.pdf]

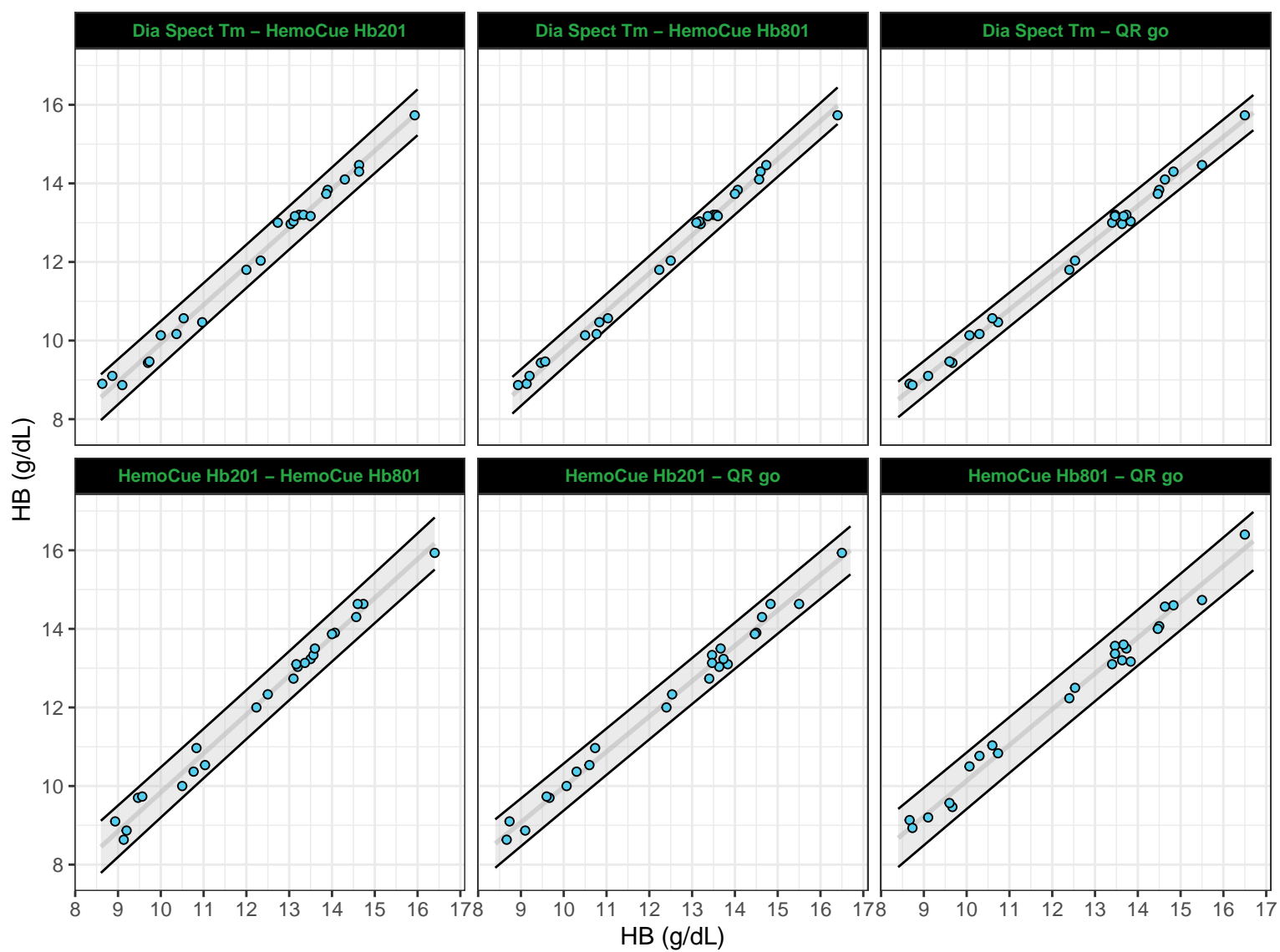

Supplement: Supplementary file 1 — Supporting Information [file BIMJ-67-e70032-s001.zip › Reproducibility resubmission v2/results pkf 22 10 2024 7 cores/Reproducing-manuscript-results_files/figure-latex/scatter-plots-for-hb-1.pdf]

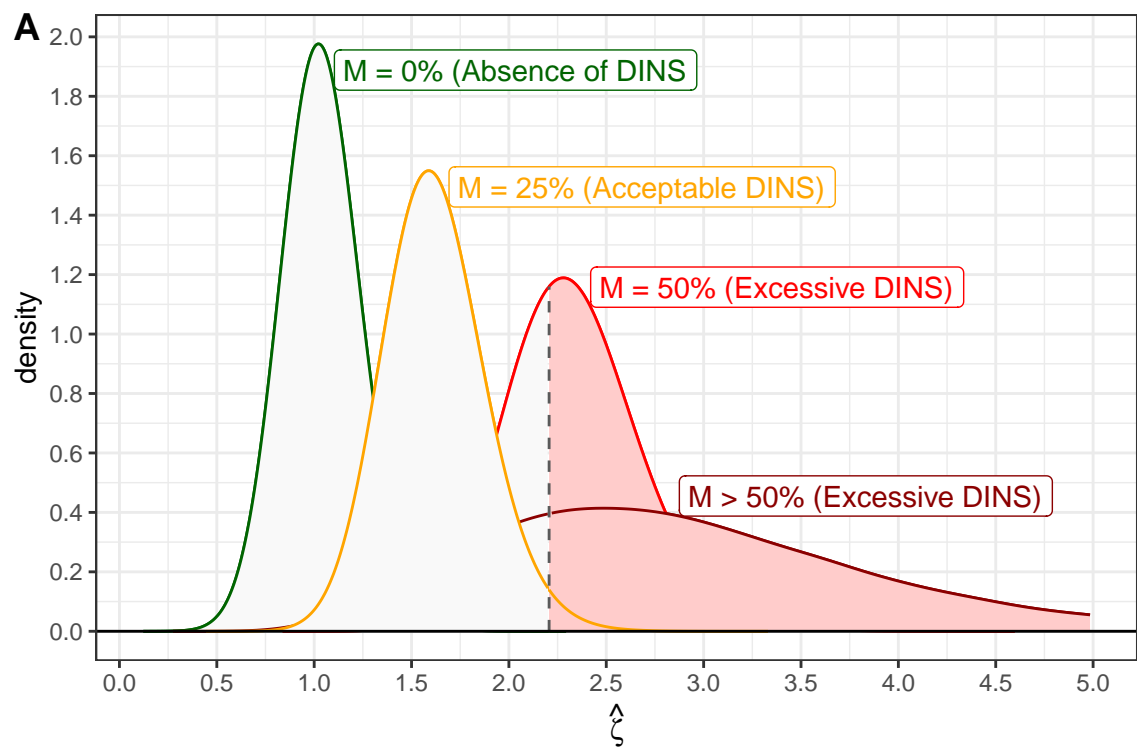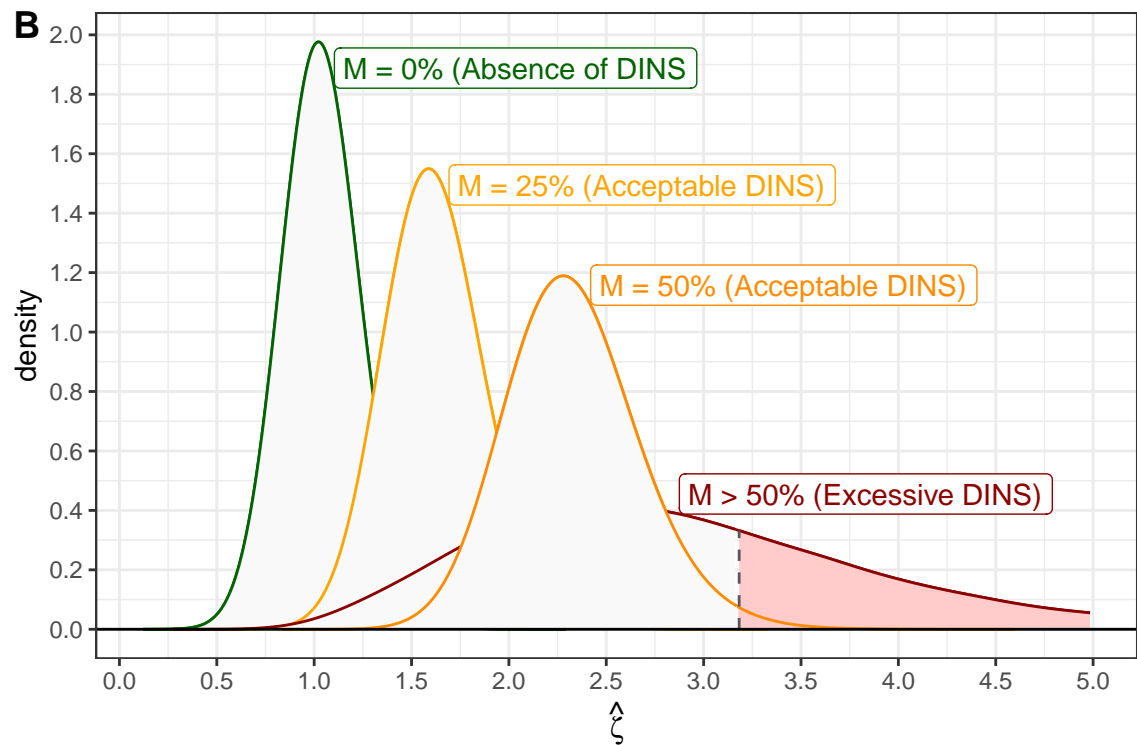

Supplement: Supplementary file 1 — Supporting Information [file BIMJ-67-e70032-s001.zip › Reproducibility resubmission v2/results pkf 22 10 2024 7 cores/Reproducing-manuscript-results_files/figure-latex/zeta-0-distribution-1.pdf]

R = a 2 a 3 a 4

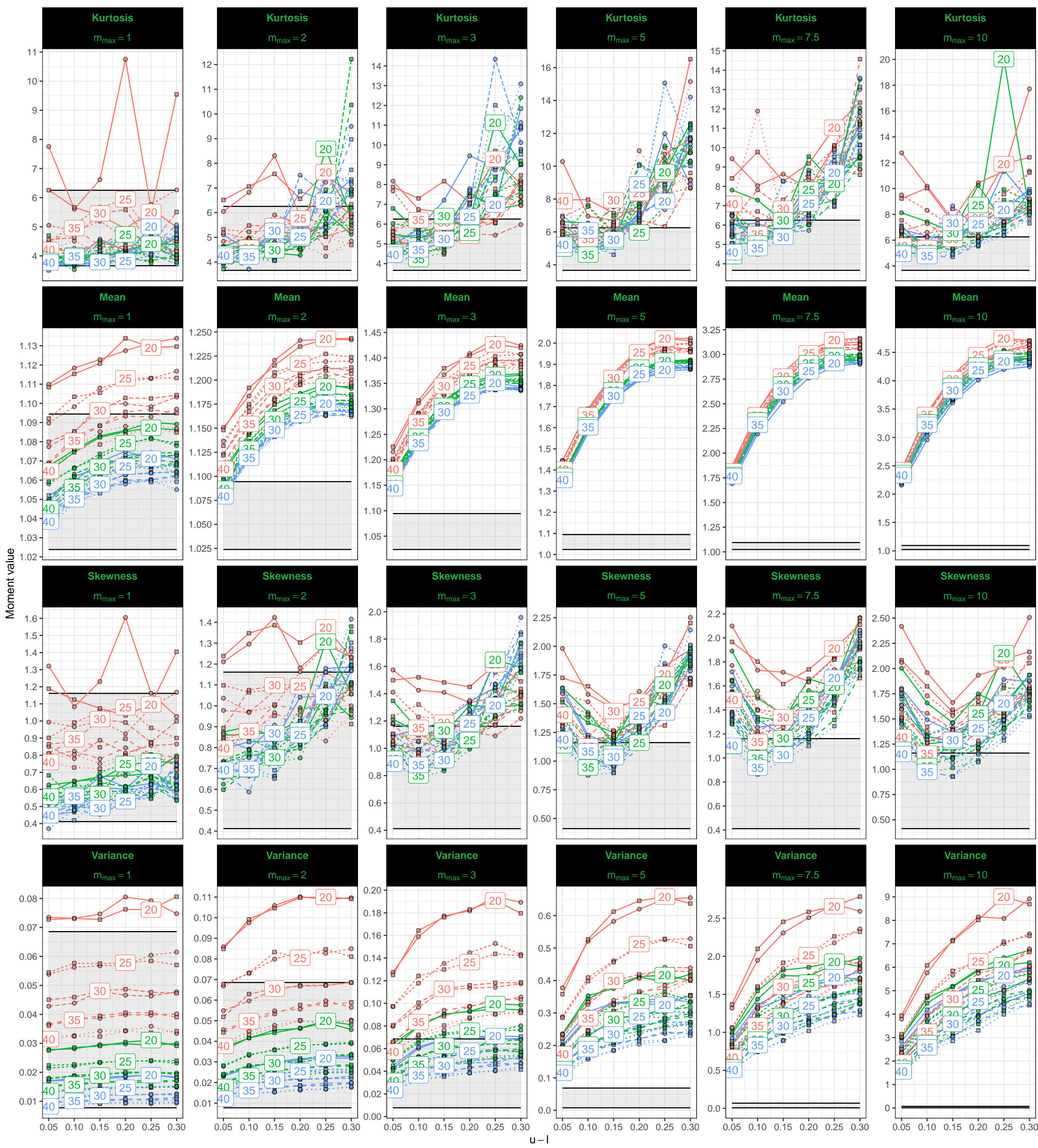

Supplement: Supplementary file 1 — Supporting Information [file BIMJ-67-e70032-s001.zip › Reproducibility resubmission v2/results pkf 22 10 2024 7 cores/Supplemental-file-repr_files/figure-latex/fifth-set-of-simulations-results-1.pdf]

**A**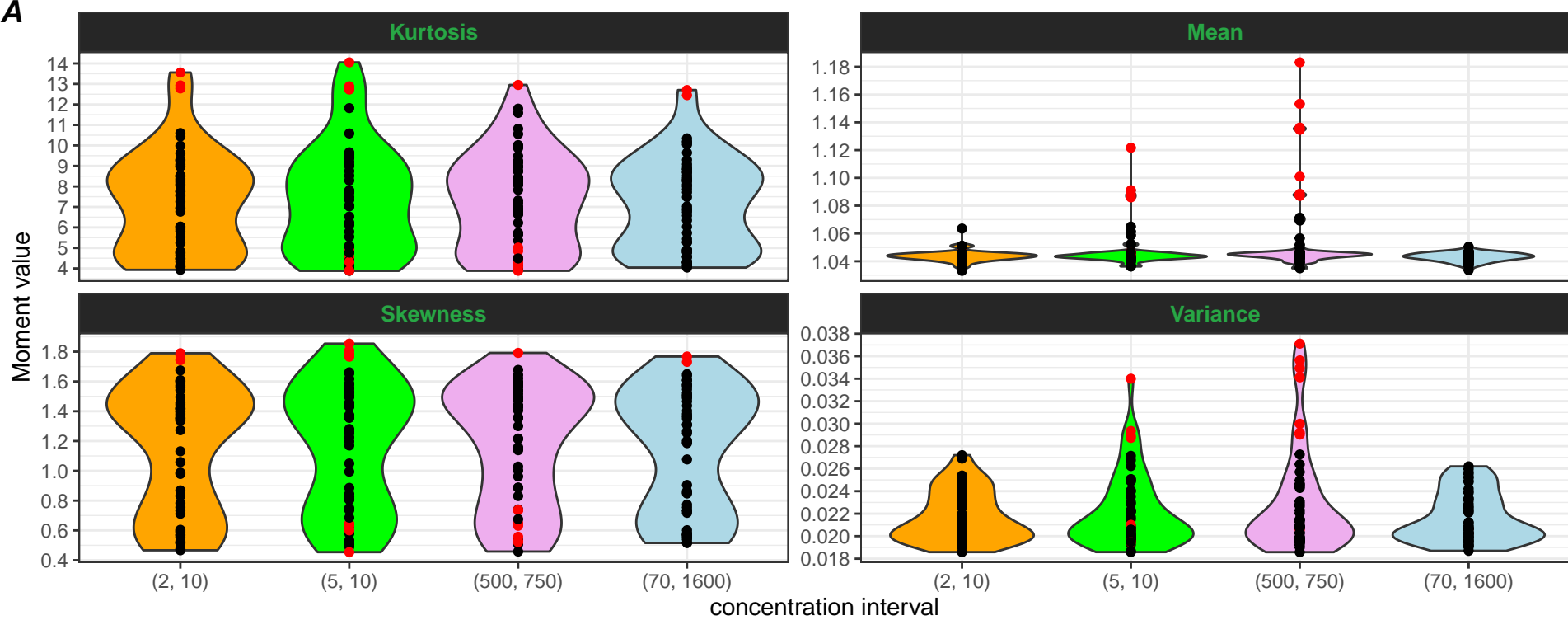**B**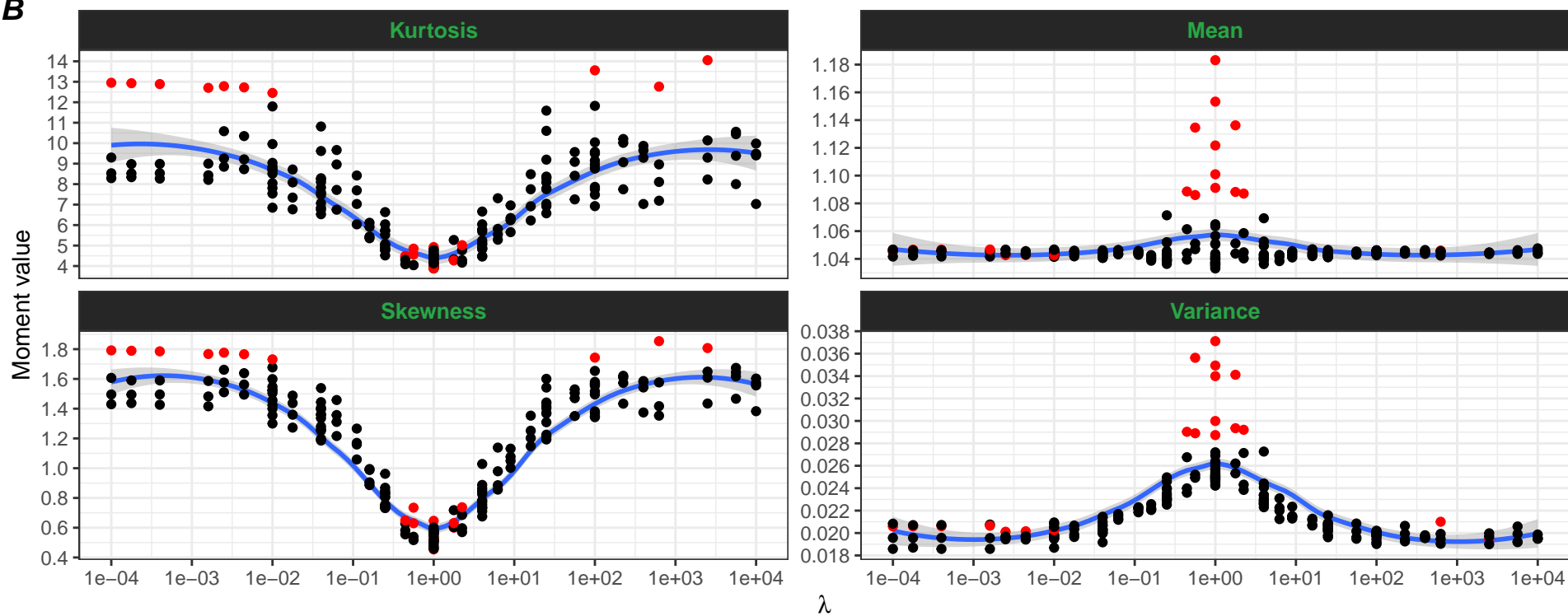

Supplement: Supplementary file 1 — Supporting Information [file BIMJ-67-e70032-s001.zip › Reproducibility resubmission v2/results pkf 22 10 2024 7 cores/Supplemental-file-repr_files/figure-latex/first-set-simulation-results-1.pdf]

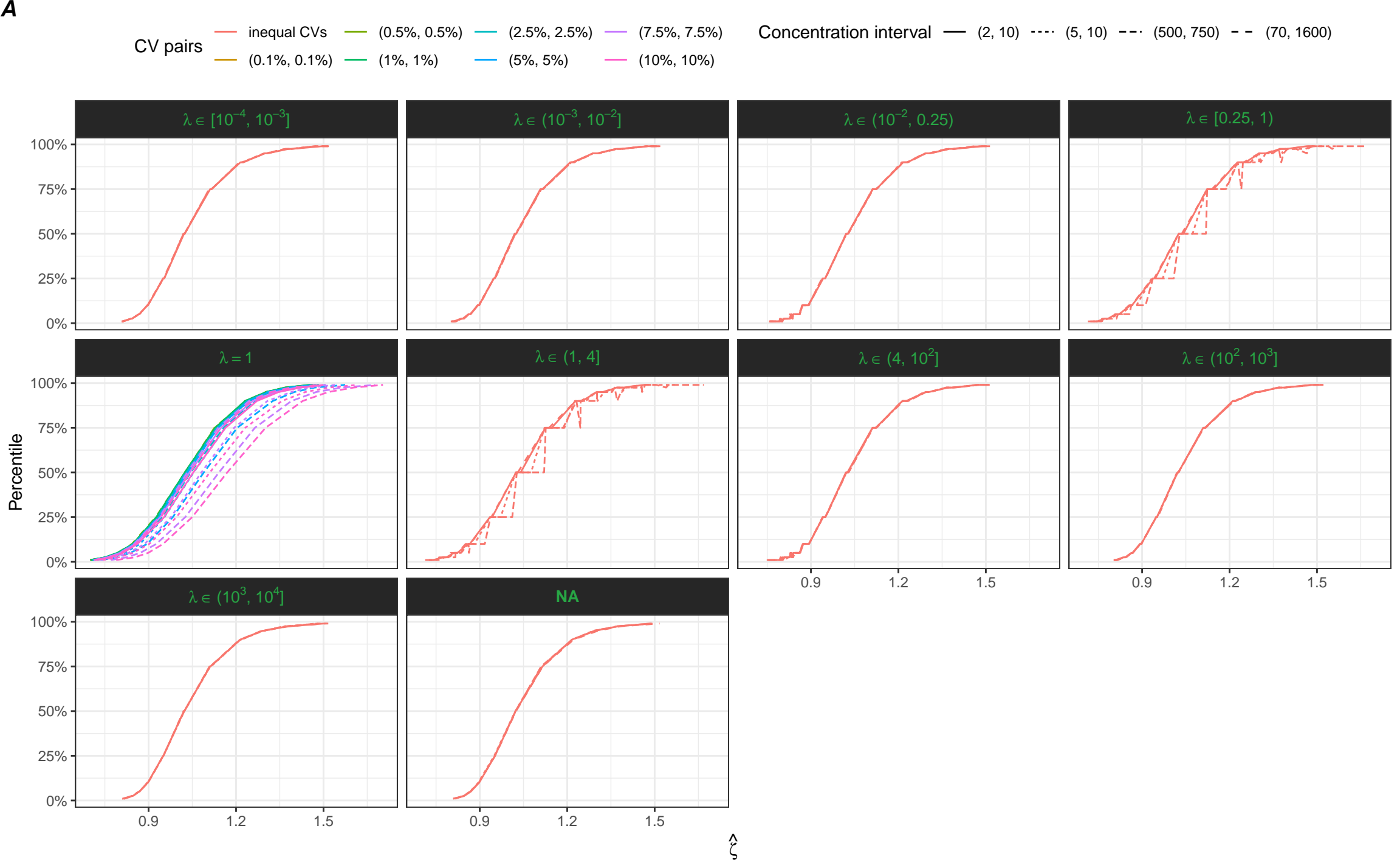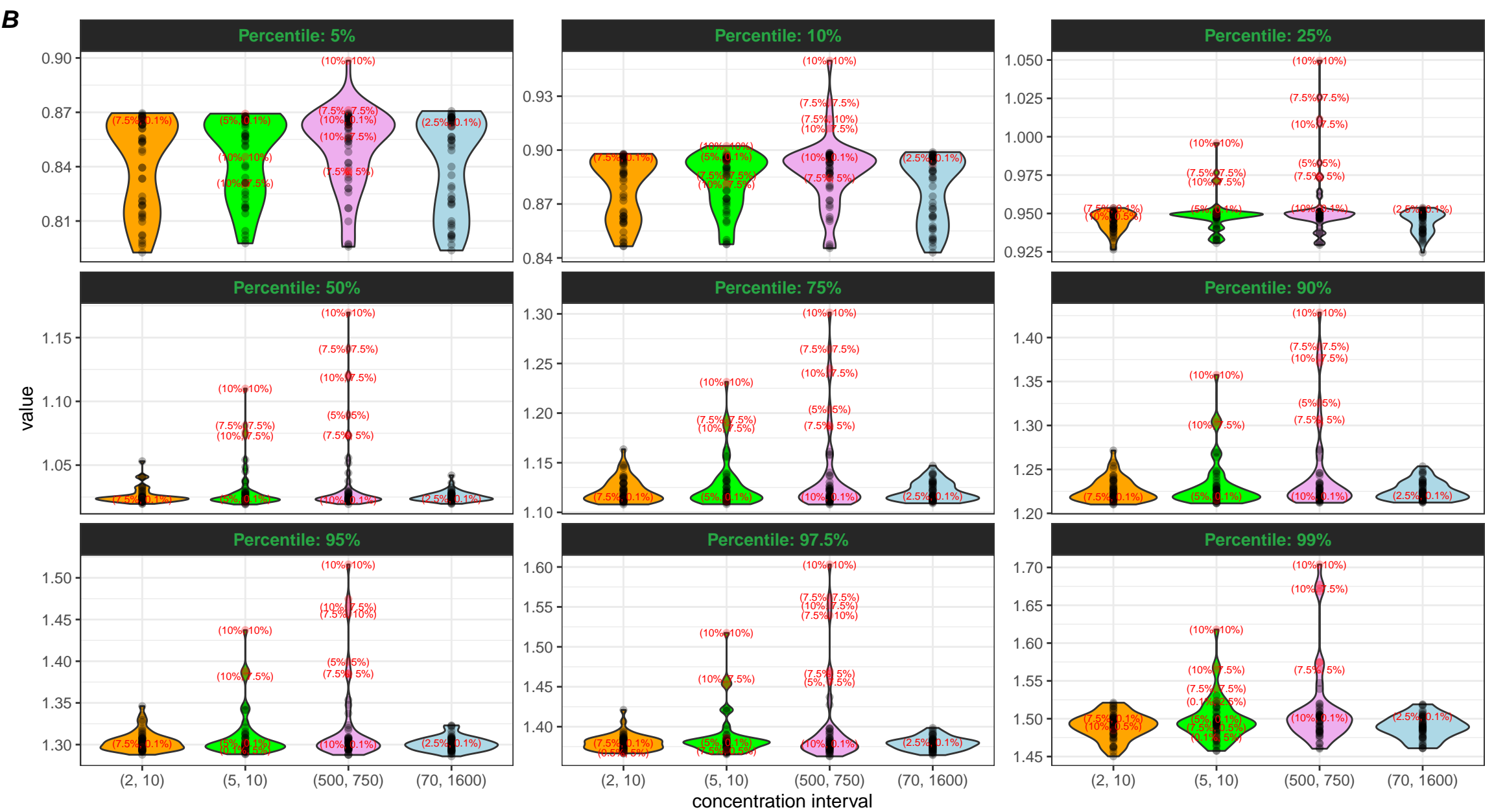

Supplement: Supplementary file 1 — Supporting Information [file BIMJ-67-e70032-s001.zip › Reproducibility resubmission v2/results pkf 22 10 2024 7 cores/Supplemental-file-repr_files/figure-latex/first-set-simulation-results-extra-1.pdf]

R = a 2 a 3 a 4

Moment value

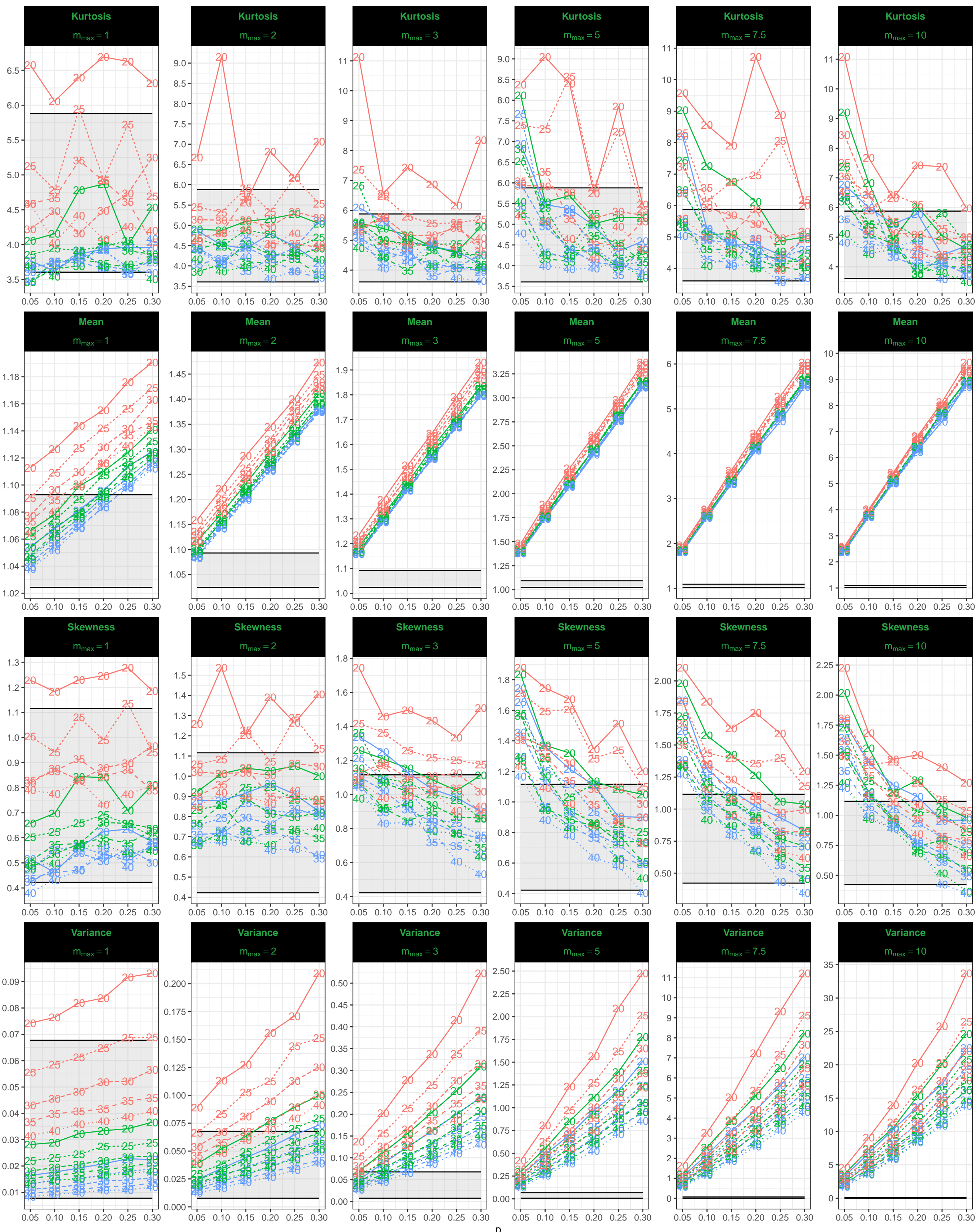

p

Supplement: Supplementary file 1 — Supporting Information [file BIMJ-67-e70032-s001.zip › Reproducibility resubmission v2/results pkf 22 10 2024 7 cores/Supplemental-file-repr_files/figure-latex/fourth-set-of-simulations-results-1.pdf]

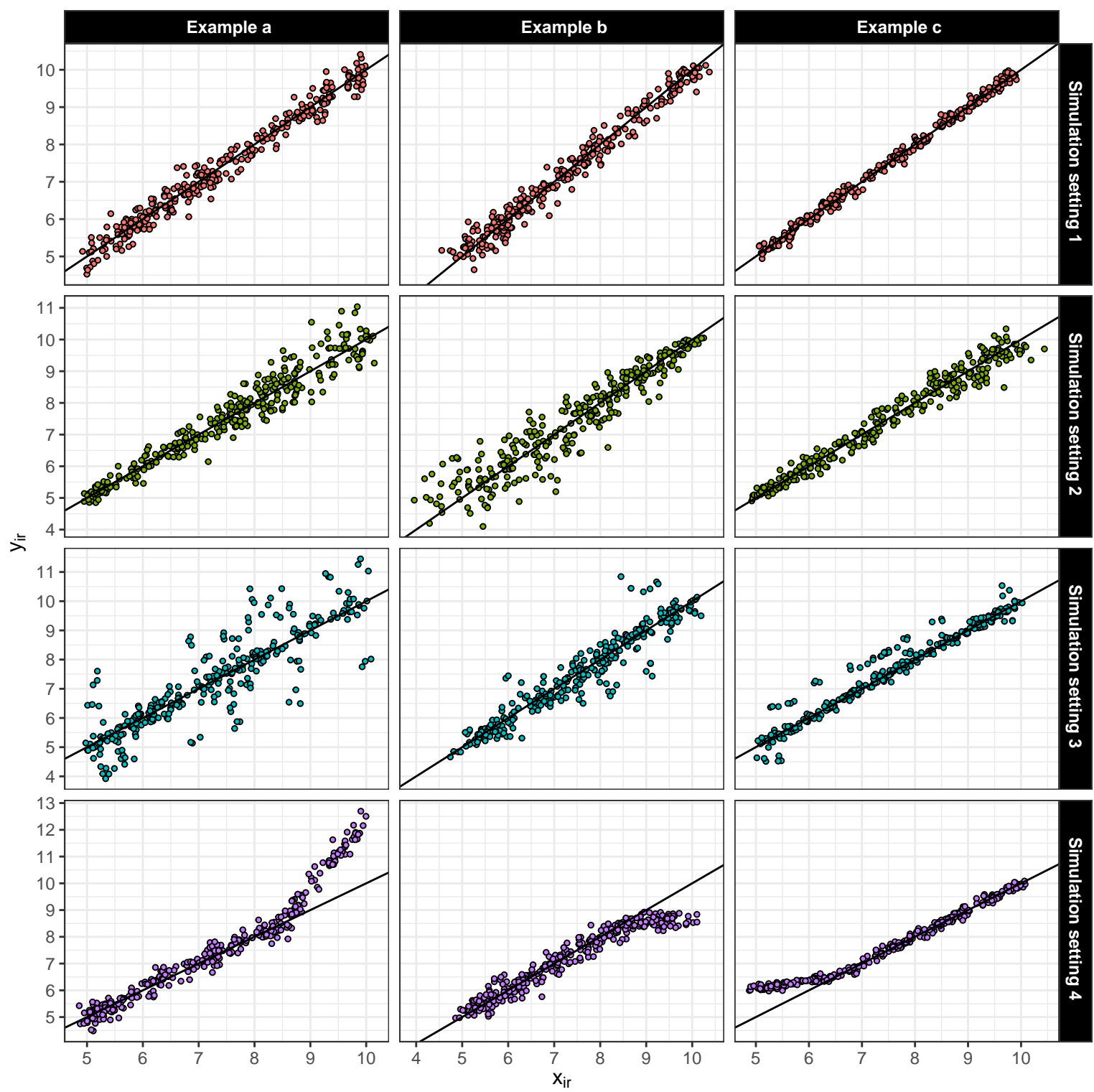

Supplement: Supplementary file 1 — Supporting Information [file BIMJ-67-e70032-s001.zip › Reproducibility resubmission v2/results pkf 22 10 2024 7 cores/Supplemental-file-repr_files/figure-latex/principle-figures-settings-1.pdf]

**-a**

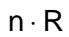

Percentile value

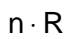

Supplement: Supplementary file 1 — Supporting Information [file BIMJ-67-e70032-s001.zip › Reproducibility resubmission v2/results pkf 22 10 2024 7 cores/Supplemental-file-repr_files/figure-latex/second-set-simulation-results-1.pdf]

R = a 2 a 3 a 4

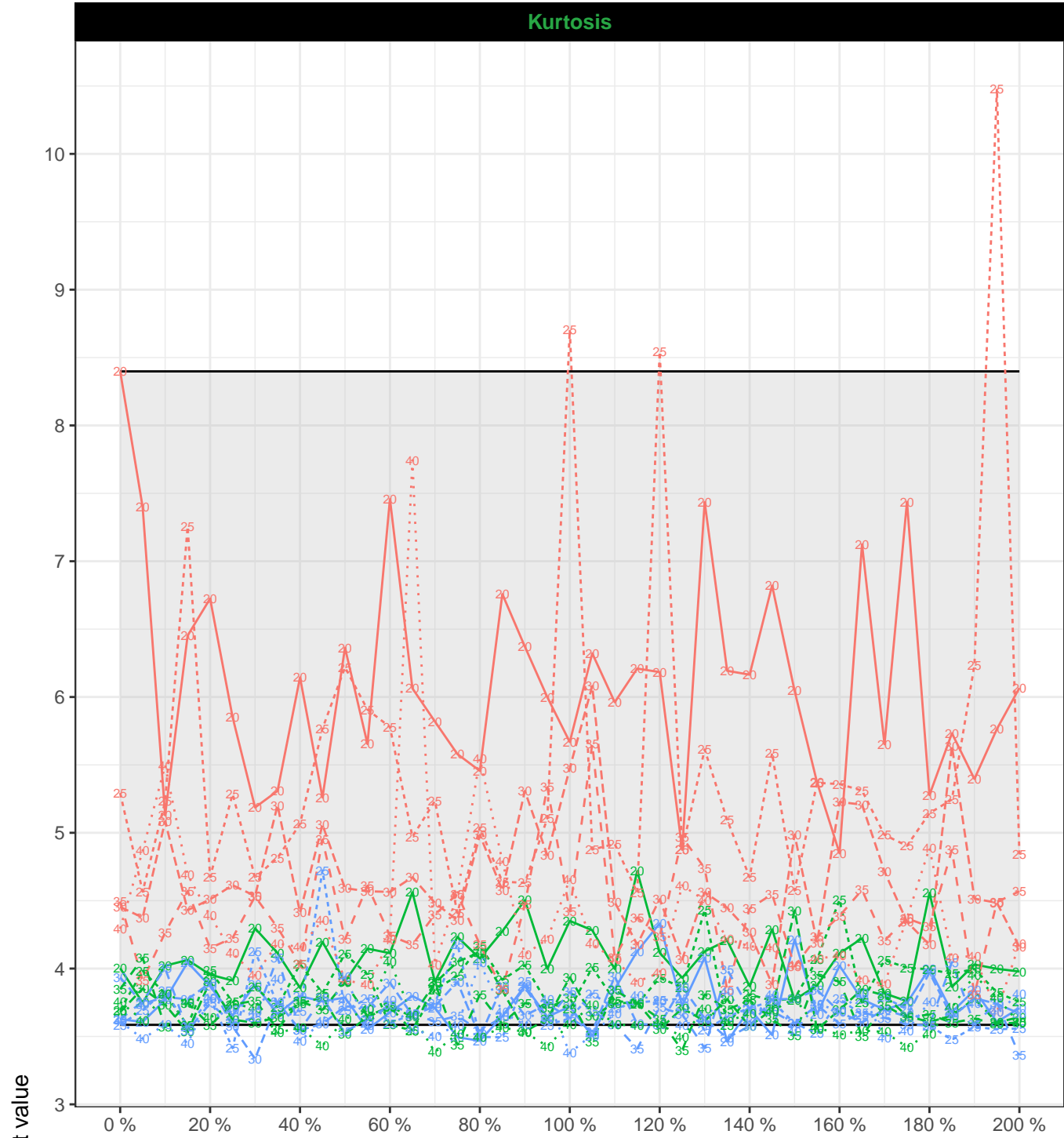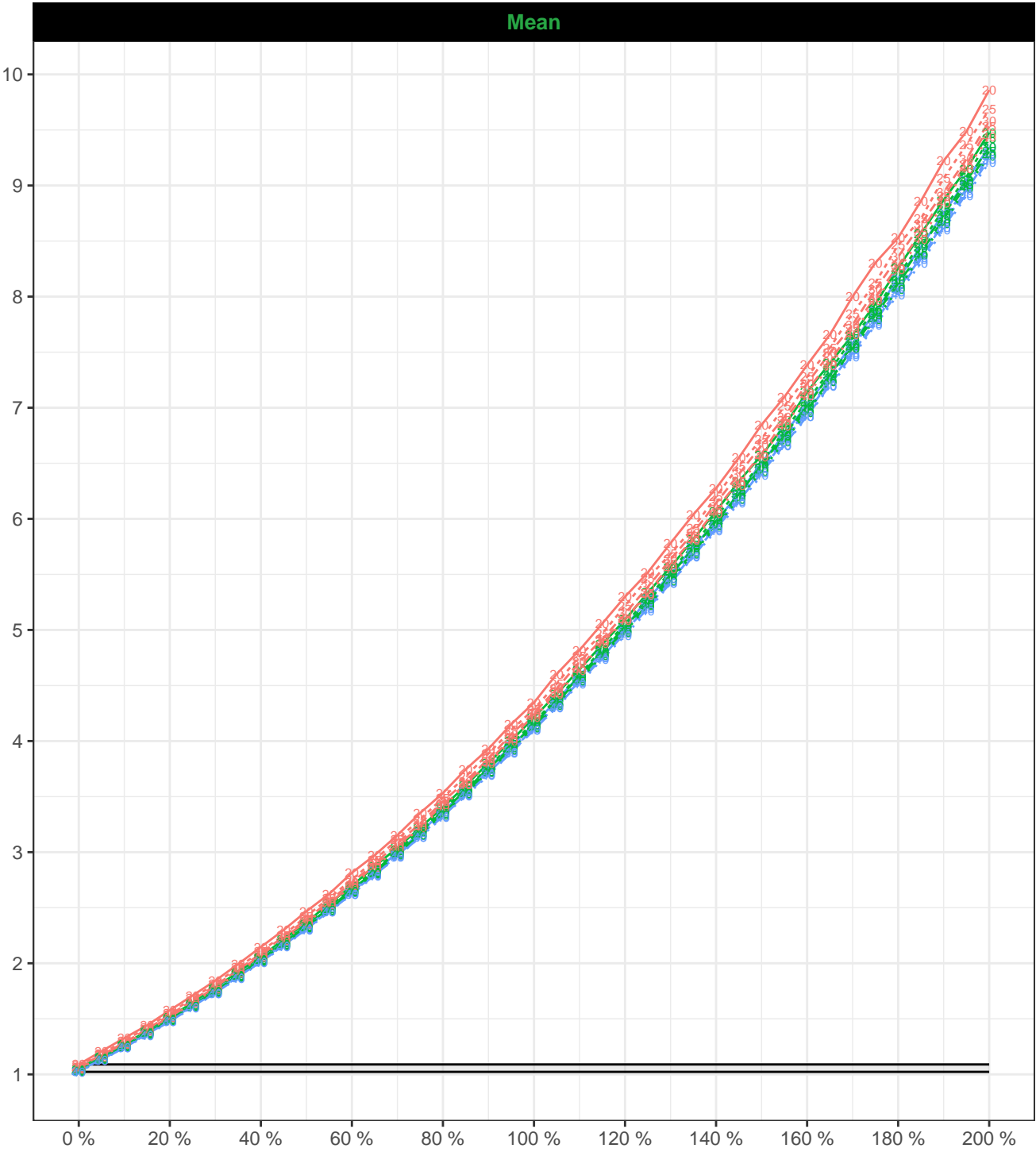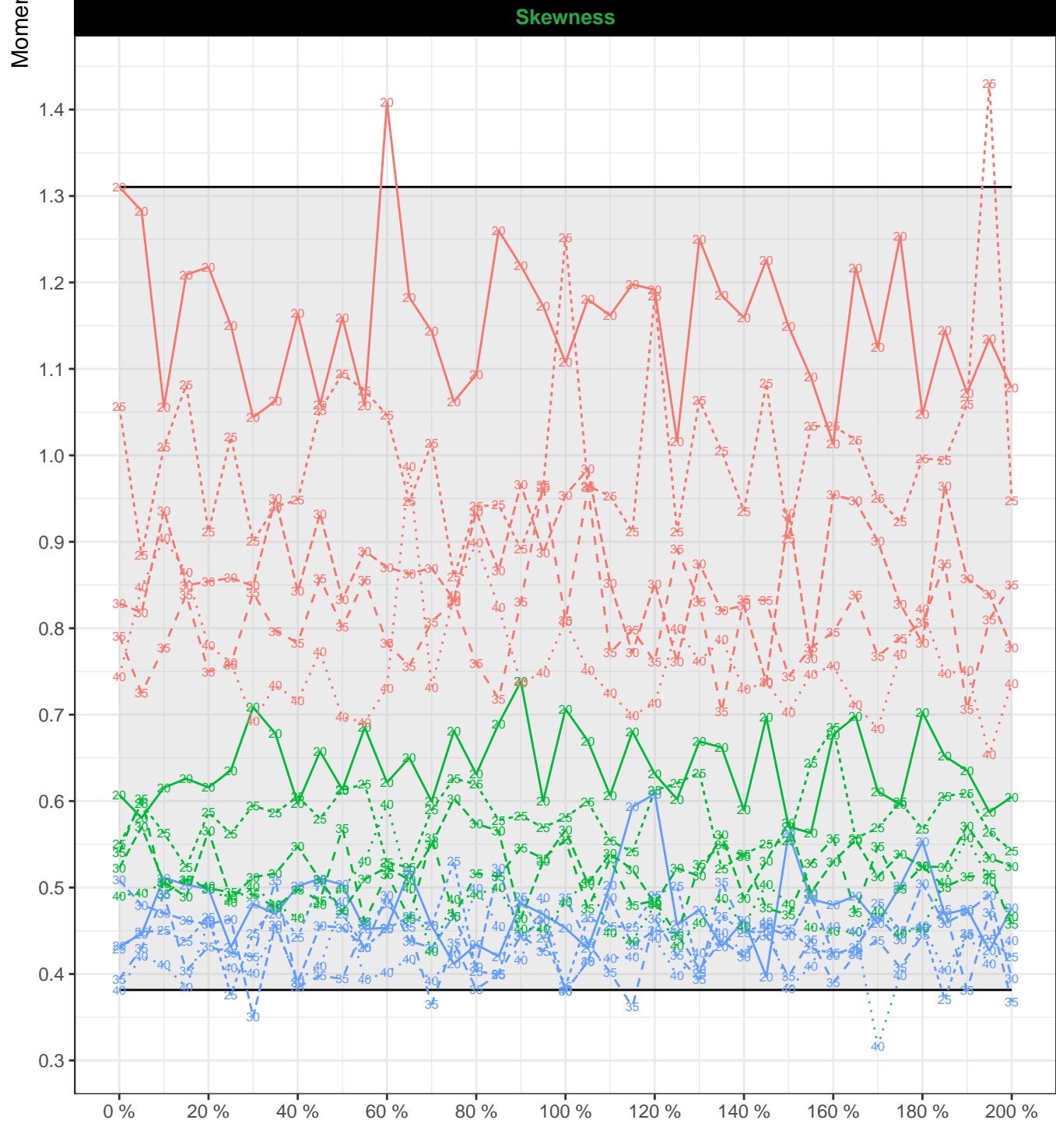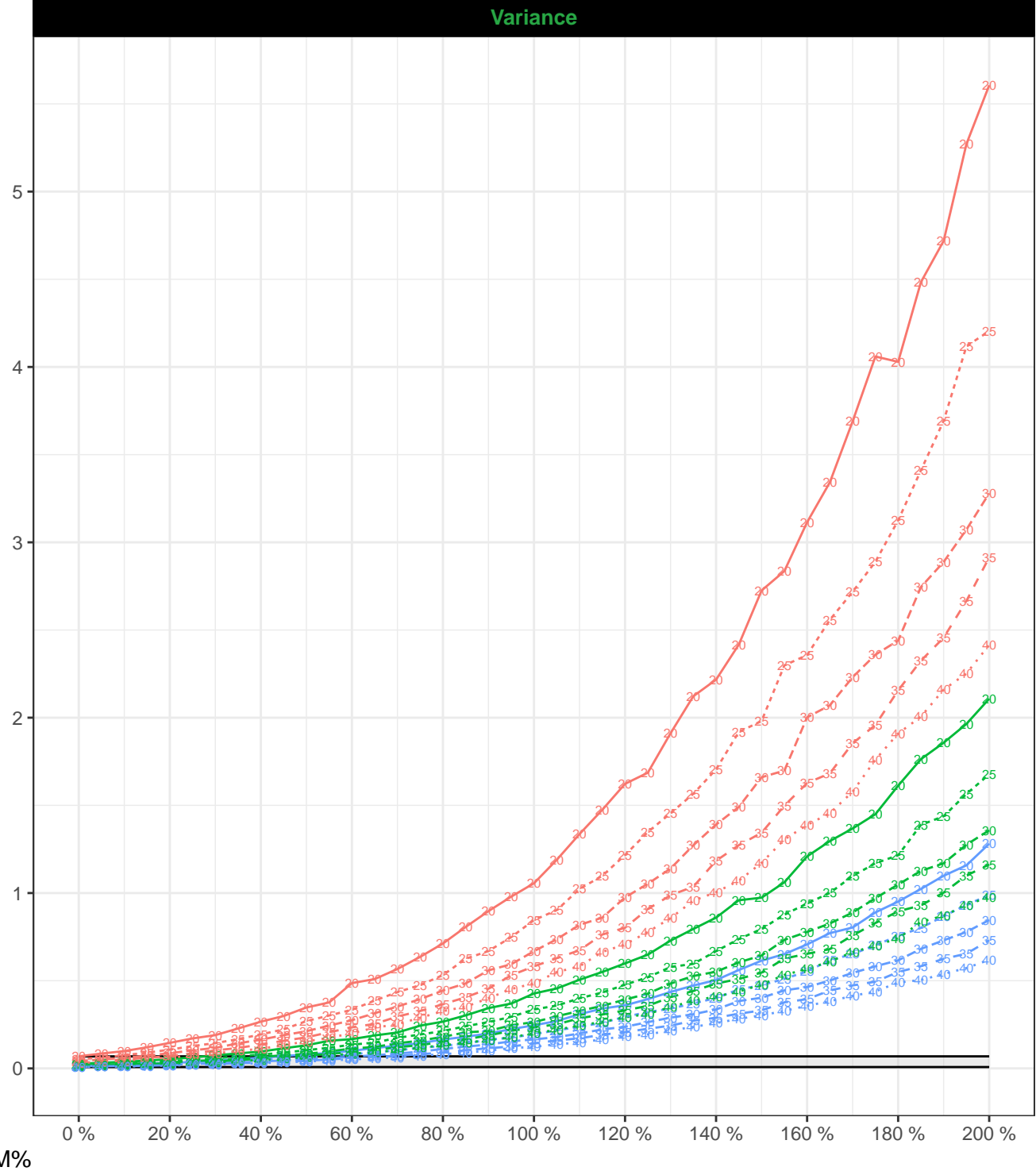

Supplement: Supplementary file 1 — Supporting Information [file BIMJ-67-e70032-s001.zip › Reproducibility resubmission v2/results pkf 22 10 2024 7 cores/Supplemental-file-repr_files/figure-latex/sixth-set-of-simulations-results-1.pdf]

R = a 2 a 3 a 4

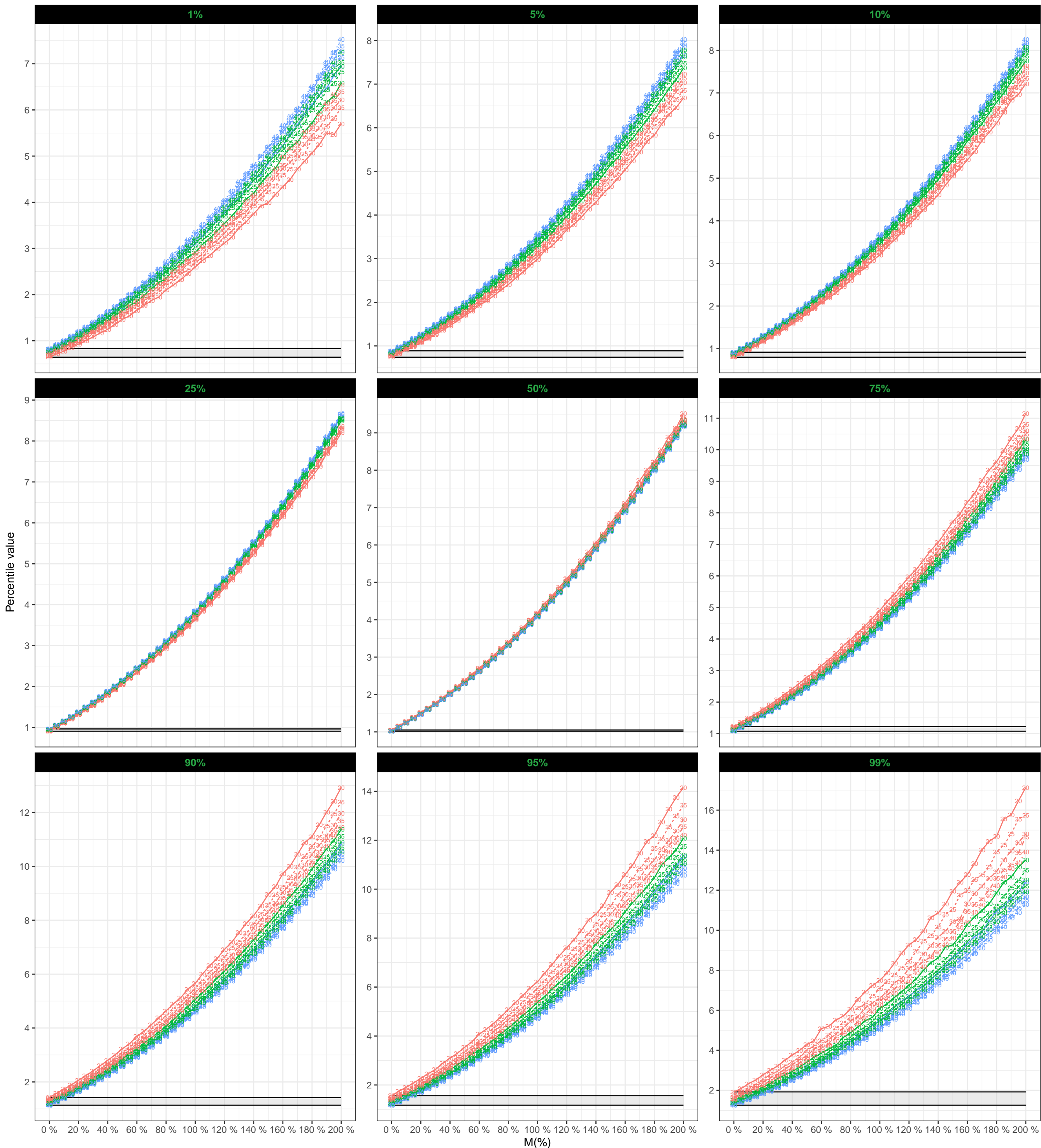

Supplement: Supplementary file 1 — Supporting Information [file BIMJ-67-e70032-s001.zip › Reproducibility resubmission v2/results pkf 22 10 2024 7 cores/Supplemental-file-repr_files/figure-latex/sixth-set-of-simulations-results-2-1.pdf]

R = a 2 a 3 a 4

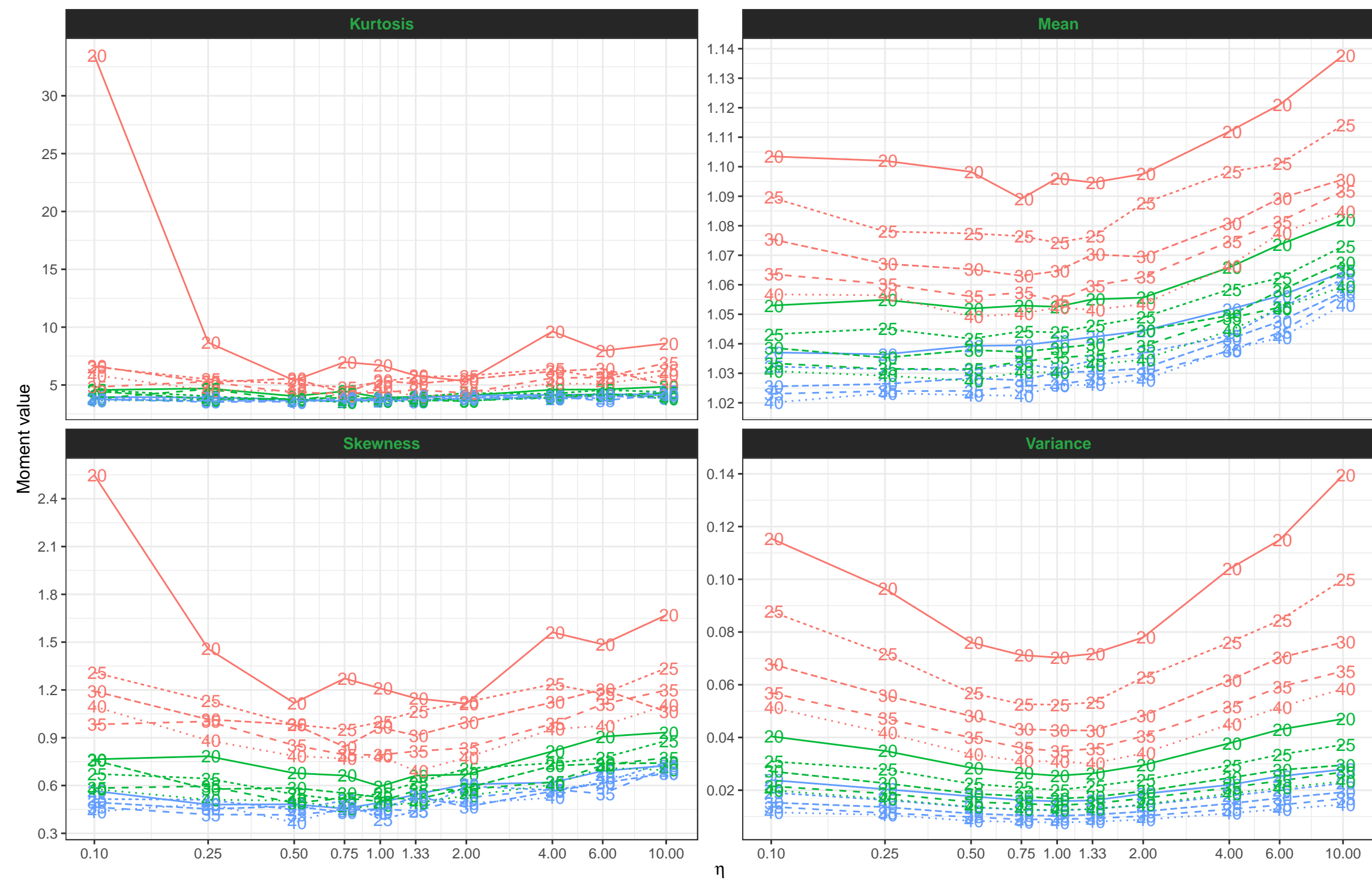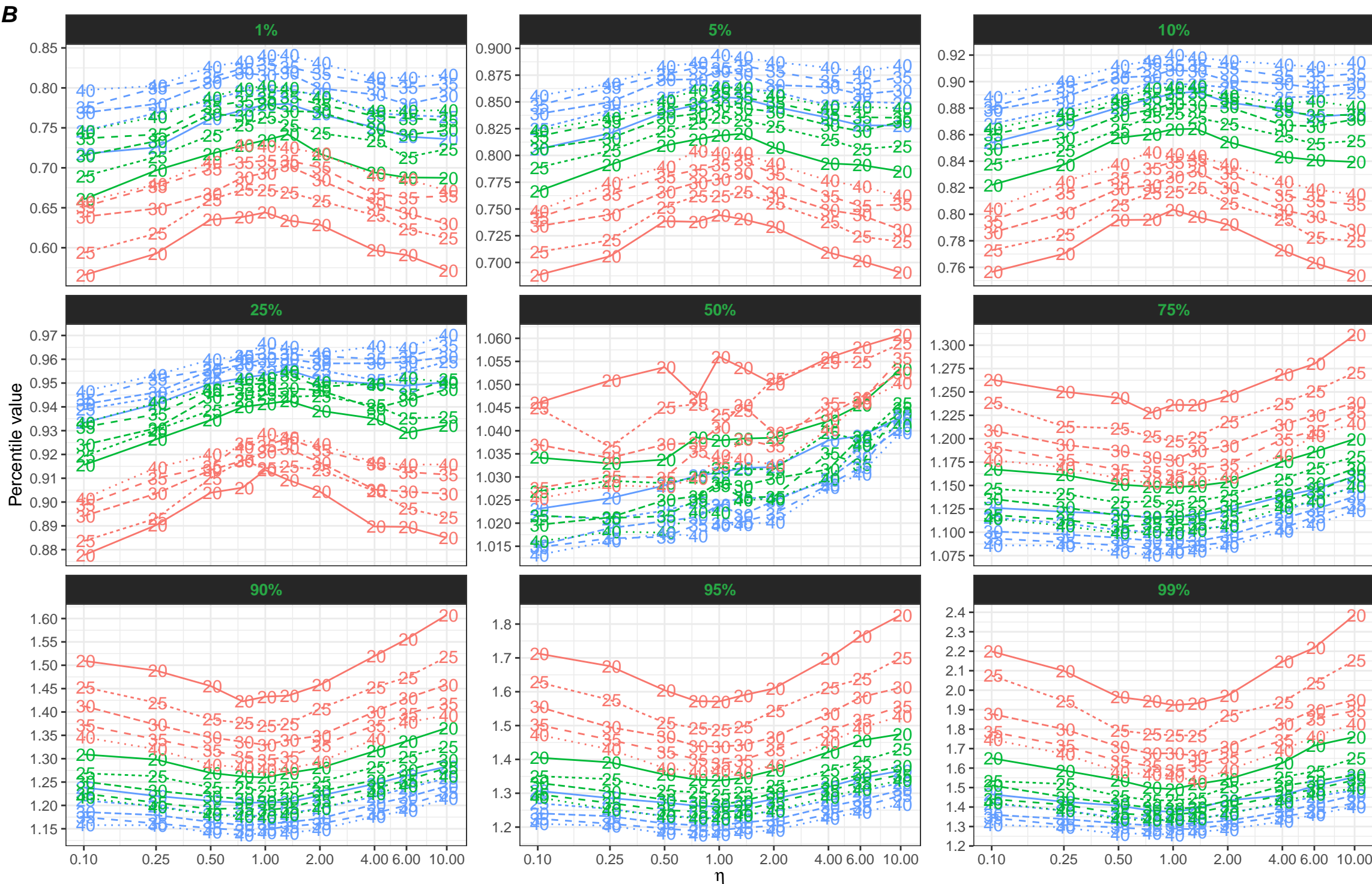

Supplement: Supplementary file 1 — Supporting Information [file BIMJ-67-e70032-s001.zip › Reproducibility resubmission v2/results pkf 22 10 2024 7 cores/Supplemental-file-repr_files/figure-latex/third-set-simulation-results-1.pdf]
